# Electrochemical CO_2_ reduction to liquid fuels: Mechanistic pathways and surface/interface engineering of catalysts and electrolytes

**DOI:** 10.1016/j.xinn.2025.100807

**Published:** 2025-01-17

**Authors:** Xueying Li, Woojong Kang, Xinyi Fan, Xinyi Tan, Justus Masa, Alex W. Robertson, Yousung Jung, Buxing Han, John Texter, Yuanfu Cheng, Bin Dai, Zhenyu Sun

**Affiliations:** 1 State Key Laboratory of Organic-Inorganic Composites, College of Chemical Engineering, Beijing University of Chemical Technology, Beijing 100029, China; 2 Department of Chemical and Biological Engineering, Institute of Chemical Processes, and Institute of Engineering Research, Seoul National University, 1 Kwanak-ro, Seoul 08826, South Korea; 3 School of Materials Science and Engineering, Beijing Institute of Technology, Beijing Key Laboratory of Environmental Science and Engineering, Beijing 100081, China; 4 Max Planck Institute for Chemical Energy Conversion, 45470 Mülheim an der Ruhr, Germany; 5 Department of Physics, University of Warwick, Coventry CV4 7AL, UK; 6 Institute of Chemistry, Chinese Academy of Sciences, Beijing 100190, China; 7 Strider Research Corporation, Rochester, NY 14610-2246, USA; 8 School of Engineering and Coating Research Institute, Eastern Michigan University, Ypsilanti, MI 48197, USA; 9 School of Chemistry and Chemical Engineering/State Key Laboratory Incubation Base for Green Processing of Chemical Engineering, Shihezi University, Shihezi 832003, China

**Keywords:** carbon dioxide, reduction, electrocatalysis, liquid chemicals, mechanism, catalyst design

## Abstract

The high energy density of green synthetic liquid chemicals and fuels makes them ideal for sustainable energy storage and transportation applications. Electroreduction of carbon dioxide (CO_2_) directly into such high value-added chemicals can help us achieve a renewable C cycle. Such electrochemical reduction typically suffers from low faradaic efficiencies (FEs) and generates a mixture of products due to the complexity of controlling the reaction selectivity. This perspective summarizes recent advances in the mechanistic understanding of CO_2_ reduction reaction pathways toward liquid products and the state-of-the-art catalytic materials for conversion of CO_2_ to liquid C_1_ (e.g., formic acid, methanol) and C_2+_ products (e.g., acetic acid, ethanol, *n*-propanol). Many liquid fuels are being produced with FEs between 80% and 100%. We discuss the use of structure-binding energy relationships, computational screening, and machine learning to identify promising candidates for experimental validation. Finally, we classify strategies for controlling catalyst selectivity and summarize breakthroughs, prospects, and challenges in electrocatalytic CO_2_ reduction to guide future developments.

## Public summary

• Electrocatalytic CO_2_ reduction (ECR) into liquid chemicals can achieve a renewable carbon cycle.
• Reaction pathways for different liquid products have been assigned.
• Recent experimental and theoretical developments of ECR have been summarized.
• Design strategies for advanced ECR catalysts have been presented.

## Graphical abstract

**Figure:**
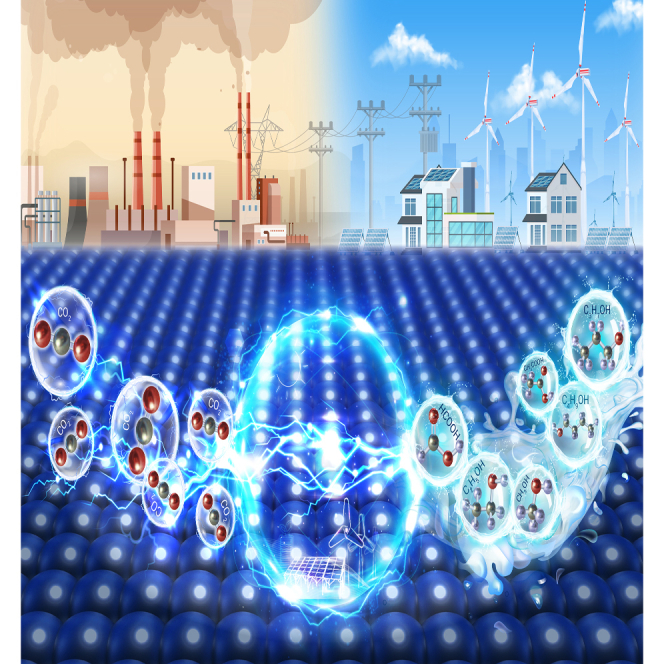

## Introduction

The concentration of carbon dioxide (CO_2_) in the atmosphere has dramatically increased due to the steady rise in fossil fuel usage leading to severe environmental and climate impacts like melting glaciers, rising sea levels, and record-high temperatures.^1^ According to statistics from the National Oceanic and Atmospheric Administration (NOAA), as of January 2024, the concentration of CO_2_ in the global atmosphere has reached 422.16 ppm, an increase of 2.85 ppm compared to 2023.^2^ The Intergovernmental Panel on Climate Change (IPCC) of the United Nations predicts that, by 2100, atmospheric CO_2_ levels will reach 570 ppm, the global average temperature will rise by 1.9°C, and, in the worst-case scenario, sea levels will rise by at least 2 m.^3^

CO_2_ capture, utilization, and storage (CCUS) technology is considered to be one of the key means to mitigate net CO_2_ emissions from the energy industry.^4^^,^^5^ Currently, 35 commercial CO_2_ capture facilities operate worldwide, capturing nearly 45 million tonnes (Mt) of CO_2_ per year. Innovative CO_2_ utilization technologies make it possible to convert captured and stored CO_2_ into valuable fuels or chemical feedstocks, thereby offsetting part of the operational costs of CCUS.^6^ At present, the thermo-catalytic conversion of CO_2_ into important chemicals and fuels has already been implemented on an industrial scale.^7^^,^^8^ However, the burning of fuels during thermo-catalytic CO_2_ transformation significantly affects the C footprint of these processes.^9^

In contrast, the use of electrocatalytic CO_2_ reduction (ECR) is more attractive, as it can be performed under mild environmental conditions and uses renewably generated electricity to convert CO_2_ into portable liquid fuels or fuel precursors.^10^ However, many of the chemicals currently obtained by ECR reactions are also produced in the operationally lower-cost fossil feedstock industry, which is one of the challenges that faces the commercialization of ECR technologies, yet its promise remains realized by governments, with the US Department of Energy recently announcing increased funding to electrochemical CO_2_ conversion.^11^

Great progress has been made in the development and understanding of catalytic materials for electrochemical conversion of CO_2_ into different C_1_–C_3_ liquid products such as formic acid/formate (HCOOH/HCOO^−^),^12^ ethanol (C_2_H_5_OH),^13^ and *n*-propanol (C_2_H_5_CH_2_OH).^14^ However, there remain challenges in producing liquid products through ECR, including controlling the reaction pathway to selectively generate specific C-based products, the parasitic competition with the hydrogen evolution reaction (HER) prevalent in aqueous media, and the need for product separation, which is inevitably necessary yet often overlooked. Current state-of-the-art catalysts allow the production of C_1_ products with more than 95% faradaic efficiency (FE; the percentage of faradaic charge utilized to generate a specific product) and C_2_ products with about 60% FE, while the FE of C_3_ compounds is limited to around 10%.^15^ Further issues include the large kinetic overpotentials (∼1.0 V) required to induce product formation at a meaningful rate^16^ and ensuring long-term catalyst stability.

Therefore, an in-depth exploration of the current state of knowledge regarding the reaction pathways for the conversion of CO_2_ to liquid fuels and chemicals during ECR is timely to inform rationally designed and effective catalysts and ECR systems to solve the above challenges. This review explores CO_2_ conversion pathways to liquid fuels and chemicals via ECR, summarizing the recent progress, strategies for improving catalyst performance for liquid products, and the key challenges and prospects for future development.

## Mechanistic insights into CO_2_ reduction

The wide product variability of ECR on different catalyst surfaces, its potential-dependent product selectivity, and structure-sensitive kinetics underscore the complexity of deciphering and controlling ECR pathways. Below, we summarize the ECR pathways for a range of liquid products.

### Possible reaction pathways of ECR toward liquid products

The electrochemical reduction of CO_2_ is a multi-step reaction that involves multiple electrons and protons, and it is strongly influenced by the catalyst surface properties. Key steps include diffusion of CO_2_ to the active sites, CO_2_ adsorption and activation, possible co-activation of reducing agents (H_2_, H_2_O, or cofactors), consecutive or concerted coupled transfer of electrons and protons to CO_2_ forming oxygenated intermediates, and desorption of by-products and products. Initial CO_2_ adsorption is often the limiting step and should thus be enhanced. Other crucial processes are H_2_ activation by catalysts, CO_2_ reaction with a cofactor (usually NADH) in enzymatic catalysts, and competing H_2_O reduction in photocatalysis and electrocatalysis.

A particularly kinetically demanding step is the first electron transfer to CO_2_, which requires a catalyst for the one-electron reduction of CO_2_ to create the CO_2_^·−^ radical anion, a requisite step for ECR toward particular products, which requires overcoming a high energy barrier to permit solvent and internal molecular reorganization.^17^ Electrocatalysis is necessary to overcome kinetic barriers and selectively reduce CO_2_ to desired liquid products.^18^ Proton-coupled multi-electron transfer of ECR can produce various C_1_–C_3_ liquid products, depending on the number of protons and electrons involved (see Table S1).

#### Formation of HCOOH/HCOO^−^

**Figure 1:**
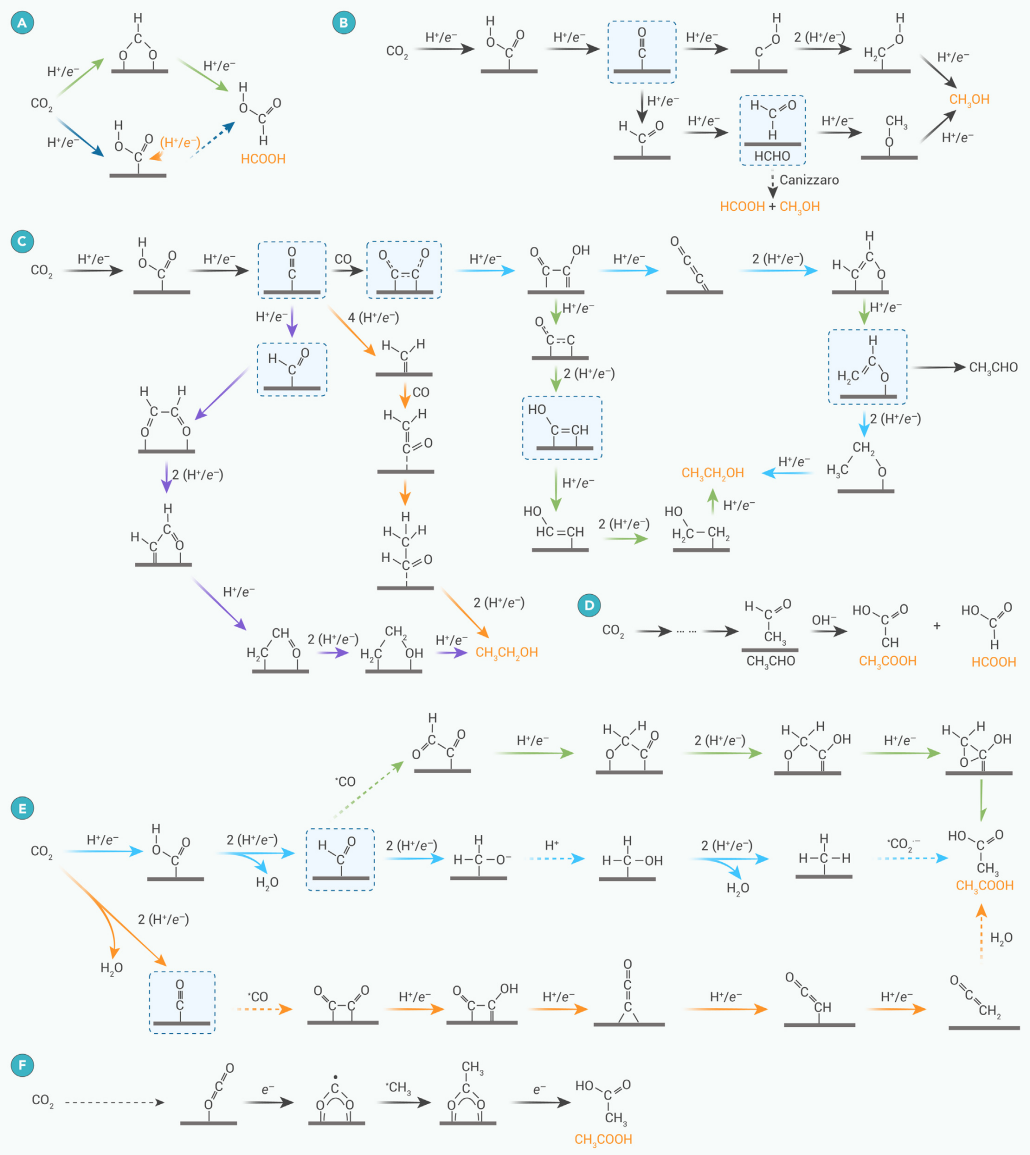
Possible pathways for generation of C_1_ and C_2_ products (A) HCOOH and (B) CH_3_OH. (C) C_2_H_5_OH and CH_3_CHO. (D–F) (D) Possible pathways for generation of CH_3_COOH via Cannizaro path, (E) C−C coupling paths, and (F) radical anion path.

HCOOH is valuable as a chemical feedstock, a hydrogen carrier, and a fuel for fuel cells. Formation of HCOOH involves the transfer of two electrons and two protons through three possible reaction pathways. In the first pathway, CO_2_ receives an electron to form ∗CO_2_^·−^, where the prefix “∗” denotes a surface-bound species that is then protonated to form an ∗OCHO intermediate (green arrows in Figure 1A). Another pathway involves transferring a proton and an electron from the solution to an adsorbed species, reducing CO_2_ to ∗COOH. A second proton then attacks the C atom in the ∗COOH intermediate, ultimately producing HCOOH.^19^^,^^20^

#### Formation of CH_3_OH

CH_3_OH can be directly used in fuel cells and as a feedstock for many commodity chemicals and fatty acid methyl esters (for biodiesel fuels, for example). There are three main reaction pathways for CH_3_OH generation. Firstly, there is OH group abstraction from the ∗COOH intermediate to form an ∗CO intermediate (Figure 1B). The ∗CO intermediate is then hydrogenated to form ∗COH or ∗CHO, which subsequently undergoes further hydrogenation to form ∗CH_2_OH and then ∗CH_3_OH, finally desorbing from the catalyst as CH_3_OH. In another pathway, ∗CHO is hydrogenated to form formaldehyde (CH_2_O) with the ∗OCH_3_ intermediate generated through a proton-electron coupled-transfer (PECT) process following CH_2_O formation. Weakly adsorbed ∗CH_2_O may undergo further electrochemical reduction steps, while CH_2_O molecules desorbed from the active site may undergo a base-induced Cannizzaro reaction near the electrode surface, producing HCOOH and CH_3_OH.^21^

#### Formation of C_2_H_5_OH and CH_3_CHO

CH_3_CHO is a critical intermediate in synthesizing pharmaceuticals, agrochemicals, and fragrances. The formation of C_2_H_5_OH requires the transfer of 12 electrons (Figure 1C). After the hydrogenation and dehydration of two ∗CO species, the formed ∗C_2_O intermediate is hydrogenated to produce ∗C_2_OH or ∗CHCO. C_2_H_5_OH is then generated through the slow hydrogenation of ∗C_2_OH. Alternatively, hydrogenating ∗CHCO yields ∗CH_*x*_CHO (*x* = 1, 2), which is hydrogenated to C_2_H_5_OH.

Some studies suggest that ∗CO dimerization should occur before proton transfer as the first step in forming C_2+_ products.^22^ Chen et al. proposed that, after C−C coupling, the oxygen atoms in ∗OCCO are hydrogenated to form ∗CO−COH.^23^ The mechanism of C−C bond formation also depends on the applied potential; at low overpotentials, the C−C bonds are formed by dimerization of ∗CO intermediates, while, at high overpotentials, they form by the reaction of ∗CO and ∗CHO (purple arrows in Figure 1C).^24^ The following ∗OCCO hydrodeoxygenation step determines the selectivity for ethylene or ethanol. A proton-electron transfer converts ∗OCCO to ∗OCCOH. The different atomic configurations of the active site induce the formation of two different adsorption structures of the ∗CCO intermediate.^25^ In one of them, the ∗CCO intermediate undergoes a further PECT step to generate the ∗HCCOH intermediate (green arrows in Figure 1C), followed by the attack of ∗H on the C atom to form the C−C−O bond, and further hydrogenation to form ∗HCCHO.^26^ In the other path, ∗H attacks the C next to the C−O bond of the ∗CCO intermediate to form the ∗CH_3_CHO intermediate (blue arrows in Figure 1C), which is further hydrogenated to form ethanol.

Recent studies have further investigated the influence of adsorbed ∗CO intermediates’ orientation on C−C formation mechanisms. Shen et al. proposed an asymmetric C−C coupling mechanism on Cu-Pd alloys,^27^ with density functional theory (DFT) calculations showing that ∗CO intermediates changed from top adsorption to bridge adsorption on the Cu-Pd surface with increasing Pd content. The ∗CO species on the bridge sites of Cu-Pd are more likely to be hydrogenated to form ∗CHO than the ∗CO species on the top sites of the Cu surface. These ∗CO species hydrogenate to ∗CHO, forming C_2_ species by C−C coupling with ∗CO at adjacent Cu sites. Furthermore, Qiao’s group verified by *in situ* attenuated total reflection infrared absorption spectroscopy (ATR-IRAS) characterization that the surface of Ag-modified Cu_2_O (dCu_2_O/Ag_2.3%_) also exhibited both top and bridge adsorption modes of ∗CO.^28^ The ∗CHO intermediates were observed on the surface of dCu_2_O/Ag_2.3%_, which further confirmed the asymmetric C−C coupling mechanism.

In addition to the adsorption of ∗CO, the role of surface adsorption of H has been investigated.^29^ ∗HCCOH acts as a key intermediate in the C_2_H_4_ vs. C_2_H_5_OH branching pathway, where the C−O bond between the hydroxyl group and ∗CCH is dissociated with the help of water molecules, and ∗CCH is further hydrogenated to form ethylene. However, the adsorbed H only participates in the C_2_H_5_OH-generation pathway, and the adsorbed H attacks ∗HCCOH to form ∗HCCHOH, the key intermediate for C_2_H_5_OH generation. Therefore, it is beneficial to increase the surface H coverage to improve the selectivity of ECR toward C_2_H_5_OH.

An asymmetric ∗CH_2_−CO coupling mechanism for C_2_H_5_OH formation uses Cu nanosheets with a Cu(111)-oriented surface (to generate ∗CH_2_ intermediate) and Ag nanoparticles (NPs) (to enhance local CO concentration) (red arrows in Figure 1C).^30^ Coupling of surface-adsorbed calculations shows that, for ∗CH_2_ and ∗CO toward C_2_H_5_OH at low applied potential, a Langmuir-Hinshelwood (L-H) mechanism occurs, while, at more negative potential, an Eley-Rideal mechanism favors C_2_H_5_OH yield. This process could be promoted by an increase in local CO concentration.

#### Formation of CH_3_COOH/CH_3_COO^−^

CH_3_COOH has applications in the food, chemical, textile, pharmaceutical, and cosmetics sectors. There are six pathways for CH_3_COOH generation by ECR. Koper et al.^31^ proposed that CH_3_COOH can be generated by the Cannizaro reaction of CH_3_CHO, facilitated by the local alkaline environment on the electrode surface caused by the HER (Figure 1D). According to theoretical calculations, the ternary ring produced by ∗OCH_2_−COH isomerization leads to acetate by-product in the ethylene (C_2_H_4_)-generation route (green arrows in Figure 1E).^32^ The ∗CO intermediate can also be hydrogenated to ∗CH_3_, which then couples with CO_2_ to form CH_3_COOH, avoiding the production of C_2_H_4_ and C_2_H_5_OH (blue arrows in Figure 1E).^33^ Another CH_3_COOH-generation mechanism was proposed by Luc et al.,^34^ involving the hydrolysis of CH_2_CO to CH_3_COOH and C_2_H_5_OH (purple arrows in Figure 1E).

The reaction path: CO_2_ → CO_2_^·−^ → (COO)_2_^·^ → CH_3_COO^−^ has been discussed for the ECR on an N-doped nanodiamond/Si rod array.^35^ The CO_2_^·−^ radical may be protonated by HCO_3_^−^ to form HCOO^−^ and HCOOH, or the CO_2_^·−^ combines with another anion radical to form the OOC−COO intermediate, which is further protonated and reduced to CH_3_COOH. Recently, Dai et al.^36^ reported a bidentate ∗OC^·^O∗ pathway at Cu(I) sites (Figure 1F) under higher [CO_2_ (aq)]/[HCO_3_^−^] conditions, where ∗OC^·^O∗ coupling with ∗CH_3_ formed at Cu(0) sites is favored over the protonation of ∗OC^·^O∗ to form formate, resulting in a higher acetic acid FE.

#### Formation of HOC_2_H_4_OH

In the formation of ethylene glycol, the ∗CO_2_^·−^ anionic radical bound on the catalyst surface generates HCOOH∗ through a one-electron hydrogen transfer step, with the OH group protonated to form ∗H_2_CO−OH_2_ and then to formaldehyde∗ (H_2_CO∗).^37^ Two adjacent H_2_CO∗ intermediates then undergo H transfer to produce HCO∗ and CH_2_OH∗. HCO∗ dissociates to produce CO∗ and H∗, where CO∗ and CH_2_OH∗ are C−C coupled. Meanwhile, the H∗ and the ketone group on the OHCH_2_−CO∗ molecule combine to form glycolaldehyde (OHCH_2_−CHO∗). Eventually OHCH_2_−CHO∗ hydrogenates to form ethylene glycol.

#### Formation of C_2_H_5_CH_2_OH and 2-propanol

**Figure 2:**
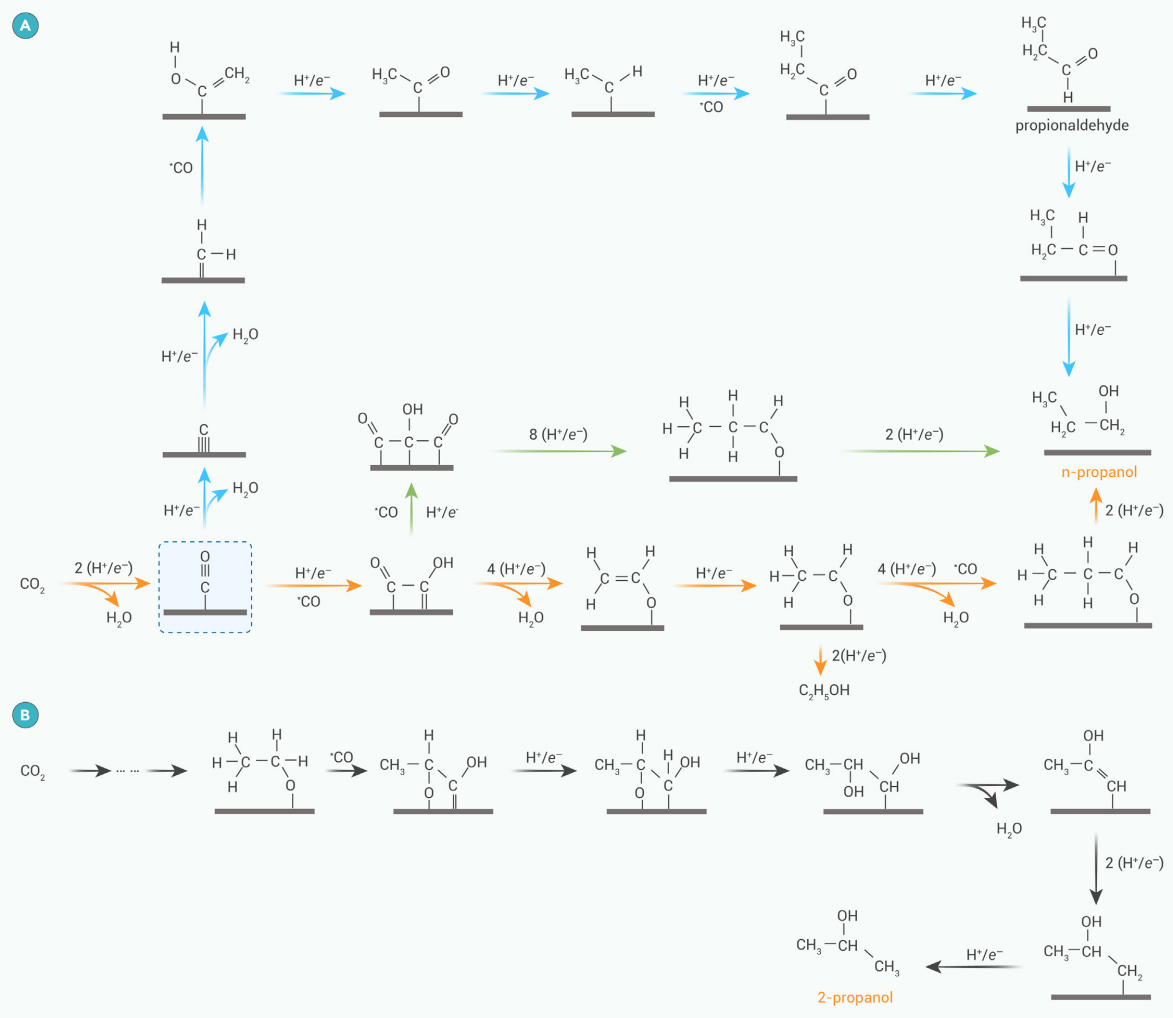
Possible pathways for generation of propanol (A) *n*-propanol and (B) 2-propanol.

C_2_H_5_CH_2_OH is a valuable C_3_ alcohol that possesses a high energy density (∼30.9 kJ g^−1^) and high octane number. Its formation on copper catalysts requires at least three Cu sites to adsorb and stabilize the critical C_2_ intermediates (∗C_2_) and ∗CO,^38^ followed by a complex coupling mechanism between the C_1_ and C_2_ species. So far, CO−CH_2_CHO^39^ and CO−OCCO^40^ have been proposed as two key intermediates. Hori et al.^41^ reported that C_2_H_5_CH_2_OH may be reduced from propionaldehyde (C_2_H_5_CHO) that is formed in the reaction, with CH_3_CHO a common intermediate for C_2_H_5_OH and C_2_H_5_CHO (blue arrows in Figure 2A). As the potential becomes more negative, the electrochemical reduction of CH_3_CHO to C_2_H_5_OH becomes unfavorable relative to C−C coupling between CH_3_CHO and CO, leading to C_2_H_5_CHO intermediate and thus promoting C_2_H_5_CH_2_OH production.

In recent years, another theory has come to the fore. CO dimers are considered to be one of the main C−C coupling pathways for ∗OCCO formation (green arrows in Figure 2A).^42^ Since ∗CO is enriched at the surface during CO_2_ reduction, coupling of CO and OCCO is a possible route.^43^ The ∗C_3_ intermediate CO−OCCO∗ is most widely used in theoretical calculations and is plausible not only for *n*-propanol but also for C_3_ products such as acetone.^44^ In addition, in the formation process of multi-C products, ∗CH_2_CHO is considered as the selective-determining intermediate.^45^ This reaction pathway is more likely to generate C_2_H_4_ than C_2_H_5_OH or C_2_H_5_CH_2_OH, because the energy barrier for generating ∗CH_3_CHO is 0.2 eV higher than that for generating C_2_H_4_. Furthermore, the formation of C_2_H_5_CH_2_OH requires ∗CH_3_CHO to undergo further C−C coupling with ∗CO (red arrows in Figure 2A). This requires a relatively high coverage of C_2_ intermediates.

Recently, Qi et al.^46^ speculated, based on DFT calculations, that 2-propanol was formed by coupling the ∗OCH_2_CH_3_ and ∗CO intermediates (Figure 2B), which was further confirmed by the addition of ethanol-d_6_ to the electrolyte in experiments. The formation of 2-propanol-d_8_ increased with the presence of ethanol-d_6_ in the electrolyte, indicating that ∗OCH_2_CH_3_ is directly involved in the formation of 2-propanol.

#### Formation of 1,2-propanediol (CH_2_OHCHOHCH_3_)

CH_2_OHCHOHCH_3_ is an important commodity chemical for fabricating pharmaceutical intermediates, degradable memory ﬁbers, and polymers.^47^ The production of CH_2_OHCHOHCH_3_ by ECR has been theoretically predicted; however, no reasonable yields have been achieved. The reaction mechanism is still elusive. Jiao et al. carried out DFT studies and suggested that the co-adsorption energy of three ∗CO intermediates (Δ*G*_3∗CO_) could be used as a descriptor for C_3_ activity. They adopted Ag-doped Cu (Ag_1_Cu) single-atom alloys (SAAs) as a model, showing they can retain oxygen atoms in the hydroxyl group and further lead to a new C_3_ product (1,2-propanediol [1,2-PDO]) rather than conventional *n*-propanol. Meanwhile, the reaction pathway for direct protonation of C to ∗COH−CO−CHOH (∗COH−CO−COH → ∗COH−CO−CHOH) is predicted to be facile because this process is exergonic (0.12 eV) and thus favorable for the subsequent steps, with the preservation of the oxygen atom in the hydroxyl group ensured by the preferential protonation of C. Hence, 1,2-PDO can be more easily formed than the other possible C_3_ products.

#### Formation of methylglyoxal and 2,3-furanediol

**Figure 3:**
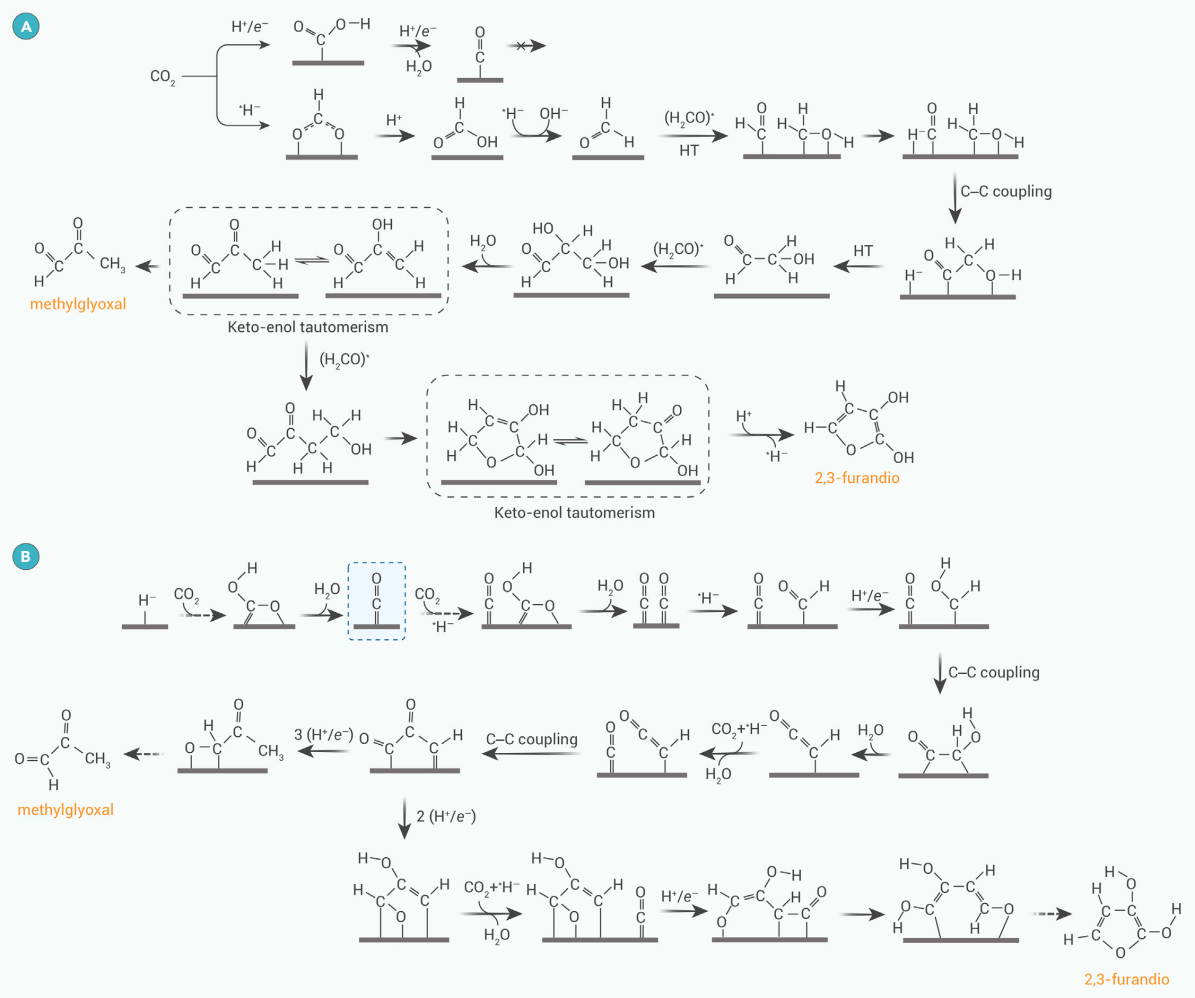
Possible pathways for generation of methylglyoxal and 2,3-furanediol (A) HT mechanism and (B) WAPT mechanism.

In the methylglyoxal and 2,3-furandiol-generation pathways (Figure 3A), CO_2_ first binds to the hydride on the catalyst surface through hydride transfer (HT) to form adsorbed HCOO^−^, which is subsequently protonated to form adsorbed HCOOH. HCOOH is then attacked by a second hydride, forming H_2_CO∗ and eliminating the hydroxide. The self-condensation of H_2_CO∗, shown by DFT calculations, involves four important steps.^48^ Hydrogens transfer between two adjacent H_2_CO∗, resulting in ∗CHO and CH_2_OH∗. Next, dissociation of ∗CHO occurs to form CO∗ and H∗. CO∗ and CH_2_OH∗ further react to form HOCH_2_−CO∗. Finally, H∗ ligates to the ketone group of HOCH_2_−CO∗, generating the C_2_ intermediate HOCH_2_−CHO∗, which then undergoes C−C coupling with H_2_CO∗ to produce glyceraldehyde∗. The OH group at the end of glyceraldehyde∗ is more likely to undergo water elimination to produce 2-hydroxy-2-propenal.^37^ Methylglyoxal can be produced by enol-keto tautomerization.^49^ Otherwise, 2-hydroxy-2-propenal can be further C−C coupled with H_2_CO∗ to produce dihydro-2-hydroxy-furanone and finally aromatization into 2,3-furanediol.

Zheng et al.^50^ also proposed a water-assisted proton transfer (WAPT) mechanism on Ni_3_P_2_ (Figure 3B). CO_2_ is hydrogenated through direct proton transfer at the Ni_3_ site to form ∗OCHO or ∗COOH, with ∗COOH formation favored in the presence of hydrogen-bond interactions. The WAPT process involves the separation and transfer of a surface proton to a nearby water molecule, the hopping of another proton from this hydrohydrate to a neighboring water molecule, and finally the hydrogenation of CO_2_ by a proton from the hydrogen-bonding network. After two WAPT processes, the second CO_2_ was hydrogenated to ∗CHOH and subjected to C−C coupling with ∗CO. Following the dehydration of ∗CO−CHOH to create ∗CO−CH, ∗CO was then C−C combined with ∗CO−CHOH to create ∗CO−CO−CH. It further undergoes a successive hydrogenation step to generate ∗CHO−CO−CH_3_, which is desorbed as methylglyoxal. Alternatively, the C_3_ intermediate ∗CO−CO−CH can be hydrogenated to ∗CHO−COH−CH and undergo C−C coupling with a fourth ∗CO to produce the C_4_ species ∗CHO−COH−CH−CO. This is subsequently transformed to 2,3-furandiol via proton tautomerization, hydrogenation, and cyclization. Although this pathway provides an explanation for the product distribution observed on Ni_3_P_2_, experimental verification of this theoretical finding is needed.

Understanding the elementary steps controlling C−C coupling and product bifurcation is crucial for targeting specific high-value products. Further exploration of reaction mechanisms after the first C−C dimerization step is needed.

#### Formation of 3-hydroxybutanal, *n*-butanol, and *t*-butanol

**Figure 4:**
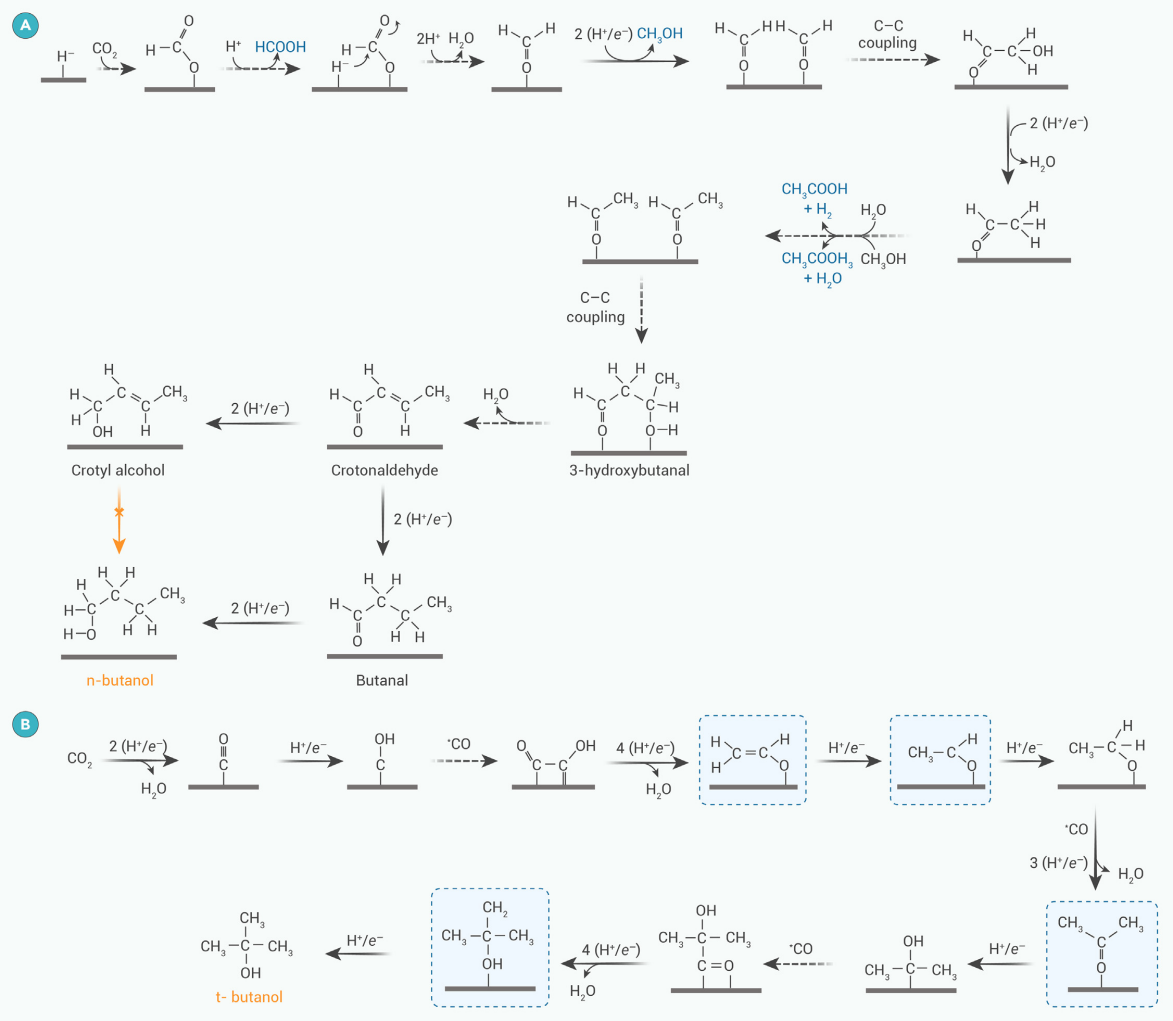
Possible pathways for generation of butanol (A) *n*-butanol and (B) *t*-butanol.

According to Cronin et al.,^51^ 3-hydroxybutanal is an intermediate in *n*-butanol formation. HCOOH is considered an important two-electron unit in the pathway from which the rest of the intermediates are generated. As with the ethylene glycol-generation pathway, CO_2_ is first electrolyzed to HCOOH, which is further converted to HCHO. Two molecules of HCHO then undergo self-condensation to form CH_3_CHO, which then condenses to form 3-hydroxybutanal.^51^ This intermediate is hydrogenated to *n*-butanol on the catalyst surface (Figure 4A). 3-Hydroxybutanal loses an *α*-hydrogen to form a carbene, leading to crotonaldehyde formation. Theoretical analysis of crotonaldehyde reduction suggests that butanal is formed by sequential hydrogenation of the *β*- and *α*-C of crotonaldehyde. At a reduction potential greater than −1.02 V (vs. standard hydrogen electrode [SHE]), butanal receives an electron from the cathode surface, forming a CH_3_CH_2_CH_2_C∗HO^−^ anion, and ultimately produces butanol.^52^

Unlike *n*-butanol generation, *t*-butanol generation involves a C_3_ intermediate and a ∗CO−∗COH coupling pathway.^53^ The ∗CO intermediate couples to the adjacent C_1_ intermediate ∗COH to form ∗COCOH, which dehydrates to CH_2_CHO∗ and is further hydrogenated to CH_3_CHO∗. This intermediate is then converted to (CH_3_)_3_CO∗ by an additional PECT process and CO insertion. Alternatively, through PCET, (CH_3_)_2_CO∗ can be converted to (CH_3_)_3_COH (Figure 4B), with key intermediates CH_2_CHO∗, CH_3_CHO∗, CH_3_CH_2_O∗, (CH_3_)_2_COH(CO)∗, and (CH_3_)_2_CH_2_C∗OH. Although this pathway has been supported by DFT calculations, there is no experimental evidence via *in situ* detection to confirm these intermediates.

### Microkinetic modeling and simulation

The numerous pathways for ECR, as elucidated in possible reaction pathways of ECR toward liquid products section, highlight the potential for producing valuable chemical feedstocks but simultaneously presents a selectivity challenge. Designing catalytic systems that selectively activate one specific product pathway requires an in-depth understanding of dominant pathway(s) and key steps under specific conditions. While DFT calculations offer insight into ECR thermodynamics and kinetics,^54^ they do not fully capture operating conditions like reactant concentration, pH, and applied potential.^55^^,^^56^ Microkinetic modeling, integrated with DFT calculations, addresses these gaps, offering a practical alternative to expensive *operando* techniques.

Microkinetic simulation modeling extrapolates kinetic knowledge from the microscopic to the macroscopic scale, estimating reaction rates under specific conditions. It begins with a detailed reaction scheme encompassing all reactants, products, and intermediates. DFT calculations then provide kinetic and thermodynamic parameters, such as adsorption energy and activation energy. These parameters are integrated into material balance equations and rate equations to predict reaction rates of the elementary steps. However, the variety of chemical species in ECR necessitates intensive computation, making microkinetic modeling less viable. The Brønsted-Evans-Polanyi (BEP) relation and linear scaling relation can be used to simplify activation energy estimation and reaction energy prediction.^57^ A linear scaling relationship, detailed elsewhere in this article, enables the estimation of reaction energies from the adsorption energy of a key intermediate. Machine learning (ML) has been utilized to diminish the number of computations needed for such modeling.^58^^,^^59^ See Motagamwala’s review^60^ for more details about microkinetic modeling formalism.

Microkinetic modeling and simulation can also be used to describe reaction mechanisms under operational conditions, identifying dominant pathways and rate-determining steps,^60^^,^^61^ as shown for the HER, oxygen evolution reaction (OER), and oxygen reduction reaction (ORR).^62^^,^^63^^,^^64^ However, the complexity of ECR network, particularly for highly reduced products, poses significant challenges.^60^ Often, only critical segments of the entire ECR network (e.g., CO → ∗OCCHO)^65^ are modeled, or just basic reactions involving two PCETs (CO_2_ → CO/HCOOH).^66^ Nevertheless, due to its significance, mechanistic studies leveraging microkinetic modeling for complex ECR kinetics have been made.^61^

Liu et al. investigated the impact of applied potential and pH on ECR kinetics on Cu(211) surfaces using a microkinetic model with about 40 elementary steps, correlating their results with experimental data.^56^ They identified a pathway via ∗OCCOH as the dominant C_2_ pathway, with the rate-limiting step varied with applied potential. Under alkaline conditions, ∗CO dimerization is the limiting step at high overpotentials (−1.0 V vs. reversible hydrogen electrode [RHE]), explaining the faster decrease in C_2_ production rate with increasing overpotential. Increasing the pH from 7 to 13 improved ECR kinetics by lowering the activation barrier for hydrogenation steps, enhancing C_2_ selectivity over C_1_ formation.

Recently, Kraft and coworkers modeled a comprehensive network of possible ECR pathways for nine products, including liquid fuels (HCOOH, CH_3_OH, CH_3_CH_2_OH), consisting of 88 elementary steps.^67^ They used Huang et al.’s^68^ experimental benchmark to construct microkinetic simulation models at different applied potentials (*U*_RHE_ = −1.15, −1.0, and −0.85 V) under neutral conditions (pH = 6.8) on a Cu(100) surface. The model revealed that ECR mechanisms toward C_2_ products are inactive except for C_2_H_4_. The dominant reaction pathway for generating HCOOH at low potential (*U*_RHE_ = −0.85 V) included CO_2_ physisorption followed by one PECT (CO_2_ → ∗CO_2_ → ∗OCHO) step. As the applied potential becomes more negative, the dominant reaction pathway changed to include a direct (CO_2_ → ∗OCHO) route, accompanied by a diminished reaction rate.

### Oxidation half-reaction

The OER is the typical counter reaction at the anode in a CO_2_ electrolyzer, but it requires a potential higher than the equilibrium potential of 1.23 V (vs. RHE), lowering the overall energy efficiency. This has been estimated to be no higher than 50% at a current density of 100 mA cm^−2^, with the OER being responsible for ∼64% of the cell voltage.^69^ Alternative anodic reactions that exhibit faster kinetics, higher durability, and more valuable products (than O_2_) are desirable. Oxidation of glycerol to HCOO^−^ and lactate, or the oxidation of glucose to gluconic acid, is preferred due to their lower equilibrium potential and yielding more valuable products.^70^ Table 1^71^ summarizes potential anodic oxidation reactions that can be used as alternatives to the OER reaction, some of which have already been coupled with ECR. Na et al.^72^ conducted a technoeconomic analysis of 295 cases of co-electrolysis that couple ECR with organic oxidation. They found that the oxidation reaction processes producing carboxylic acids (e.g., formic acid^25^ and 2,5-furandicarboxylic acid^73^) are the best candidates for electrochemical co-production. However, practical considerations, such as the separation of anode products from the anolyte, need to be addressed. Beyond economic benefits, the availability of organic substrates is also a key factor in achieving large-scale co-electrolysis. For example, controlling global CO_2_ emissions is incompatible with the production of glycerol, creating a significant supply gap for glycerol.^70^ As a result, more readily available substrates like CH_4_^74^ and C_2_H_4_^75^ are preferred.

**Table 1:** Possible alternative oxidation reactions in alkali media and their respective equilibrium potentials (*E*^0^ vs. RHE)

Possible anode reaction	Reaction equation in alkali media	*E*^0^/V
H_2_O → H_2_O_2_	2OH^−^ → H_2_O_2_ + 2*e*^−^	1.78
Cl^−^ → Cl_2_	2Cl^−^ → Cl_2_ + 2*e*^−^	1.36
H_2_O → O_2_	4OH^−^ → O_2_ + 2H_2_O + 4*e*^−^	1.23
Cl^−^ → ClO^−^	Cl^−^ + 2OH^−^ → ClO^−^ +H_2_O + 2*e*^−^	0.89
S^2−^ → S_x_^2−^	2HS^−^ + 2OH^−^ → S_2_^2−^ + 2H_2_O + 2*e*^−^	0.14
Urea → N_2_ + CO_2_	CO(NH_2_)_2_ + 7OH^−^ → N_2_ + HCO_3_^−^ + 5H_2_O + 6*e*^−^	0.07
NH_3_ → N_2_ + H_2_O	2NH_3_ + 6OH^−^ → N_2_ + 6H_2_O + 6*e*^−^	0.06
N_2_H_4_ → N_2_ + H_2_O	N_2_H_4_ + 4OH^−^ → N_2_ + 4H_2_O + 4*e*^−^	−0.33
Glucose → Gluconic acid	C_6_H_12_O_6_ + 2OH^−^ → C_6_H_12_O_7_ + H_2_O + 2*e*^−^	0.90
Glycerol → Lactic acid	C_3_H_8_O_3_ + 3OH^−^ → C_3_H_5_O_3_^−^ + 3H_2_O + 2*e*^−^	0.25
Glycerol → HCOO^−^	C_3_H_8_O_3_ + 11OH^−^ → 3HCOO^−^ + 8H_2_O + 8*e*^−^	0.14
1,2-Propanediol → Lactic acid	C_3_H_8_O_2_ + 5OH^−^ → C_3_H_5_O_3_^−^ + 4H_2_O + 2*e*^−^	–
Ethanol → Acetate	C_2_H_5_OH + 3OH^−^ → CH_3_COO^−^ + 3H_2_O + 2*e*^−^	0.06
CH_3_OH → HCOO^−^	CH_3_OH + 3OH^−^ → HCOO^−^ + 3H_2_O + 2*e*^−^	0.11
R−CHO → R−COO^−^	R−CHO + 3OH^−^ → R−COO^−^ + 2H_2_O + 2*e*^−^	–
5-Hydroxymethylfurfural (HMF) →2,5-Furandicarboxylicacid (FDCA)	C_6_H_6_O_3_ + 6OH^−^ → C_6_H_2_O_5_^2−^ + 4H_2_O + 4*e*^−^	0.30
Furfural → Furoic acid	C_5_H_4_O_2_ + 3OH^−^ → C_5_H_3_O_3_^−^ + 2H_2_O + 2*e*^−^	–
R−CHO → R−COO^−^ + H_2_	2R−CHO + 4OH^−^ → 2R−COO^−^ + H_2_ + 2H_2_O + 2*e*^−^	–
HMF → 5-Hydroxymethyl-2-furancarboxylic acid (HMFCA) + H_2_	2C_6_H_6_O_3_ + 4OH^−^ → 2C_6_H_3_O_4_^−^ + H_2_ + 2H_2_O + 2*e*^−^	–
Furfural → Furoic acid + H_2_	2C_5_H_4_O_2_ + 4OH^−^ → 2C_5_H_3_O_3_^−^ + H_2_ + 2H_2_O + 2*e*^−^	–
R−CH_2_−NH_2_ → R−CN	R−CH_2_−NH_2_ + 4OH^−^ → R−CN + 4H_2_O + 4*e*^−^	–
Octylamine → Octanenitrile	C_8_H_19_N + 4OH^−^ → C_8_H_15_N + 4H_2_O + 4*e*^−^	–
C_2_H_4_ → Ethylene oxide	C_2_H_4_ + 2OH^−^ → C_2_H_4_O + H_2_O + 2*e*^−^ (mediated by Cl^−^)	1.36
CH_4_ → CH_3_OH	CH_4_ + 2OH^−^ → CH_3_OH + H_2_O + 2*e*^−^	0.58

### Protocols for CO_2_ electrolysis

For meaningful and comparable ECR results, and to ensure consistency and reproducibility, experimental conditions such as electrolyte properties, cell configuration, CO_2_ flow rate, and catalyst ink formulation need to be identical. Chemicals and commercial catalysts or raw materials used should come from the same vendors or batches to avoid uncontrolled performance variations.^76^ It is important to measure intrinsic catalytic activity under conditions that minimize mass transfer effects, as ECR is susceptible to concentration polarization. A recent study highlighted this polarization by controlling the catalyst’s microenvironment, where Nafion (Naf.) was employed for stabilization of the catalyst morphology during ECR. High water/alcohol ratios and low Naf. fractions in the catalyst ink created stable microenvironments, increasing the local CO_2_/H_2_O concentration ratio and facilitating high CO surface coverage for efficient CO_2_ electrolysis.^77^

#### Impurity effects

Trace levels of contaminants in electrolytes, raw materials, and the reaction vessel can alter the electrode-electrolyte interface by forming new active sites or blocking catalytic sites, thus influencing the ECR activity and selectivity.^78^ Metallic impurities arising from the electrode materials or electrolyte salts can be reduced and deposited on the cathode during electrolysis, intensifying the parasitic HER.^79^ To remove metal impurities, a pre-cathodic polarization step or periodic CV treatment with chelating agents like ethylenediaminetetraacetic acid (EDTA) is recommended.^80^ Raw CO_2_ feed gas may contain impurities, such as CO, O_2_, SO_x_, NO_x_, and volatile organic compounds. Low-concentration SO_2_ can hydrogenate with ∗H on the catalyst surface, with the accumulated ∗H and ∗S species poisoning the catalyst. This also transforms the CO_2_ hydrogenation from an Eley-Rideal mechanism, with protons from H_2_O, to a Langmuir-Hinshelwood mechanism, with ∗H, thus suppressing ∗CO formation and C_2+_ production. In addition, ∗S coverage impedes ∗COOH adsorption, and the reduction of SO_2_ to form ∗SOOH and ∗HSO_2_ species is preferred over ECR. An SO_2_-tolerant system using a perfluorosulfonic acid (PFSA)@Cu/polytetrafluoroethylene (PTFE) heterostructure was designed to decrease hydrogen adsorption and also facilitate CO_2_ migration over SO_2_ molecules.^81^ A C_2+_ FE as high as 84% with an absolute partial current density of 790 mA cm^−2^ was attained in CO_2_ containing 400 ppm SO_2_.

Enhanced C_2+_ formation via co-electrocatalysis of CO_2_ and O_2_ to stabilize the surface hydroxyl group of Cu catalysts was reported.^82^ Adding 20% O_2_ to CO_2_ feedstock resulted in a 170-fold, 55-fold, and 35-fold increase in the yield rate of C_2_H_4_, C_2_H_5_OH, and CH_3_COO^−^, respectively, at −0.75 V (vs. RHE) in 0.1 M KHCO_3_. The yield of C_2_H_5_CH_2_OH was also observed at this potential in the presence of O_2_, whereas no C_2_H_5_CH_2_OH was detectable in an oxygen-free feed gas. However, the presence of O_2_ as an impurity in CO_2_ led to loss of 99% of the applied current due to the occurrence of the more thermodynamically preferred ORR.^83^ Operating in an acidic electrolyte can inhibit ORR on Cu, enabling oxygen-tolerant C_2+_ yield.^83^

A small amount of CO and O_2_ can promote the ECR by lowering the reduction potential and altering the catalyst’s oxidation state.^84^ DFT studies suggest that O_2_ in the CO_2_ gas feed is rapidly reduced to ∗OH,^85^ which can draw K^+^ to the copper surface through electrostatic interactions, increasing the concentration of K^+^ at the interface and lowering the kinetic barrier for C−C coupling. Other contaminants may also inhibit the adsorption of CO_2_ and intermediates or poison the catalyst surface and are thus detrimental to the ECR. Metal may leach from the counter electrode or from a metallic anode, which can be subsequently deposited on the cathode surface, having a significant effect on the ECR.

#### Standards for data acquisition and reporting

Combined metrics, including faradaic efficiency, energy efficiency, partial current density, and product-generation rate (proportional to its partial current density), should be used to report electrocatalytic activity and selectivity. For meaningful comparisons of intrinsic activity among different catalysts, measured rates and current densities must be normalized by the number of available catalytic sites and the electrochemically active surface area (ECSA), respectively, to rule out surface area effects.

Accurate assessment of high-rate CO_2_ electrolysis in flow cells should consider the outlet flow rate, gas products removed by the flowing catholyte, crossover of liquid products (e.g., HCOO^−^, CH_3_COO^−^, and C_2_H_5_OH) from the cathode to the anode, and evaporation of the liquid products (e.g., CH_3_CHO, C_2_H_5_OH, and C_2_H_5_CH_2_OH) from gas-diffusion electrodes (GDEs).^86^ CO_2_ utilization efficiency can be investigated using the ratio of the CO_2_ reduction rate at the cathode to the sum of the CO_2_ reduction rate and the HCO_3_^−^ and CO_3_^2−^ fluxes through the cell. A decrease in membrane thickness (reaction length) accelerates the transport of H^+^ (with more rapid mobility than either HCO_3_^−^ or CO_3_^2−^) from the anode to the cathode and neutralizing the CO_3_^2−^.^87^ Additionally, the HCO_3_^−^ diffusion gradient toward the cathode enables a higher CO_3_^2−^ flux toward the cathode, increasing utilization. Alternatively, lowering the CO_2_ partial pressure also improves CO_2_ utilization by alleviating CO_3_^2−^ formation and favors OH^−^ flux over CO_3_^2−^ flux.

The FEs for gas products in flow cells can be overestimated if CO_2_ consumption is not considered, especially at high current densities and low CO_2_ inlet flow rates. This is due to the formation of CO_3_^2−^ stemming from the reaction of CO_2_ with the OH^−^ at the cathode, which migrates to the anode, converts back to CO_2_, and leaves the cell through the anolyte. Bubble accumulation at the electrode-electrolyte interface and evaporation of liquids along with the outlet gas are accelerated under larger current densities and faster catholyte flow rates, while the issue of crossover becomes more severe at lower volumes of catholyte and reduced catholyte flow rates. To mitigate these problems, the outlet gas can be scrubbed by the catholyte, which is then mixed with potential gas products from the catholyte for online gas chromatography (GC) quantification through a mass flow meter. The application of a bipolar membrane (BPM) instead of an anion-exchange membrane (AEM) can hinder electromigration and electroosmotic drag and alleviate crossover. Note that C balance analyses should be conducted for CO_2_ electrolysis operated at large cathodic currents to ensure that CO_2_ consumption is fully accounted for.

Overall, unified protocols are required for reporting, measuring, and analyzing all of the above metrics for an objective evaluation of ECR catalysts.

#### Temperature and pressure effects

Increasing the operating temperature to within certain ranges can improve reaction rates and energy efficiency (i.e., the applied bias divided by the thermodynamic voltage); however, a mechanistic explanation for temperature effects on the ECR is still lacking. Temperature can affect the local pH, CO_2_ solubility, reactant diffusion rates, intermediate adsorption, and electrolyte resistance.^88^ Higher temperatures result in lower CO_2_ solubility due to Henry’s law and the acid/base buffer equilibria (CO_2_/HCO_3_^−^/CO_3_^2−^) and also favor the parasitic HER. As a result, the enhancement in ECR kinetics may be counteracted by a decrease in CO_2_ solubility. An optimal working temperature needs to be ascertained that balances these factors. 50 °C was reported to be preferred for HCOO^−^ formation on a SnO_2_-based GDE with an HCOO^−^ FE of over 80% at a current density of −1.0 A cm^−2^.^89^ Above or below 50 °C, more severe HER occurred. The optimal reaction temperature varies for different catalysts, and the highest temperature that both the GDE and membrane can sustain should also be acknowledged.

Elevating the CO_2_ pressure in liquid electrolytes augments its solubility from 0.03 M under ambient pressure to 1.16 M at 50 bar.^90^ This maintains the bulk catholyte pH at ∼6.2, which otherwise increases gradually due to the stoichiometric OH^−^ production. Enhanced HCOO^−^ selectivity was observed under pressure in the 1−50 bar range on a variety of catalysts, which was attributed to the higher CO_2_ coverage and lower proton concentration on the cathode surface under elevated pressure, favoring HCOO^−^ formation.^91^

#### Tafel slope of CO_2_ reduction

The kinetics, mechanism, and selectivity of ECR are largely controlled by the applied potential. The dependence of the measured current or product formation rate on the applied potential provides insights into both the reaction pathway and underlying mechanism of CO_2_ reduction. The reaction pathway and mechanism of simple electrocatalytic reactions involving the transfer of a single electron or two electrons can be determined experimentally through Tafel slope analysis, complemented by observation of reaction intermediates and products using *in situ* analytical techniques and through kinetic modeling and simulation. However, experimental determination of the Tafel slope for products involving the transfer of more than two electrons is prone to error. Extrinsic factors, such as electrolyte pH and particle size and morphology, are also known to influence the selectivity and thus mechanism of ECR, further complicating the experimental deduction of ECR reaction mechanisms using Tafel slopes from current-potential dependencies. Tafel slopes for the two-electron reduction of CO_2_ to CO or HCOOH can be determined with better reliability but only in the case of 100% selectivity and absence of concurrent background reactions and mass transport limitations. In effect, the associated kinetic parameters such as electron-transfer coefficient and exchange current density for ECR cannot be reliably determined experimentally.

The Tafel slope for ECR on Ag and Au, which are renowned for reducing CO_2_ predominantly to CO, is invariably very close to 59 mV dec^−1^, which precludes CO_2_ + *e*^−^ → CO_2_^−^ as the initial electron-transfer step.^92^ ECR on Au(111) yields almost exclusively CO in ionic liquid electrolytes.^93^ Owing to the aforementioned factors, it is challenging to experimentally determine ECR pathways and mechanisms for C_2+_ products that involve more than four PECT steps and multi-C coupling steps just from current-potential relationships. Online spectroscopic techniques coupled to electrochemical cells, and downstream product analysis, are thus frequently employed for reliably deducing ECR reaction pathways and mechanisms.^94^

## Heterogeneous catalysis of ECR to liquid products

### Selectivity of HCOOH/HCOO^−^ formation

In pioneering work conducted by Hori and coworkers,^95^ metallic catalysts were divided into four categories according to their primary ECR products, among which the *p*-block metals In,^96^ Sn,^97^ Pb,^98^ Bi,^99^ and Cd^80^ are conducive for HCOOH generation. The benchmark FE for HCOOH formation is nearly 100% in H-type electrolytic cells with advanced catalysts, while the maximum HCOOH FE in a flow cell is 93% (Table S2). The highest reported absolute cathodic partial current density toward HCOOH is −2.0 A cm^−2^ at −0.95 V (vs. RHE) on a Bi_2_S_3_-derived catalyst in a flow cell, with stable performance for 100 h.^100^ The actual active sites for CO_2_ reduction on Bi-catalyzed ECR are debated, with metallic Bi and Bi^3+^ both suggested.^101^ Bismuth oxide catalyst for ECR in a CO_2_ electrolytic cell to produce ∼5 M HCOOH gave an average FE of 80% in the first 100 h but decreased to about 75% after 500 h and 65% ∼to 70% after 1,000 h.^102^

Fang and coworkers^103^ used a Pb catalyst from waste lead-acid batteries to convert CO_2_ into HCOOH in a proton exchange membrane (PEM) system, achieving over 93% FE in an electrolyte with pH 1.0 under a full-cell voltage of 2.4 V. They showed stable performance for 5200 h at 2.2 V and −600 mA cm^−2^. This PEM system with cathodic ECR and anodic hydrogen oxidation reaction (HOR) offers a new concept for CO_2_ catalytic conversion.

### Selectivity of CH_3_OH formation

To date, the highest FE for ECR to CH_3_OH has been reported on a Co(CO_3_)_0.5_(OH)·0.11H_2_O catalyst, reaching 97% at −0.98 V (vs. saturated calomel electrode [SCE]) in a 0.5 M NaHCO_3_ electrolyte.^104^ However, the CH_3_OH partial current density was only −0.59 mA cm^−2^. For most catalysts in aqueous electrolytes, the current density is often below 50 mA cm^−2^ when the CH_3_OH FE exceeds 50% (Table S3).

Cu-based catalysts show excellent potential for ECR into CH_3_OH. Cuprous cyanamide (Cu_2_NCN) achieved an FE of 70% for CH_3_OH production, with a partial current density of −92.3 mA cm^−2^.^105^ Using 0.5 M KHCO_3_ as the electrolyte, the production rate of CH_3_OH in a membrane electrode assembly (MEA)-based electrolyzer reached 0.16 μmol s^−1^ cm^−2^. Cu_2_NCN significantly reduces the Cu−O interaction of the adsorbed ∗OCH_3_ intermediate, making it weaker than the O−C interaction at the critical reaction bifurcation point of Cu−∗O−CH_3_, directing the reaction pathway toward the release of ∗OCH_3_ and the formation of CH_3_OH.

Mahrim and coworkers discussed using ionic liquids to form catalytic Cu NPs for reducing CO_2_ to HCOOH.^106^ Recently Noh et al.^107^ showed that imidazolium cations (from ionic liquids) can promote ∗CO formation during ECR by lowering the activation energy to about zero through lower entropy ordering of imidazolium cations at the electrode surface. The negative view^108^ of ionic liquids as potential electrolytes for ECR is disappearing as their costs decrease.

In addition to metal-based catalysts, metal-free catalysts for efficient CH_3_OH generation remains challenging. B-doped diamond^109^ and B, N co-doped nanodiamonds^110^ have been used to convert CO_2_ into CH_3_OH, but the FE is below 30%. The best performance for a metal-free catalyst for CH_3_OH formation was observed on boron phosphide, which afforded a 92% FE at −0.5 V (vs. RHE) in a 0.1 M KHCO_3_ electrolyte,^111^ although it was attained at a low current density.

### Selectivity of C_2_H_5_OH formation

A low onset potential of −0.4 V (vs. RHE) for CO_2_ conversion to C_2_H_5_OH was attained with Cu_*x*_ clusters (*x* = 3 and 4) stabilized by hydroxyl groups and water on a C support. This catalyst delivered a remarkable C_2_H_5_OH FE of 91% at −0.6/−0.7 V (vs. RHE).^112^ However, the obtained maximum C_2_H_5_OH partial current density (1.12 mA cm^−2^) is too low for industrial applications and declined when the Cu loading was increased to 1.6%. Kinetic analysis is required to elucidate whether the reaction is via either an HCOO∗ and/or a CO∗ mechanism. Cu-based catalysts for C_2_H_5_OH FEs are often lower than 70% when the absolute partial current density is greater than 200 mA cm^−2^ (Table S4). Recently, Hu et al.^113^ constructed oxygen-bridged binuclear Cu sites on mesoporous N-doped C nanocages, with an FE for C_2_H_5_OH of 56.3% at a potential of −0.3 V (vs. RHE), which represents the lowest overpotential reported to date.

Beyond Cu, transition metal phosphides such as Ni_2_P, FeP, and MoP also enable C_2_H_5_OH formation.^114^ In particular, a MoP electrocatalyst coated with an imidazolium-functionalized ionomer provides a C_2_H_5_OH FE of 77.4% at a potential of only −200 mV (vs. RHE).^114^ CoO NPs immobilized on N-doped mesoporous C and C nanotubes were shown to yield C_2_H_5_OH with an FE of 60.1% at −0.32 V (vs. RHE) and a partial current density of −5.1 mA cm^−2^.^115^ Ag particles anchored on graphene-wrapped N-doped C foams were reported to give a higher C_2_H_5_OH FE of 82.1–85.2% at −0.6 to −0.7 V (vs. RHE).^116^ The good activity was attributed to pyridinic N species in the catalyst enhancing the binding of ∗CO intermediates, while the Ag particles promote the conversion of ∗CO to ∗OC−COH and then to C_2_H_5_OH.

A 93.2% FE for C_2_H_5_OH was observed on B, N co-doped nanodiamond at −1.0 V (vs. RHE).^110^ The doped B atom improves CO_2_ capture by connecting with one O atom of ∗CO_2_, while the doped N facilitates ∗H transfer during hydrogenation. B atoms play an important role by forming B−O bonds in the steps required for C_2_H_5_OH formation.

### Selectivity of CH_3_COOH/CH_3_COO^−^ formation

Only a few catalysts selectively produce CH_3_COOH/CH_3_COO^−^. Polymeric Cu-ligand complex core-shell microspheres can catalyze the ECR to CH_3_COO^−^ with an FE of ∼64% in a 0.5 M KHCO_3_ electrolyte at −0.37 V (vs. RHE).^117^ A higher CH_3_COO^−^ FE of 90.3% at a more negative potential (−0.8 V vs. RHE) was achieved by using a conductive 2D copper phthalocyanine-based covalent organic framework (COF) electrocatalyst in 0.1 M KHCO_3_,^33^ with stable performance over 80 h.

Reduction of CO_2_ to CH_3_COOH at −0.1 V (vs. RHE) for up to 100 h was obtained on bismuth-based transition metal chalcogenides, such as AgBiS_2_, CuBiS_2_, and AgBiSe_2_.^118^ Lone-pair electrons in Bi^3+^ expedites adsorption and activation of CO_2_ by assisting electron transfer, while nucleophilic chalcogens boost water activation and trap CO_2_, forming C-bound CO_2_^·−^ species. The shorter X−Bi (X = S/Se)-bond distance of AgBiSe_2_ and CuBiS_2_ favors closer proximity of adsorbed C atoms relative to AgBiS_2_, which enables C−C coupling to generate CH_3_COOH at lower potentials. The facile charge transfer between Bi and Se in AgBiSe_2_ accelerates adsorption of the ∗COOH species, leading to a higher CH_3_COOH FE on AgBiSe_2_ compared to CuBiS_2_.

A tandem catalytic system consisting of a Ni-based COF and a Cu-(3,5-diethyl-1,2,4-triazole) metal-organic framework was designed to convert CO_2_ to CO and subsequently CO to CH_3_COO^−^ via a ∗CCO intermediate, respectively.^119^ A CH_3_COO^−^ FE of 51.2% with a current density of −410 mA cm^−2^ was attained using an MEA electrolyzer with a solid-state electrolyte (SSE), yielding up to 2.72 mmol m^−2^ s^−1^ CH_3_COO^−^ at a cell voltage of 3.1 V. Competition between the CO donor and CO consumer was proposed, favoring ECR to produce CO over CH_3_COO^−^.

Aside from catalyst design, selective ECR of CH_3_COOH can also be achieved by adjusting the type and properties of the electrolyte. Using the Keggin-type polyoxometalates (POM) [SiW_9_V_3_O_40_]^−^ (SiW_9_V_3_) as a catholyte,^120^ the CH_3_COO^−^ FE on In reached 96.5% at −0.7 V (vs. RHE). The impressive CH_3_COO^−^ FE was linked to the V-center valence transition of SiW_9_V_3_ during electrolysis of the CO_2_, where the electron-transfer process in SiW_9_V_3_ participates in the ECR, leading to reduced overpotential of the reaction.

### Selectivity of CH_3_CHO formation

The production of CH_3_CHO during ECR is seldom reported. By using synergistic catalysis of Au (for CO_2_ to ∗CO), Cu (for ∗CO dimerization), and 4,4′-bipyridine (for CO_2_∗ stabilization and protonation), an FE of ∼25% for CO_2_ to CH_3_CHO conversion was achieved at −0.9 V (vs. RHE).^121^

A high CH_3_CHO FE of up to 60% with a cathodic current density of −5.1 mA cm^−2^ at −0.4 V (vs. RHE) was obtained using hexagonal-close-packed Co nanosheets in a 0.5 M KHCO_3_ electrolyte.^122^ Interlayer electron transfer in the Co nanosheets promoted ∗[OC−CO] coupling and inhibited the complete hydrogenation of the intermediates to C_2_H_4_, thereby resulting in a high CH_3_CHO selectivity.

Ag adatoms on Cu were shown to weaken the binding strength of CH_3_CHO intermediates and hinder its further conversion to C_2_H_5_OH, thus enabling the reduction of CO to CH_3_CHO with ∼70% FE and over 90% selectivity on a C basis at −0.536 V (vs. RHE).^123^ Inspired by this finding, a route to improve CH_3_CHO yield would be the design of a cascade system combining the CO_2_-to-CO step and CO-to-CH_3_CHO step.

### Selectivity of (CH_2_OH)_2_ formation

The electrochemical reduction of CO_2_ to diols is challenging due to the need to retain both oxygen atoms. The unique spatial-confinement provided by densely arrayed Cu nanopyramids (Cu-DAN) has been predicted to facilitate C−C coupling in the ECR to generate (CH_2_OH)_2_. The structure is supposed to preserve the oxygen atoms and favor the ∗COH−CHO pathway toward (CH_2_OH)_2_ and C−O bond cleavage in ∗CH_2_OH−CH_2_O intermediates is also inhibited.

CO_2_ can be reduced to (CH_2_OH)_2_ at low overpotentials on Fe_2_P, reaching 10% FE at 0 V (vs. RHE) and ∼25% at −0.05 V in 0.5 M K_2_SO_4_.^37^ The strong Fe−P bond and weak hydride adsorption energy on Fe_2_P promote the glyoxal intermediate to be rapidly hydrogenated, suppressing the formation of long-chain C_3_ and C_4_ products. HCOO∗, rather than ∗CO, is first generated from surface phosphino-hydrides, which is then converted to ∗H_2_CO that undergoes C−C coupling with other ∗H_2_CO molecules on adjacent Fe sites to yield (CH_2_OH)_2_.

### Selectivity of C_2_H_5_CH_2_OH formation

In early studies of the ECR reaction, Hori et al.^124^ observed the formation of C_2_H_5_CH_2_OH when using a Cu foil electrode. However, the reported FE (3.0% at −1.44 V vs. normal hydrogen electrode [NHE]) is low. The highest C_2_H_5_CH_2_OH FE reported thus far originates from graphene/ZnO/Cu_2_O heterostructures (Table S5).^125^ The composite was supposed to stabilize Cu(I) species and improve the selectivity of C_2_H_5_CH_2_OH. The maximum FE of C_2_H_5_CH_2_OH was 30%, attained at −0.9 V (vs. Ag/AgCl), but the partial current density was only −0.55 mA cm^−2^. Zhang et al.^126^ achieved a C_2_H_5_CH_2_OH FE of 12.1% at a potential of −1.1 V (vs. RHE) with a partial current density reaching −101.6 mA cm^−2^ by constructing bicontinuous Cu_2_O/Cu nanodomains. By using a two-step tandem catalytic system, where the CO_2_-to-CO step occurs on Ni single atoms and the subsequent CO-to-C_2_H_5_CH_2_OH step occurs on Cu_2_O, yielded a C_2_H_5_CH_2_OH FE of 15.9% along with a half-cell power conversion efficiency of 19.3%.^127^ A Br-bridged dinuclear Cu(I) complex (CuBr–4PP) that has a ligand rich in *π* electrons and robust active sites, Cu_2_(μ-Br)_2_(triphenylphosphine)_2_(4-phenylpyridine)_2_, was recently demonstrated to enable C_2_H_5_CH_2_OH production with an FE of approximately 10% at −2.2 V (vs. Ag/AgCl).^128^ The CuBr–4PP was capable of adsorbing and retaining CO_2_ owing to the phenyl group in the complex. A bridging intermediate between two Cu sites (e.g., [Cu–CHOCO–Cu]^+^) was formed, which flexibly altered the Cu–Cu distance, attracting reducing moieties to one Cu with another inserted intermediate coordinated to the other Cu. C–C coupling occurred at the bridging site in the flexible Cu binuclear complex, giving rise to C_2_H_5_CH_2_OH. The structure and Cu(I) oxidation state of the molecular catalyst were preserved during the ECR.

Besides Cu, MoS_2_ electrodes were first observed to catalyze the ECR to produce C_2_H_5_CH_2_OH as the major ECR product with an FE of 2%–5% in 0.1 M Na_2_CO_3_ acidified to pH 6.8 and 1 atm CO_2_.^129^ The terraces rather than edges of MoS_2_ were hypothesized to be the active sites for reduction of CO_2_ to C_2_H_5_CH_2_OH. Despite the progress outlined above, selective production of C_2_H_5_CH_2_OH still remains challenging.

### Selectivity of methylglyoxal (CH_3_COCHO) and 2,3-furanediol formation

CH_3_COCHO is an intermediate in the formation of 2,3-furanediol. Nickel phosphide catalysts such as NiP_2_ can convert CO_2_ into methylglyoxal with an FE of 84% at −1.0 V (vs. RHE) in 0.5 M KHCO_3_.^49^ Ni_2_P can reduce CO_2_ to 2,3-furanediol with a 71% FE at 0 V (vs. RHE), which is attributed to the multi-center synergy of densely dispersed Ni_3_ sites.^50^ The trinuclear Ni_3_ catalytic center in Ni_2_P can adsorb two C_1_ species with a shortened distance as compared to that on a metal surface, benefiting C−C coupling. In addition, the electron-delocalized Ni_3_ sites can promote electron transfer to adsorbates, which is conducive to their activation. Furthermore, transfer of surface protons between neighboring Ni_3_ sites facilitates ∗CO hydrogenation and reduces the energy penalty for C−C coupling. Despite this low overpotential and high C_3_ and C_4_ selectivity, the absolute cathodic partial current densities toward methylglyoxal and 2,3-furanediol are low, less than 1.0 mA cm^−2^, indicating a need for further study to enhance reaction rates.

### Selectivity of *n*-C_4_H_9_OH, 3-hydroxybutanal (CH_3_CHOHCH_2_CHO), and *t*-C_4_H_9_OH ((CH_3_)_3_COH) formation

*n*-C_4_H_9_OH was first observed to be generated on CuO-derived Cu with an FE of 0.056% and a partial current density of −0.08 mA cm^−2^ at −0.48 V (vs. RHE) in an alkaline medium.^130^ CO_2_ was initially electro-reduced to CH_3_CHO, which was then converted to 3-hydroxybutanal via a base-catalyzed aldol condensation reaction followed by OH^−^ elimination to form crotonaldehyde (CH_3_CHCHCHO) on Cu, with a final two-step electroreduction to *n*-C_4_H_9_OH. The highest FE for *n*-C_4_H_9_OH formation attained was up to 42% at −1.48 V (vs. Ag/AgCl) and pH 4.1 on Ni-incorporated (Cr_2_O_3_)_3_(Ga_2_O_3_).^51^ A cascade scheme was proposed; i.e., (Cr_2_O_3_)_3_(Ga_2_O_3_) generates CH_3_CHO while Ni catalyzes its aldol condensation and further hydrogenation to form *n*-C_4_H_9_OH.

During the production of *n*-C_4_H_9_OH, CH_3_CHOHCH_2_CHO was also detected as the primary ECR product in the potential range from −1.0 to −1.4 V (vs. Ag/AgCl) on Ni-enhanced (Cr_2_O_3_)_3_(Ga_2_O_3_).^51^ The maximum observed FE of CH_3_CHOHCH_2_CHO was ∼23% at −1.4 V (vs. Ag/AgCl), beyond which it was converted to *n*-C_4_H_9_OH.

Selective formation of C_4_H_9_OH is attractive with facile phase separation from water at concentrations above 9%. Multiple liquid products may be generated during ECR, and they are typically mixed with the electrolyte solvents such as KHCO_3_ and KOH. In application scenarios, additional separation and concentration steps are needed to recover the pure liquid products from solution, and the extra downstream separation steps increase the cost of industrial ECR technology.

Converting CO_2_ to (CH_3_)_3_COH has been rarely reported. Only Cu_x_Ir_1−x_ alloy NPs with oxophilic Ir-rich surfaces were found to catalyze the ECR to produce (CH_3_)_3_COH with reasonable selectivity.^53^ A (CH_3_)_3_COH FE of ∼14.8% with a partial current density of ∼ −0.21 mA cm^−2^ was attained at −0.57 V (vs. RHE) on Cu_0.48_Ir_0.52_ NPs. The C_4_ product likely evolves from a C_3_ intermediate (CH_3_)_2_CO on CuIr. Hydrogenation of CH_2_CHO∗ to CH_3_CHO∗ appears to be the potential-determining step (PDS) for formation of (CH_3_)_3_COH. Oxophilic Ir helps stabilize the O-bound intermediates to facilitate the generation of the C_4_ product.

## Computational screening of materials for ECR to yield liquid products

The pursuit of effective catalysts for ECR to produce liquid fuels has led to exploring diverse materials like two-dimensional (2D) materials and alloys^131^^,^^132^^,^^133^^,^^134^^,^^135^ However, the vast structural and compositional diversity within this domain presents an exploration challenge for simple trial-and-error approaches. Computational screening, aided by increased computation power and workflow management tools such as FireWorks and the Atomic Simulation Environment (ASE),^136^^,^^137^ offers systematic and cost-effective approaches for material evaluation and design.^138^ This section highlights how computational screening leverages DFT calculations and ML approaches for catalyst selection.

### DFT calculations for ECR catalysts screening

Since the 2000s when early works of high-throughput screening emerged,^139^^,^^140^^,^^141^ DFT-based computational screening has matured as a prominent method for identifying promising catalyst candidates for various reactions. Effective screening requires well-defined descriptors and criteria, tailored to the specific domain. This section highlights key descriptors obtained by DFT calculation for screening stable, active, and selective ECR catalysts.

Stability descriptors like formation energy, Δ*E*_f_, and dissolution potential, *U*_dis_, ensure thermodynamic and electrochemical stability, guided by negative formation energy and positive dissolution potential.^127^^,^^133^ In single-atom catalysts (SACs) or dual-atom catalysts (DACs), adsorption energy, Δ*E*_ad_ (or binding energy, *E*_bind_), of the atom(s) and its diffusion barrier on the host inform of possibility of agglomeration of the single/dual atoms into particles.^133^^,^^142^^,^^143^ Molecular dynamics (MD) simulations can observe notable structural modifications, such as the detachment of a single atom in SAC with increasing temperature.^143^

For catalyst activity, limiting potential is a straightforward activity descriptor. The computational hydrogen electrode^144^^,^^145^ helps establish the energetics of complex electrochemical reactions involving proton-electron pairs. The elementary step with the highest barrier is identified as the PDS, with its reaction barrier, Δ*G*_max_, expressed as Δ*G*_max_ = max {Δ*G*_1_, Δ*G*_2_, …, Δ*G*_N_}, where *N* is the number of elementary steps. The limiting potential, *U*_lim_, is defined as *U*_lim_ = −Δ*G*_max_/*ne*, where *n* is the number of electrons involved in the PDS. Thus, less positive Δ*G*_max_ or less negative *U*_lim_ is a straightforward indicator for lower overpotential and higher activity of the catalyst.^142^^,^^146^

Δ*E*_ad_(CO) is another popular activity descriptor. Linear scaling relationships between the Δ*E*_ad_ of adsorbates with equivalent binding species^147^ lead to Δ*G*_N_ as a linear function of Δ*E*_ad_ of a key adsorbate. This linear scaling relationship simplifies catalyst design into finding optimal Δ*E*_ad_ values, often represented in a volcano plot, a quantitative manifestation of Sabatier’s principle. The Δ*E*_ad_(CO) and Δ*E*_ad_(OH) are mostly adopted as ECR activity descriptors in the linear scaling relationship scheme.^148^^,^^149^ As another example of catalytic activity descriptor, the Δ*E*_ad_(CO_2_), or first PCET step energy, Δ*G*_(∗CO2 + H+ +_ _*e*− → ∗COOH/∗HCOO)_, was utilized to ensure CO_2_ activation.^127^^,^^150^

Selectivity is a crucial attribute of an ECR catalyst. A catalyst’s proficiency in exclusively activating specific ECR products while inhibiting others is assessed by comparing the limiting potentials of competing reactions. For instance, a positive difference in limiting potentials between ECR and HER indicates the selectivity of ECR over the HER.^133^

**Figure 5:**
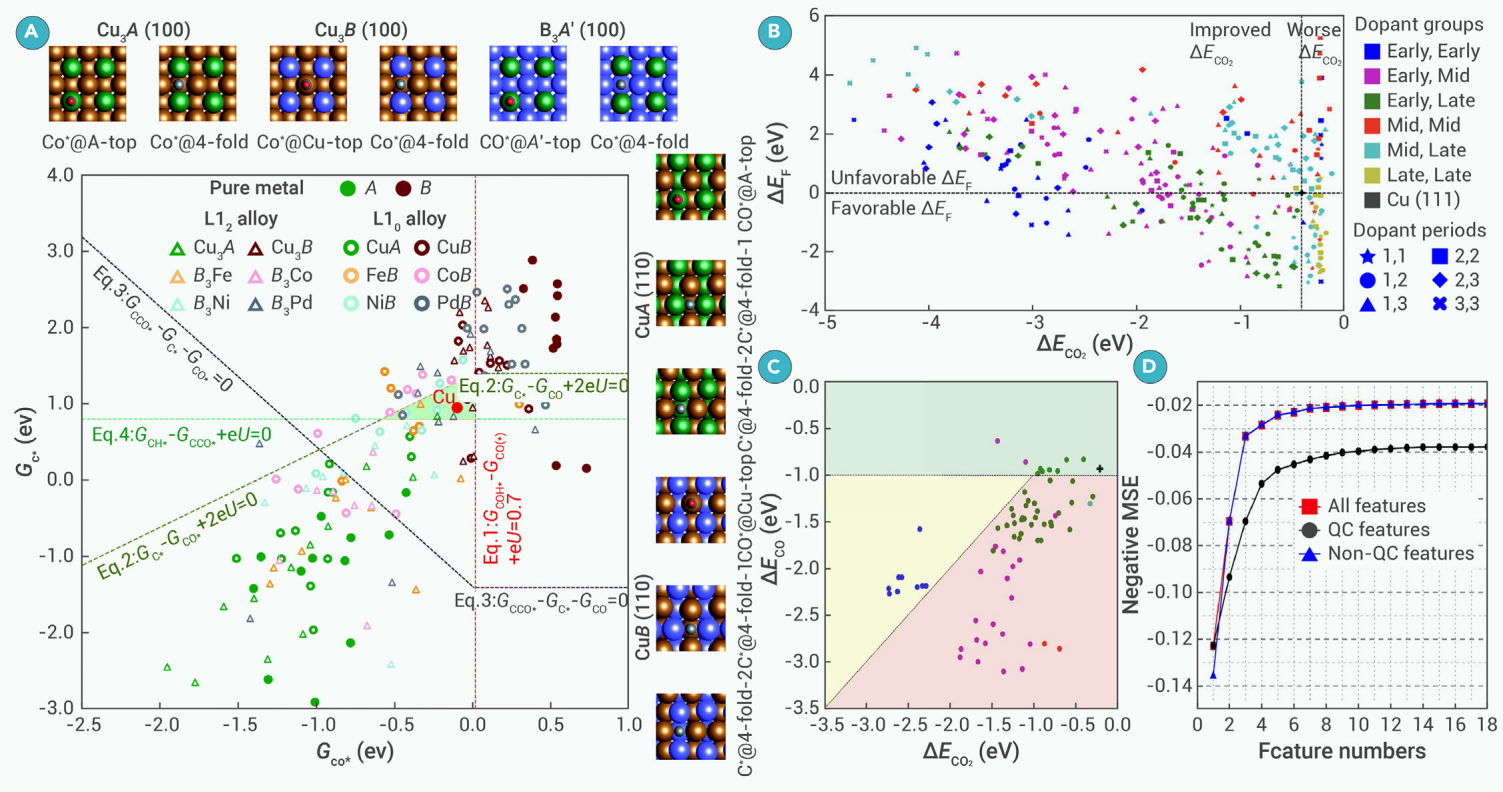
Computational screening for ECR to liquid products (A) Selectivity map of (100) L1_2_ and (110) L1_0_ alloy surfaces (*U*_RHE_ = −0.7 V, pH = 7). The light green region represents the sets of promising surfaces with primary selectivity toward C_2(+)_ products.^153^ Copyright 2022, Royal Society of Chemistry. (B) Scatterplot showing the screening results for the initial set of candidate DAAs. The dotted lines denote the energies of Cu. SAAs are considered to be DAAs with the late transition metals (TM) being Cu. Early TM, groups 3–5; mid TM, groups 6–9; late TM, groups 10–12. Gamma-point high-throughput calculations are performed for all the alloys.^157^ Copyright 2023, American Chemical Society. (C) The candidates in the green region have CO binding similar to pristine Cu (black cross). The yellow and red regions represent materials whose CO adsorption is stronger than desired, while materials in the yellow region could be outcompeted by stronger CO_2_ binding, and materials in the red regions are prone to CO poisoning.^157^ Copyright 2023, American Chemical Society. (D) Cross-validation scores (negative mean square error [MSE]) versus the corresponding feature numbers of the best ML models obtained by the modified sequential forward selection (SFS) algorithm.^158^ Copyright 2023, American Chemical Society.

Selectivity for a specific ECR product is also evaluated by comparing the limiting potentials for different ECR pathways or reaction energies at a bifurcation point.^127^^,^^148^^,^^151^ For example, comparing Δ*G*_(∗HCOOH + H+ +_ _*e*− → ∗CHO)_ and Δ*G*_(∗HCOOH → HCOOH(l)_ ensures selective HCOOH production on an SAC on supported silicomolybdic acid.^143^ Zhi et al. leveraged M−H affinity and M−O affinity as another selectivity descriptor to categorize 12 SAAs into three groups by their products: two-electron-transfer products (CO and HCOOH), more reduced C_1_ products (CH_4_ and CH_3_OH), and C_2_ products.^146^ Pederson and coworkers utilized Δ*G*_ad_(CO) and Δ*G*_ad_(C) as selectivity descriptors to screen C_2+_-selective binary alloy surfaces out of 142 candidates.^152^ Their selectivity maps, including one in Figure 5A, suggested Cu-based and non-Cu-based binary alloys in the green-colored region as the C_2+_-selective alloy.^153^ The rationale for employing Δ*G*_ad_(CO) and Δ*G*_ad_(C) as selectivity descriptors is that coupling of C and CO is a feasible route to C_2+_ chemical production. However, establishing the C−C coupling mechanism is still under debate, so careful selection of descriptors is necessary.^56^^,^^65^^,^^154^^,^^155^^,^^156^

In summary, combining stability, activity, and selectivity descriptors helps identify promising ECR catalysts. Behrendt et al. used high-throughput computations to screen over 450 dual-atom alloy (DAA) or SAA combinations embedded in Cu(111) for C_2+_-selective ECR.^157^ The initial selection prioritized thermodynamically stable and potential CO_2_-activating catalysts by excluding materials with positive Δ*E*_f_ and Δ*E*_ad_(CO_2_) greater than −0.5 eV, yielding approximately 100 viable candidates located in the bottom-left region of Figure 5B. The selection was further refined based on the proximity to optimum Δ*E*_ad_(CO) on Cu(111), −0.93 eV. Too-strong CO adsorption leads to CO poisoning, while weak CO adsorption leads to CO desorption without C−C coupling, represented in the red region and the yellow region of Figure 5C, respectively. Their work suggested potential ECR catalysts for C_2+_ products, highlighting two SAAs and seven DAAs. The results of this work, as well as other promising ECR catalysts suggested by computational screening research, are listed in Table S6 with target liquid fuels and corresponding limiting potential.

### ML methods for high-throughput screening of ECR electrocatalysts

The ML method streamlines the prediction of catalyst properties using a trained non-linear surrogate function, reducing reliance on expensive DFT calculations. This expands the exploration space in computational screening for ECR catalysts, allowing the consideration of multi-elements and complex structures.^159^^,^^160^^,^^161^^,^^162^ Notable achievements include suggestions of novel binary intermetallics^163^^,^^164^ and a DAC with an unreported structure,^165^ followed by experimental validation. This section outlines the ML approach for ECR catalyst screening and discusses strategies to address challenges in ML-aided ECR catalyst screening and future research prospects.

#### ML-aided ECR catalyst screening

ML methods have aided ECR catalyst design, where, for example, Ma et al. demonstrated their effectiveness over traditional *d*-band-center-based models in predicting Δ*E*_ad_(CO) on multi-metallic alloys.^166^ The ML-aided research process encompasses task definition, data collection, feature engineering, model selection, and validation, with emphasis on selecting appropriate features and models for better accuracy and generalizability. The development of feature and model selection has enhanced ML model prediction accuracy to below DFT uncertainty levels (∼0.2 eV), supporting the feasibility of ML in aiding computational screening.

We distinguish the term “feature” from “descriptor” for clarity throughout this section. Often, they are used interchangeably, but in the current manuscript we use feature to emphasize its role as an input for the ML model while descriptor serves as a measure of catalytic property as used in the previous section.

Feature engineering is a process of selecting and optimizing features that effectively represent materials, such as atomic radius, atomic number, coordination number, valence electrons, and electronegativity.^167^ Effective feature selection, such as Wu et al.’s identification of six dominant features out of 52 features to predict CO dimerization energy,^168^ enhances model performance and interpretability while avoiding overfitting. Feature importance weighing, Pearson correlation coefficient, or advanced algorithms like sequential forward selection have been used to refine feature sets without significantly compromising accuracy.^158^^,^^168^^,^^169^^,^^170^^,^^171^ Composite features and new feature development can further improve model accuracy.^169^^,^^172^^,^^173^^,^^174^ Ding et al. transformed 88 features into 10 mathematical expressions by a symbolic transformer, significantly enhancing prediction accuracy in limiting potentials of ECR toward HCOOH.^174^ Intrinsic descriptors, estimated from valence electrons and electronegativity, can effectively predict ECR intermediate adsorption behavior.^160^^,^^175^^,^^176^^,^^177^ Additionally, novel features like the Fukui function, complemented with work function, showed strong predictive power for Δ*E*_ad_(CO) on different facets of Ni and NiGa alloys.^173^ Context-specific features for alloys, with encapsulating information about the adsorption site environment, are vital as they determine the electronic structure of the surface via ligand effects. The microstructure of the adsorption environment can be divided into zones according to the adjacency to the binding site. Pederson et al.^178^ and Roy et al.^135^ converted the zone-divided microstructure into a feature vector, while Mok et al.^179^ and Roy et al.^177^ in their successive works incorporated the atomistic properties with zone information. Good features, containing physically and chemically relevant information, should be readily obtainable. Noh et al.^180^ and Xing et al.^149^ demonstrated the effectiveness of non-*ab initio*-based features in predicting Δ*E*_ad_ of ECR intermediates using kernel ridge regression (KRR) and gradient-boosting regression (GBR) models, respectively. Furthermore, it was reported, as shown in Figure 5D, that models utilizing features not requiring quantum chemistry (QC) calculations (non-QC features) outperform those relying on QC-derived features in predicting Δ*E*_ad_(CO) of layered alloys.^158^ However, an exception occurs when the feature vector is one-dimensional; in this case, models leveraging QC features, particularly the d-band center, demonstrate superior performance due to its strong correlation with adsorption energy.

Model selection is key in ML-aided catalyst design. Classical ML models are suitable for limited data, while neural-network (NN)-based models are better for large datasets. Blending ML models can improve performance. Ulissi and his coworkers utilized the tree-based pipeline optimization tool (TPOT) to construct composite models for accurate prediction of Δ*E*_ad_ on intermetallic compounds with various facets.^163^^,^^164^ The root-mean-square error (RMSE) for Δ*E*_ad_(CO) prediction was 0.46 eV and the RMSE for Δ*E*_ad_(H) prediction was 0.41 eV. Amaral et al.^181^ constructed ensemble models of three ML models (support vector regression [SVR], model, NN-based model, and Extreme Gradient Boosting Regressor [XGBR] model) and stacked a meta-model on them to balance the variance and bias of the prediction results, enhancing accuracy in Δ*E*_ad_(CO) and Δ*E*_ad_(CO_2_) prediction.

Crystal graph convolutional neural network (CGCNN) and other graph-based variant models like labeled-site CGCNN (LS-CGCNN) have shown remarkable accuracy for Δ*E*_ad_ prediction (Δ*E*_ad_(CO) MAE < 0.13 eV, and Δ*E*_ad_(H) prediction MAE <0.1 eV).^182^^,^^183^^,^^184^ These models convert catalyst surfaces into graphic-based data according to the constituent atom positions and their connectivity. These graphic types of data can delineate information of the local environment information effectively into their nodes and edges, which alleviates the requirement of feature engineering. However, careful selection of ML models is essential, as advanced models do not always guarantee enhanced performance. Mok et al. employed the GBR model, a classical ML approach, to predict Δ*E*_ad_ using surface microstructure-embedded input features that do not require DFT calculation, still maintaining comparable accuracy with much less training data.^179^ This implies model selection should be context-optimized.

#### Challenges in ML-aided ECR catalyst screening and outlook

ML-aided screening has gained prominence, yet several challenges should be addressed, including data scarcity, insufficient catalytic performance descriptors, and the gap between computation and experimentation.

One of the main challenges in harnessing ML models is securing a database with both sufficient quantity and high quality. High quality means consistency within the data-generation procedure and unbiased distribution of data within chemical space. In this context, pre-established databases in surface chemistry, such as the datasets from the Open Catalyst Project (OC20,^185^ OC22,^186^ ODAC23,^187^ OC20-Dense,^188^ OC20NEB,^189^ and OMat24^190^) and GASpy,^163^ facilitate ML-aided research. Comprehensive datasets from computation can complement success bias often present in experimental data, where records of failures are frequently absent. Including failure data is essential for robust ML models. However, the specificity of heterogeneous catalytic systems often limits database utilization, necessitating efficient use of limited data or data augmentation through accelerated computational processes.

Active learning (AL) effectively trains ML models with minimal data by selecting informative data with a greedy selection algorithm that will be validated by human intervention and utilized to update and retrain the model. This iterative process optimizes the search trajectory in chemical space. The success of AL in ECR catalyst design includes Ulissi et al.’s exploration of over ∼1.7 million distinct binding sites of 1,499 bimetallics.^163^^,^^164^ They selected ∼20,000 binding sites for each of the CO and H adsorptions by searching a surface with a near-optimal Δ*E*_ad_, which is a very small selection considering the size of the search space. AL also demonstrated its viability in smaller chemical spaces, achieving a 70% success rate in discovering HCOOH-selective dual-metal-site catalysts (DMSCs) from a dataset containing 282 DMSCs over three iterations.^174^

Transfer learning (TL) addresses data scarcity challenge by transferring knowledge efficiently from the source domain to the target domain, enhancing performance through fine-tuning the parameters of the model trained in the source domain. Although the TL framework has not been fully exploited in ML-aided ECR catalyst screening for liquid fuels, a proof-of-concept study employed TL with a pre-trained model for the prediction of Δ*E*_ad_(CO).^191^ Discussions on transferability include applying reaction energy-prediction models trained on metal-zeolite systems to metal-organic frameworks (MOFs), 2D materials, and molecular complex systems^192^ and applying a Δ*E*_ad_ prediction model trained for Cu-based binary alloys to tertiary alloys.^193^ Since the success of TL is largely dependent on relevancy between the domains, the discussions on the transferability imply the potential of applying TL in designing ECR catalysts to produce liquid fuels.

ML potential (MLP) provides a computationally efficient approach to predict the total energy and atomic forces of a system without solving the Kohn-Sham equation,^194^ significantly accelerating various simulations like geometry optimization, NEB calculation, and MD simulation.^189^^,^^195^ By overcoming DFT’s limited scalability, MLP facilitates exploring vast chemical spaces for complex catalyst systems.^196^ For instance, minimum energy paths (MEPs) for the conversion of CO_2_ to ethanol were identified on several types of metal oxide surfaces, including surfaces with oxygen vacancies, reduced metal oxides, and doped metal oxides.^197^ The high computational efficiency of MLP enables the examination of multiple MEPs for each reaction step, ultimately leading to the identification of an alternative path with a 40% reduced activation energy. MLP also helps identify catalyst surface structures and active sites,^188^^,^^198^^,^^199^^,^^200^^,^^201^ as shown by Lan et al.’s graph-based MLP models that reduce computational load significantly.^188^ State-of-the-art MLP models,^202^^,^^203^^,^^204^ pre-trained on OC20 datasets, exhibits comparable or enhanced accuracy compared to heuristics-based DFT calculations with reduced computational load by orders of magnitude. This result underscores the potential of MLP to accelerate data generation while retaining reliability.

Selecting suitable electrocatalytic descriptors, beyond Δ*E*_ad_(CO), is crucial for ECR catalyst screening. While Δ*E*_ad_(CO) is a common ECR activity descriptor,^205^ its limitation as the sole descriptor has been noted.^159^^,^^160^^,^^178^ Recent ML models predict various activity descriptors, such as Δ*E*_ad_ of adsorbates (i.e., CH_*n*_O (*n* = 1–3), COOH, HCOO, COCOH, etc.),^135^^,^^149^^,^^177^^,^^181^^,^^192^ CO dimerization energy and CO hydrogenation energy,^168^ and the limiting potential of the reaction.^165^^,^^174^ The difference between predicted limiting potentials of products or between predicted Δ*E*_ad_ of intermediates has been employed as a measure of selectivity. Also, descriptors for selectivity and stability have been suggested. For example, product probability on zeolite was predicted to determine product selectivity between CH_3_OH and CH_4_.^192^ Also, it was reported that the difference between binding energy and cohesive energy of a metal at the active center of SACs is a stability descriptor.^165^

Bridging the gap between computational screening and experimental application remains challenging. Exclusive reliance on computational data fails to capture the role of operating conditions (synthesis, type of cell, membrane, electrolyte, applied potential, and morphological change).^206^ Accelerated by ML, multi-scale modeling techniques such as microkinetic modeling or MD enable the extrapolation of atomistic-level insights to a more practical, application-oriented scale, as has been comprehensively outlined.^207^ Additionally, incorporating experimental data offers a complementary approach. There have been attempts to leverage experimental data from literature or databases to train ML models and predict catalytic performance, including the ECR FE and rate.^208^^,^^209^ The integration of computational and experimental data can be facilitated by autonomous laboratories and high-throughput experimentation combined with ML.^210^^,^^211^^,^^212^^,^^213^

ML’s support in computational screening is shown by the list of candidate catalysts suggested in Table S6, which could be of interest for experimental investigation. It should be noted that some ML studies did not specify the type of target products being pursued. However, since they adopted the property of Cu, which is known to reduce CO_2_ to various multi-C products, as a benchmarking material, we present their screening results (Table S6).

## Strategies for tailoring CO_2_ reduction pathways toward liquid products

### Modification of catalysts

#### Surface functionalization

Surface modification plays multiple roles in regulating ECR performance. It can affect mass transfer of reactants by modifying hydrophobic groups on a catalyst surface to prevent proton migration to the active sites or by modifying functional moieties to promote CO_2_ adsorption.^214^ It may also change the intrinsic activity of metal catalysts, such as by reducing the activation energy of the rate-determining step of the reaction through modification of the active sites, alteration of the surface chemical state, or changing the configuration of intermediates on the catalyst surface.^215^ Thirdly, the species used for surface modification may confer potent new active sites to enhance the ECR catalytic activity.^216^ Fourthly, incorporation of modifiers in the surface of catalysts can enhance their stability toward leaching, aggregation, or rapid reduction during ECR.

**Figure 6:**
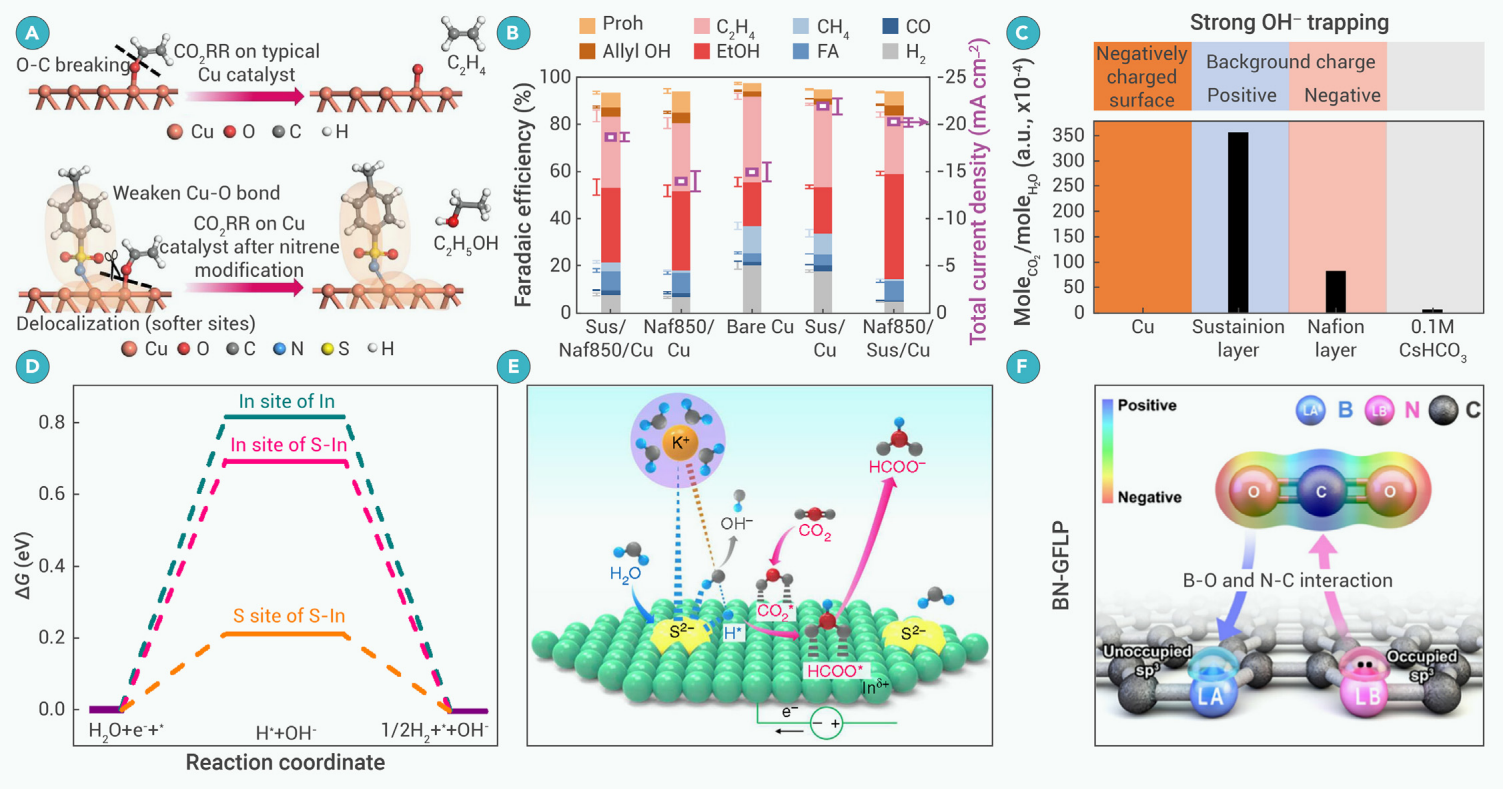
Surface functionalization, heteroatom engineering strategies for ECR to liquid products (A) Schematic illustration of the adsorbed CH_2_=CHO∗ intermediate on a Cu surface, as the bifurcation point toward the ethylene or ethanol pathway during ECR. On a typical Cu surface, the O−C bond energy is lower than that of the O−Cu bond and easier to cleave, thus producing ethylene. On a nitrene-functionalized Cu surface, the electron delocalization makes the Cu site a “softer” acid and thus reduces the O−Cu bond energy, so the O–Cu bond is easier to cleave than the O−C bond and the selectivity is switched to produce ethanol.^217^ Copyright 2024, American Chemical Society. (B) ECR performance at −1.15 V vs. RHE using stacked ionomers on Cu in the presence of 0.1 M CsHCO_3_ electrolyte.^218^ Copyright 2021, Springer Nature. (C) Schematic illustration of Naf850/Sus/Cu and Sus/Naf850/Cu in terms of local CO_2_/H_2_O ratio and spatial charge configuration. Areas shaded orange, blue, red, and gray correspond to Cu, Sus., Naf., and electrolyte solution, respectively.^218^ Copyright 2021, Springer Nature. (D) Gibbs free energies for the formation of H∗ on pure In(101), In, and S sites of S−In(101) surfaces.^219^ Copyright 2019, Springer Nature. (E) Schematic illustration for the role of S^2−^ in promoting water dissociation and H∗ formation for the reduction of CO_2_ to formate.^219^ Copyright 2019, Springer Nature. (F) Schematic of molecular interactions between BN-GFLP and CO_2_.^220^ Copyright 2024, American Chemical Society.

The cleavage of the CH_2_ = CHO∗ intermediate, connected to Cu via a Cu−O−C bond, determines the production of C_2_H_4_ or C_2_H_5_OH. Modifying the Cu surface with methyl benzenesulfonyl azide is beneficial for constructing an electron-delocalized state on the Cu surface, facilitating the delocalization of electrons from Cu to the methyl benzenesulfonyl azide molecule (Figure 6A).^217^ This promotes the cleavage of the Cu−O bond, leading to the formation of C_2_H_5_OH. Methyl benzenesulfonyl azide-functionalized Cu (Cu=N) achieved an FE_C2H5OH_ of 45% at −0.82 V (vs. RHE), which is 3.2 times that of bare Cu.

The microenvironments of Cu-catalyzed CO_2_ electrolysis can be tailored for C_2_H_5_OH selectivity by introducing functional layers. Modifying Cu with metal hydroxide of Pourbaix stability (e.g., Ce(OH)_*x*_) facilitates water dissociation and hydrogen adsorption (H_ad_), but it is unlikely to impact the adsorption of carbonaceous intermediates.^29^ The H_ad_ attacks ∗HCCOH, forming ∗HCCHOH toward C_2_H_5_OH instead of C_2_H_4_. Coating Cu with ion-conducting polymers (ionomers) such as Naf. (a perfluorosulfonic acid, cation-conducting ionomer) and Sustainion (Sus., a polystyrene vinylbenzyl methylimidazolium, anion-conducting ionomer) was demonstrated to create favorable microenvironments (higher local CO_2_/H_2_O ratio and pH) for selective C_2_H_5_OH and C_2+_ production.^218^ The surface roughness of Cu remains unaffected, and the electrochemical surface areas for bare Cu and all ionomer-coated Cu samples were close. The partial current density for ECR increases with the local CO_2_/H_2_O ratio following the order of bare Cu < Naf.1100 (Naf. with 1,100 g mol_eq_^−1^ equivalent weight)/Cu < Sus./Cu (Figure 6B). The enhanced C_2+_ selectivity (including C_2_H_5_OH and C_2_H_5_CH_2_OH) for Naf.1100/Cu and the further enhancement for Naf.850/Cu are likely due to a Donnan exclusion at the interface between the Cu and the ionomer. Naf.850 relative to Naf.1100 possesses a higher background charge density and stronger Donnan exclusion, leading to more local accumulated OH^−^ anions and increased C_2+_ selectivity. An optimized coating with Naf. as the outermost layer followed by Sus. (i.e., Naf.850/Sus./Cu) provides the best C_2+_ FE, originating from its high local ratio of CO_2_/H_2_O, greater amounts of OH^−^, and hindrance of HCO_3_^−^ transport from the bulk electrolyte to the reaction surface (Figure 6C).

Graphene nanodisks with oxygen-containing groups (−COOH, −OH, C–O–C, and –C=O) facilitated HCOO^−^ yield with an FE of 90% at −0.68 V (vs. RHE).^221^ The C atoms adjacent to these moieties were surmised to be the active center where the main species contributing to the activity is the carboxyl group. In another work, modification of N-doped CNTs with polyethylenimine (PEI) enhanced the FE toward HCOO^−^ from 59% with a partial current density of −3.0 mA cm^−2^ to 85% with a partial current density of −7.2 mA cm^−2^ at a potential of −1.13 V (vs. RHE).^222^ The N atoms in PEI were hypothesized to stabilize the singly reduced intermediate CO_2_^−^ via the formation of NCNT–N–C(O)O∗ … H–N–PEI, with the PEI coating concentrating CO_2_ near the electrode surface and boosting the reaction rate.

Surface modification also protects active sites from deterioration during CO_2_ electrolysis. For example, the reduction-induced deactivation of cobalt phthalocyanine (CoPc) during ECR was alleviated by introducing four amino groups at the *β* sites of the phthalocyanine ligand.^223^ The electron-donating −NH_2_ substituents lowered the reduction potential of CoPc. in contrast to pristine CoPc dispersed on C nanotubes (CNTs), which yielded a CH_3_OH FE of only 0.6% after 5 h of electrolysis. The modified CoPc catalyst maintained good stability with minor degradation in CH_3_OH FE after 12 h of reaction. Coating Cu with poly(4-vinyl pyridine) (P4VP/Cu) increases the electrode robustness with the pyridyl groups in P4VP bound to the Cu sites, screening the Cu against leaching into the electrolyte.^224^ As a consequence, HCOO^−^ production by P4VP/Cu was sustained for 30 h, exceeding the prior performance reported for Cu, Sn, and Bi electrodes. Likewise, depositing a C layer over a CuO_x_ surface was found to stabilize Cu^+^ species, leading to a C_2_H_5_OH FE of 46% with a partial current density of −166 mA cm^−2^.^225^

In addition, since electrolytes containing alkali metal cations can induce carbonate deposition on the cathode and block the CO_2_ mass transfer channel, the conversion of CO_2_ to HCOOH in acidic electrolyte without alkali metal cations can be achieved by loading polyelectrolyte on the surface of catalysts. Cross-linked poly(diallyldimethylammonium chloride) (c-PDDA)-modified In NPs achieve 75% FE_HCOOH_ at −1.84 V (vs. SHE) in 0.1 M H_2_SO_4_.^226^ The cation sites carried by the polymer layer play a similar role to that of the alkali cations dissolved in the acidic electrolyte in enabling CO_2_ reduction (that is, inhibiting the migration of H and enhancing the electric field of the Stern layer).^227^^,^^228^

For surface functionalization, two issues need to be considered. Some modified systems exhibit lower current densities than bare Cu references, suggesting unwanted blocking of active sites by the modifiers. Deconvoluting whether a modified catalyst possesses better intrinsic activity than the pristine catalyst requires metrics such as the partial current density normalized against ECSA and the turnover frequency (TOF).^229^

#### Heteroatom engineering

Introducing heteroatoms into a catalyst can regulate product formation by adjusting the electronic structure of the catalysts and/or the adsorption dynamics of the intermediates. For example, Co-doped Bi nanosheets achieved ∼90% selectivity for HCOO^−^ at a current density of −200 mA cm^−2^ and a low overpotential of ∼1.0 V. Compared to bare Bi, Co-doped Bi has a stronger binding energy for the key intermediate OCHO∗, thus requiring a lower applied potential for the conversion of CO_2_ to HCOOH.^230^ Replacing O in Sn-based oxides with less electronegative chalcogens S and Se to form SnS_x_^231^ and SnSe_2_^232^ increases the electronic conductivity, improves CO_2_ adsorption, and decreases ∗OCOH adsorption energy, thus boosting HCOO^−^ production. S doping may also hamper hydrogen or water adsorption (Figure 6D). S-doped In (S-In) catalyst was reported to enhance the HCOO^−^ yield, outperforming bare In, Se-In, and Te-In catalysts (Figure 6E).^219^ To address the over-reduction of S dopants at large overpotentials (and a resulting rapid increase in HER), phase engineering of a SnS (*π*-SnS) pre-catalyst was demonstrated to stabilize S dopants in the derived metallic Sn for selective acidic CO_2_-to-HCOOH electrolysis at −1 A cm^−2^.^233^ S, being more electronegative than Cu, withdraws electrons from Cu, allowing the negatively charged S atom to adsorb a CO_2_ molecule or a hydride. The adsorbed CO_2_ is selectively reduced to HCOO^−^ on the neighboring Cu site through a protonation of the C atom or alternatively through a ∗H pathway.^234^

Metal-like doping of Cu with boron (B) and phosphorus (P) stabilizes ∗CO and alters the reactivity and selectivity for C_2_H_5_CH_2_OH production.^235^ B doping promotes C_2+_ products, while P doping leads to faster ∗CO consumption. B doping reduces the thermodynamic energy barrier in the CO hydrogenation reaction, thereby improving the selectivity of C_2_H_5_OH on Cu(111).^236^ Electrons transfer from Cu to B and gather around the B atom, which acts as the charge-transfer medium. The intermediate isomerization step puts C in a reverse charge state, making C−C coupling and C_2_H_5_OH formation more favorable. As opposed to doping with S, P, and O, an N-engineered Cu catalyst was demonstrated to exhibit the best CO_2_-to-C_2+_ productivity with ampere-level current.^237^ Engineering with N atoms promoted ∗CO adsorption on both the bridge and atop sites of Cu, lowering the energy barrier for C−C coupling. Additionally, B and N co-doping can construct surface-frustrated Lewis pairs (FLPs), with B and N serving as Lewis acid and Lewis base sites, respectively (Figure 6F).^220^ The lone-pair electrons of the O atom in CO_2_ can be donated to the surface Lewis acid site, while the C atom in CO_2_ can receive electrons from the Lewis base site. The synergistic effect of FLPs reduces the free energy for CO_2_ activation and C−C coupling. B and N co-doped graphene achieved an FE of 87.9% for C_2+_ products at the potential of −0.7 V (vs. RHE), with an FE of 50.4% for *n*-propanol. In contrast, B-doped graphene and N-doped graphene only produced formate.

#### Design of bi(multi-)metallics

Bi(multi-)metallic catalysts can enhance performance relative to their monometallic counterparts via electronic effects (including the ionic ligand effect, covalent ligand effect, and strain effect) and geometric effects (including the ensemble effect, ordering effect, and steric effect). These effects usually function concurrently to facilitate adsorption and catalysis. Addition of another element can alter the electronic structure, thereby influencing the binding affinity of adsorbed intermediates. Bi(multi-)metallics may break the scaling relationships among adsorbates to lower the overpotential. The atomic number and/or configuration of active sites may also be changed, which varies the nature of adsorption of the intermediates at the surface. In addition, adjacent metallic sites can impart distinct catalytic roles, enabling synergistic activity (sequential catalysis on bi(multi-)metallics is discussed in the following section). Incorporating a metal that has a higher oxophilicity and strong M–Cu bonding energy (e.g., early 3*d* metals such as Sc, Ti and *p*-group metals such as Al, In, and Bi) tends to stabilize Cu against reconstruction during the ECR.^238^

Bimetallic Cu-M (M refers to *p*-block metals such as Bi,^239^ Sn,^240^ and Pb^241^) have been reported to enhance HCOOH formation. For example, the addition of Bi in Pd to form intermetallic Pd_3_Bi prevents the overbinding of ∗H or ∗CO on Pd and avoids CO poisoning, substantially lowering the ∗CO adsorption energy from −1.11 to −0.1 eV,^239^ leading to selective reduction of CO_2_ to HCOOH with an FE approaching 100%. Incorporation of Ag in Cu can induce compressive strains on Cu surface atoms, shifting the valence band structure of Cu to deeper levels, dampening ∗H adsorption and thereby lowering the HER activity,^242^ while the FE for multi-C oxygenates (mainly CH_3_COO^−^ and CH_3_CHO) was significantly improved. This was ascribed to the lowered rates of C−O bond cleavage as a result of HER inhibition and the depressed oxophilicity of the compressively strained Cu, which weakens the deep reduction of the carbonyl-containing oxygenates. Using Pd_83_Cu_17_ alloy electrocatalyst and 1-butyl-3-methylimidazolium tetrafluoroborate ([Bmim]BF_4_) aqueous solution catholyte promotes the selective formation of CH_3_OH via ECR with an FE up to 80%,^243^ whereas the catalyst is inactive for ECR and produces predominantly H_2_ in 0.5 M NaHCO_3_ and Na_2_SO_4_ aqueous solutions. In a 0.5 M KHCO_3_ aqueous solution, an impressive CH_3_OH FE of ∼77.3% at a low reduction potential of −0.3 V (vs. RHE) was achieved on CuGa_2_.^244^ It was hypothesized that the adsorbed H at the Cu site can spill over to the Ga site and then further react with the adsorbed ∗OCHO to produce CH_3_OH.

The emerging area of high-entropy alloys (HEAs), which confine multiple atomic species into the same lattice, can provide multi-center active site cooperation. Compositional and structural engineering of HEAs enables optimization of catalytic properties. As an example, a PdCuAuAgBiIn HEA aerogel (Figure S1A) was reported to be active for ECR to yield HCOOH with an FE of 98.1% at −1.1 V (vs. RHE).^226^ The good activity was likely from the interactions between the different metals and the surface unsaturated sites, optimizing HCOO∗ intermediate adsorption/desorption for enhanced HCOOH production.

#### Construction of tandem/cascade architectures

In recent studies, cascade catalysis has enhanced the selectivity of C_2+_ liquid products. In a typical tandem catalytic system, a mainly CO-producing metal-based catalyst (e.g., Au,^245^ Ag,^246^ Pd,^247^ Zn,^248^ and Fe-porphyrin^112^) is coupled with Cu, allowing ∗CO or CO molecules^249^ formed by the ECR to be transferred and reduced on the Cu to generate high-order hydrocarbons and oxygenates (Figure S1B). Increasing the density of CO-selective sites on Cu improves ∗CO coverage at the expense of ∗H, expediting C−C coupling reactions and favoring oxygenate yield over hydrocarbons. On a tandem electrode (e.g., Cu_x_Zn and Ag-Cu_2_O) with low hydrogen-generation rates and small overpotential regions (e.g., Au/Cu), the selectivity of oxygenated C−C coupled products (i.e., C_2_H_5_OH) is promoted over hydrogenated C−C coupled products (i.e., C_2_H_4_).^250^ Another report demonstrated that C_2_H_5_OH is selectively enhanced through the coupling of ∗CH_x_ and ∗CO at the terraces of Cu(111)-Ag in the CO-enriched environment provided by the Ag spheres.^251^ Distinctively, on Cu(100)-Ag, C_2_H_5_OH is selectively generated following the same pathway at the edges and corners of Cu(100), while C_2_H_4_ is produced at the terraces.

Another type of tandem system is Cu(II)- and Bi(III)-based MOFs, which afford a C_2_H_5_OH FE of 28.3%.^252^ The improved C_2_H_5_OH formation can be explained by the reduction of CO_2_ at Bi to HCOO^−^, which is subsequently transported to Cu and further reduced to alcohol. However, the poor stability of the reported electrodes remains an issue.

Tandem catalysis has also been achieved using with interdigitated Au and Cu microfabricated electrodes on an insulating SiO_2_ substrate.^253^ CO formed on the Au lines can be further reduced by the Cu lines. Reducing Cu coverage shifts the C_2+_ product distribution to oxygenates over C_2_H_4_. Alternatively, sequential catalysis can be achieved by combining two tandem electrolytic cells^127^ that convert CO_2_ to CO and CO to C_2_H_5_CH_2_OH, respectively.

A cascade system integrating CO_2_ reduction and CO reduction steps enables high-efficiency production of C_2_H_5_CH_2_OH through the precise voltage control of the CO_2_ electrolyzer system. The FE toward C_2_H_5_CH_2_OH reached 15.9% with a partial current density of −20 mA cm^−2^ and half-cell energy conversion efficiency up to 19.3%.^254^ A medium-temperature solid-oxide electrolysis cell avoids carbonate formation (2OH^−^ + CO_2_ → CO_3_^2−^ + H_2_O) and achieves a reasonable single-pass conversion (i.e., the percentage of C that is transformed to reduction products in a single pass-through electrolyzer) during the CO_2_-to-CO conversion step (Figure S1C).^255^ A cascade approach was also demonstrated for HOC_2_H_4_OH production using CO_2_ instead of fossil-fuel-derived C_2_H_4_ as the feedstock with a production rate of 0.5 mmol h^−1^.^256^

#### Defect regulation

Vacancies and low-coordination sites enhance electron capture and accessibility of selective sites.^257^ Oxygen-vacancy-rich In_2_O_3_ exhibited better HCOOH production performance during ECR than its less defective counterpart, as oxygen vacancies reduced the barrier for the formation of ∗COOH intermediates.^258^ The introduction of S vacancies in In_4_SnS_8_ boosted HCOOH production with an FE of 91% at −1.0 V (vs. RHE) (Figure S1D),^259^ facilitating CO_2_ adsorption and favoring the ∗HCOO-mediated HOOH pathway while suppressing the competing HER (Figure S1E). Relatedly, the creation of S vacancies in a Cu_2_S_1−x_ electrocatalyst afforded enhanced C_2_H_5_OH FE of 73.3% at a low overpotential of 0.19 V,^260^ with DFT calculations showing strong electron-donating ability of abundant Cu^δ+^ catalyst surface sites, which reduce the energy barrier of the C−C coupling step to produce C_2_H_5_OH. Alternatively, the presence of surface Cu vacancies in a Cu_2_S–Cu core-shell nanostructure was shown to increase the energy barrier toward C_2_H_4_ formation, whereas the C_2_H_5_OH pathway was unaffected, thus boosting the selectivity of C_2_H_5_OH formation.^43^

Lattice dislocations can improve conductivity, create more active sites, and regulate the adsorption energetics of reactants and intermediates.^261^ A Bi nanowire catalyst with lattice dislocation on copper foam showed a 95% FE for HCOOH production at −0.69 V (vs. RHE).^262^ There are more undercoordinated step sites on metal catalysts with high dislocation density, and the energy barrier for the formation of CO_2_ reduction intermediates is lower.^263^ Grain boundaries formed by adjacent nanocrystals are also important active sites that promote electrocatalytic CO_2_ reduction by regulating the binding energy of reaction intermediates.^264^

#### Selective facet exposure

Some facets may break the adsorption-energy scaling relations, thus promoting activation and reduction of CO_2_ and key intermediates. Designing materials with tailored crystal facets is a rational strategy to regulate product selectivity. Wei et al.^159^ tailored the growth orientation of the crystal facets of an In-Cu electrocatalyst by controlling the In/Cu ratio. Only the In(101) facet in the In_1.5_Cu_0.5_ catalyst effectively stabilized the ∗OCHO intermediate, which was favorable for HCOOH formation. The exposed (111) facet on Bi monolayers was observed to have a lower CO_2_ reduction overpotential (0.58 V) than the HER (1.28 V) and facilitates HCOOH formation through an ∗OCHO intermediate, while the (011) facet of thick Bi layers binds the intermediate too strongly, thus poisoning the catalyst.^265^

On Cu electrodes, ECR shows a strong facet dependence for the formed product, which can be described according to the binding energies of O∗, H∗, C∗, OH∗, CO∗, OCCOH∗, and COOH∗.^266^ Cu(111) favors CH_4_ and HCOO^−^, while Cu(100) favors C_2_H_4_ formation. The (111) steps on (100) facets are mainly selective to C_2_H_4_, whereas the (110) steps on (100) planes are more inclined to generate C_2_H_5_OH.

Cu(110) enables secondary C_2_ products such as CH_3_COO^−^, CH_3_CHO, and C_2_H_5_OH.^267^ The (111)−(100) motif in periodic face-centered cubic Cu is proposed as an active ensemble for promoting C_2+_ yield due to its favorable formation energy for ∗OCCOH.^268^ Among the four planar-square (p-sq), step-square (s-sq), concave-square (cc-sq), and convex-square (cv-sq) facets that were extracted as slab models to calculate the energetics of ∗CO coupling over oxide-derived Cu by DFT, the s-sq provided the strongest affinity to bind ∗CH_2_CHO for further protonation to C_2_H_5_OH.^269^ The n(100) × (110) steps have been also identified as active sites for C_2_H_5_OH production.^266^ Wrinkled Cu ﬁlms with exposed (310) facets were shown to deliver impressive C_2_H_5_OH selectivity with an FE reaching 40% at −0.9 V (vs. RHE).^270^ Such high selective production of C_2_H_5_OH was calculated to be due to the high density of step sites with abundant (310) facets, which possess a low C−C coupling energy penalty (0.5 eV) toward C_2_H_5_OH formation.

#### Surface oxidation state modulation

A metastable oxide/hydroxide layer on Sn^271^ and Pb^272^ electrodes enhances HCOO^−^ formation, whereas RuO_x_·IrO_x_ and RuO_x_/IrO_x_ modified with Cu and Cd adatoms produced (CH_3_)_2_CHO and CH_3_OH.^273^ Theoretical calculations suggest that the major intermediate on transition metal oxides (TMO) is ∗OCHO (for HCOOH formation) instead of ∗COOH (for CO formation). At regions with ∗OH adsorption energies lower than −0.34 eV, CH_3_OH is preferably generated on TMOs, whereas HCOOH is favored at ∗OH binding energies higher than −0.21 eV.^274^ Among the various tin oxides, Sn_3_O_4_ was reported to deliver the highest HCOOH FE of 97.7% at −0.9 V (vs. RHE).^275^ The superior activity was attributed to the collaborative effect of Sn^2+^ and Sn^4+^, which bind the intermediates weakly and strongly, respectively.

**Figure 7:**
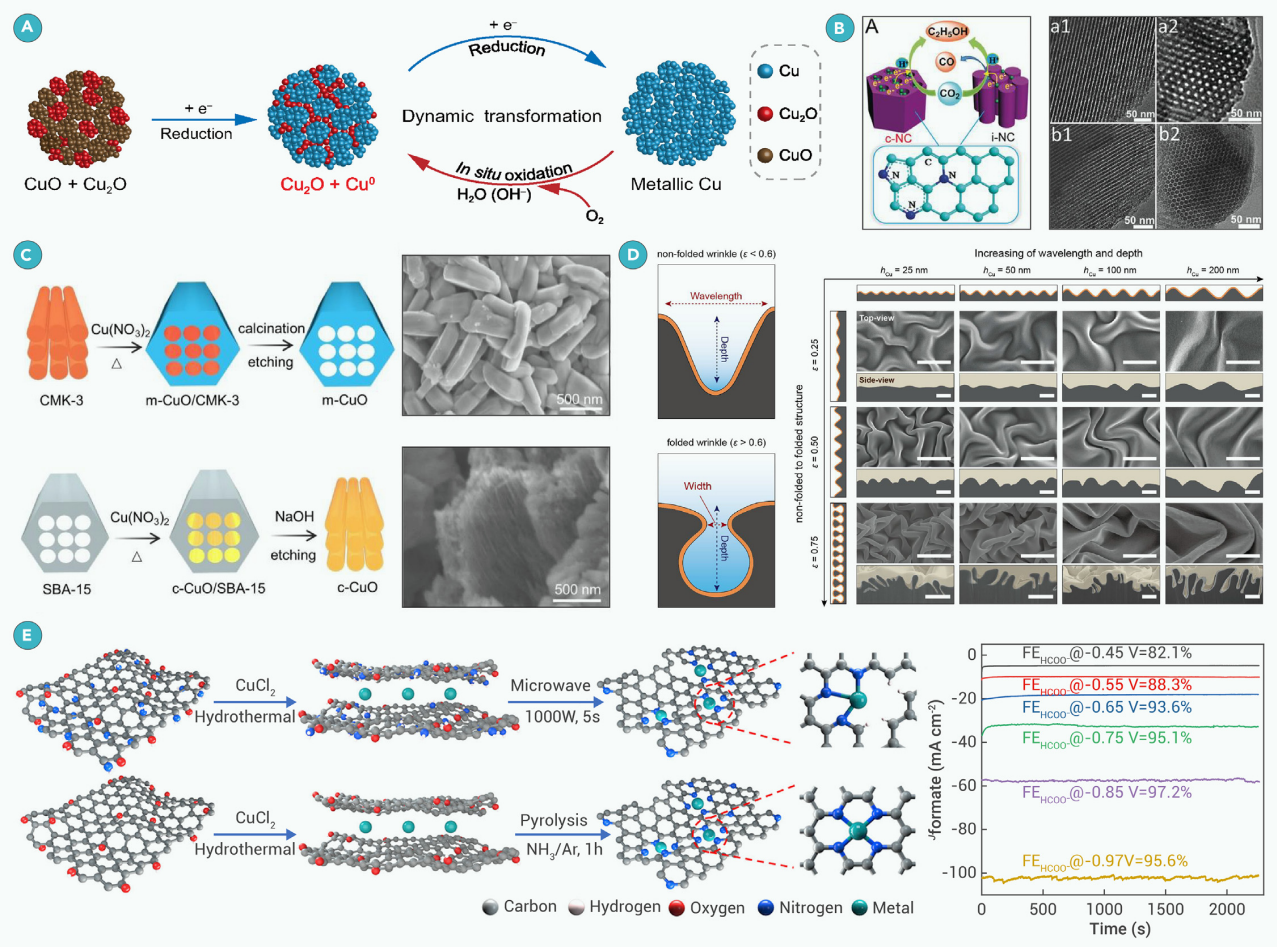
Oxidation state modulation, nanoconfinement control and coordination engineering strategies for ECR to liquid products (A) Schematic diagram of dynamic reduction and re-oxidation mechanism of copper for the Cu_2_O−Cu^0^ catalyst during ECR.^284^ Copyright 2024, Springer Nature. (B) Illustration of cylindrical mesoporous N-doped C (denoted as c-NC) and inverse mesoporous N-doped C (denoted as i-NC) for CO_2_ electroreduction. Transmission electron microscopy (TEM) images of (a1, a2) c-NC and (b1, b2) i-NC viewed along (a1, b1) [110] and (a2, b2) [100] directions.^285^ Copyright 2017, Wiley-VCH GmbH. (C) Schematic illustration of the template routes to *m*-CuO and c-CuO catalysts and SEM and TEM images.^286^ Copyright 2024, Wiley-VCH GmbH. (D) Feature shape and dimension of Cu wrinkle model.^287^ Copyright 2023, Wiley-VCH GmbH. (E) Schematic illustration of the preparation strategy for PSB-CuN_3_ and PS-CuN_4_ and chronoamperometric responses of PSB-CuN_3_ in CO_2_-saturated electrolyte at different potentials. The potential was corrected with 100% *iR* compensation.^288^ Copyright 2023, Springer Nature.

The thermodynamics and kinetics of the CO_2_ reduction and ∗CO dimerization on Cu were calculated to be greatly enhanced in the presence of subsurface oxygen through orbital overlapping between oxygen from Cu and CO_2_ molecules.^276^ As opposed to Cu^2+^, which produces mainly CH_4_,^277^ a synergy between Cu^+^ and Cu^0^ (with an optimal Cu^0^-to-Cu^+^ surface ratio of ∼0.5) can accelerate C_2+_ formation (e.g., C_2_H_5_OH).^278^ Cu^0^ promotes CO_2_ activation by lowering the thermodynamic energy barrier, while Cu^+^ strengthens ∗CO binding, thereby boosting C−C coupling. In another study by Arán-Ais et al.^279^ Cu^+^-Cu^0^ pairs and defects were shown to enable higher C_2_H_5_OH production. When the reduction of Cu(I) is minimized at 278.15 K during pulsed ECR, C_2_H_5_OH dominates the C_2_ products. Alternatively, it has been suggested that strongly bound bridge-adsorbed CO (CO_B_) occurs on low-coordination Cu^0^, while a Cu^+^ site binds linearly to adsorbed CO (CO_L_).^280^ The adjacent CO_B_ and CO_L_ are readily coupled to form C_2+_ products. From these scenarios, strategies such as oxygen plasma treatment,^281^ elemental doping,^246^ anodic pulse,^282^ and engineering of mixed metal oxide interfaces^283^ have been developed to create and stabilize Cu^δ+^. Selection of some supports that possess rapid electron transport channels has been postulated to hinder the accumulation of electrons around Cu^δ+^ sites, thus protecting them against electrochemical reduction.^277^ An alternative avenue is to re-oxidize lower-valence-state species back to the original higher valence state through pulsed electrolysis.^282^ However, how to maintain the mixed oxide states during prolonged operation and under aggressively reducing conditions still remains an issue. Recently, He et al.^284^ reported the stable existence of Cu_2_O-Cu^0^ catalysts during ECR, where Cu^+^ formation was speculated to be due to *in situ* re-oxidation (Figure 7A) from the small amount of O_2_ generated from the oxidation of the anode H_2_O passing through the membrane to the cathode. The high concentration of OH^−^ on the surface of the catalyst might also promote Cu^0^ re-oxidation.

#### Nanoconfinement control

Nanoconfinement describes confining a reaction within nanosized regions, with dimensions of less than 100 nm, to break the linear scaling limitation in electrocatalysis.^289^ In nanoconfined spaces, the transport of HCO_3_^−^ (into) and generated OH^−^ is hindered, resulting in higher local pH values compared to the bulk solutions. This suppresses the HER while lowering the C–C coupling energy barrier and facilitating the reduction of the ∗CO intermediate to C_2+_ products (including C_2_H_5_OH and C_3_H_7_OH).

Creation of porosity and/or cavities can prolong intermediate residence time and speed up the reactions of adsorbed CO_2_ and intermediates to create C_2+_ hydrocarbon and alcohol products (Figure 7B).^290^ Construction of surface nanocavities can stabilize Cu^+^ and prevent its reduction under ECR reaction conditions, thereby enhancing C_2+_ production.^291^ Alteration of the size of the nanoconfined region can influence the reaction kinetics by causing strain as a result of changes in curvature and coordination number,^292^ thus impacting the binding strength of reactants. Of note is that nanoconfinement is most effective when the reaction is limited by electron-transfer kinetics rather than mass transport. Alternatively, the effectiveness of nanoconfinement can be influenced by the adsorption affinity of reactants or intermediates. Reactants that bind weakly tend to collide more frequently with active sites within confined regions, resulting in a more significant nanoconfinement effect compared to those that bind strongly. In addition to surface coverage of ∗CO intermediate, the configuration of adsorbed ∗CO can be tuned to improve selectivity toward C_2+_ alcohols through nanoconfinement.^286^ Ordered mesoporous CuO was found to favor bridged adsorption of ∗CO, while cylindrical CuO preferred a top adsorption configuration. Bridge-adsorbed ∗CO species were inclined to undergo deep protonation to form ∗OCH_3_, which promoted its further coupling with ∗CO to yield C_2_H_5_OH and C_2_H_5_CH_2_OH. This contrasted with top adsorbed ∗CO, which tended to couple with the formed ∗COH to generate C_2_H_4_ (Figure 7C).

To understand nanoconfinement’s impact, designing well-defined nanoconfined systems with controlled size, distribution, and hierarchy of pores, cavities, and channels as well as degree of freedom is crucial. This, in turn, can guide the development of high-performing ECR electrocatalysts toward oxygenated liquid compounds. A Cu catalyst with a wrinkle structure, tunable in shape and dimension (in terms of shrinkage degree [i.e., areal strain, *ϵ*] and Cu film thickness [*h*_Cu_]) was fabricated and investigated for ECR.^287^ Increasing *ϵ* enabled variation of the wrinkle from an open (non-folded, *ϵ* < ∼0.6) to a confined (folded, *ϵ* > ∼0.6) structure. Manipulation of *h*_Cu_ and *ϵ* enabled tuning of the dimensions of the wrinkles (i.e., wavelength and depth). A pronounced increase in C_2_H_4_ FE was observed with the increase of confinement degree for the non-folded shape (i.e., *ϵ* from 0.25 to 0.50), while there was no such change for CH_4_ FE and C_2_H_5_OH FE. As the wrinkle transformed from a non-folded shape to a folded structure (with *ϵ* elevated from 0.50 to 0.75), the C_2_H_5_OH FE increased markedly from 14.3% to 27% at 1.3 V (vs. RHE) in contrast to a notable decrease in CH_4_ FE from 21.0% to 8.0% and an unchanged C_2_H_4_ FE. The C−C coupling was enhanced in more strained wrinkles, maximizing in folded wrinkles regardless of *h*_Cu_. The folded wrinkle geometry limited the mass transfer of C_1_ intermediates and OH^−^ ions, especially favoring C_2_H_5_OH generation (Figure 7D). The C_2_H_5_OH FE was further improved to 41.9% on a folded wrinkle using 0.1 M KClO_4_ solutions as a catholyte (without buffering ability, thus leading to high local pH values).

#### Coordination engineering

C-supported SACs mostly possess a quasi-planar D_*4h*_ local symmetry with a MN_4_ coordination structure, which limits modulation of the metal sites’ electronic configuration.^293^^,^^294^ By disrupting the quasi-planar D_*4h*_ local symmetry through coordination engineering, the electrons in specific *d*/*s*/*p* orbitals of the metal sites can be redistributed across energy levels, thereby maximizing the modulation of their hybrid interactions. Consequently, this manipulation of local symmetry can enhance the adsorption and activation of CO_2_ molecules and reactive intermediates, resulting in high activity and selectivity for the ECR reaction.^295^ For example, at a potential of −0.73 V (vs. RHE), the locally planar-symmetry-broken CuN_3_ (PSB-CuN_3_) SAC achieves a formate FE of 94.3%, surpassing the planar-symmetry CuN_4_ (PS-CuN_4_) catalyst (72.4% at −0.93 V vs. RHE) (Figure 7E). The overlap between the Cu-*d* orbitals and O-*p* orbitals in the PSB-CuN_3_ catalyst is significantly greater than that between the Cu-*d* and C-*p* orbitals, making the PSB-CuN_3_ more inclined to bind with ∗OCO. Additionally, the overlap between the Cu-*d* orbitals and O-*p* orbitals in the PSB-CuN_3_ catalyst is much higher than that in the PS-CuN_4_, indicating that adjusting the local coordination structure can effectively modulate the binding capability between the catalyst and intermediates.^288^ SACs tend to generate C_1_ products due to their single active site. Despite this being the case, construction of a 2D MOF with Cu-N_1_O_3_ asymmetric units was shown to enable re-distribution of local electron structure, leading to atop- and bridge-type ∗CO adsorption on Cu and Cu–N sites, respectively.^296^ The atop-type ∗CO species on Cu sites was protonated to form ∗COH/∗CHO intermediates, which could couple with bridge-type ∗CO on Cu–N sites to generate C_2_H_4_/C_2_H_5_OH.

#### Synergy with supports

An appropriate support can enhance dispersion and mechanical stability of catalyst materials. Support properties contributing to CO_2_ electrocatalysis include (1) base property (e.g., MgO), (2) redox property (e.g., CeO_2_ and HfO_2_), (3) oxygen storage/release capability (e.g., CeO_2_ and ZrO_2_), (4) conductivity (e.g., C), and (5) strong metal−support interaction (e.g., COFs, SiO_2_).^297^^,^^298^^,^^299^ In particular, the interaction between support and active phases can alter the electronic structure of the catalyst and/or add interfacial sites, affecting the catalyst performance. For example, electronically asymmetric Cu-Cu/Cu-N-C (Cu/CuNC) interface sites anchored on three-dimensional honeycomb-like porous C were demonstrated to enhance the adsorption of ∗CO intermediates and lower the reaction barrier of C−C coupling in ECR.^300^ This composite boosted electrocatalytic CO_2_-to-C_2_H_5_OH conversion with a C_2_H_5_OH FE of 55% at a relatively positive applied potential of −0.35 V (vs. RHE).

### Interfacial electrolyte tuning

#### Cation impacts

The FE for C_2_ products derived from CO_2_ reduction demonstrates an unexpected dependence on cation size, with the efficiency increasing in the order of Li^+^ < Na^+^ < K^+^ < Rb^+^ < Cs^+^.^301^^,^^302^^,^^303^ There are four main hypotheses to explain how metal cations at the interface alter the activity and selectivity during CO_2_ electrolysis: (1) cation accumulation at the outer Helmholtz plane (OHP) suppresses the HER^304^; (2) hydrated cations act as pH-buffering agents to adjust local pH and CO_2_ concentration^305^; (3) the electrostatic field generated by hydrated cations promotes the stabilization of adsorbed CO_2_ or other polar intermediates (i.e., ∗CO_2_, ∗CO, and ∗OCCO) with large dipole moments according to a ﬁeld-dipole theory, while not affecting the HER due to the absence of a dipole in ∗H^306^; (4) metal cations may stabilize CO_2_ by forming complexes, thus facilitating the formation of short-lived ∗CO_2_^−^ and subsequent protonation to ∗COOH.^307^

**Figure 8:**
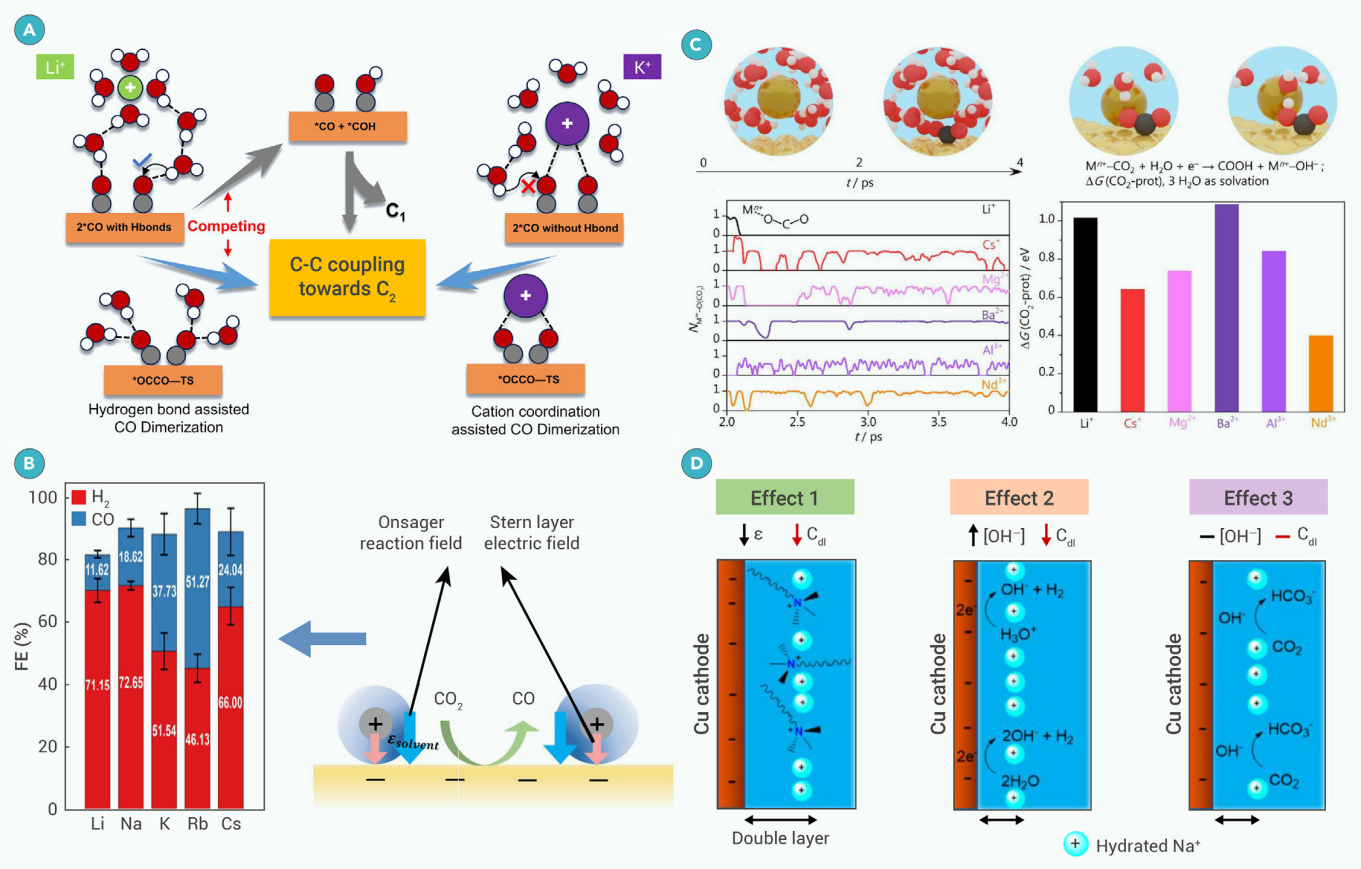
Interfacial electrolyte tuning strategie for ECR to liquid products (A) Schematic representation of the integrated mechanism elucidating the cation-dependent C_2_ selectivity through the combination effects of interfacial water molecules and cations.^310^ Copyright 2024, American Chemical Society. (B) Schematic diagram of the Onsager field resulting from the solvation shell of cations.^311^ Copyright 2022, The Authors. Published by American Chemical Society. (C) Models for *Ab initio* molecular dynamics (AIMD) simulation performed at 300 K: equilibration of Au/water/M^*n+*^ systems (0−2 ps); equilibration of Au/water/M^*n+*^/∗CO_2_ (2−4 ps), with M^*n+*^ = Li^+^, Cs^+^, Mg^2+^, Ba^2+^, Al^3+^, and Nd^3+^.^312^ Copyright 2021, The Authors. Published by American Chemical Society. (D) Proposed mechanism for changes in capacitive behavior.^313^ Copyright 2019, American Chemical Society.

Larger cations accumulate more at the surface/electrolyte interface, resulting in a more positive potential at the OHP, as per a recent theoretical study that solved the generalized modiﬁed Poisson-Nernst-Planck equations for CO_2_ reduction.^308^ As a consequence, H_3_O^+^ coverage is lowered due to electrostatic repulsion and the competitive adsorption effect, whereas neutral CO_2_ molecules are unaffected, thus hindering mass transport of protons. Meanwhile, the adsorbed cations were demonstrated to have a negligible impact on the hydrogen adsorption energy in acid.^309^ These aspects favor ECR over HER in acidic media. A recent theoretical study has shown that, for the production of C_2_ products,^310^ larger cations can coordinate with ∗OCCO, partially replacing the hydrogen bonds with water for stability. When larger cations (e.g., K^+^) coordinate with ∗CO + ∗CO, they can repel surrounding water molecules, thereby creating a localized hydrophobic environment. This reduces the risk of ∗CO being protonated to form C_1_ products and also explains why larger cations are beneficial in promoting C−C coupling (Figure 8A).^310^

Near a negatively charged electrode, the hydration shell of a large alkali cation (with low cation-water coordination numbers and short distances) generates a stronger electrostatic field, giving rise to a smaller p*K*_a_ of hydrolysis (Table S7)^314^ and a more neutral interfacial pH, consequently increasing CO_2_ concentrations at the surface. In the absence of alkali cations, rapid neutralization of CO_2_ can occur by adjacent OH^−^ to form carbonate, resulting in a significant decrease in CO_2_ concentration and ECR activity.

An induced electrostatic ﬁeld was found to stabilize ∗CO more effectively than ∗CHO, thus lifting the energy barrier for the conversion of ∗CO to ∗CHO.^315^ Alternatively, large cations such as Cs^+^ have been suggested to stabilize the adsorbed intermediates through surface interactions but are unlikely to block the CO-binding sites.^316^ However, the Onsager field resulting from the solvation shell of cations instead of the Stern layer field was proposed to play a role in enhancing kinetics by stabilizing bent CO_2_ molecules following the trend Li^+^ < Na^+^ < K^+^ < Rb^+^ (Figure 8B).^311^

Quaternary alkyl ammonium cations (methyl_4_N^+^, ethyl_4_N^+^, propyl_4_N^+^, and butyl_4_N^+^) were claimed to affect the intermolecular interaction between surface-adsorbed CO and interfacial H_2_O.^317^ Larger cations impair this interaction, hindering hydrogenation of surface-bound CO to form C_2+_ products. However, a different trend was observed in acetonitrile electrolytes. Larger cations (TBA^+^ > TPA^+^ > TEA^+^) resulted in a less notable increase in CO FE due to the weaker Cu–CO interaction in acetonitrile than in H_2_O. Larger cations were more likely to perturb the interfacial structure of acetonitrile and tetraalkylammonium cations, contributing to boosted CO desorption.^318^ Small cations (e.g., TEA^+^) favored oxalate generation via CO_2_^−^ radical dimerization without H_2_O being involved, whereas, with the increase of cation chain length, the yield of oxalate substantially decreased, pointing to the reaction pathway toward CO with the involvement of H_2_O.

A short-range interaction between cations and CO_2_ molecules was demonstrated to take place especially for weakly hydrated cations (e.g., K^+^ and Cs^+^).^307^ This may boost CO_2_ adsorption, decrease the O−C−O angle (to ∼130°), and enhance the ﬁrst *e*^−^ transfer from the surface to form the ∗CO_2_^·−^ intermediate. For multivalent cations, ECR activity in terms of cation−CO_2_ coordination number was shown to decrease with an increase in cation acidity (deﬁned as the ratio of charge and ionic radius) at low overpotentials with weak water reduction.^319^ Only Ba^2+^ and Cs^+^ were calculated to exhibit high ECR rates at large overpotentials (Figure 8C). However, to rationalize the actual roles of cations, the interplay between the promotional impacts on both ECR and HER should be considered.

Besides the above effects, alkali cations were observed to induce Cu cathodic corrosion at potentials more negative than the onset value (e.g., −0.4 V vs. RHE when using 0.1 M KHCO_3_).^320^ The resulting surface reconstruction of Cu led to degradation of long-term selectivity and activity. Although Cu nanocubes provided better stability and ECR selectivity compared to Cu NPs, future research needs to address such alkali-cation-induced cathodic corrosion issues.

#### Anion impacts

Anions influence ECR through four possible mechanisms: (1) buffering local pH, (2) bicarbonate anions provide protons in electrochemical reactions, (3) promoting CO_2_ activation and interactions with intermediates, (4) speciﬁcally adsorbing and maybe poisoning the surface.

For bicarbonate electrolytes, instead of acting as a buffer or proton donor, HCO_3_^−^ increases the local CO_2_ concentration via rapid chemical equilibration with CO_2(aq)_ and may also serve as a CO_2_ donor.^321^ Meanwhile, HCO_3_^−^ was postulated to stabilize the adsorption of ∗OCO^−^ instead of ∗COOH, thereby promoting HCOO^−^ production.^277^ Nonetheless, high HCO_3_^−^ concentrations usually give rise to severe formation of H_2_ and CH_4_. Therefore, low concentrations of HCO_3_^−^ (e.g., 0.1 M KHCO_3_) are usually employed.

Transfer of electrons from Cl^−^ to the unoccupied orbital of CO_2_ via the formation of C−Cl bonds was reported, thereby accelerating the activation of CO_2_.^322^ Adsorbed Cl^−^ anions on Bi electrodes have also been demonstrated to facilitate chemical interactions with reduction intermediates to boost HCOO^−^ formation.^323^

Anions may be adsorbed on the surface of catalysts even at potentials below the potential of zero charge (PZC). SO_4_^2−^ and H_x_PO_4_^(3−x)−^ were observed to be adsorbed on the surface of Cu in the potential range from −0.2 to −0.7 V (vs. RHE).^324^ The adsorbed anions may poison the Cu surface and shift the onset of ECR to more negative potentials. HPO_4_^2−^ and PO_4_^3−^ were calculated to be specifically adsorbed on Cu(100) and Cu(111), respectively, therefore restraining CO adsorption.^325^

#### Local pH effects

Surface pH (or local/localized pH) can vary substantially owing to proton consumption during ECR and/or OH^−^ production from HER, affecting the conversion of CO_2_ into the relatively inert CO_3_^2−^ (which can be transported to the anode through the membrane; i.e., CO_2_ crossover), lowering the concentrations of CO_2_ (or cations) and HCO_3_^−^, thereby causing loss of ECR activity. Additionally, the HER is affected by the local pH. Increasing the surface pH was surmised to favor C_2+_ production because the rate-limiting step for the C_2+_ formation is independent on the pH (SHE scale), therefore reducing the overpotential by 59 mV per unit increase of pH.^41^ Note that a pH gradient layer can form originating from a neutralization reaction between the CO_2_ and OH^−^. Hence, the decrease in overpotential reported in prior literature may be overestimated in strongly alkaline electrolytes. The selectivity for CH_3_COO^−^ versus the other C_2_ products was found to increase in concentrated alkaline solutions. This was attributed to the reaction of a ketene intermediate (H_2_CCO) with OH^−^.^326^

The local pH can be manipulated by introducing concentrated K^+^ to enhance CO_2_ activation in strong acids. Concurrently, the HER from water reduction is largely inhibited under a proton-depleted local environment.^327^ Alternatively, the HER can be dramatically hampered under mildly acidic pH resulting from proton consumption by OH^−^ obtained from the ECR, thus eliminating CO_2_/CO_3_^2−^ homogeneous reactions.^328^

#### Electrolyte engineering with additives

The water content and structure near the surface profoundly affect the reactivity and selectivity of ECR. Adding organic molecules like *N*,*N*-dimethylformamide (DMF) to an aqueous electrolyte alters interfacial water structure, reducing HER by excluding water from the interface and intensifying DMF–water hydrogen bonds,^329^ as observed by *in situ* surface-enhanced infrared absorption spectroscopy in the attenuated total reflection mode (ATR-SEIRAS). Benzotriazole (BTA) in 0.5 M KHCO_3_ was found to facilitate the reduction of CO_2_ to CH_3_COO^−^ (with an FE ∼21% at −1.33 V vs. RHE at 0°C) on Cu-Ag clusters in stark contrast to the major product of CO in the absence of BTA.^330^ However, the role of BTA remains elusive. Ionic liquid electrolytes on copper catalysts favor CO_2_ reduction and inhibit the HER.^331^

Cationic surfactants such as alkyl-trimethylammonium bromides have also been used as electrolyte additives to induce an interfacial electric ﬁeld due to the accumulation of the alkyl-trimethylammonium head groups at the interface, which acts to limit the available protons and thwart the competing HER (Figure 8D).^313^ The hydrophobic interactions of the alkyl chain were surmised to dominate at high surfactant concentrations, causing a more compact double layer and increasing the double-layer capacitance. Increasing the chain length to induce a more hydrophobic environment, however, was observed to result in low C−C product formation but high HCOO^−^ activity.^332^

The impact of aqueous electrolytes or additives on the surface restructuring of catalysts is less explored and deserves further investigation.

#### Solid electrolytes

GDEs overcome CO_2_ diffusion limitations in aqueous systems, achieving current densities orders of magnitude higher. Although flow cells currently seem to be a better platform for ECR studies at industry-related current densities, flooding of the GDE with liquid electrolytes in the flow cell is a source of instability for the reaction system and greatly reduces the diffusion of CO_2_ to the catalyst. The liquid electrolyte also increases the overall resistance of the electrolytic cell, resulting in a higher voltage under high current densities. The cathode side of MEA cells with a solid polymer as an electrolyte does not need liquid electrolytes, which can eliminate the flooding problem of GDEs and improve the stability of the system. The application of MEA also avoids catalyst deactivation caused by impurities in the electrolyte. However, in an MEA system, the high concentration of liquid products at the catalyst-membrane interface leads to the cross-reaction of products through the membrane, which may lead to the re-oxidation of products to CO_2_ at the anode. Therefore, developing stable and efficient ECR membranes is one way to promote the industrial application of ECR technology.

**Figure 9:**
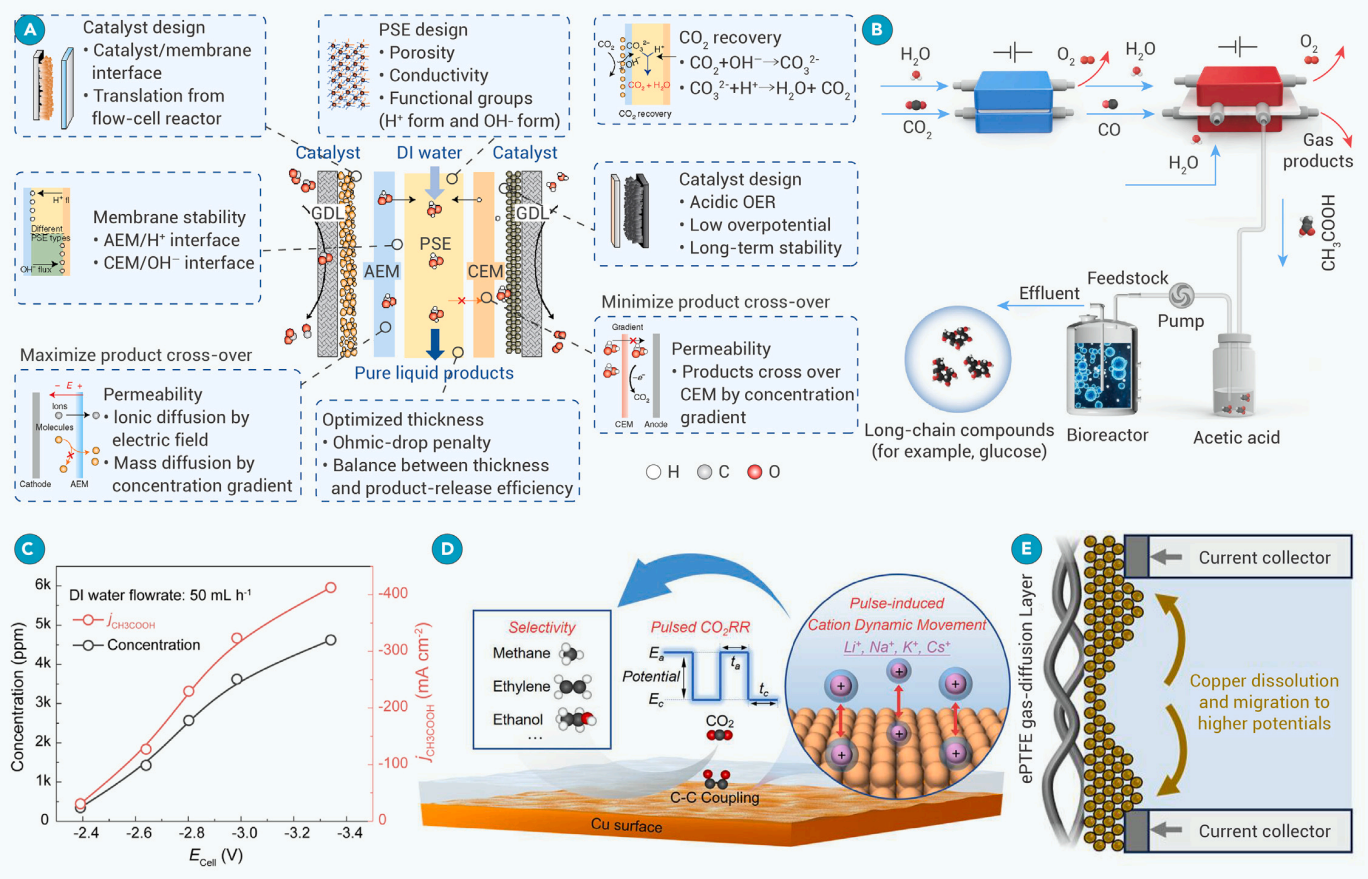
Solid electrolytes, coupled with bio-electrocatalysis and pulse-facilitated CO_2_ electrolysis sttrategies for ECR to liquid products (A) Schematic representation of an ECR electrolyzer with a porous solid electrolyte (PSE). As shown in the main cell schematic, from left to right, the components of an electrolyzer are gas-diffusion layer (GDL), cathodic catalyst, AEM, PSE (or solid-electrolyte layer), cation-exchange membrane (CEM), anodic catalyst, and GDL.^334^ Copyright 2021, Springer Nature. (B) Schematic illustration of the *in vitro* artificial sugar synthesis system. CO_2_ was first converted to pure acetic acid through two-step electrolysis; this product was then directly fed into a bioreactor for microorganism fermentation to produce long-chain compounds (for example, glucose).^75^ Copyright 2022, Springer Nature. (C) The average partial current density and concentrations of generated acetic acid under a deionized (DI) water flow rate of 50 mL h^−1^.^75^ Copyright 2022, Springer Nature. (D) Schematic of pulsed ECR.^335^ Copyright 2024, American Chemical Society. (E) Schematic illustrating directional dissolution and migration as a result of uneven potential distribution.^336^ Copyright 2024, The Authors. Published by American Chemical Society.

Inspired by solid-state batteries, SSEs have recently been used for ECR, facilitating the transfer of electrogenic cations or anions to form high-purity liquid products. Impressive production of HCOOH with a concentration of 12 M via ECR with an SSE was reported.^333^ In a 100 h stability test, the selectivity of HCOOH remained above 80%. However, the application of SSEs still faces many challenges (Figure 9A).^334^ For example, due to the absence of cations in SSEs, the cationic promotion observed in flow cells cannot be replicated directly in SSE reactors. In addition, the extra layer between the cathode and the anode contributes additional ohmic resistance, which reduces the energy efficiency of the reactor.

### CO_2_ electrolysis coupled with bio-electrocatalysis

The role of bio-electrocatalysis and the integration of electrocatalysis and bio-catalysis in producing fuels has recently been reviewed by Tan and Nielson.^337^ More complex chemicals, such as butyrate,^338^ glucose,^75^ and polyhydroxybutyrate,^339^ can be synthesized using bio-electrocatalytic systems. Biocatalyst systems include microbial electrosynthesis systems (MESs) and enzyme electrocatalysis systems (EESs), depending on the nature of the biocatalyst. MESs rely on chemolithotrophic bacteria, which are able to absorb electrons from a cathode in order to catalyze a low-potential reduction of CO_2_ into fuels or value-added chemicals.^340^^,^^341^ EESs use enzymes to replace microorganisms, showing higher selectivity and electrocatalytic efficiency than metals and MESs.^342^ However, microbial attachment characteristically has several challenges. For instance, this approach only works for a tiny subset of organisms, primarily anaerobic microorganisms that function through a reductive acetyl-coenzyme A (acetyl-CoA) pathway, leading to a narrow range of products, primarily CH_3_COO^−^ and CH_4_.^343^

In recent years, the products of microbial electrosynthesis have been expanding, especially butyric acid and caproic acid. It will be productive to combine ECR with microbial electrosynthesis to produce chemicals with higher value in the pursuit of C neutrality. A coupled electro-biosystem has been developed and utilized to upgrade CO_2_ into long-chain compounds (Figure 9B). Zheng et al.^75^ combined a nanostructured copper catalyst and a solid-electrolyte reactor to first convert CO_2_ into high-purity CH_3_COOH with a partial current density of −413 mA cm^−2^ (Figure 9C). The electrocatalytic process was followed by microbial fermentation using growing *Saccharomyces cerevisiae* to further transform CH_3_COOH into glucose *in vitro*. It is envisioned that, with the production of more high-purity chemicals such as CO, HCOOH, and CH_3_OH from ECR, a wider variety of valuable chemicals can be obtained via the engineering of bioelectrocatalysts using such a cascade hybrid system. This area is now attracting more attention in biochemistry.

### Pulse-facilitated CO_2_ electrolysis

Compared to static electrolysis, dynamically controlling the cathode potential (pulsing between two different voltages) can (1) modulate the local CO_2_ concentration, thus promoting CO_2_ mass transport; and (2) reconstruct the catalyst surface by conducting pulsed electrolysis with cathode potentials either above (anodic scan) or below (cathodic scan) the standard reduction potential of the catalyst, thereby improving the ECR and mitigating the HER.^344^ Differential electrochemical mass spectroscopy (DEMS)^345^ in tandem with GC and high-pressure liquid chromatography (HPLC) can be utilized to probe both local and bulk concentrations of ECR products and enable direct observation of product-evolution hysteresis between cathodic and anodic scans. Upon a cathodic scan, the local pH increases due to the stoichiometric generation of OH^−^, causing a slower drop in surface CO_2_ concentration. The continuous pulsing results in a transient state of high local pH and CO_2_ concentration as a result of the replenishment of the local CO_2_ concentration during the anodic scan and hence boosts the ECR selectivity. A shorter potential pulse (∼5 s) was observed to facilitate enhanced C_2+_ FEs, plausibly owing to more repeated access to the enhanced transient state.^346^ Pulsed electrolysis was observed to induce cation enrichment, consequently boosting C_2_H_5_OH and C_2_H_5_CH_2_OH production. In contrast to static condition where the C_2+_/CH_4_ ratio increased from 0.7 to 1.9 following the order Li^+^ < Na^+^ < K^+^ < Cs^+^, a much more pronounced increase in C_2+_/CH_4_ ratio from 0.1 to 8.4 was attained when using pulsed electrolysis. The induced cation enrichment was associated with the radius of hydrated ions with smaller hydrated cations being more easily enriched than the larger hydrated cations (Figure 9D).^335^

More recently, an asymmetric low-frequency pulsed strategy (ALPS) was adopted to control ECR product selectivity by manipulating the size, crystal plane, and oxidation state of Cu-based nanoclusters.^282^ This ALPS maximizes the effective ECR time and also enhances the energy utilization efficiency. Coupling of electrolyte engineering and pulsed CO_2_ electrolysis can further improve the ECR, warranting deeper investigation.

Multiple oxidation/reduction cycles could lead to the dissolution and re-deposition of Cu fragments on both sides of the GDE (Figure 9E).^336^ Combining chemical oxidation by increasing O_2_ levels (to promote the ORR to form hydroxide, an oxygen source for Cu_2_O) and electrochemical oxidation by applying anodic potentials can minimize the reconstruction process to lengthen Cu lifetimes.

### Supercritical (SC) CO_2_-assisted ECR

SC CO_2_ serves as reaction medium and reactant alike, providing high CO_2_ availability, limiting H_2_O mass transport, and accelerating ECR kinetics to yield HCOOH on Cu electrodes.^347^ Strikingly, a 4.6-fold increase in current densities was obtained in an SC CO_2_/acetonitrile (MeCN)/H_2_O system using an electrode comprising Cu NPs supported on graphite. The FE of the HER was greatly reduced from about 60% to less than 8% at elevated pressures. Additionally, a 2-fold increase in HCOOH selectivity (66% current efficiency) was observed. Another interesting aspect is that the SC CO_2_ medium provides an alternative pathway for the formation of CH_3_OH via the ECR. Employing a nonpolar SC CO_2_ mixture in place of the polar MeCN electrolyte may change the CO_2_ intermediate from ∗OCOH to ∗COOH, which is regarded as the most likely intermediate for CH_3_OH formation. Future research should focus on advanced catalysts for SC CO_2_, effective co-solvents, and enhancing electrode and membrane stability.

## Summary

Significant advances in CO_2_ conversion have been made to close the C cycle and store intermittent renewable energy. Although research efforts aiming at producing liquid chemicals or fuels by ECR have registered a lot of progress, both in terms of mechanistic studies and understanding of catalyst properties, translating laboratory results to meaningful industrial scales is still somewhat distant. Electrocatalysis is crucial to overcome ECR’s kinetic and thermodynamic barriers, which determine product selectivity and turnover rate.

**Figure 10:**
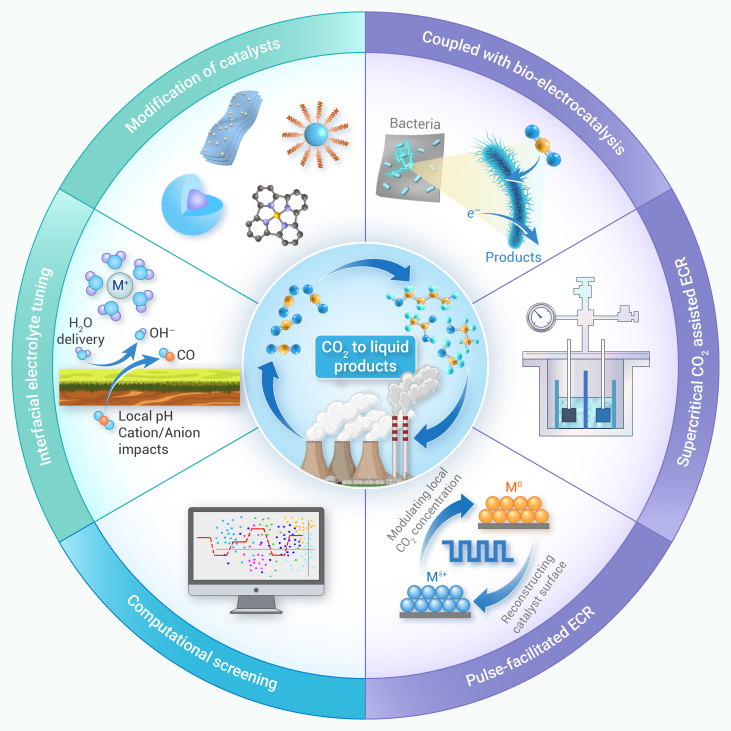
Various strategies for enhancing ECR to liquid products

We have elaborated the possible reaction pathways for different liquid products of ECR from both theoretical and experimental standpoints and introduced design strategies for advanced catalysts and catalytic systems to improve the selectivity of ECR (Figure 10). In-depth understanding of the reaction pathways and mechanisms that favor specific products for a given catalyst is necessary to inform the rational design of more effective advanced electrocatalysts. However, owing to the complex nature of ECR, which involves the transfer of up to 16 electrons via competing reaction pathways, deciphering the rate-limiting reactions for a particular product using standard Tafel slope analysis is challenging. Consequently, this information is typically acquired from coupled *in situ* and online product analysis techniques that are in themselves not without errors due to the tendency of the ECR to concurrently form multiple products.

Selectivity and mechanistic pathways for electrochemical CO_2_ conversion to formate, methanol, ethanol, acetal, acetate, ethylene glycol, 2-propanol, 1,2-propane diol, 3-hydroxybutanal, *n*-butanol, and *t*-butanol were articulated. FEs in excess of 90% have been reported for formate (94% with planar symmetry-broken CuN_3_ catalyst),^288^ methanol (Co(CO_3_)_0.5_(OH) electrocatalyst, 97%^104^; metal-free boron phosphide catalyst, 92%^111^), ethanol (B, N co-doped nanodiamond^110^), and acetate (Keggin-based electrolyte-catalyst, [SiW_9_V_3_O_40_]^−^^120^). Conversions of CO_2_ to ethanol using Ag particles anchored on graphene-wrapped N-doped C foams as heterostructured electrocatalysts and to methylglyoxal with a nickel phosphide catalyst have been demonstrated with FEs of 82%–85%^116^ and 84%,^49^ respectively. An FE of 71% for subsequent conversion of methylglyoxal to 2,3-furandiol has been obtained.^50^ *n*-Butanol was generated with an FE of 42% using Ni-incorporated (Cr_2_O_3_)_3_(Ga_2_O_3_) as a catalyst.^51^ This same catalyst achieved CO_2_ to 3-hydroxybutanal conversion with an FE of 23%. The highest *n*-propanol FE (30%)^125^ reported so far was obtained using graphene/ZnO/Cu_2_O heterostructured catalysts. Cu_x_Ir_1−x_ NPs were used to produce *t*-butanol with an FE of 15%.^53^

Computational approaches to catalyst screening to address stability, activity, and selectivity for promising ECR catalysts have been described. The use of ionic-liquid-based electrolytes for ECR is steadily increasing. Similarly, polymerized ionic liquids are being developed as solid electrolytes and offer opportunities for tuning activity via counter-ion exchange.

Electrocatalytic CO_2_ reduction to high value-added chemicals provides a feasible strategy for negating the greenhouse effect and fostering environmental protection. Although ECR technologies still face many challenges, especially in producing high-purity liquid chemicals, it is anticipated that practical CO_2_ electrolysis for clean fuels and chemical production will be realized as a result of sustained research and development of improved catalysts and optimization of the concepts of tandem catalysis, cascade electrolysis, and cell perfection.

## Outlook

Replication of the demonstrated catalytic efficacy of nanosheet-based catalysts to other important reactions is envisaged to increase steadily. Bio-electrocatalysis (microbial based) will become increasingly significant for the industrial conversion of CO_2_ to liquid fuels.^337^ ML and artificial intelligence (AI) will play increasingly important roles in catalyst, reactor, and ECR system design and optimization.

The costs of purifying CO_2_ must be decreased.^348^ Alternatively, catalysts robust to impure CO_2_ feed streams are needed. Important issues associated with controlling overall product distribution, unsatisfactory current efficiencies, and limited durability of catalyst systems must be more intensively addressed. For example, the selectivity and stability of electrocatalysts remain significant obstacles to industrializing ECR,^349^^,^^350^ especially^351^ when generating higher hydrocarbons. It is crucial to improve the selectivity and stability of liquid products produced by ECR through rational catalyst design. In particular, reaction mechanisms after the first C−C dimerization step need to be further explored. An understanding of energy dissipation and how to block side reactions is needed for tuning selectivities and lengthening performance lifetimes of catalysts.^352^^,^^353^

Coupling ECR with more valuable and thermodynamically favorable anodic oxidation reactions to substitute the OER is highly desirable. This maximizes the use of electrical energy while co-producing high-value chemicals at the anode, thus boosting the overall economic value. Three main types of reactions are intriguing: (1) oxidative upgrading of biomass-derived compounds (e.g., HMF to FDCA, HMFCA, 2,5-diformylfuran, and furfurylidenelevulinic acid; glycerol to formic acid, glycolic acid, oxalic acid, glyceraldehyde, dihydroxyacetone, glyceric acid, lactic acid, and tartronic acid; glucose to gluconic acid, glucaric acid, and formic acid); (2) upcycling of plastics including polyesters (polyethylene terephthalate and polybutylene terephthalate) and vinyl polymers (polyethylene, polystyrene, and polyvinyl chloride); and (3) electro-organic synthesis via dissociation and formation of C-, H-, O-, N-, and S-based bonds through dehydrogenation, oxygenation, and oxidative coupling, among others. Furthermore, integrating CO_2_ with other substrates such as nitrogenous small molecules, propargyl alcohols, and epoxides via electrocatalysis deserves future major efforts to produce a wider range of chemicals and fuels, facilitating the effective and sustainable utilization of CO_2_.

Gas-diffusion electrode membrane assembly cells will dominate reactor designs for the foreseeable future. Electrolyzer cell (reactor) designs will be optimized in order to support ECR commercialization.^348^ These designs include those for various 3D materials and membrane electrode cell assemblies.^337^

Facile ion diffusion will be key for at least one phase used in advanced electrodes, and MXenes may provide this important property. Improved structure-activity relationships are needed in order to better inform approaches to optimizing catalyst and system designs for reaction steps encountered in synthesizing particular liquid fuels and feedstocks.

Liquid product crossover remains a pervasive problem in ECR.^354^ Advances are needed in designing polyelectrolyte membranes that more extensively suppress liquid product crossover while facilitating ion diffusion.

The development of effective strategies to modify the electronic structure and properties of electrocatalysts in a controlled manner are needed. Variations in the band gap as a function of dimensionality (for example, modulating the band gap through control of nanosheet thicknesses) distinguish the properties and performance of many of these 2D materials from their bulk counterparts and offer potential tuning efficiencies for electron-hole separation and for intrinsic CO_2_ photo/electroreduction activity.^355^

Further advances in fabricating and controlling surface chemistry are needed to enhance ECR performance. Tailoring 2D-based nanostructures and their interfaces with specific synergies between different catalytic functionalities is especially important for maximizing catalytic activity. Heightened attention should be paid to compositions intercalated between 2D layers that are otherwise difficult to prepare via traditional synthesis protocols. These compositions are expected to afford unusual catalytic properties due to confinement effects and electrolyte sequestering properties.

Improved *in situ* characterization methods are needed for catalytic mechanistic studies and for quantifying ion transport and diffusion through multiphase heterostructures. Developing *in situ* characterization techniques will help inform theoretical models and deepen our understanding of catalytic structure-property relationships. Li and coworkers have summarized^356^ the recent progress in this area.

The importance and impact of heterostructured catalysts, tandem catalysis, and cascade reactors will steadily increase, particularly for designing multi-step fuel syntheses emanating from CO_2_. New catalysts with nanoarchitectures will be developed by manipulating 2D materials and scaffolds. For example, nanosheets can be surface decorated with catalytic NP structures to direct particularly desired reaction steps, and such surface modifications can be used to design multiple mechanistic catalytic steps.

## Acknowledgments

This work was supported by the Joint Funds of the 10.13039/501100001809National Natural Science Foundation of China (U24B20201), 10.13039/501100001809National Natural Science Foundation of China (22372007 and 21972010), and the 10.13039/501100012226Fundamental Research Funds for the Central Universities (JD2427). Y.J. acknowledges the SRC Center for Electron Transfer (2021R1A5A1030054) funded by NRF Korea and AI Graduate School Program (RS-2021-II211343). The funders had no role in study design, data collection and analysis, decision to publish, or preparation of the manuscript.

## Author contributions

Z.S. proposed the topic of the review. X.L. and W.K. wrote the draft. X.F. and Y.C. edited the figures and tables. X.T., J.M., A.W.R., J.T., Y.J., B.D., and Z.S. revised the manuscript. B.H. contributed to discussions about the review. All authors contributed to the manuscript and approved the final version.

## Declaration of interests

B.H. is an Editorial Board member of *The Innovation* and was blinded from reviewing or making final decisions on the manuscript. Peer review was handled independently of this member and their research group.

## Supplemental information

Document S1. Figure S1 and Tables S1–S7

Document S2. Article plus supplemental information
