# Supplementary material for: Electrochemical CO2 reduction to liquid fuels: Mechanistic pathways and surface/interface engineering of catalysts and electrolytes
Source: Innovation (Camb). 2025 Jan 17;6(3):100807. doi: 10.1016/j.xinn.2025.100807 (PMC11910886; doi:10.1016/j.xinn.2025.100807)
Supplement: Document S1. Figure S1 and Tables S1–S7 [file mmc1.pdf]

The Innovation, Volume *6*

Supplemental Information

Electrochemical CO_2 reduction to liquid fuels: Mechanistic pathways and surface/interface engineering of catalysts and electrolytes

Xueying Li, Woojong Kang, Xinyi Fan, Xinyi Tan, Justus Masa, Alex W. Robertson, Yousung Jung, Buxing Han, John Texter, Yuanfu Cheng, Bin Dai, and Zhenyu Sun

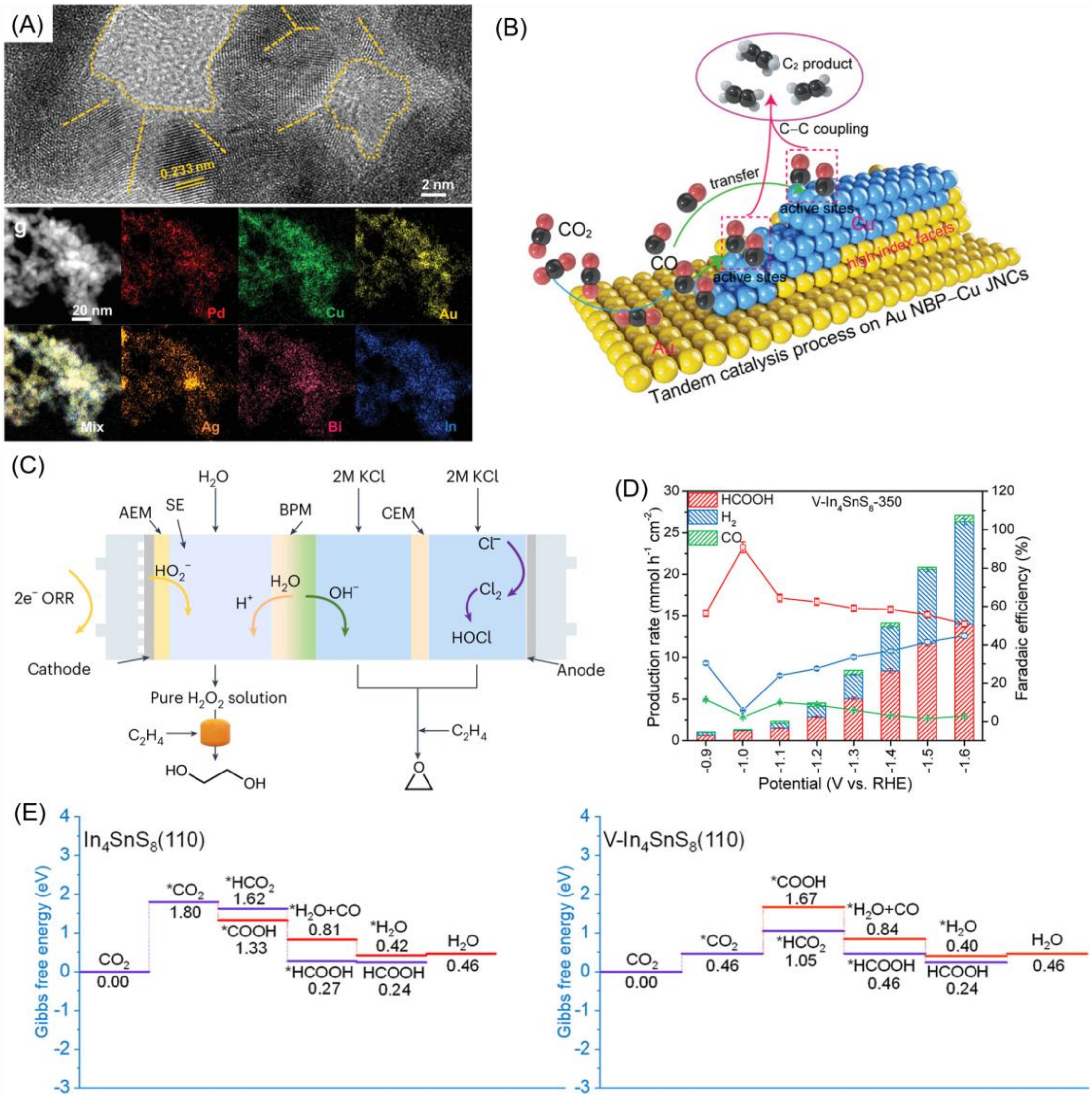

Figure S1. (A) HRTEM and STEM-EDS elemental mappings of PdCuAuAgBiIn HEAs.[1] Copyright 2023, Springer Nature. (B) Proposed mechanism for C_2 production at adjacent Au and Cu sites.[2] Copyright 2021, Wiley-VCH GmbH. (C) Schematic illustration of the three-compartment reactor for ethylene glycol production at the cathodic side and ethylene oxide production at the anodic side. BPM refers to bipolar membrane.[3] Copyright 2023, Springer Nature. (D) Electrochemical performance measurements of V-In_4SnS_8.[4] Copyright 2023, American Chemical Society. (E) Gibbs free energy diagrams of CO_2 reduction to HCOOH on pristine In_4SnS_8(110) and corresponding sulfur defective V-In_4SnS_8(110).[4] Copyright 2023, American Chemical Society.

Table S1. Various liquid products of ECR with their respective equilibrium potentials (E^0 *vs.* reversible hydrogen electrode [RHE])[5,6]

Product	Chemical reaction	E^0 /V
Formic acid ($HCOOH$)	$CO_2 + 2H^+ + 2e^- \rightarrow HCOOH$ (aq)	−0.12
Methanol (CH_3OH)	CO_2 (g) + $6H^+ + 6e^- \rightarrow CH_3OH$ (aq) + H_2O	0.03
Ethanol (C_2H_5OH)	$2CO_2$ (g) + $12H^+ + 12e^- \rightarrow C_2H_5OH$ (aq) + $3H_2O$	0.09
Acetic acid (CH_3COOH)	$2CO_2$ (g) + $8H^+ + 8e^- \rightarrow CH_3COOH$ (aq) + $2H_2O$	0.11
Acetaldehyde (CH_3CHO)	$2CO_2 + 10H^+ + 10e^- \rightarrow CH_3CHO$ (aq) + $3H_2O$	0.06
Ethylene glycol (HOC_2H_4OH)	$2CO_2 + 10H^+ + 10e^- \rightarrow HOC_2H_4OH$ (aq) + $2H_2O$	0.20
n-Propanol (*n*-C_3H_7OH)	$3CO_2 + 18H^+ + 18e^- \rightarrow$ *n*-C_3H_7OH (aq) + $5H_2O$	0.10
Propionaldehyde (C_2H_5CHO)	$3CO_2 + 16H^+ + 16e^- \rightarrow C_2H_5CHO$ (aq) + $5H_2O$	0.09
Methylglyoxal ($C_3H_4O_2$)	$3CO_2 + 12H^+ + 12e^- \rightarrow C_3H_4O_2$ (aq) + $4H_2O$	0.02
2,3-Furandiol ($C_4H_4O_3$)	$4CO_2 + 14H^+ + 14e^- \rightarrow C_4H_4O_3$ (aq) + $5H_2O$	0.01

Table S2. Summary of recent reported ECR catalysts for formic acid/formate production.

Catalyst	Electrolyte	Electrolytic cell	Applied potential (V *vs.* RHE)	Partial current density (mA cm^{-2})	Faradaic efficiency (%)	Stability (h)	Ref.
$In_{1.5}Cu_{0.5}$ NPs	0.1 M $KHCO_3$	H-type	−1.2	−4.0	90	5	[7]
BMNS	0.5 M $KHCO_3$	H-type	−0.8	−23.0	98	40	[8]
NW-SnO_2	0.5 M $KHCO_3$	H-type	−1.0	−22.0	87.4	18	[9]
Atomically isolated In (In−N−C)	0.5 M $KHCO_3$	H-type	−0.79	−6.8	80	/	[10]
Cu−MOF derived Cu	$BmimBF_4$, (0.5 M)/(MeCN)/H_2O (1.0 M)	H-type	−1.85 (V *vs.* Ag/Ag^+)	−102.1	98.2	10	[11]
Bi-Sn Aerogel	0.1 M $KHCO_3$	H-type	−1.0	−9.3	93.9	10	[12]
Bi_2S_3-derived catalyst	1.0 M KOH	Flow cell with GDE	−0.95	−2000	93	100	[13]
HCS/Cu	0.5 M $KHCO_3$	H-type	−0.81	−26	82.4	24 h	[14]
CuSn alloy @ Cu doped SnO	0.5 M $KHCO_3$	H-type	−1.2	/	95.4	10	[15]
Monolayer Bi_2WO_6	0.5 M $KHCO_3$	H-type	−1.0	−47.18	98.64	12	[16]
	1.0 M KOH	Flow cell with GDE	−1.3	−300	> 97 at −250 mA cm^{-2}	5	
Bi_2O_3 spheres	0.5 M $KHCO_3$	H-type	−0.9	−8	91	24	[17]
Bi-decorated SnO_x	0.5 M $KHCO_3$	H-type	−1.37	−45	90.8	10	[18]
$Bi_2O_2CO_3$ nanosheets	0.5 M $KHCO_3$	H-type	−1.8 (V *vs.* Ag/AgCl)	−35	92.6	8	[19]
Cu_3PdN nanocrystal	0.1 M $KHCO_3$	H-type	−1.0	/	43	17	[20]
Ch-Sn_3Se_7 derived SnO_2	0.1 M $KHCO_3$	H-type	−1.06	−11.5	94.5	100	[21]
Bimetallic Zn_3Sn_2	0.5 M $KHCO_3$	H-type	−1.1	−26	96.7	10	[22]
BiNS	0.05 M H_2SO_4 + 3 M KCl	Flow cell with GDE	−1.28	−257.1	92.2	/	[23]
MIL-	0.1 M	Flow cell	−1.1	−108	94.4	24	[24]

68(In)-NH_2	$KHCO_3$	with GDE					
Zn(Pb)-4	0.1 M $KHCO_3$	H-type	−1.2	−47	95	/	25
Bi_2O_3/BiO_2	0.5 M $KHCO_3$	Flow cell with GDE	−1.3	−111.42	98.12	/	26
	/	MEA-installed cell	−3.7 (full cell)	−220	95.4	30	
S-In_2O_3 derived In	0.5 M $KHCO_3$	H-type	−0.98	−57	93	10	27
	0.5 M $CsHCO_3$	H-type	−0.98	−84	93	/	
Cu–CTAB	0.5 M $KHCO_3$	H-type	−0.5	−2.48	82.3	10	28
Bi nanobelts	0.5 M $KHCO_3$	H-type	−0.88	−46.0 (at −1.18 V *vs.* RHE)	93.3	/	29
	1 M KOH	Flow cell with GDE	−1.47	−400	95	23	
BiOx@C	1 M $KHCO_3$	H-type	−1.7	−37.8	89.3	/	30
Oxygen vacancy-rich In_2O_3	0.1 M $KHCO_3$	H-type	−1.27	−22.1	91.2	80	31
N-Sn(S) nanosheets	0.1 M $KHCO_3$	H-type	−0.7	/	93.3	20	32
PdBi nanosheets	0.5 M $KHCO_3$	H-type	−1.0	−30.75	91.9	10	33
Ag/$Bi_2O_2CO_3$	0.5 M $KHCO_3$	H-type	−0.86	−96.6 (at −1.36 V *vs.* RHE)	98	9.7	34
BiO_{2-x} Nanosheets	0.1 M $KHCO_3$	H-type	−1.1	−12	99.1	15	35
	1 M KOH	Flow cell with GDE	−1.0	−319	91.3	/	
Bi nanotubes	0.5 M $KHCO_3$	H-type	−0.9	−31.1	97.1	65	36
Pd_3Bi intermetallic alloys	0.1 M $KHCO_3$	H-type	−0.17	−3	99	8.3	37
S-doped Bi nanosheets	0.5 M $KHCO_3$	H-type	−0.9	−25.47	96.7	35	38
	1 M KOH	Flow cell with GDE	−0.9	−182.1	97.8	/	
SnO_2/NC	0.5 M $KHCO_3$	H-type	−1.13	−18.2	87.6	20	39
$Bi_{19}Br_3S_{27}$ nanowire	1 M KOH	Flow cell with GDE	−0.95	−250	95	2.7	40
In@InO_x nanoparticle	0.5 M $KHCO_3$	H-type	−1.0	−32.6	98	25	41
InS nanorods	1 M KOH	Flow cell with GDE	−0.7	/	94.2	58	42

$Au_{0.50}NP$-in-PdNS	0.1 M $KHCO_3$	H-type	−0.6	−13.7	97.8	2.7	43
Vo-BOC-NS	0.1 M $KHCO_3$	H-type	−0.62	−286	95	12	44
Nanoporous SnTe	0.1 M $KHCO_3$	H-type	−1.0	/	93	60	45
lattice-distorted $Bi_2O_2CO_3$	1 M KOH	Flow cell with GDE	/	−800	91 at −200 mA cm^{-2}	26	46
$(BiO)_2CO_3$ Nanoflower	0.1 M $KHCO_3$	H-type	−1.0	−22.2	98.9	36	47
	1 M KOH	Flow cell with GDE	−1.2	−316	97.3	5	
$AgIn_5S_8$	0.5 M $KHCO_3$	H-type	−1.0	−30.8	91.7	12	48
	1 M KOH	Flow cell with GDE	−0.95	−560	94	/	
$Pb_3(CO_3)_2(OH)_2$ (4 wt%)	0.1 M $KHCO_3$	H-type	−1.2	−13	96.5	10	49
Sb_2Bi_6	0.5 M $KHCO_3$	H-type	−0.9	−33.7	95.8	12	50
	1 M KOH	Flow cell with GDE	−0.9	−734	95.8		
Cu-doped Bismuth	1 M KOH	Flow cell with GDE	−0.86	−660	96.1	5	51
	1 M KOH	MEA-installed cell	/	/	~80	100 at −400 mA cm^{-2}	
FTO/C	1 M KOH	Flow cell with GDE	/	/	95 at −100 mA cm^{-2}	160	52
In/N-dG	1 M KOH	Flow cell with GDE	−1.17	−1152	96	14	53
Pb-PhyA	$[Bzmim]BF_4$–MeCN–H_2O	H-type	−2.25	−28.3	92.7	5	54
$Bi@Bi_2O_3$-NDs	0.1 M $KHCO_3$	H-type	−0.9	−11.4	92.3	4	55
PVB-C	0.1 M $KHCO_3$	H-type	−1.03	−31.0	93.6	108	56
S-CuSn	0.5 M $KHCO_3$	H-type	−1.2	−54.9	91.5	30	57

Table S3. Summary of recent reported ECR catalysts for methanol production.

Catalyst	Electrolyte	Electrolytic cell	Applied potential (V *vs.* RHE)	Partial current density (mA cm^{-2})	Faradaic efficiency (%)	Stability	Ref.
CoO/CN/Ni	0.5 M $KHCO_3$	H-type	−0.7	−10.6	70.7	10	58
SA-Cu-MXene	0.1 M $KHCO_3$	H-type	−1.4	−21.3	59.1	30	59
Mo-Bi BMC	MeCN / $[Bmim]BF_4$ (0.5 M)	H-type	−0.7 (V *vs.* SHE)	−12.1	71.2	5	60
2D hierarchical Pd/SnO_2	0.1 M $NaHCO_3$	H-type	−0.24	−0.8	54.8	8	61
BP/CP	0.1 M $KHCO_3$	H-type	−0.5	/	92	18	62
$Cu_2O_{(OL-MH)}$/Ppy	0.5 M $KHCO_3$	H-type	−0.85	−0.21	93	1	63
$Co(CO_3)_{0.5}(OH)\cdot 0.11H_2O$	0.5 M $NaHCO_3$	H-type	−0.98 (V *vs.* SCE)	−0.59	97	10	64
$Cu_{1.63}Se(1/3)$	$[Bmim]PF_6$ /H_2O	H-type	−2.1 (V *vs.* Ag/Ag^+)	−41.5	77.6	25	65
Ag,S-Cu_2O/Cu	$BMImBF_4$/ H_2O	H-type	−1.18	−82.7	67.4	24	66
Pt_xZn/C	0.1 M $NaHCO_3$	H-type	−0.9	/	81.4	16 h	67
CoPc/CNT	0.1 M $KHCO_3$	H-type	−0.82	−10.6	44	1 h	68
CoPc–NH_2/CNT	0.1 M $KHCO_3$	H-type	−1.00	−10.2	32	12 h	
MOF-derived Cu@Cu_2O	0.5 M $KHCO_3$	H-type	−0.7	/	45	10 h	69
Ni-2D-O-SA-CNT	0.1 M $KHCO_3$	H-type	−0.9	−0.94 (at −0.95 V *vs.* RHE)	27	5.5 h	70
CuSAs/TCNFs	0.1 M $KHCO_3$	H-type	−0.9	−93	44	50 h	71
$Pd_{83}Cu_{17}$ aerogel	$[Bmim]BF_4$ /H_2O	H-type	−2.1 (V *vs.* Ag/ Ag^+)	−31.8	80	24 h	72
Sn/Vo-CuO	$[Bmim]BF_4$ /H_2O	H-type	−2.0 (V *vs.* Ag/ Ag^+)	−67	88.6	36 h	73
CuO NWs	1 M KOH	Flow cell with GDE	/	−29.7	66.1	/	74

Mo–Bi BMC@CNT	[Emim]BF_4/H_2O	H-type	−0.3 (V *vs.* SCE)	−5.6	81	12 h	75
Boron-doped diamond	1 M NH_3	H-type	−1.3 (V *vs.* Ag/AgCl)	/	24.3	/	76
cobalt-phthalocyanine-based CONs (iminium-CONs)	0.2 M KOH and 1.5 M KCl	Flow cell with GDE	−0.78	−91.7	54	6 h	77
CoPc/SWCNTs	0.5 M $KHCO_3$	H-type	−0.93	−8.8	53.4	10 h	78
		Flow cell with GDE	−0.9	−66.8	31.3	/	

Table S4. Summary of recent reported ECR catalysts for ethanol production.

Catalyst	Electrolytes	Electrolytic cell	Applied potential (V *vs.* RHE)	Partial current density (mA cm^{-2})	Faradaic efficiency (%)	Stability (h)	Ref.
D-Cu_2O/Cu	0.1 M KCl	H-type	−1.2	−25.1	20.5	10	[79]
ZIS NSAs/NDCC	0.5 M $KHCO_3$	H-type	−0.7	−1.3	42	4	[80]
nitrogen-doped graphene/Cu foam	0.1 M $KHCO_3$	H-type	−0.8	−22	33.1	1	[81]
Cu/Cu_2O–CV	0.1 M $KHCO_3$	H-type	−1.06	−4.2	56.6	120	[80]
Cu-PTFE-99 NN	0.1 M $KHCO_3$	H-type	−1.5	/	42.3	/	[66]
	1 M KOH	Flow cell with GDE	/	/	40.1 at −300 mA cm^{-2}	27	
Hex-2Cu-O	0.1 M $KHCO_3$	H-type	−1.2	−8.9	32.5	25	[82]
dCu_2O/Ag2.3%	1 M KOH	Flow cell with GDE	/	/	40.8 at −800 mA cm^{-2}	/	[83]
$Cu_{1.22}V_{0.19}Se$ nanotube	1 M KOH	Flow cell with GDE	−0.8	−304	68.3	14	[84]
	0.1 M $KHCO_3$	H-type	−0.8	−21.3	70.5	138	
Cu_3Sn	0.1 M $KHCO_3$	H-type	−1.0	−8.8	64	48	[85]
K–F–Cu–CO_2	1 M KOH	Flow cell with GDE	/	/	52.9 at −800 mA cm^{-2}	12	[86]
B/N-doped sp^3/sp^2 hybridized nanocarbon (BNHC)	0.1 M $KHCO_3$	H-type	−0.6	−0.4	56	16	[87]
Cu_2S_{1-x} HN	0.5 M $KHCO_3$	Flow cell with GDE	−0.3	/	73.3	6	[88]
Cu-KOH/Ethanol-CV	0.1 M $KHCO_3$	H-type	−1.4	−35.1	42.1	12	[89]
MoP-Im	3 M KOH and 3 M KCl	Flow cell with GDE	−0.5	−90.0	77.4	12	[90]

np-Cu@VO_2-5%	0.1 M $KHCO_3$	H-type	−0.62	−16.0	30.1	/	91
	1.0 M KOH	Flow cell with GDE	−0.8	−38.0	38.2		
Cu/Cu_2O aerogel	0.1 M KCl	H-type	−1.1	−32.5	41.2	/	92
	1 M KCl	Flow cell with GDE	−0.7	−72.1	45.0		
Cu/C-0.4	0.1 M $KHCO_3$	RDE	−0.7	−1.12	91	16	93
defect-site-rich Cu structure Cu-$_{DS}$	0.1 M $KHCO_3$	H-type	−1.08	/	53	/	94
	1 M KOH	Flow cell with GDE	−0.95	−100	52	/	
	/	MEA-installed cell	−3.5 (Full cell)	/	60 at −200 mA cm^{-2}	30	
$CuAl_2O_4$/CuO	1 M KOH	Flow cell with GDE	−1.0	−82	41	150	95
CuNi@C/N-npG	0.5 M $KHCO_3$	H-type	−0.78	/	84	36	96
Cu/CuNC	0.1 M $KHCO_3$	H-type	−0.35	−0.54	55	1	97
GO-VB_6-Cu	0.1 M $KHCO_3$	H-type	−0.25	−2.57	56.3	24	98
CuNi@C/N-npG	0.5 M $KHCO_3$	H-type	−0.78	/	84	36	96
$CuAl_2O_4$/CuO	1 M KOH	Flow cell with GDE	/	/	41 at −200 mA cm^{-2}	100	99
Ag-CuNPs 5 %	3 M KOH	Flow cell with GDE	−0.78	−173.7	52.6	60	100
a-CuTi@Cu	0.1 M $KHCO_3$	H-type	−0.8	/	23.96	6	101
FeTPP[Cl]/Cu	1 M $KHCO_3$	Flow cell with GDE	−0.82	−124	41	12	102
Cu GNC	0.5 M $KHCO_3$	H-type	−0.87	−10.4	70.52	/	103
Cu_xAu_y NWA	0.1 M $KHCO_3$	H-type	−0.7	−0.4	48	8	104
Cu sandwich	0.1 M $KHCO_3$	H-type	−0.3	−0.3	31	18	105
BND	0.1 M $NaHCO_3$	H-type	−1.0	/	93.2	16	106
Cu-Cu_2O-3	0.1 M KCl	H-type	−0.4	−2.94	39.2	/	107

Table S5. Summary of recent reported ECR catalysts for *n*-propanol production.

Catalysts	Electrolyte	Electrolytic cell	Applied potential (V *vs.* RHE)	Partial current density (mA cm^{-2})	Faradaic efficiency （%）	Stability (h)	Ref.
R-Cu-C	0.1M $KHCO_3$	H-type	−1.05	−8.1	17.3	/	[108]
Hex-2Cu-O	0.1M $KHCO_3$	H-type	−1.10	−1.9	17.2	25	[82]
CuSx-DSV	1M KOH	Flow cell	−0.85	−9.9	3.1	1.25	[109]
	0.1M $KHCO_3$	H-type	−1.05	−3.1	15.4	10	
Cu_2S-Cu-V	0.1M $KHCO_3$	H-type	–0.95	−2.5	8	16	[110]
Cu-on-Cu_3N	0.1M $KHCO_3$	H-type	–0.95	−1.3	6	30	[111]
Cu nanoparticles	0.1M $KHCO_3$	H-type	–0.81	−0.8	5.9	/	[112]
Activated Cu mesh	0.5M $KHCO_3$	H-type	–0.90	−1.3	13.1	6	[113]
Agglomerated Cu nanocrystals	0.1M $KHCO_3$	H-type	–0.95	−1.7	8.8	6	[114]
Cu_2O-derived	0.5M $NaHCO_3$	H-type	−0.85	−0.9	5.7	/	[115]
Oxide-derived Pd_9Cu_{91}	0.5M $KHCO_3$	H-type	−0.65	−1.15	13.7	102	[116]
Pulsed-Cu(100)	0.1M $KHCO_3$	H-type	−1.00	/	5.5	/	[117]
Cu-B	0.1M $KHCO_3$	H-type	−1.15	−0.58	7.1	/	[118]
Cu-P	0.1M $KHCO_3$	H-type	−1.15	−0.41	5.1	/	[118]
Cu nanoparticles	1M $KHCO_3$	H-type	−0.97	−13.8	4.6	20	[119]

Table S6. List of ECR catalysts suggested via computational screening for CO_2 reduction to liquid fuels. Catalytic selectivity encompasses either competition with HER or product selectivity in ECR, while stability encompasses at least one of thermodynamic, electrochemical, or thermal stability. The major screening method is noted, with ML/DFT implies screening results of ML-aided screening is validated by DFT calculations. Due to diverse material types, we followed the conventions of individual literature in presenting ECR catalyst candidates. Further structural and operational details of a catalyst are available in the corresponding literature.

ECR catalysts	Product	U_{lim} (V)	Selectivity considered	Stability considered	Method	Ref.
Pd/W	HCOOH	−0.23	yes	yes	DFT	[120]
Au/Hf	HCOOH	−0.16	yes	yes	DFT	[120]
Au/Zr	HCOOH	−0.17	yes	yes	DFT	[120]
Fe_2N_2-Gra	HCOOH	−0.23	yes	yes	DFT	[121]
Mn-Ti_2CN_2	HCOOH	−0.32	yes	yes	DFT	[10]
Fe-Ti_2CN_2	HCOOH	−0.43	yes	yes	DFT	[10]
Mn-Na_4SiMo_{12}	HCOOH	−0.48	yes	yes	DFT	[122]
Co@2D-FeS_2	HCOOH	−0.22	yes	yes	DFT	[123]
Pt@Ag	HCOOH	−0.25	yes	yes	DFT	[124]
CrPd@PC_6	HCOOH	−0.25	Yes	yes	DFT	[125]
ZnN_4@BC_6N	HCOOH	−0.28	yes	yes	DFT	[126]
Co_2-N@Gra	HCOOH	−0.34	yes	yes	DFT	[127]
W/Au	CH_3OH	−0.63	yes	yes	DFT	[128]
Pt@dv-Gr	CH_3OH	−0.27	yes	yes	DFT	[129]
Ag/Ta	CH_3OH	−0.67	no	yes	DFT	[120]
Ru@C_2N	CH_3OH	−0.56	yes	yes	DFT	[130]
Au_2B	CH_3OH	−0.29	yes	yes	DFT	[131]
Ir-Mo_2B_2	CH_3OH	−0.27	yes	yes	DFT	[132]
Sc@Cu	C_{2+}	N/A	yes	yes	DFT	[133]

Y@Cu	C_{2+}	N/A	yes	yes	DFT	133
(Sc, Ag) @Cu	C_{2+}	N/A	yes	yes	DFT	133
(Y, Ag) @Cu	C_{2+}	N/A	yes	yes	DFT	133
(Y, Fe) @Cu	C_{2+}	N/A	yes	yes	DFT	133
(Y, Ru) @Cu	C_{2+}	N/A	yes	yes	DFT	133
(Y, Cd) @Cu	C_{2+}	N/A	yes	yes	DFT	133
(Y, Au) @Cu	C_{2+}	N/A	yes	yes	DFT	133
(V, Ag) @Cu	C_{2+}	N/A	yes	yes	DFT	133
Cu_9In_4	HCOOH	−0.19	yes	yes	ML/DFT	134
CuNiZn	CH_3OH	N/A	yes	yes	ML	135
CuCoZn	CH_3OH	N/A	yes	yes	ML	135
CuCoNiZn	CH_3OH	N/A	yes	yes	ML	135
CuCoNiZnSn	CH_3OH	N/A	yes	yes	ML	136
CuNiZnSn	CH_3OH	N/A	yes	yes	ML	136
Mn-zeolite	CH_3OH	−0.5	yes	yes	ML	137
Pb-zeolite	CH_3OH	−0.87	yes	yes	ML	137
Ti@Pd	CH_3OH	−0.63	yes	no	ML/DFT	138
RhIr⊥gra	CH_3OH	−0.35	yes	yes	ML/DFT	139
RhPt⊥gra	CH_3OH	−0.25	yes	yes	ML/DFT	139
C/C on Cu	C_{2+}	N/A	yes	no	ML	140
AgCu	C_{2+}	N/A	no	no	ML	141
AuCu	C_{2+}	N/A	no	no	ML	141
AlAu	N/A	N/A	no	no	ML	142
AlNi*	N/A	N/A	no	no	ML	142
AlPd*	N/A	N/A	no	no	ML	142
AuSn	N/A	N/A	no	no	ML	142
CuAl	N/A	N/A	no	no	ML	142

CuAu	N/A	N/A	no	no	ML	[142]
CuGa	N/A	N/A	no	no	ML	[142]
CuSi	N/A	N/A	no	no	ML	[142]
CuSn	N/A	N/A	no	no	ML	[142]
PdSi*	N/A	N/A	no	no	ML	[142]
$Co_9Ga_{42}Ni7Zn_{42}$	N/A	N/A	yes	no	ML	[143]
$Ga_{83}Ni_{17}$	N/A	N/A	yes	no	ML	[143]
$Ag_{69}Cu_{31}$	N/A	N/A	yes	no	ML	[143]
$Ag_{84}Pd_{16}$	N/A	N/A	yes	no	ML	[143]
$Au_{84}Pd_{16}$	N/A	N/A	yes	no	ML	[143]
$Co\text{-}CS_3$	N/A	N/A	yes	no	ML	[144]
$Fe\text{-}C_2S_2$	N/A	N/A	yes	no	ML	[144]
$Ni\text{-}C_2NP$	N/A	N/A	yes	no	ML	[144]
$Sc\text{-}CN_3$	N/A	N/A	yes	no	ML	[144]
$Ti\text{-}C_2S_2$	N/A	N/A	yes	no	ML	[144]
$V\text{-}NP_3$	N/A	N/A	yes	no	ML	[144]
$Zr\text{-}CN_2S$	N/A	N/A	yes	no	ML	[144]
$Sc\text{-}C_2O_2$	N/A	N/A	yes	yes	ML	[144]
$Sc\text{-}CN_2O$	N/A	N/A	yes	yes	ML	[144]
$Y\text{-}CN_2O$	N/A	N/A	yes	yes	ML	[144]
P@Cu	N/A	N/A	yes	yes	ML	[145]
Ag@Cu	N/A	N/A	yes	yes	ML	[145]
Ga@Cu	N/A	N/A	yes	yes	ML	[145]
Zn@Cu	N/A	N/A	yes	yes	ML	[145]
Sn@Cu	N/A	N/A	yes	yes	ML	[145]
Ge@Cu	N/A	N/A	yes	yes	ML	[145]
In@Cu	N/A	N/A	yes	yes	ML	[145]

Si@Cu	N/A	N/A	yes	yes	ML

Table S7. p*K*a of hydrolysis of cations in the electrolyte.[146]

Cation	Cation size (pm)	pK_a in bulk electrolyte
Li^+	69	13.6
Na^+	102	14.2
K^+	138	14.5
Rb^+	149	14.6
Cs^+	170	14.7

REFERENCES

1. Qin H. G., Du Y. F., Bai Y. Y., et al. (2023). Surface-immobilized cross-linked cationic polyelectrolyte enables CO_2 reduction with metal cation-free acidic electrolyte. *Nat. Commun.* **14**:5640. DOI:10.1038/s41467-023-41396-2
2. Jia H., Yang Y., Chow T. H., et al. (2021). Symmetry-broken Au–Cu heterostructures and their tandem catalysis process in electrochemical CO_2 reduction. *Adv. Funct. Mater.* **31**:2101255. DOI:10.1002/adfm.202101255
3. Fan L., Zhao Y., Chen L., et al. (2023). Selective production of ethylene glycol at high rate via cascade catalysis. *Nat. Catal.* **6**: 585-595. DOI:10.1038/s41929-023-00977-6
4. Niu Z., Gao X., Lou S., et al. (2023). Theory-guided S-defects boost selective conversion of CO_2 to HCOOH over In_4SnS_8 nanoflowers. *ACS Catal.* **13**:2998-3006. DOI:10.1021/acscatal.2c05957
5. Nitopi S., Bertheussen E., Scott S. B., et al. (2019). Progress and perspectives of electrochemical CO_2 reduction on copper in aqueous electrolyte. *Chem. Rev.* **119**:7610-7672. DOI:10.1021/acs.chemrev.8b00705
6. Calvinho K. U. D., Alherz A. W., Yap K. M. K., et al. (2021). Surface hydrides on Fe_2P electrocatalyst reduce CO_2 at low overpotential: steering selectivity to ethylene glycol. *J. Am. Chem. Soc.* **143**:21275-21285. DOI:10.1021/jacs.1c03428
7. Wei B., Xiong Y., Zhang Z., et al. (2021). Efficient electrocatalytic reduction of CO_2 to HCOOH by bimetallic In-Cu nanoparticles with controlled growth facet. *Appl. Catal., B* **283**:119646. DOI:10.1016/j.apcatb.2020.119646
8. Li N., Yan P., Tang Y., et al. (2021). In-situ formation of ligand-stabilized bismuth nanosheets for efficient CO_2 conversion. *Appl. Catal., B* **297**:120481. DOI:10.1016/j.apcatb.2021.120481
9. Chen Z., Fan T., Zhang Y.-Q., et al. (2020). Wavy SnO_2 catalyzed simultaneous reinforcement of carbon dioxide adsorption and activation towards electrochemical conversion of CO_2 to HCOOH. *Appl. Catal., B* **261**:118243.

DOI:10.1016/j.apcatb.2019.118243

10. Lu P., Tan X., Zhao H., et al. (2021). Atomically dispersed indium sites for selective CO_2 electroreduction to formic acid. *ACS Nano* **15**:5671-5678. DOI:10.1021/acsnano.1c00858

11. Zhu Q., Yang D., Liu H., et al. (2020). Hollow metal-organic-framework-mediated in situ architecture of copper dendrites for enhanced CO_2 electroreduction. *Angew. Chem., Int. Ed.* **59**:8896-8901. DOI:10.1002/anie.202001216

12. Wu Z., Wu H., Cai W., et al. (2021). Engineering bismuth–tin interface in bimetallic aerogel with a 3D porous structure for highly selective electrocatalytic CO_2 reduction to HCOOH. *Angew. Chem., Int. Ed.* **60**:12554-12559. DOI:10.1002/anie.202102832

13. Lin L., He X., Zhang X. G., et al. (2023). A nanocomposite of bismuth clusters and $Bi_2O_2CO_3$ sheets for highly efficient electrocatalytic reduction of CO_2 to formate. *Angew. Chem., Int. Ed.* **62**:e202214959. DOI:10.1002/anie.202214959

14. Du J., Xin Y., Dong M., et al. (2021). Copper/carbon heterogenous interfaces for enhanced selective electrocatalytic reduction of CO_2 to formate. *Small* **17**:2102629. DOI:10.1002/smll.202102629

15. Zhang M., Zhang Z., Zhao Z., et al. (2021). Tunable selectivity for electrochemical CO_2 reduction by bimetallic Cu–Sn catalysts: elucidating the roles of Cu and Sn. *ACS Catal.* **11**:11103-11108. DOI:10.1021/acscatal.1c02556

16. Liu S., Wang C., Wu J., et al. (2021). Efficient CO_2 electroreduction with a monolayer Bi_2WO_6 through a metallic intermediate surface state. *ACS Catal.* **11**:12476-12484. DOI:10.1021/acscatal.1c02495

17. Deng P., Wang H., Qi R., et al. (2020). Bismuth oxides with enhanced bismuth–oxygen structure for efficient electrochemical reduction of carbon dioxide to formate. *ACS Catal.* **10**:743-750. DOI:10.1021/acscatal.9b04043

18. Yuan T., Hu Z., Zhao Y., et al. (2020). Two-dimensional amorphous SnO_x from liquid metal: mass production, phase transfer, and electrocatalytic CO_2 reduction toward formic acid. *Nano Lett.* **20**:2916-2922.

DOI:10.1021/acs.nanolett.0c00844

19. Wang Y., Wang B., Jiang W., et al. (2022). Sub-2 nm ultra-thin $Bi_2O_2CO_3$ nanosheets with abundant Bi−O structures toward formic acid electrosynthesis over a wide potential window. *Nano Res.* **15**:2919-2927. DOI:10.1007/s12274-021-3903-0

20. Jia J., Hao X., Chang Y., et al. (2021). Rational design of Cu_3PdN nanocrystals for selective electroreduction of carbon dioxide to formic acid. *J. Colloid Interface Sci.* **586**:491-497. DOI:10.1016/j.jcis.2020.10.112

21. Zhang S., Sun M., Wang K. Y., et al. (2021). Conversion of organically directed selenidostannate into porous SnO_2 exhibiting effective electrochemical reduction of CO_2 to C_1 products. *ACS Sustainable Chem. Eng.* **9**:2358-2366. DOI:10.1021/acssuschemeng.0c08634

22. Liu W., Zhang Z., Huo S., et al. (2023). Bimetallic Zn_3Sn_2 electrocatalyst derived from mixed oxides enhances formate production towards CO_2 electroreduction reaction. *Appl. Surf. Sci.* **608**:155110. DOI:10.1016/j.apsusc.2022.155110

23. Qiao Y., Lai W., Huang K., et al. (2022). Engineering the local microenvironment over Bi nanosheets for highly selective electrocatalytic conversion of CO_2 to HCOOH in strong acid. *ACS Catal.* **12**:2357-2364. DOI:10.1021/acscatal.1c05135

24. Wang Z., Zhou Y., Xia C., et al. (2021). Efficient electroconversion of carbon dioxide to formate by a reconstructed amino-functionalized indium–organic framework electrocatalyst. *Angew. Chem., Int. Ed.* **60**:19107-19112. DOI:10.1002/anie.202107523

25. Mohamed A. G. A., Zhou E., Zeng Z., et al. (2022). Asymmetric oxo-bridged ZnPb bimetallic electrocatalysis boosting CO_2-to-HCOOH reduction. *Adv. Sci.* **9**:2104138. DOI:10.1002/advs.202104138

26. Feng X., Zou H., Zheng R., et al. (2022). Bi_2O_3/BiO_2 nanoheterojunction for highly efficient electrocatalytic CO_2 reduction to formate. *Nano Lett.* **22**:1656-1664. DOI:10.1021/acs.nanolett.1c04683

27. Ma W., Xie S., Zhang X. G., et al. (2019). Promoting electrocatalytic CO_2 reduction to formate via sulfur-boosting water activation on indium surfaces. *Nat. Commun.* **10**:892. DOI:10.1038/s41467-019-08805-x

28. Tao Z., Wu Z., Wu Y., et al. (2020). Activating copper for electrocatalytic CO_2 reduction to formate via molecular interactions. *ACS Catal.* **10**:9271-9275. DOI:10.1021/acscatal.0c02237

29. Zeng G., He Y., Ma D. D., et al. (2022). Reconstruction of ultrahigh-aspect-ratio crystalline bismuth–organic hybrid nanobelts for selective electrocatalytic CO_2 reduction to formate. *Adv. Funct. Mater.* **32**:2201125. DOI:10.1002/adfm.202201125

30. Zhao X. H., Chen Q. S., Zhuo D. H., et al. (2021). Oxygen vacancies enriched Bi based catalysts for enhancing electrocatalytic CO2 reduction to formate. *Electrochim. Acta* **367**:137478. DOI:10.1016/j.electacta.2020.137478

31. Cheng Q., Huang M., Xiao L., et al. (2023). Unraveling the influence of oxygen vacancy concentration on electrocatalytic CO_2 reduction to formate over indium oxide catalysts. *ACS Catal.* **13**:4021-4029. DOI:10.1021/acscatal.2c06228

32. Cheng H., Liu S., Zhang J., et al. (2020). Surface nitrogen-injection engineering for high formation rate of CO_2 reduction to formate. *Nano Lett.* **20**:6097-6103. DOI:10.1021/acs.nanolett.0c02144

33. Xie L., Liu X., Huang F., et al. (2022). Regulating Pd-catalysis for electrocatalytic CO_2 reduction to formate via intermetallic PdBi nanosheets. *Chin. J. Catal.* **43**:1680-1686. DOI:10.1016/S1872-2067(21)63999-2

34. Guo X., Xu S.-M., Zhou H., et al. (2022). Engineering hydrogen generation sites to promote electrocatalytic CO_2 reduction to formate. *ACS Catal.* **12**:10551-10559. DOI:10.1021/acscatal.2c02548

35. Tan Z., Zhang J., Yang Y., et al. (2022). BiO_{2-x} nanosheets with surface electron localizations for efficient electrocatalytic CO_2 reduction to formate. *CCS Chem.* **5**:133-144. DOI:10.31635/ccschem.022.202202068

36. Zheng H., Wu G., Gao G., et al. (2021). The bismuth architecture assembled by nanotubes used as highly efficient electrocatalyst for CO_2 reduction to formate.

Chem. Eng. J. **421**:129606. DOI:10.1016/j.cej.2021.129606

37. Jia L., Sun M., Xu J., et al. (2021). Phase-dependent electrocatalytic CO_2 reduction on Pd_3Bi nanocrystals. *Angew. Chem., Int. Ed.* **60**:21741-21745. DOI:10.1002/anie.202109288

38. Wang M., Liu S., Chen B., et al. (2023). Co-regulation of intermediate binding and water activation in sulfur-doped bismuth nanosheets for electrocatalytic CO_2 reduction to formate. *Chem. Eng. J.* **451**:139056. DOI:10.1016/j.cej.2022.139056

39. Wu Z., Jing H., Zhao Y., et al. (2023). Grain boundary and interface interaction Co-regulation promotes SnO_2 quantum dots for efficient CO_2 reduction. *Chem. Eng. J.* **451**:138477. DOI:10.1016/j.cej.2022.138477

40. Lv L., Lu R., Zhu J., et al. (2023). Coordinating the edge defects of bismuth with sulfur for enhanced CO_2 electroreduction to formate. *Angew. Chem., Int. Ed.* **62**:e202303117. DOI:10.1002/anie.202303117

41. Yang Y., Fu J. j., Tang T., et al. (2022). Regulating surface In–O in In@InO_x core-shell nanoparticles for boosting electrocatalytic CO_2 reduction to formate. *Chin. J. Catal.* **43**:1674-1679. DOI:10.1016/S1872-2067(21)63943-8

42. Zhang Y., Lan J., Xie F., et al. (2022). Aligned InS nanorods for efficient electrocatalytic carbon dioxide reduction. *ACS Appl. Mater. Interfaces* **14**:25257-25266. DOI:10.1021/acsami.2c01152

43. Gao N., Wang F., Ding J., et al. (2022). Intercalated gold nanoparticle in 2D palladium nanosheet avoiding CO poisoning for formate production under a wide potential window. *ACS Appl. Mater. Interfaces* **14**:10344-10352. DOI:10.1021/acsami.1c23430

44. Zhang Y., Chen Y., Liu R., et al. (2023). Oxygen vacancy stabilized $Bi_2O_2CO_3$ nanosheet for CO_2 electroreduction at low overpotential enables energy efficient CO-production of formate. *InfoMat* **5**:e12375. DOI:10.1002/inf2.12375

45. Yang Q., Zhao Y., Meng L., et al. (2022). Nanoporous intermetallic SnTe enables efficient electrochemical CO_2 reduction into formate via promoting the fracture of metal–oxygen bonding. *Small* **18**:2107968.

DOI:10.1002/smll.202107968

46. Wang Z., Zu X., Li X., et al. (2022). Industrial-current-density CO_2-to-formate conversion with low overpotentials enabled by disorder-engineered metal sites. *Nano Res.* **15**:6999-7007. DOI:10.1007/s12274-022-4335-1

47. Sui P. F., Gao M. R., Liu S., et al. (2022). Carbon dioxide valorization via formate electrosynthesis in a wide potential window. *Adv. Funct. Mater.* **32**:2203794. DOI:10.1002/adfm.202203794

48. Zhang J., Fan T., Huang P., et al. (2022). Electro-reconstruction-induced strain regulation and synergism of Ag-In-S toward highly efficient CO_2 electrolysis to formate. *Adv. Funct. Mater.* **32**:2113075. DOI:10.1002/adfm.202113075

49. Huang W., Wang Y., Liu J., et al. (2022). Efficient and selective CO_2 reduction to formate on Pd-doped $Pb_3(CO_3)_2(OH)_2$: dynamic catalyst reconstruction and accelerated CO_2 protonation. *Small* **18**:2107885. DOI:10.1002/smll.202107885

50. Yang W., Si C., Zhao Y., et al. (2022). Activating inert antimony for selective CO_2 electroreduction to formate via bimetallic interactions. *Appl. Catal., B* **316**:121619. DOI:10.1016/j.apcatb.2022.121619

51. Shen H., Zhao Y., Zhang L., et al. (2023). In-situ constructuring of copper-doped bismuth catalyst for highly efficient CO_2 electrolysis to formate in ampere-level. *Adv. Energy Mater.* **13**:2202818. DOI:10.1002/aenm.202202818

52. Ko Y. J., Kim J. Y., Lee W. H., et al. (2022). Exploring dopant effects in stannic oxide nanoparticles for CO_2 electro-reduction to formate. *Nat. Commun.* **13**:2205. DOI:10.1038/s41467-022-29783-7

53. Bi J., Li P., Liu J., et al. (2023). High-rate CO_2 electrolysis to formic acid over a wide potential window: an electrocatalyst comprised of indium nanoparticles on chitosan-derived graphene. *Angew. Chem., Int. Ed.* **62**:e202307612. DOI:10.1002/anie.202307612

54. Wu H., Song J., Xie C., et al. (2018). Design of naturally derived lead phytate as an electrocatalyst for highly efficient CO_2 reduction to formic acid. *Green Chem.* **20**:4602-4606. DOI:10.1039/C8GC02457J

55. Yang S., Jiang M., Zhang W., et al. (2023). In situ structure refactoring of

bismuth nanoflowers for highly selective electrochemical reduction of CO_2 to formate. *Adv. Funct. Mater.* **33**:2301984. DOI:10.1002/adfm.202301984

56. Zhang Z., Dou H., Gao R., et al. (2022). Steering carbon hybridization state in carbon-based metal-free catalysts for selective and durable CO_2 electroreduction. *ACS Catal.* **12**:15218-15229. DOI:10.1021/acscatal.2c03055

57. Li K., Xu J., Zheng T., et al. (2022). In situ dynamic construction of a copper tin sulfide catalyst for high-performance electrochemical CO_2 conversion to formate. *ACS Catal.* **12**:9922-9932. DOI:10.1021/acscatal.2c02627

58. Wang L., Xu Y., Chen T., et al. (2021). Ternary heterostructural CoO/CN/Ni catalyst for promoted CO_2 electroreduction to methanol. *J. Catal.* **393**:83-91. DOI:10.1016/j.jcat.2020.11.012

59. Zhao Q., Zhang C., Hu R., et al. (2021). Selective etching quaternary MAX phase toward single atom copper immobilized MXene ($Ti_3C_2Cl_x$) for efficient CO_2 electroreduction to methanol. *ACS Nano* **15**:4927-4936. DOI:10.1021/acsnano.0c09755

60. Sun X., Zhu Q., Kang X., et al. (2016). Molybdenum–bismuth bimetallic chalcogenide nanosheets for highly efficient electrocatalytic reduction of carbon dioxide to methanol. *Angew. Chem., Int. Ed.* **55**:6771-6775. DOI:10.1002/anie.201603034

61. Zhang W., Qin Q., Dai L., et al. (2018). Electrochemical reduction of carbon dioxide to methanol on hierarchical Pd/SnO_2 nanosheets with abundant Pd–O–Sn interfaces. *Angew. Chem., Int. Ed.* **57**:9475-9479. DOI:10.1002/anie.201804142

62. Mou S., Wu T., Xie J., et al. (2019). Boron phosphide nanoparticles: a nonmetal catalyst for high-selectivity electrochemical reduction of CO_2 to CH_3OH. *Adv. Mater.* **31**:1903499. DOI:10.1002/adma.201903499

63. Periasamy A. P., Ravindranath R., Senthil Kumar S. M., et al. (2018). Facet- and structure-dependent catalytic activity of cuprous oxide/polypyrrole particles towards the efficient reduction of carbon dioxide to methanol. *Nanoscale* **10**:11869-11880. DOI:10.1039/C8NR02117A

64. Huang J., Hu Q., Guo X., et al. (2018). Rethinking $Co(CO_3)_{0.5}(OH)\cdot 0.11H_2O$: a new property for highly selective electrochemical reduction of carbon dioxide to methanol in aqueous solution. *Green Chem.* **20**:2967-2972. DOI:10.1039/C7GC03744A

65. Yang D., Zhu Q., Chen C., et al. (2019). Selective electroreduction of carbon dioxide to methanol on copper selenide nanocatalysts. *Nat. Commun.* **10**:677. DOI:10.1038/s41467-019-08653-9

66. Yang B., Liu K., Li H., et al. (2022). Accelerating CO_2 electroreduction to multicarbon products via synergistic electric–thermal field on copper nanoneedles. *J. Am. Chem. Soc.* **144**:3039-3049. DOI:10.1021/jacs.1c11253

67. Payra S., Shenoy S., Chakraborty C., et al. (2020). Structure-sensitive electrocatalytic reduction of CO_2 to methanol over carbon-supported intermetallic PtZn nano-alloys. *ACS Appl. Mater. Interfaces* **12**:19402-19414. DOI:10.1021/acsami.0c00521

68. Wu Y., Jiang Z., Lu X., et al. (2019). Domino electroreduction of CO_2 to methanol on a molecular catalyst. *Nature* **575**:639-642. DOI:10.1038/s41586-019-1760-8

69. Yang X., Cheng J., Yang X., et al. (2022). MOF-derived $Cu@Cu_2O$ heterogeneous electrocatalyst with moderate intermediates adsorption for highly selective reduction of CO_2 to methanol. *Chem. Eng. J.* **431**:134171. DOI:10.1016/j.cej.2021.134171

70. Liang Z., Wang J., Tang P., et al. (2022). Molecular engineering to introduce carbonyl between nickel salophen active sites to enhance electrochemical CO_2 reduction to methanol. *Appl. Catal., B* **314**:121451. DOI:10.1016/j.apcatb.2022.121451

71. Yang H., Wu Y., Li G., et al. (2019). Scalable production of efficient single-atom copper decorated carbon membranes for CO_2 electroreduction to methanol. *J. Am. Chem. Soc.* **141**:12717-12723. DOI:10.1021/jacs.9b04907

72. Lu L., Sun X., Ma J., et al. (2018). Highly efficient electroreduction of CO_2 to methanol on palladium–copper bimetallic aerogels. *Angew. Chem., Int. Ed.*

57:14149-14153. DOI:10.1002/anie.201808964

73. Guo W., Liu S., Tan X., et al. (2021). Highly efficient CO_2 electroreduction to methanol through atomically dispersed Sn ccoupled with defective CuO catalysts. *Angew. Chem., Int. Ed.* **60**:21979-21987. DOI:10.1002/anie.202108635

74. Azenha C., Mateos-Pedrero C., Alvarez-Guerra M., et al. (2020). Enhancement of the electrochemical reduction of CO_2 to methanol and suppression of H_2 evolution over CuO nanowires. *Electrochim. Acta* **363**:137207. DOI:10.1016/j.electacta.2020.137207

75. Chi C., Duan D., Zhang Z., et al. (2020). Mo–Bi bimetallic chalcogenide nanoparticles supported on CNTs for the efficient electrochemical reduction of CO_2 to methanol. Coatings.

76. Jiwanti P. K., Natsui K., Nakata K., et al. (2016). Selective production of methanol by the electrochemical reduction of CO_2 on boron-doped diamond electrodes in aqueous ammonia solution. *RSC Adv.* **6**:102214-102217. DOI:10.1039/C6RA20466J

77. Song Y., Guo P., Ma T., et al. (2023). Ultrathin, cationic covalent organic nanosheets for enhanced CO_2 electroreduction to methanol. *Adv. Mater.* **36**:2310037. DOI:10.1002/adma.202310037

78. Su J. J., Musgrave C. B., Song Y., et al. (2023). Strain enhances the activity of molecular electrocatalysts via carbon nanotube supports. *Nat. Catal.* **6**:818-828. DOI: 10.1038/s41929-023-01005-3

79. Chang F., Wei J., Liu Y., et al. (2023). Surface/interface reconstruction in-situ on Cu_2O catalysts with high exponential facets toward enhanced electrocatalysis CO_2 reduction to C_{2+} products. *Appl. Surf. Sci.* **611**:155773. DOI:10.1016/j.apsusc.2022.155773

80. Cai F., Hu X., Gou F., et al. (2023). Ultrathin $ZnIn_2S_4$ nanosheet arrays activated by nitrogen-doped carbon for electrocatalytic CO_2 reduction reaction toward ethanol. *Appl. Surf. Sci.* **611**:155696. DOI:10.1016/j.apsusc.2022.155696

81. Zang D., Gao X. J., Li L., et al. (2022). Confined interface engineering of self-

supported Cu@N-doped graphene for electrocatalytic CO_2 reduction with enhanced selectivity towards ethanol. *Nano Res.* **15**:8872-8879. DOI:10.1007/s12274-022-4698-3

82. Yang B., Chen L., Xue S., et al. (2022). Electrocatalytic CO_2 reduction to alcohols by modulating the molecular geometry and Cu coordination in bicentric copper complexes. *Nat. Commun.* **13**:5122. DOI: 10.1038/s41467-022-32740-z

83. Wang P., Yang H., Tang C., et al. (2022). Boosting electrocatalytic CO_2-to-ethanol production via asymmetric C–C coupling. *Nat. Commun.* **13**:3754. DOI:10.1038/s41467-022-31427-9

84. Sun W., Wang P., Jiang Y., et al. (2022). V-doped Cu_2Se hierarchical nanotubes enabling flow-cell CO_2 electroreduction to ethanol with high efficiency and selectivity. *Adv. Mater.* **34**:2207691. DOI:10.1002/adma.202207691

85. Shang L., Lv X., Zhong L., et al. (2022). Efficient CO_2 electroreduction to ethanol by Cu_3Sn catalyst. *Small Methods* **6**:2101334. DOI:10.1002/smtd.202101334

86. Peng C., Yang S., Luo G., et al. (2022). Surface Co-modification of halide anions and potassium cations promotes high-rate CO_2-to-ethanol electrosynthesis. *Adv. Mater.* **34**:2204476. DOI:10.1002/adma.202204476

87. Liu Y., Yang H., Fan X., et al. (2022). Promoting electrochemical reduction of CO_2 to ethanol by B/N-doped sp^3/sp^2 nanocarbon electrode. *Chin. Chem. Lett.* **33**:4691-4694. DOI:10.1016/j.cclet.2021.12.063

88. Guo C., Guo Y., Shi Y., et al. (2022). Electrocatalytic reduction of CO_2 to ethanol at close to theoretical potential via engineering abundant electron-donating $Cu^{\delta+}$ species. *Angew. Chem., Int. Ed.* **61**:e202205909. DOI:10.1002/anie.202205909

89. Fu Y., Xie Q., Wan L., et al. (2022). Ethanol assisted cyclic voltammetry treatment of copper for electrochemical CO_2 reduction to ethylene. *Mater. Today Energy* **29**:101105. DOI:10.1016/j.mtener.2022.101105

90. Esmaeilirad M., Kondori A., Shan N., et al. (2022). Efficient electrocatalytic

conversion of CO_2 to ethanol enabled by imidazolium-functionalized ionomer confined molybdenum phosphide. *Appl. Catal., B* **317**:121681. DOI:10.1016/j.apcatb.2022.121681

91. Yang Q., Liu X., Peng W., et al. (2021). Vanadium oxide integrated on hierarchically nanoporous copper for efficient electroreduction of CO_2 to ethanol. *J. Mater. Chem. A* **9**:3044-3051. DOI:10.1039/D0TA09522B

92. Kim C., Cho K. M., Park K., et al. (2021). Cu/Cu_2O interconnected porous aerogel catalyst for highly productive electrosynthesis of ethanol from CO_2. *Adv. Funct. Mater.* **31**:2102142. DOI:10.1002/adfm.202102142

93. Xu H., Rebollar D., He H., et al. (2020). Highly selective electrocatalytic CO_2 reduction to ethanol by metallic clusters dynamically formed from atomically dispersed copper. *Nat. Energy* **5**:623-632. DOI:10.1038/s41560-020-0666-x

94. Gu Z., Shen H., Chen Z., et al. (2021). Efficient electrocatalytic CO_2 reduction to C_{2+} alcohols at defect-site-rich Cu surface. *Joule* **5**:429-440. DOI:10.1016/j.joule.2020.12.011

95. Zhang T., Yuan B., Wang W., et al. (2023). Tailoring *H intermediate coverage on the $CuAl_2O_4$/CuO catalyst for enhanced electrocatalytic CO_2 reduction to ethanol. *Angew. Chem., Int. Ed.* **62**:e202302096. DOI:10.1002/anie.202302096

96. Zhang K., Wang J., Zhang W., et al. (2023). Regulated surface electronic states of CuNi nanoparticles through metal-support interaction for enhanced electrocatalytic CO_2 reduction to ethanol. *Small* **19**:2300281. DOI:10.1002/smll.202300281

97. Yang Y., Fu J., Ouyang Y., et al. (2023). In-situ constructed Cu/CuNC interfaces for low-overpotential reduction of CO_2 to ethanol. *Natl. Sci. Rev.* **10**:nwac248. DOI:10.1093/nsr/nwac248

98. Yuan J., Yang M.-P., Zhi W.-Y., et al. (2019). Efficient electrochemical reduction of CO_2 to ethanol on Cu nanoparticles decorated on N-doped graphene oxide catalysts. *J. CO_2 Util.* **33**:452-460. DOI:10.1016/j.jcou.2019.07.014

99. Zhang T., Yuan B., Wang W., et al. (2023). Tailoring *H intermediate coverage on the $CuAl_2O_4$/CuO catalyst for enhanced electrocatalytic CO_2 reduction to

ethanol. *Angewandte Chemie International Edition* **62**:e202302096. DOI:10.1002/anie.202302096

100. Cai Z., Cao N., Zhang F., et al. (2023). Hierarchical Ag-Cu interfaces promote C−C coupling in tandem CO_2 electroreduction. *Appl. Catal., B* **325**:122310. DOI:10.1016/j.apcatb.2022.122310

101. Hu F., Yang L., Jiang Y., et al. (2021). Ultrastable Cu catalyst for CO_2 electroreduction to multicarbon liquid fuels by tuning C–C coupling with CuTi subsurface. *Angew. Chem., Int. Ed.* **60**:26122-26127. DOI:10.1002/anie.202110303

102. Li F., Li Y. C., Wang Z., et al. (2020). Cooperative CO_2-to-ethanol conversion via enriched intermediates at molecule–metal catalyst interfaces. *Nat. Catal.* **3**:75-82. DOI:10.1038/s41929-019-0383-7

103. Zhang Y., Li K., Chen M., et al. (2020). Cu/Cu_2O nanoparticles supported on vertically ZIF-L-coated nitrogen-doped graphene nanosheets for electroreduction of CO_2 to ethanol. *ACS Appl. Nano Mater.* **3**:257-263. DOI:10.1021/acsanm.9b01935

104. Zhu W., Zhao K., Liu S., et al. (2019). Low-overpotential selective reduction of CO_2 to ethanol on electrodeposited Cu_xAu_y nanowire arrays. *J. Energy Chem.* **37**:176-182. DOI:10.1016/j.jechem.2019.03.030

105. Daiyan R., Saputera W. H., Zhang Q., et al. (2019). 3D heterostructured copper electrode for conversion of carbon dioxide to alcohols at low overpotentials. *Adv. Sustainable Syst.* **3**:1800064. DOI:10.1002/adsu.201800064

106. Liu Y., Zhang Y., Cheng K., et al. (2017). Selective electrochemical reduction of carbon dioxide to ethanol on a boron- and nitrogen-co-doped nanodiamond. *Angew. Chem., Int. Ed.* **56**:15607-15611. DOI:10.1002/anie.201706311

107. Zhu Q., Sun X., Yang D., et al. (2019). Carbon dioxide electroreduction to C_2 products over copper-cuprous oxide derived from electrosynthesized copper complex. *Nat. Commun.* **10**:3851. DOI:10.1038/s41467-019-11599-7

108. Tan D., Wulan B., Ma J., et al. (2023). Electrochemical-driven reconstruction for efficient reduction of carbon dioxide into alcohols. *Chem Catal.* **3**:100512.

DOI:10.1016/j.checat.2023.100512

109. Peng C., Luo G., Zhang J., et al. (2021). Double sulfur vacancies by lithium tuning enhance CO_2 electroreduction to n-propanol. *Nat. Commun.* **12**:1580. DOI:10.1038/s41467-021-21901-1

110. Zhuang T. T., Liang Z. Q., Seifitokaldani A., et al. (2018). Steering post-C–C coupling selectivity enables high efficiency electroreduction of carbon dioxide to multi-carbon alcohols. *Nat. Catal.* **1**:421-428. DOI:10.1038/s41929-018-0084-7

111. Liang Z. Q., Zhuang T. T., Seifitokaldani A., et al. (2018). Copper-on-nitride enhances the stable electrosynthesis of multi-carbon products from CO_2. *Nat. Commun.* **9**:3828. DOI:10.1038/s41467-018-06311-0

112. Kim D., Kley C. S., Li Y., et al. (2017). Copper nanoparticle ensembles for selective electroreduction of CO_2 to C_2–C_3 products. *Proc. Natl. Acad. Sci. U. S. A.* **114**:10560-10565. DOI:10.1073/pnas.1711493114

113. Rahaman M., Dutta A., Zanetti A., et al. (2017). Electrochemical reduction of CO_2 into multicarbon alcohols on activated Cu mesh catalysts: an identical location (IL) study. *ACS Catal.* **7**:7946-7956. DOI:10.1021/acscatal.7b02234

114. Ren D., Wong N. T., Handoko A. D., et al. (2016). Mechanistic insights into the enhanced activity and stability of agglomerated Cu nanocrystals for the electrochemical reduction of carbon dioxide to n-propanol. *J. Phys. Chem. Lett.* **7**:20-24. DOI:10.1021/acs.jpclett.5b02554

115. Li C. W. and Kanan M. W. (2012). CO_2 reduction at low overpotential on Cu electrodes resulting from the reduction of thick Cu_2O films. *J. Am. Chem. Soc.* **134**:7231-7234. DOI:10.1021/ja3010978

116. Rahaman M., Kiran K., Montiel I. Z., et al. (2020). Selective n-propanol formation from CO_2 over degradation-resistant activated PdCu alloy foam electrocatalysts. *Green Chem.* **22**:6497-6509. DOI:10.1039/D0GC01636E

117. Arán-Ais R. M., Scholten F., Kunze S., et al. (2020). The role of in situ generated morphological motifs and Cu^{I} species in C_{2+} product selectivity during CO_2 pulsed electroreduction. *Nat. Energy* **5**:317-325.

DOI:10.1038/s41560-020-0594-9

118. Li H., Qin X., Jiang T., et al. (2019). Changing the product selectivity for electrocatalysis of CO_2 reduction reaction on plated Cu electrodes. *ChemCatChem* **11**:6139-6146. DOI:10.1002/cctc.201901748

119. Romero Cuellar N. S., Wiesner-Fleischer K., Fleischer M., et al. (2019). Advantages of CO over CO_2 as reactant for electrochemical reduction to ethylene, ethanol and n-propanol on gas diffusion electrodes at high current densities. *Electrochim. Acta* **307**:164-175. DOI:10.1016/j.electacta.2019.03.142

120. Zhao Z. L. and Lu G. (2018). Computational screening of near-surface alloys for CO electroreduction. *ACS Catal.* **8**:3885-3894. DOI:10.1021/acscatal.7b03705

121. Meng Y., Li K., Xiao D., et al. (2020). High selective and efficient Fe_2–N_6 sites for CO_2 electroreduction: a theoretical investigation. *Int. J. Hydrogen Energy* **45**:14311-14319. DOI:10.1016/j.ijhydene.2020.03.134

122. Zhao C. C., Su X. F., Wang S., et al. (2022). Single-atom catalysts on supported silicomolybdic acid for CO electroreduction: a DFT prediction. *J. Mater. Chem. A* **10**:6178-6186. DOI:10.1039/d1ta08285j

123. Yang Y. J., Liu J., Wu D. W., et al. (2021). Two-dimensional pyrite supported transition metal for highly-efficient electrochemical CO reduction: a theoretical screening study. *Chem. Eng. J.* **424**. DOI:10.1016/j.cej.2021.130541

124. Xiong B., Yang Y. J., Liu J., et al. (2022). Electrocatalytic reduction of CO to C products over bimetal catalysts: a DFT screening study. *Fuel Process. Technol.* **233**:107315. DOI:10.1016/j.fuproc.2022.107315

125. Li C. Y., Liu X., Xu F., et al. (2022). High-throughput screening of dual-atom doped PC6 electrocatalysts for efficient CO_2 electrochemical reduction to CH_4 by breaking scaling relations. *Electrochim. Acta* **426**. DOI:10.1016/j.electacta.2022.140764

126. Xu F., Wang X. H., Liu X., et al. (2022). Computational screening of TMN based graphene-like BCN for CO electroreduction to C hydrocarbon products.

Mol. Catal. **530**:112571. DOI:10.1016/j.mcat.2022.112571

127. Wei X., Cao S., Wei S., et al. (2022). Theoretical investigation on electrocatalytic reduction of CO_2 to methanol and methane by bimetallic atoms TM_1/TM_{2-N}@Gra (TM = Fe, Co, Ni, Cu). *Appl. Surf. Sci.* **593**:153377. DOI:10.1016/j.apsusc.2022.153377

128. Back S., Kim H. and Jung Y. (2015). Selective heterogeneous CO_2 electroreduction to methanol. *ACS Catal.* **5**:965-971. DOI:10.1021/cs501600x

129. Back S., Lim J., Kim N. Y., et al. (2017). Single-atom catalysts for CO_2 electroreduction with significant activity and selectivity improvements. *Chem. Sci.* **8**:1090-1096. DOI:10.1039/c6sc03911a

130. Cui X., An W., Liu X., et al. (2018). C_2N-graphene supported single-atom catalysts for CO_2 electrochemical reduction reaction: mechanistic insight and catalyst screening. *Nanoscale* **10**:15262-15272. DOI:10.1039/c8nr04961k

131. Xiao Y., Shen C. and Hadaeghi N. (2021). Quantum mechanical screening of 2D MBenes for the electroreduction of CO_2 to C_1 hydrocarbon fuels. *J. Phys. Chem. Lett.* **12**:6370-6382. DOI:10.1021/acs.jpclett.1c01499

132. Bai X., Zhao Z. and Lu G. (2023). Breaking the scaling relationship on single-atom embedded MBene for selective CO_2 electroreduction. *J. Phys. Chem. Lett.* **14**:5172-5180. DOI:10.1021/acs.jpclett.3c00903

133. Behrendt D., Banerjee S., Clark C., et al. (2023). High-throughput computational screening of bioinspired dual-atom alloys for CO_2 activation. *J. Am. Chem. Soc.* **145**:4730-4735. DOI:10.1021/jacs.2c13253

134. Xing M., Zhang Y., Li S., et al. (2022). Prediction of carbon dioxide reduction catalyst using machine learning with a few-feature model: WLEDZ. *J. Phys.Chem. C* **126**:17025-17035. DOI:10.1021/acs.jpcc.2c02161

135. Roy D., Mandal S. C. and Pathak B. (2021). Machine learning-driven high-throughput screening of alloy-based catalysts for selective CO_2 hydrogenation to methanol. *ACS Appl. Mater. Interfaces* **13**:56151-56163. DOI:10.1021/acsami.1c16696

136. Roy D., Mandal S. C. and Pathak B. (2022). Machine learning assisted

exploration of high entropy alloy-based catalysts for selective CO_2 reduction to methanol. *J. Phys. Chem. Lett.* **13**:5991-6002. DOI:10.1021/acs.jpclett.2c00929

137. Zhu Q., Gu Y., Liang X., et al. (2022). A machine learning model to predict CO_2 reduction reactivity and products transferred from metal-zeolites. *ACS Catal.* **12**:12336-12348. DOI:10.1021/acscatal.2c03250

138. Yang Z., Gao W. and Jiang Q. (2020). A machine learning scheme for the catalytic activity of alloys with intrinsic descriptors. *J. Mater. Chem. A* **8**:17507-17515. DOI:10.1039/D0TA06203K

139. Yu L., Li F., Huang J., et al. (2023). Double-atom catalysts featuring inverse sandwich structure for CO_2 reduction reaction: a synergetic first-principles and machine learning investigation. *ACS Catal.* **13**:9616-9628. DOI:10.1021/acscatal.3c01584

140. Wu D., Zhang J., Cheng M. J., et al. (2021). Machine learning investigation of supplementary adsorbate influence on copper for enhanced electrochemical CO_2 reduction performance. *J. Phys.Chem. C* **125**:15363-15372. DOI:10.1021/acs.jpcc.1c05004

141. Gariepy Z., Chen G., Xu A., et al. (2023). Machine learning assisted binary alloy catalyst design for the electroreduction of CO_2 to C_2 products. *Energy Adv.* **2**:410-419. DOI:10.1039/D2YA00316C

142. Tran K. and Ulissi Z. W. (2018). Active learning across intermetallics to guide discovery of electrocatalysts for CO_2 reduction and H_2 evolution. *Nat. Catal.* **1**:696-703. DOI:10.1038/s41929-018-0142-1

143. Pedersen J. K., Batchelor T. A. A., Bagger A., et al. (2020). High-entropy alloys as catalysts for the CO_2 and CO reduction reactions. *ACS Catal.* **10**:2169-2176. DOI:10.1021/acscatal.9b04343

144. Chen A., Zhang X., Chen L., et al. (2020). A machine learning model on simple features for CO_2 reduction electrocatalysts. *J. Phys.Chem. C* **124**:22471-22478. DOI:10.1021/acs.jpcc.0c05964

145. Wang D., Cao R., Hao S., et al. (2023). Accelerated prediction of Cu-based single-atom alloy catalysts for CO_2 reduction by machine learning. *Green*

Energy Environ. **8**:820-830. DOI:10.1016/j.gee.2021.10.003

146. Singh M. R., Kwon Y., Lum Y., et al. (2016). Hydrolysis of electrolyte cations enhances the electrochemical reduction of CO_2 over Ag and Cu. *J. Am. Chem. Soc.* **138**:13006-13012. DOI:10.1021/jacs.6b07612
