# Supplementary material for: Electrochemical CO2 reduction to liquid fuels: Mechanistic pathways and surface/interface engineering of catalysts and electrolytes
Source: Innovation (Camb). 2025 Jan 17;6(3):100807. doi: 10.1016/j.xinn.2025.100807 (PMC11910886; doi:10.1016/j.xinn.2025.100807)
Supplement: Document S2. Article plus supplemental information [file mmc2.pdf]

OPEN ACCESS

Electrochemical CO_2 reduction to liquid fuels: Mechanistic pathways and surface/interface engineering of catalysts and electrolytes

Xueying Li,[1,10] Woojong Kang,[2,10] Xinyi Fan,[1] Xinyi Tan,[3,*] Justus Masa,[4,*] Alex W. Robertson,[5] Yousung Jung,[2,*] Buxing Han,[6] John Texter,[7,8] Yuanfu Cheng,[1] Bin Dai,[9] and Zhenyu Sun[1,*]

*Correspondence: xinyitan@bit.edu.cn (X.T.); masajustus@googlemail.com (J.M.); yousung.jung@snu.ac.kr (Y.J.); sunzy@mail.buct.edu.cn (Z.S.)

Received: August 23, 2024; Accepted: January 14, 2025; Published Online: May 5, 2025; https://doi.org/10.1016/j.xinn.2025.100807

Crown Copyright © 2025 Published by Elsevier Inc. on behalf of Youth Innovation Co., Ltd. This is an open access article under the CC BY license (http://creativecommons.org/licenses/by/4.0/).

GRAPHICAL ABSTRACT

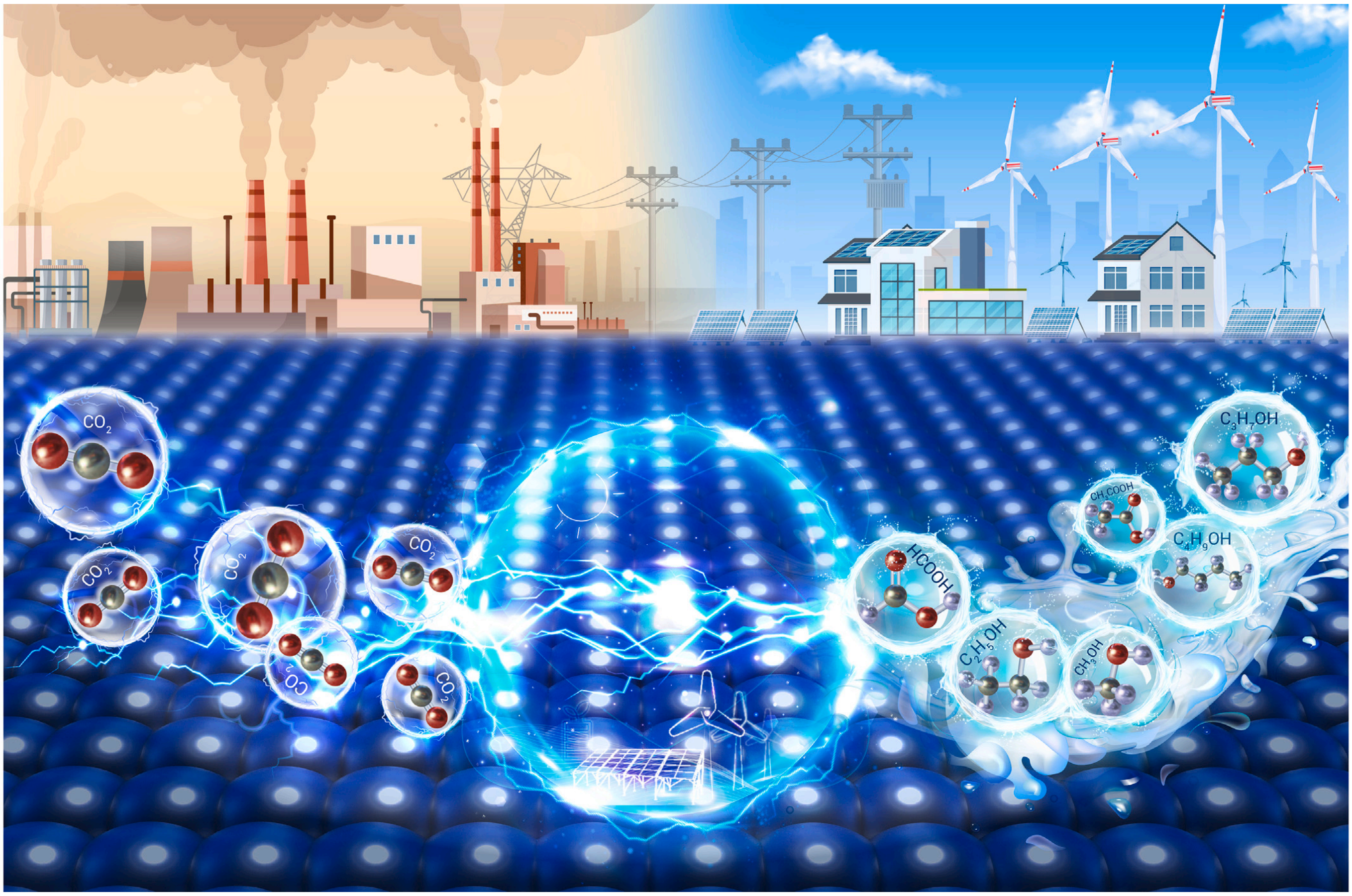

PUBLIC SUMMARY

- Electrocatalytic CO_2 reduction (ECR) into liquid chemicals can achieve a renewable carbon cycle.
- Reaction pathways for different liquid products have been assigned.
- Recent experimental and theoretical developments of ECR have been summarized.
- Design strategies for advanced ECR catalysts have been presented.

OPEN ACCESS

REVIEW

The Innovation

Electrochemical CO_2 reduction to liquid fuels: Mechanistic pathways and surface/interface engineering of catalysts and electrolytes

Xueying Li,[1,10] Woojong Kang,[2,10] Xinyi Fan,[1] Xinyi Tan,[3,*] Justus Masa,[4,*] Alex W. Robertson,[5] Yousung Jung,[2,*] Buxing Han,[6] John Texter,[7,8] Yuanfu Cheng,[1] Bin Dai,[9] and Zhenyu Sun[1,*]

[1]State Key Laboratory of Organic-Inorganic Composites, College of Chemical Engineering, Beijing University of Chemical Technology, Beijing 100029, China

[2]Department of Chemical and Biological Engineering, Institute of Chemical Processes, and Institute of Engineering Research, Seoul National University, 1 Kwanak-ro, Seoul 08826, South Korea

[3]School of Materials Science and Engineering, Beijing Institute of Technology, Beijing Key Laboratory of Environmental Science and Engineering, Beijing 100081, China

[4]Max Planck Institute for Chemical Energy Conversion, 45470 Mülheim an der Ruhr, Germany

[5]Department of Physics, University of Warwick, Coventry CV4 7AL, UK

[6]Institute of Chemistry, Chinese Academy of Sciences, Beijing 100190, China

[7]Strider Research Corporation, Rochester, NY 14610-2246, USA

[8]School of Engineering and Coating Research Institute, Eastern Michigan University, Ypsilanti, MI 48197, USA

[9]School of Chemistry and Chemical Engineering/State Key Laboratory Incubation Base for Green Processing of Chemical Engineering, Shihezi University, Shihezi 832003, China

[10]These authors contributed equally

*Correspondence: xinyitan@bit.edu.cn (X.T.); masajustus@googlemail.com (J.M.); yousung.jung@snu.ac.kr (Y.J.); sunzy@mail.buct.edu.cn (Z.S.)

Received: August 23, 2024; Accepted: January 14, 2025; Published Online: May 5, 2025; https://doi.org/10.1016/j.xinn.2025.100807

Crown Copyright © 2025 Published by Elsevier Inc. on behalf of Youth Innovation Co., Ltd. This is an open access article under the CC BY license (http://creativecommons.org/licenses/by/4.0/).

Citation: Li X., Kang W., Fan X., et al., (2025). Electrochemical CO_2 reduction to liquid fuels: Mechanistic pathways and surface/interface engineering of catalysts and electrolytes. The Innovation **6(3)**, 100807.

The high energy density of green synthetic liquid chemicals and fuels makes them ideal for sustainable energy storage and transportation applications. Electroreduction of carbon dioxide (CO_2) directly into such high value-added chemicals can help us achieve a renewable C cycle. Such electrochemical reduction typically suffers from low faradaic efficiencies (FEs) and generates a mixture of products due to the complexity of controlling the reaction selectivity. This perspective summarizes recent advances in the mechanistic understanding of CO_2 reduction reaction pathways toward liquid products and the state-of-the-art catalytic materials for conversion of CO_2 to liquid C_1 (e.g., formic acid, methanol) and C_{2+} products (e.g., acetic acid, ethanol, *n*-propanol). Many liquid fuels are being produced with FEs between 80% and 100%. We discuss the use of structure-binding energy relationships, computational screening, and machine learning to identify promising candidates for experimental validation. Finally, we classify strategies for controlling catalyst selectivity and summarize breakthroughs, prospects, and challenges in electrocatalytic CO_2 reduction to guide future developments.

INTRODUCTION

The concentration of carbon dioxide (CO_2) in the atmosphere has dramatically increased due to the steady rise in fossil fuel usage leading to severe environmental and climate impacts like melting glaciers, rising sea levels, and record-high temperatures.[1] According to statistics from the National Oceanic and Atmospheric Administration (NOAA), as of January 2024, the concentration of CO_2 in the global atmosphere has reached 422.16 ppm, an increase of 2.85 ppm compared to 2023.[2] The Intergovernmental Panel on Climate Change (IPCC) of the United Nations predicts that, by 2100, atmospheric CO_2 levels will reach 570 ppm, the global average temperature will rise by 1.9°C, and, in the worst-case scenario, sea levels will rise by at least 2 m.[3]

CO_2 capture, utilization, and storage (CCUS) technology is considered to be one of the key means to mitigate net CO_2 emissions from the energy industry.[4,5] Currently, 35 commercial CO_2 capture facilities operate worldwide, capturing nearly 45 million tonnes (Mt) of CO_2 per year. Innovative CO_2 utilization technologies make it possible to convert captured and stored CO_2 into valuable fuels or chemical feedstocks, thereby offsetting part of the operational costs of CCUS.[6] At present, the thermo-catalytic conversion of CO_2 into important chemicals and fuels has already been implemented on an industrial scale.[7,8] However, the burning of fuels during thermo-catalytic CO_2 transformation significantly affects the C footprint of these processes.[9]

In contrast, the use of electrocatalytic CO_2 reduction (ECR) is more attractive, as it can be performed under mild environmental conditions and uses renewably generated electricity to convert CO_2 into portable liquid fuels or fuel precursors.[10] However, many of the chemicals currently obtained by ECR reactions are also produced in the operationally lower-cost fossil feedstock industry, which is one of the challenges that faces the commercialization of ECR technologies, yet its promise remains realized by governments, with the US Department of Energy recently announcing increased funding to electrochemical CO_2 conversion.[11]

Great progress has been made in the development and understanding of catalytic materials for electrochemical conversion of CO_2 into different C_1–C_3 liquid products such as formic acid/formate ($HCOOH/HCOO^-$),[12] ethanol (C_2H_5OH),[13] and *n*-propanol ($C_2H_5CH_2OH$).[14] However, there remain challenges in producing liquid products through ECR, including controlling the reaction pathway to selectively generate specific C-based products, the parasitic competition with the hydrogen evolution reaction (HER) prevalent in aqueous media, and the need for product separation, which is inevitably necessary yet often overlooked. Current state-of-the-art catalysts allow the production of C_1 products with more than 95% faradaic efficiency (FE; the percentage of faradaic charge utilized to generate a specific product) and C_2 products with about 60% FE, while the FE of C_3 compounds is limited to around 10%.[15] Further issues include the large kinetic overpotentials (~1.0 V) required to induce product formation at a meaningful rate[16] and ensuring long-term catalyst stability.

Therefore, an in-depth exploration of the current state of knowledge regarding the reaction pathways for the conversion of CO_2 to liquid fuels and chemicals during ECR is timely to inform rationally designed and effective catalysts and ECR systems to solve the above challenges. This review explores CO_2 conversion pathways to liquid fuels and chemicals via ECR, summarizing the recent progress, strategies for improving catalyst performance for liquid products, and the key challenges and prospects for future development.

MECHANISTIC INSIGHTS INTO CO_2 REDUCTION

The wide product variability of ECR on different catalyst surfaces, its potential-dependent product selectivity, and structure-sensitive kinetics underscore the complexity of deciphering and controlling ECR pathways. Below, we summarize the ECR pathways for a range of liquid products.

Possible reaction pathways of ECR toward liquid products

The electrochemical reduction of CO_2 is a multi-step reaction that involves multiple electrons and protons, and it is strongly influenced by the catalyst surface properties. Key steps include diffusion of CO_2 to the active sites, CO_2 adsorption and activation, possible co-activation of reducing agents (H_2, H_2O, or cofactors), consecutive or concerted coupled transfer of electrons and protons to CO_2 forming oxygenated intermediates, and desorption of by-products and products. Initial CO_2 adsorption is often the limiting step and should thus be enhanced. Other crucial processes are H_2 activation by catalysts, CO_2 reaction with a cofactor (usually NADH) in enzymatic catalysts, and competing H_2O reduction in photocatalysis and electrocatalysis.

A particularly kinetically demanding step is the first electron transfer to CO_2, which requires a catalyst for the one-electron reduction of CO_2 to create the $CO_2^{\cdot-}$ radical anion, a requisite step for ECR toward particular products, which requires overcoming a high energy barrier to permit solvent and internal molecular reorganization.[17] Electrocatalysis is necessary to overcome kinetic barriers

and selectively reduce CO_2 to desired liquid products.[18] Proton-coupled multi-electron transfer of ECR can produce various C_1–C_3 liquid products, depending on the number of protons and electrons involved (see Table S1).

Formation of HCOOH/HCOO$^-$. HCOOH is valuable as a chemical feedstock, a hydrogen carrier, and a fuel for fuel cells. Formation of HCOOH involves the transfer of two electrons and two protons through three possible reaction pathways. In the first pathway, CO_2 receives an electron to form $*CO_2^{\cdot-}$, where the prefix "*" denotes a surface-bound species that is then protonated to form an *OCHO intermediate (green arrows in Figure 1A). Another pathway involves transferring a proton and an electron from the solution to an adsorbed species, reducing CO_2 to *COOH. A second proton then attacks the C atom in the *COOH intermediate, ultimately producing HCOOH.[19,20]

Formation of CH_3OH. CH_3OH can be directly used in fuel cells and as a feedstock for many commodity chemicals and fatty acid methyl esters (for biodiesel fuels, for example). There are three main reaction pathways for CH_3OH generation. Firstly, there is OH group abstraction from the *COOH intermediate to form an *CO intermediate (Figure 1B). The *CO intermediate is then hydrogenated to form *COH or *CHO, which subsequently undergoes further hydrogenation to form $*CH_2OH$ and then $*CH_3OH$, finally desorbing from the catalyst as CH_3OH. In another pathway, *CHO is hydrogenated to form formaldehyde (CH_2O) with the $*OCH_3$ intermediate generated through a proton-electron coupled-transfer (PECT) process following CH_2O formation. Weakly adsorbed $*CH_2O$ may undergo further electrochemical reduction steps, while CH_2O molecules desorbed from the active site may undergo a base-induced Cannizzaro reaction near the electrode surface, producing HCOOH and CH_3OH.[21]

Formation of C_2H_5OH and CH_3CHO. CH_3CHO is a critical intermediate in synthesizing pharmaceuticals, agrochemicals, and fragrances. The formation of C_2H_5OH requires the transfer of 12 electrons (Figure 1C). After the hydrogenation and dehydration of two *CO species, the formed $*C_2O$ intermediate is hydrogenated to produce $*C_2OH$ or *CHCO. C_2H_5OH is then generated through the slow hydrogenation of $*C_2OH$. Alternatively, hydrogenating *CHCO yields $*CH_xCHO$ (x = 1, 2), which is hydrogenated to C_2H_5OH.

Some studies suggest that *CO dimerization should occur before proton transfer as the first step in forming C_{2+} products.[22] Chen et al. proposed that, after C–C coupling, the oxygen atoms in *OCCO are hydrogenated to form *CO–COH.[23] The mechanism of C–C bond formation also depends on the applied potential; at low overpotentials, the C–C bonds are formed by dimerization of *CO intermediates, while, at high overpotentials, they form by the reaction of *CO and *CHO (purple arrows in Figure 1C).[24] The following *OCCO hydrodeoxygenation step determines the selectivity for ethylene or ethanol. A proton-electron transfer converts *OCCO to *OCCOH. The different atomic configurations of the active site induce the formation of two different adsorption structures of the *CCO intermediate.[25] In one of them, the *CCO intermediate undergoes a further PECT step to generate the *HCCOH intermediate (green arrows in Figure 1C), followed by the attack of *H on the C atom to form the C–C–O bond, and further hydrogenation to form *HCCHO.[26] In the other path, *H attacks the C next to the C–O bond of the *CCO intermediate to form the $*CH_3CHO$ intermediate (blue arrows in Figure 1C), which is further hydrogenated to form ethanol.

Recent studies have further investigated the influence of adsorbed *CO intermediates' orientation on C–C formation mechanisms. Shen et al. proposed an asymmetric C–C coupling mechanism on Cu-Pd alloys,[27] with density functional theory (DFT) calculations showing that *CO intermediates changed from top adsorption to bridge adsorption on the Cu-Pd surface with increasing Pd content. The *CO species on the bridge sites of Cu-Pd are more likely to be hydrogenated to form *CHO than the *CO species on the top sites of the Cu surface. These *CO species hydrogenate to *CHO, forming C_2 species by C–C coupling with *CO at adjacent Cu sites. Furthermore, Qiao's group verified by *in situ* attenuated total reflection infrared absorption spectroscopy (ATR-IRAS) characterization that the surface of Ag-modified Cu_2O ($dCu_2O/Ag_{2.3\%}$) also exhibited both top and bridge adsorption modes of *CO.[28] The *CHO intermediates were observed on the surface of $dCu_2O/Ag_{2.3\%}$, which further confirmed the asymmetric C–C coupling mechanism.

In addition to the adsorption of *CO, the role of surface adsorption of H has been investigated.[29] *HCCOH acts as a key intermediate in the C_2H_4 vs. C_2H_5OH branching pathway, where the C–O bond between the hydroxyl group and *CCH is dissociated with the help of water molecules, and *CCH is further hydrogenated to form ethylene. However, the adsorbed H only participates in the C_2H_5OH-generation pathway, and the adsorbed H attacks *HCCOH to form *HCCHOH, the key intermediate for C_2H_5OH generation. Therefore, it is beneficial to increase the surface H coverage to improve the selectivity of ECR toward C_2H_5OH.

An asymmetric $*CH_2$–CO coupling mechanism for C_2H_5OH formation uses Cu nanosheets with a Cu(111)-oriented surface (to generate $*CH_2$ intermediate) and Ag nanoparticles (NPs) (to enhance local CO concentration) (red arrows in Figure 1C).[30] Coupling of surface-adsorbed calculations shows that, for $*CH_2$ and *CO toward C_2H_5OH at low applied potential, a Langmuir-Hinshelwood (L-H) mechanism occurs, while, at more negative potential, an Eley-Rideal mechanism favors C_2H_5OH yield. This process could be promoted by an increase in local CO concentration.

Formation of CH_3COOH/CH_3COO^-. CH_3COOH has applications in the food, chemical, textile, pharmaceutical, and cosmetics sectors. There are six pathways for CH_3COOH generation by ECR. Koper et al.[31] proposed that CH_3COOH can be generated by the Cannizaro reaction of CH_3CHO, facilitated by the local alkaline environment on the electrode surface caused by the HER (Figure 1D). According to theoretical calculations, the ternary ring produced by $*OCH_2$–COH isomerization leads to acetate by-product in the ethylene (C_2H_4)-generation route (green arrows in Figure 1E).[32] The *CO intermediate can also be hydrogenated to $*CH_3$, which then couples with CO_2 to form CH_3COOH, avoiding the production of C_2H_4 and C_2H_5OH (blue arrows in Figure 1E).[33] Another CH_3COOH-generation mechanism was proposed by Luc et al.,[34] involving the hydrolysis of CH_2CO to CH_3COOH and C_2H_5OH (purple arrows in Figure 1E).

The reaction path: $CO_2 \rightarrow CO_2^{\cdot-} \rightarrow (COO)_2^{\cdot} \rightarrow CH_3COO^-$ has been discussed for the ECR on an N-doped nanodiamond/Si rod array.[35] The $CO_2^{\cdot-}$ radical may be protonated by HCO_3^- to form $HCOO^-$ and HCOOH, or the $CO_2^{\cdot-}$ combines with another anion radical to form the OOC–COO intermediate, which is further protonated and reduced to CH_3COOH. Recently, Dai et al.[36] reported a bidentate $*OC^{\cdot}O*$ pathway at Cu(I) sites (Figure 1F) under higher [CO_2 (aq)]/[HCO_3^-] conditions, where $*OC^{\cdot}O*$ coupling with $*CH_3$ formed at Cu(0) sites is favored over the protonation of $*OC^{\cdot}O*$ to form formate, resulting in a higher acetic acid FE.

Formation of HOC_2H_4OH. In the formation of ethylene glycol, the $*CO_2^{\cdot-}$ anionic radical bound on the catalyst surface generates HCOOH* through a one-electron hydrogen transfer step, with the OH group protonated to form $*H_2CO$–OH_2 and then to formaldehyde* (H_2CO*).[37] Two adjacent H_2CO* intermediates then undergo H transfer to produce HCO* and CH_2OH*. HCO* dissociates to produce CO* and H*, where CO* and CH_2OH* are C–C coupled. Meanwhile, the H* and the ketone group on the $OHCH_2$–CO* molecule combine to form glycolaldehyde ($OHCH_2$–CHO*). Eventually $OHCH_2$–CHO* hydrogenates to form ethylene glycol.

Formation of $C_2H_5CH_2OH$ and 2-propanol. $C_2H_5CH_2OH$ is a valuable C_3 alcohol that possesses a high energy density (~30.9 kJ g^{-1}) and high octane number. Its formation on copper catalysts requires at least three Cu sites to adsorb and stabilize the critical C_2 intermediates ($*C_2$) and *CO,[38] followed by a complex coupling mechanism between the C_1 and C_2 species. So far, CO–CH_2CHO[39] and CO–OCCO[40] have been proposed as two key intermediates. Hori et al.[41] reported that $C_2H_5CH_2OH$ may be reduced from propionaldehyde (C_2H_5CHO) that is formed in the reaction, with CH_3CHO a common intermediate for C_2H_5OH and C_2H_5CHO (blue arrows in Figure 2A). As the potential becomes more negative, the electrochemical reduction of CH_3CHO to C_2H_5OH becomes unfavorable relative to C–C coupling between CH_3CHO and CO, leading to C_2H_5CHO intermediate and thus promoting $C_2H_5CH_2OH$ production.

In recent years, another theory has come to the fore. CO dimers are considered to be one of the main C–C coupling pathways for *OCCO formation (green arrows in Figure 2A).[42] Since *CO is enriched at the surface during CO_2 reduction, coupling of CO and OCCO is a possible route.[43] The $*C_3$ intermediate CO–OCCO* is most widely used in theoretical calculations and is plausible not only for *n*-propanol but also for C_3 products such as acetone.[44] In addition, in the formation process of multi-C products, $*CH_2CHO$ is considered as the selective-determining intermediate.[45] This reaction pathway is more likely to generate C_2H_4 than C_2H_5OH or $C_2H_5CH_2OH$, because the energy barrier for generating $*CH_3CHO$ is 0.2 eV higher than that for generating C_2H_4. Furthermore, the formation of $C_2H_5CH_2OH$ requires $*CH_3CHO$ to undergo further C–C coupling with *CO (red arrows in Figure 2A). This requires a relatively high coverage of C_2 intermediates.

Recently, Qi et al.[46] speculated, based on DFT calculations, that 2-propanol was formed by coupling the $*OCH_2CH_3$ and *CO intermediates (Figure 2B),

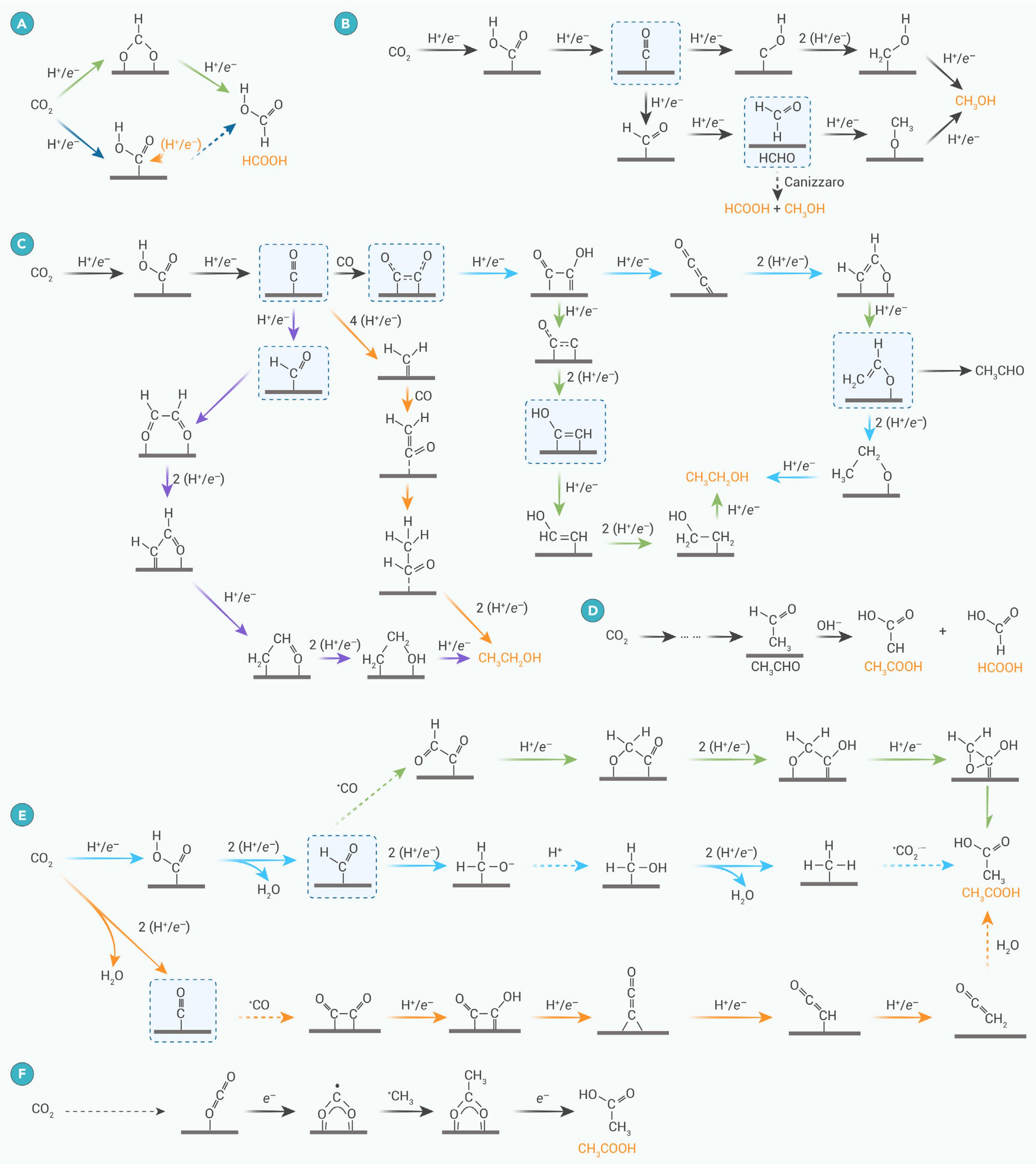

Figure 1. Possible pathways for generation of C_1 and C_2 products (A) HCOOH and (B) CH_3OH. (C) C_2H_5OH and CH_3CHO. (D–F) (D) Possible pathways for generation of CH_3COOH via Cannizaro path, (E) C—C coupling paths, and (F) radical anion path.

which was further confirmed by the addition of ethanol-d_6 to the electrolyte in experiments. The formation of 2-propanol-d_8 increased with the presence of ethanol-d_6 in the electrolyte, indicating that $*OCH_2CH_3$ is directly involved in the formation of 2-propanol.

Formation of 1,2-propanediol ($CH_2OHCHOHCH_3$). $CH_2OHCHOHCH_3$ is an important commodity chemical for fabricating pharmaceutical intermediates, degradable memory fibers, and polymers.[47] The production of $CH_2OHCHOH$ CH_3 by ECR has been theoretically predicted; however, no reasonable yields have been achieved. The reaction mechanism is still elusive. Jiao et al. carried out DFT studies and suggested that the co-adsorption energy of three *CO intermediates (ΔG_{3*CO}) could be used as a descriptor for C_3 activity. They adopted Ag-doped Cu (Ag_1Cu) single-atom alloys (SAAs) as a model, showing they can retain oxygen atoms in the hydroxyl group and further lead to a new C_3 product (1,2-propanediol [1,2-PDO]) rather than conventional *n*-propanol. Meanwhile, the reaction

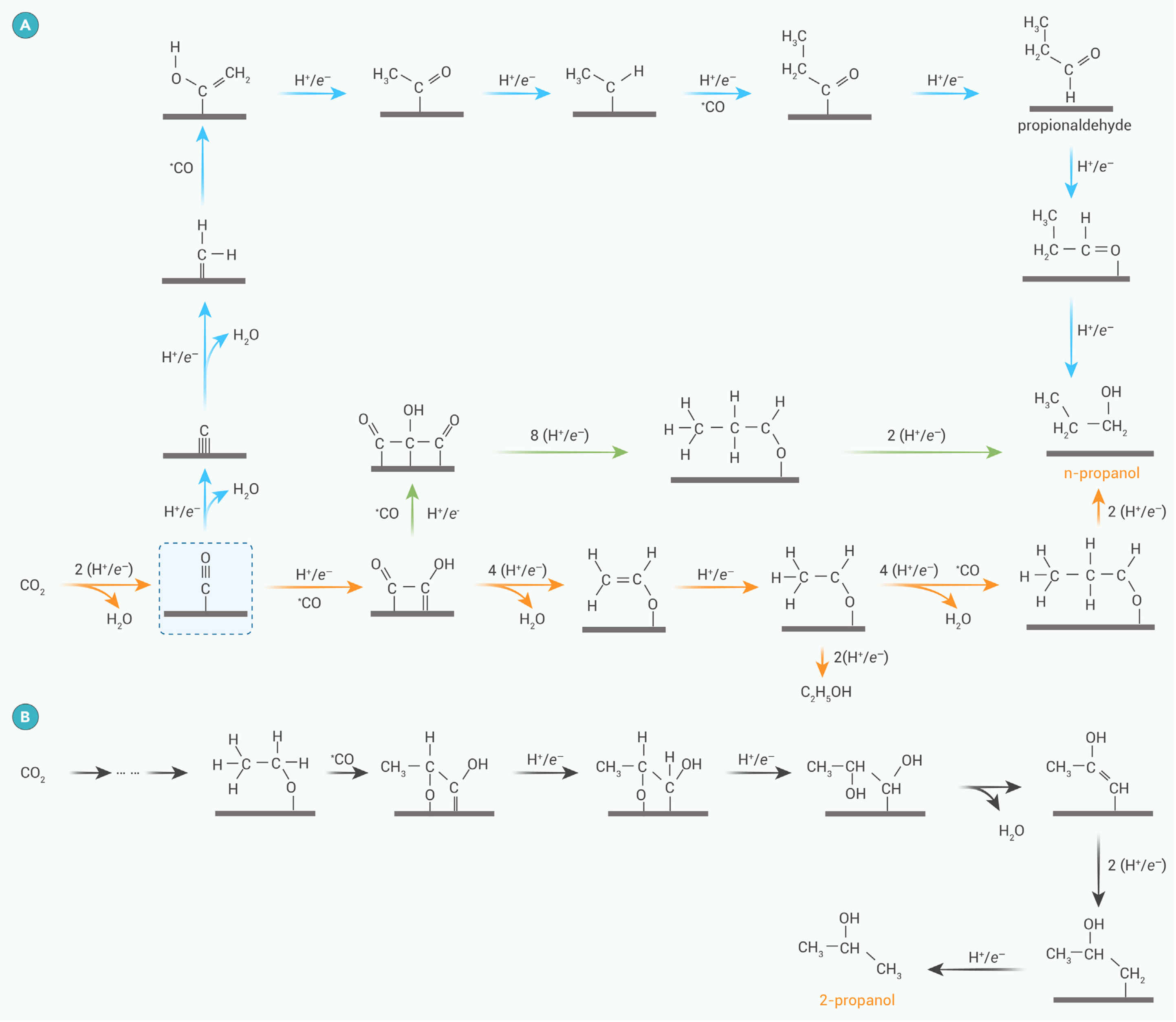

Figure 2. Possible pathways for generation of propanol (A) *n*-propanol and (B) 2-propanol.

pathway for direct protonation of C to *COH–CO–CHOH (*COH–CO–COH → *COH–CO–CHOH) is predicted to be facile because this process is exergonic (0.12 eV) and thus favorable for the subsequent steps, with the preservation of the oxygen atom in the hydroxyl group ensured by the preferential protonation of C. Hence, 1,2-PDO can be more easily formed than the other possible C_3 products.

Formation of methylglyoxal and 2,3-furanediol. In the methylglyoxal and 2,3-furandiol-generation pathways (Figure 3A), CO_2 first binds to the hydride on the catalyst surface through hydride transfer (HT) to form adsorbed $HCOO^-$, which is subsequently protonated to form adsorbed HCOOH. HCOOH is then attacked by a second hydride, forming H_2CO* and eliminating the hydroxide. The self-condensation of H_2CO*, shown by DFT calculations, involves four important steps.[48] Hydrogens transfer between two adjacent H_2CO*, resulting in *CHO and CH_2OH*. Next, dissociation of *CHO occurs to form CO* and H*. CO* and CH_2OH* further react to form $HOCH_2$–CO*. Finally, H* ligates to the ketone group of $HOCH_2$–CO*, generating the C_2 intermediate $HOCH_2$–CHO*, which then undergoes C–C coupling with H_2CO* to produce glyceraldehyde*. The OH group at the end of glyceraldehyde* is more likely to undergo water elimination to produce 2-hydroxy-2-propenal.[37] Methylglyoxal can be produced by enol-keto tautomerization.[49] Otherwise, 2-hydroxy-2-propenal can be further C–C coupled with H_2CO* to produce dihydro-2-hydroxy-furanone and finally aromatization into 2,3-furanediol.

Zheng et al.[50] also proposed a water-assisted proton transfer (WAPT) mechanism on Ni_3P_2 (Figure 3B). CO_2 is hydrogenated through direct proton transfer at the Ni_3 site to form *OCHO or *COOH, with *COOH formation favored in the presence of hydrogen-bond interactions. The WAPT process involves the separation and transfer of a surface proton to a nearby water molecule, the hopping of another proton from this hydrohydrate to a neighboring water molecule, and finally the hydrogenation of CO_2 by a proton from the hydrogen-bonding network. After two WAPT processes, the second CO_2 was hydrogenated to *CHOH and subjected to C–C coupling with *CO. Following the dehydration of *CO–CHOH to create *CO–CH, *CO was then C–C combined with *CO–CHOH to create *CO–CO–CH. It further undergoes a successive hydrogenation step to generate *CHO–CO–CH_3, which is desorbed as methylglyoxal. Alternatively, the C_3 intermediate *CO–CO–CH can be hydrogenated to *CHO–COH–CH and undergo C–C coupling with a fourth *CO to produce the C_4 species *CHO–COH–CH–CO. This is subsequently transformed to 2,3-furandiol via proton tautomerization, hydrogenation, and cyclization. Although this pathway provides an explanation for the product distribution observed on Ni_3P_2, experimental verification of this theoretical finding is needed.

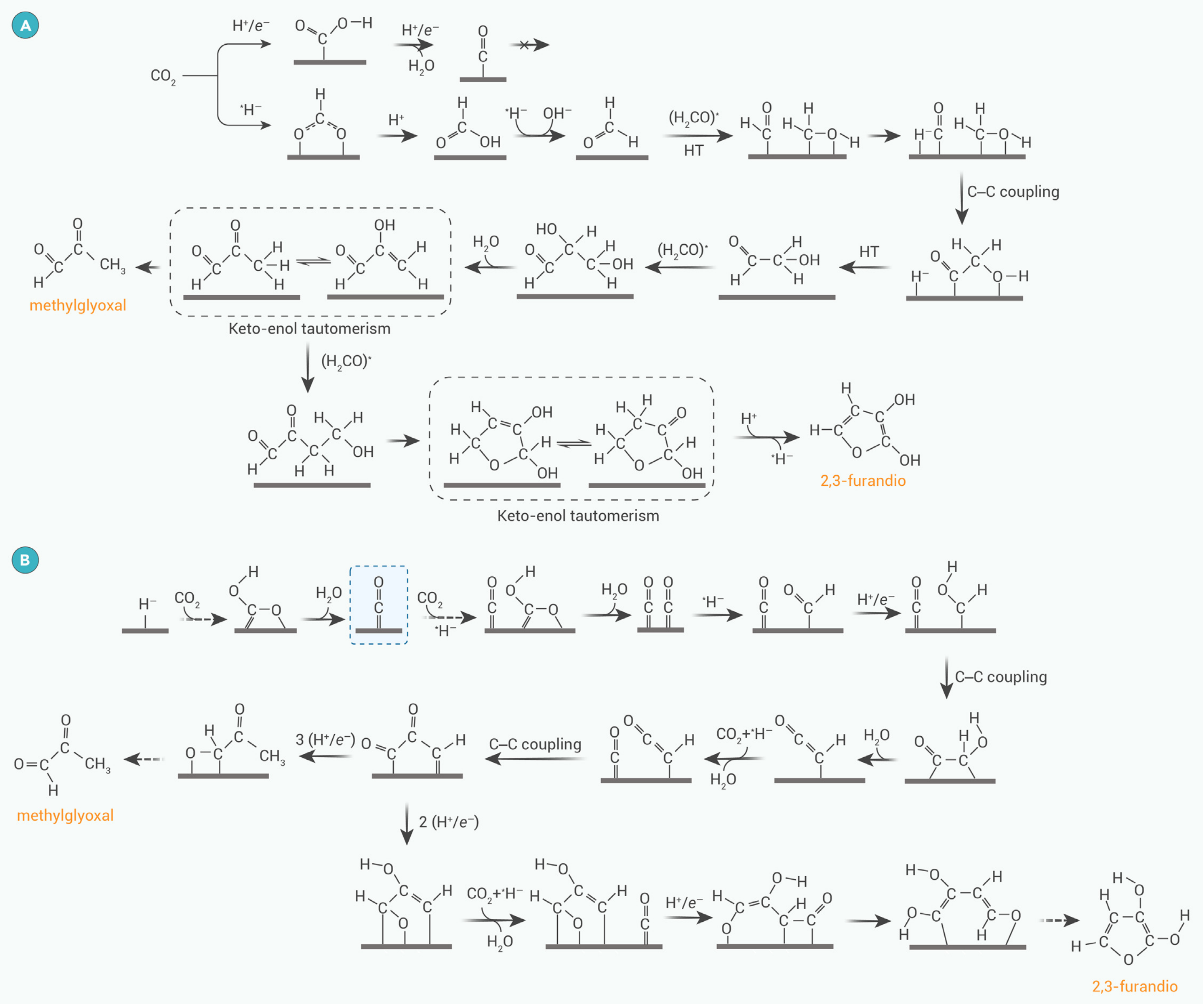

Figure 3. Possible pathways for generation of methylglyoxal and 2,3-furanediol (A) HT mechanism and (B) WAPT mechanism.

Understanding the elementary steps controlling C–C coupling and product bifurcation is crucial for targeting specific high-value products. Further exploration of reaction mechanisms after the first C–C dimerization step is needed.

Formation of 3-hydroxybutanal, n-butanol, and t-butanol. According to Cronin et al.,[51] 3-hydroxybutanal is an intermediate in *n*-butanol formation. HCOOH is considered an important two-electron unit in the pathway from which the rest of the intermediates are generated. As with the ethylene glycol-generation pathway, CO_2 is first electrolyzed to HCOOH, which is further converted to HCHO. Two molecules of HCHO then undergo self-condensation to form CH_3CHO, which then condenses to form 3-hydroxybutanal.[51] This intermediate is hydrogenated to *n*-butanol on the catalyst surface (Figure 4A). 3-Hydroxybutanal loses an α-hydrogen to form a carbene, leading to crotonaldehyde formation. Theoretical analysis of crotonaldehyde reduction suggests that butanal is formed by sequential hydrogenation of the β- and α-C of crotonaldehyde. At a reduction potential greater than −1.02 V (vs. standard hydrogen electrode [SHE]), butanal receives an electron from the cathode surface, forming a $CH_3CH_2CH_2C^{*}HO^{-}$ anion, and ultimately produces butanol.[52]

Unlike *n*-butanol generation, *t*-butanol generation involves a C_3 intermediate and a *CO–*COH coupling pathway.[53] The *CO intermediate couples to the adjacent C_1 intermediate *COH to form *COCOH, which dehydrates to CH_2CHO^{*} and is further hydrogenated to CH_3CHO^{*}. This intermediate is then converted to $(CH_3)_3CO^{*}$ by an additional PECT process and CO insertion. Alternatively, through PCET, $(CH_3)_2CO^{*}$ can be converted to $(CH_3)_3COH$ (Figure 4B), with key intermediates CH_2CHO^{*}, CH_3CHO^{*}, $CH_3CH_2O^{*}$, $(CH_3)_2COH(CO)^{*}$, and $(CH_3)_2CH_2C^{*}OH$. Although this pathway has been supported by DFT calculations, there is no experimental evidence via *in situ* detection to confirm these intermediates.

Microkinetic modeling and simulation

The numerous pathways for ECR, as elucidated in possible reaction pathways of ECR toward liquid products section, highlight the potential for producing valuable chemical feedstocks but simultaneously presents a selectivity challenge. Designing catalytic systems that selectively activate one specific product pathway requires an in-depth understanding of dominant pathway(s) and key steps under specific conditions. While DFT calculations offer insight into ECR thermodynamics and kinetics,[54] they do not fully capture operating conditions like reactant concentration, pH, and applied potential.[55,56] Microkinetic modeling, integrated with DFT calculations, addresses these gaps, offering a practical alternative to expensive *operando* techniques.

Microkinetic simulation modeling extrapolates kinetic knowledge from the microscopic to the macroscopic scale, estimating reaction rates under specific conditions. It begins with a detailed reaction scheme encompassing all reactants,

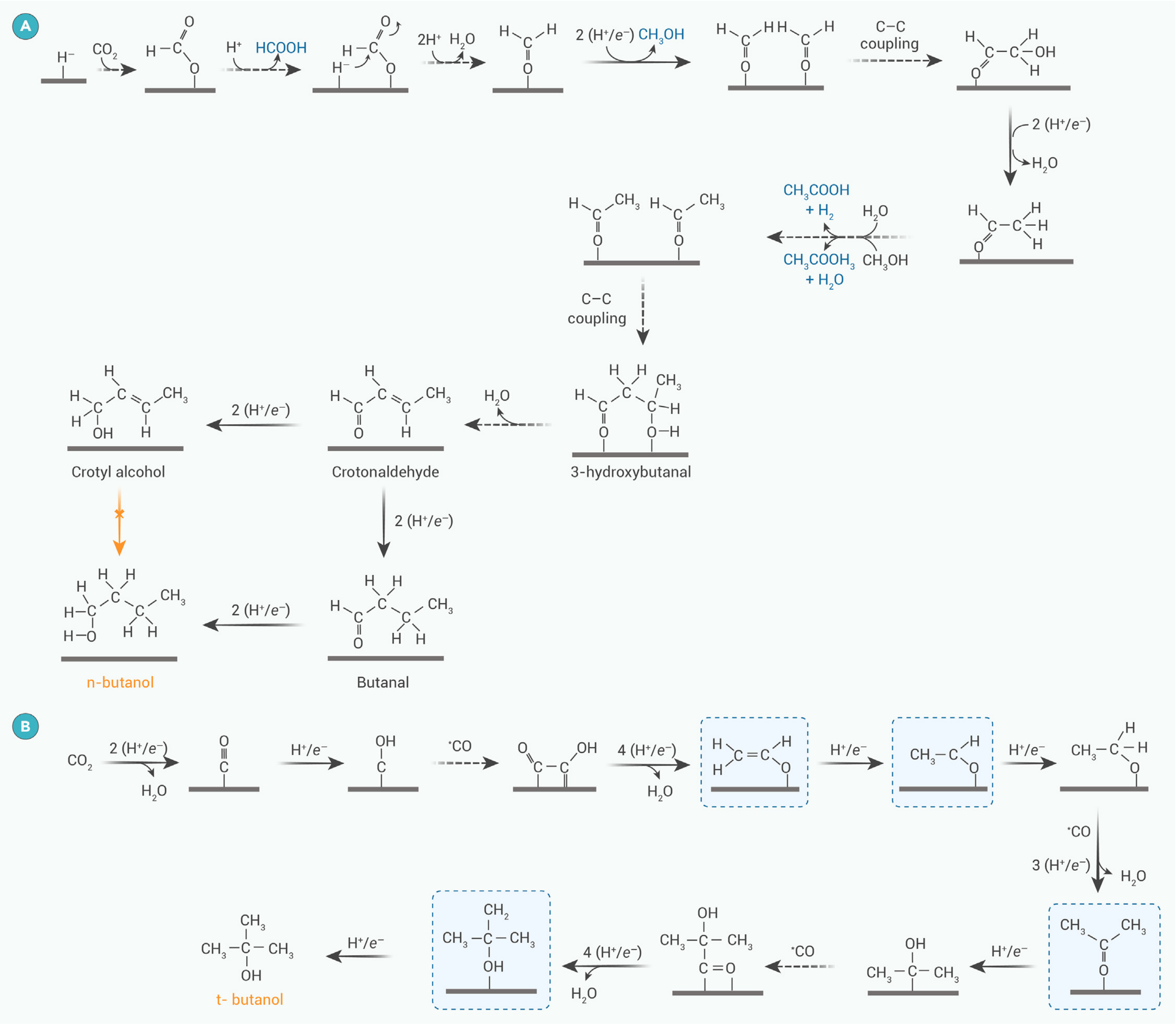

Figure 4. Possible pathways for generation of butanol (A) *n*-butanol and (B) *t*-butanol.

products, and intermediates. DFT calculations then provide kinetic and thermodynamic parameters, such as adsorption energy and activation energy. These parameters are integrated into material balance equations and rate equations to predict reaction rates of the elementary steps. However, the variety of chemical species in ECR necessitates intensive computation, making microkinetic modeling less viable. The Brønsted-Evans-Polanyi (BEP) relation and linear scaling relation can be used to simplify activation energy estimation and reaction energy prediction.[57] A linear scaling relationship, detailed elsewhere in this article, enables the estimation of reaction energies from the adsorption energy of a key intermediate. Machine learning (ML) has been utilized to diminish the number of computations needed for such modeling.[58,59] See Motagamwala's review[60] for more details about microkinetic modeling formalism.

Microkinetic modeling and simulation can also be used to describe reaction mechanisms under operational conditions, identifying dominant pathways and rate-determining steps,[60,61] as shown for the HER, oxygen evolution reaction (OER), and oxygen reduction reaction (ORR).[62–64] However, the complexity of ECR network, particularly for highly reduced products, poses significant challenges.[60] Often, only critical segments of the entire ECR network (e.g., CO → *OCCHO)[65] are modeled, or just basic reactions involving two PCETs (CO_2 → CO/HCOOH).[66] Nevertheless, due to its significance, mechanistic studies leveraging microkinetic modeling for complex ECR kinetics have been made.[61]

Liu et al. investigated the impact of applied potential and pH on ECR kinetics on Cu(211) surfaces using a microkinetic model with about 40 elementary steps, correlating their results with experimental data.[56] They identified a pathway via *OCCOH as the dominant C_2 pathway, with the rate-limiting step varied with applied potential. Under alkaline conditions, *CO dimerization is the limiting step at high overpotentials (−1.0 V vs. reversible hydrogen electrode [RHE]), explaining the faster decrease in C_2 production rate with increasing overpotential. Increasing the pH from 7 to 13 improved ECR kinetics by lowering the activation barrier for hydrogenation steps, enhancing C_2 selectivity over C_1 formation.

Recently, Kraft and coworkers modeled a comprehensive network of possible ECR pathways for nine products, including liquid fuels (HCOOH, CH_3OH, CH_3CH_2OH), consisting of 88 elementary steps.[67] They used Huang et al.'s[68] experimental benchmark to construct microkinetic simulation models at different applied potentials (U_{RHE} = −1.15, −1.0, and −0.85 V) under neutral conditions (pH = 6.8) on a Cu(100) surface. The model revealed that ECR mechanisms toward C_2 products are inactive except for C_2H_4. The dominant reaction pathway for generating HCOOH at low potential (U_{RHE} = −0.85 V) included

Table 1. Possible alternative oxidation reactions in alkali media and their respective equilibrium potentials (E^0 vs. RHE)

Possible anode reaction	Reaction equation in alkali media	E^0/V
$H_2O \rightarrow H_2O_2$	$2OH^- \rightarrow H_2O_2 + 2e^-$	1.78
$Cl^- \rightarrow Cl_2$	$2Cl^- \rightarrow Cl_2 + 2e^-$	1.36
$H_2O \rightarrow O_2$	$4OH^- \rightarrow O_2 + 2H_2O + 4e^-$	1.23
$Cl^- \rightarrow ClO^-$	$Cl^- + 2OH^- \rightarrow ClO^- + H_2O + 2e^-$	0.89
$S^{2-} \rightarrow S_x^{2-}$	$2HS^- + 2OH^- \rightarrow S_2^{2-} + 2H_2O + 2e^-$	0.14
Urea → $N_2 + CO_2$	$CO(NH_2)_2 + 7OH^- \rightarrow N_2 + HCO_3^- + 5H_2O + 6e^-$	0.07
$NH_3 \rightarrow N_2 + H_2O$	$2NH_3 + 6OH^- \rightarrow N_2 + 6H_2O + 6e^-$	0.06
$N_2H_4 \rightarrow N_2 + H_2O$	$N_2H_4 + 4OH^- \rightarrow N_2 + 4H_2O + 4e^-$	−0.33
Glucose → Gluconic acid	$C_6H_{12}O_6 + 2OH^- \rightarrow C_6H_{12}O_7 + H_2O + 2e^-$	0.90
Glycerol → Lactic acid	$C_3H_8O_3 + 3OH^- \rightarrow C_3H_5O_3^- + 3H_2O + 2e^-$	0.25
Glycerol → $HCOO^-$	$C_3H_8O_3 + 11OH^- \rightarrow 3HCOO^- + 8H_2O + 8e^-$	0.14
1,2-Propanediol → Lactic acid	$C_3H_8O_2 + 5OH^- \rightarrow C_3H_5O_3^- + 4H_2O + 2e^-$	–
Ethanol → Acetate	$C_2H_5OH + 3OH^- \rightarrow CH_3COO^- + 3H_2O + 2e^-$	0.06
$CH_3OH \rightarrow HCOO^-$	$CH_3OH + 3OH^- \rightarrow HCOO^- + 3H_2O + 2e^-$	0.11
R–CHO → R–COO^-	R–CHO + $3OH^- \rightarrow$ R–$COO^- + 2H_2O + 2e^-$	–
5-Hydroxymethylfurfural (HMF) →2,5-Furandicarboxylicacid (FDCA)	$C_6H_6O_3 + 6OH^- \rightarrow C_6H_2O_5^{2-} + 4H_2O + 4e^-$	0.30
Furfural → Furoic acid	$C_5H_4O_2 + 3OH^- \rightarrow C_5H_3O_3^- + 2H_2O + 2e^-$	–
R–CHO → R–$COO^- + H_2$	2R–CHO + $4OH^- \rightarrow$ 2R–$COO^- + H_2 + 2H_2O + 2e^-$	–
HMF → 5-Hydroxymethyl-2-furancarboxylic acid (HMFCA) + H_2	$2C_6H_6O_3 + 4OH^- \rightarrow 2C_6H_3O_4^- + H_2 + 2H_2O + 2e^-$	–
Furfural → Furoic acid + H_2	$2C_5H_4O_2 + 4OH^- \rightarrow 2C_5H_3O_3^- + H_2 + 2H_2O + 2e^-$	–
R–CH_2–NH_2 → R–CN	R–CH_2–$NH_2 + 4OH^- \rightarrow$ R–CN + $4H_2O + 4e^-$	–
Octylamine → Octanenitrile	$C_8H_{19}N + 4OH^- \rightarrow C_8H_{15}N + 4H_2O + 4e^-$	–
C_2H_4 → Ethylene oxide	$C_2H_4 + 2OH^- \rightarrow C_2H_4O + H_2O + 2e^-$ (mediated by Cl^-)	1.36
$CH_4 \rightarrow CH_3OH$	$CH_4 + 2OH^- \rightarrow CH_3OH + H_2O + 2e^-$	0.58

CO_2 physisorption followed by one PECT ($CO_2 \rightarrow *CO_2 \rightarrow *OCHO$) step. As the applied potential becomes more negative, the dominant reaction pathway changed to include a direct ($CO_2 \rightarrow *OCHO$) route, accompanied by a diminished reaction rate.

Oxidation half-reaction

The OER is the typical counter reaction at the anode in a CO_2 electrolyzer, but it requires a potential higher than the equilibrium potential of 1.23 V (vs. RHE), lowering the overall energy efficiency. This has been estimated to be no higher than 50% at a current density of 100 mA cm^{-2}, with the OER being responsible for ∼64% of the cell voltage.[69] Alternative anodic reactions that exhibit faster kinetics, higher durability, and more valuable products (than O_2) are desirable. Oxidation of glycerol to $HCOO^-$ and lactate, or the oxidation of glucose to gluconic acid, is preferred due to their lower equilibrium potential and yielding more valuable products.[70] Table 1[71] summarizes potential anodic oxidation reactions that can be used as alternatives to the OER reaction, some of which have already been coupled with ECR. Na et al.[72] conducted a technoeconomic analysis of 295 cases of co-electrolysis that couple ECR with organic oxidation. They found that the oxidation reaction processes producing carboxylic acids (e.g., formic acid[25] and 2,5-furandicarboxylic acid[73]) are the best candidates for electrochemical co-production. However, practical considerations, such as the separation of anode products from the anolyte, need to be addressed. Beyond economic benefits, the availability of organic substrates is also a key factor in achieving large-scale co-electrolysis. For example, controlling global CO_2 emissions is incompatible with the production of glycerol, creating a significant supply gap for glycerol.[70] As a result, more readily available substrates like CH_4[74] and C_2H_4[75] are preferred.

Protocols for CO_2 electrolysis

For meaningful and comparable ECR results, and to ensure consistency and reproducibility, experimental conditions such as electrolyte properties, cell configuration, CO_2 flow rate, and catalyst ink formulation need to be identical. Chemicals and commercial catalysts or raw materials used should come from the same vendors or batches to avoid uncontrolled performance variations.[76] It is important to measure intrinsic catalytic activity under conditions that minimize mass transfer effects, as ECR is susceptible to concentration polarization. A recent study highlighted this polarization by controlling the catalyst's microenvironment, where Nafion (Naf.) was employed for stabilization of the catalyst morphology during ECR. High water/alcohol ratios and low Naf. fractions in the catalyst ink created stable microenvironments, increasing the local CO_2/H_2O concentration ratio and facilitating high CO surface coverage for efficient CO_2 electrolysis.[77]

Impurity effects. Trace levels of contaminants in electrolytes, raw materials, and the reaction vessel can alter the electrode-electrolyte interface by forming new active sites or blocking catalytic sites, thus influencing the ECR activity and selectivity.[78] Metallic impurities arising from the electrode materials or electrolyte salts can be reduced and deposited on the cathode during electrolysis, intensifying the parasitic HER.[79] To remove metal impurities, a pre-cathodic polarization step or periodic CV treatment with chelating agents like ethylenediaminetetraacetic acid (EDTA) is recommended.[80] Raw CO_2 feed gas may contain impurities, such as CO, O_2, SO_x, NO_x, and volatile organic compounds. Low-concentration SO_2 can hydrogenate with *H on the catalyst surface, with the accumulated *H and *S species poisoning the catalyst. This also transforms the CO_2 hydrogenation from an Eley-Rideal mechanism, with protons from H_2O, to a Langmuir-Hinshelwood mechanism, with *H, thus suppressing *CO formation and C_{2+} production. In addition, *S coverage impedes *COOH adsorption, and the reduction of SO_2 to form *SOOH and *HSO_2 species is preferred over ECR. An SO_2-tolerant system using a perfluorosulfonic acid (PFSA)@Cu/polytetrafluoroethylene (PTFE) heterostructure was designed to decrease hydrogen adsorption and also facilitate CO_2 migration over SO_2 molecules.[81] A C_{2+} FE as high as 84% with an absolute partial current density of 790 mA cm^{-2} was attained in CO_2 containing 400 ppm SO_2.

Enhanced C_{2+} formation via co-electrocatalysis of CO_2 and O_2 to stabilize the surface hydroxyl group of Cu catalysts was reported.[82] Adding 20% O_2 to CO_2 feedstock resulted in a 170-fold, 55-fold, and 35-fold increase in the yield rate of C_2H_4, C_2H_5OH, and CH_3COO^-, respectively, at −0.75 V (vs. RHE) in 0.1 M $KHCO_3$. The yield of $C_2H_5CH_2OH$ was also observed at this potential in the presence of O_2, whereas no $C_2H_5CH_2OH$ was detectable in an oxygen-free feed gas. However, the presence of O_2 as an impurity in CO_2 led to loss of 99% of the applied current due to the occurrence of the more thermodynamically preferred ORR.[83] Operating in an acidic electrolyte can inhibit ORR on Cu, enabling oxygen-tolerant C_{2+} yield.[83]

A small amount of CO and O_2 can promote the ECR by lowering the reduction potential and altering the catalyst's oxidation state.[84] DFT studies suggest that O_2 in the CO_2 gas feed is rapidly reduced to *OH,[85] which can draw K^+ to the copper surface through electrostatic interactions, increasing the

concentration of K^+ at the interface and lowering the kinetic barrier for C−C coupling. Other contaminants may also inhibit the adsorption of CO_2 and intermediates or poison the catalyst surface and are thus detrimental to the ECR. Metal may leach from the counter electrode or from a metallic anode, which can be subsequently deposited on the cathode surface, having a significant effect on the ECR.

Standards for data acquisition and reporting. Combined metrics, including faradaic efficiency, energy efficiency, partial current density, and product-generation rate (proportional to its partial current density), should be used to report electrocatalytic activity and selectivity. For meaningful comparisons of intrinsic activity among different catalysts, measured rates and current densities must be normalized by the number of available catalytic sites and the electrochemically active surface area (ECSA), respectively, to rule out surface area effects.

Accurate assessment of high-rate CO_2 electrolysis in flow cells should consider the outlet flow rate, gas products removed by the flowing catholyte, crossover of liquid products (e.g., $HCOO^-$, CH_3COO^-, and C_2H_5OH) from the cathode to the anode, and evaporation of the liquid products (e.g., CH_3CHO, C_2H_5OH, and $C_2H_5CH_2OH$) from gas-diffusion electrodes (GDEs).[86] CO_2 utilization efficiency can be investigated using the ratio of the CO_2 reduction rate at the cathode to the sum of the CO_2 reduction rate and the HCO_3^- and CO_3^{2-} fluxes through the cell. A decrease in membrane thickness (reaction length) accelerates the transport of H^+ (with more rapid mobility than either HCO_3^- or CO_3^{2-}) from the anode to the cathode and neutralizing the CO_3^{2-}.[87] Additionally, the HCO_3^- diffusion gradient toward the cathode enables a higher CO_3^{2-} flux toward the cathode, increasing utilization. Alternatively, lowering the CO_2 partial pressure also improves CO_2 utilization by alleviating CO_3^{2-} formation and favors OH^- flux over CO_3^{2-} flux.

The FEs for gas products in flow cells can be overestimated if CO_2 consumption is not considered, especially at high current densities and low CO_2 inlet flow rates. This is due to the formation of CO_3^{2-} stemming from the reaction of CO_2 with the OH^- at the cathode, which migrates to the anode, converts back to CO_2, and leaves the cell through the anolyte. Bubble accumulation at the electrode-electrolyte interface and evaporation of liquids along with the outlet gas are accelerated under larger current densities and faster catholyte flow rates, while the issue of crossover becomes more severe at lower volumes of catholyte and reduced catholyte flow rates. To mitigate these problems, the outlet gas can be scrubbed by the catholyte, which is then mixed with potential gas products from the catholyte for online gas chromatography (GC) quantification through a mass flow meter. The application of a bipolar membrane (BPM) instead of an anion-exchange membrane (AEM) can hinder electromigration and electroosmotic drag and alleviate crossover. Note that C balance analyses should be conducted for CO_2 electrolysis operated at large cathodic currents to ensure that CO_2 consumption is fully accounted for.

Overall, unified protocols are required for reporting, measuring, and analyzing all of the above metrics for an objective evaluation of ECR catalysts.

Temperature and pressure effects. Increasing the operating temperature to within certain ranges can improve reaction rates and energy efficiency (i.e., the applied bias divided by the thermodynamic voltage); however, a mechanistic explanation for temperature effects on the ECR is still lacking. Temperature can affect the local pH, CO_2 solubility, reactant diffusion rates, intermediate adsorption, and electrolyte resistance.[88] Higher temperatures result in lower CO_2 solubility due to Henry's law and the acid/base buffer equilibria (CO_2/HCO_3^-/CO_3^{2-}) and also favor the parasitic HER. As a result, the enhancement in ECR kinetics may be counteracted by a decrease in CO_2 solubility. An optimal working temperature needs to be ascertained that balances these factors. 50 °C was reported to be preferred for $HCOO^-$ formation on a SnO_2-based GDE with an $HCOO^-$ FE of over 80% at a current density of -1.0 A cm^{-2}.[89] Above or below 50 °C, more severe HER occurred. The optimal reaction temperature varies for different catalysts, and the highest temperature that both the GDE and membrane can sustain should also be acknowledged.

Elevating the CO_2 pressure in liquid electrolytes augments its solubility from 0.03 M under ambient pressure to 1.16 M at 50 bar.[90] This maintains the bulk catholyte pH at ~6.2, which otherwise increases gradually due to the stoichiometric OH^- production. Enhanced $HCOO^-$ selectivity was observed under pressure in the 1−50 bar range on a variety of catalysts, which was attributed to the higher CO_2 coverage and lower proton concentration on the cathode surface under elevated pressure, favoring $HCOO^-$ formation.[91]

Tafel slope of CO_2 reduction. The kinetics, mechanism, and selectivity of ECR are largely controlled by the applied potential. The dependence of the measured current or product formation rate on the applied potential provides insights into both the reaction pathway and underlying mechanism of CO_2 reduction. The reaction pathway and mechanism of simple electrocatalytic reactions involving the transfer of a single electron or two electrons can be determined experimentally through Tafel slope analysis, complemented by observation of reaction intermediates and products using *in situ* analytical techniques and through kinetic modeling and simulation. However, experimental determination of the Tafel slope for products involving the transfer of more than two electrons is prone to error. Extrinsic factors, such as electrolyte pH and particle size and morphology, are also known to influence the selectivity and thus mechanism of ECR, further complicating the experimental deduction of ECR reaction mechanisms using Tafel slopes from current-potential dependencies. Tafel slopes for the two-electron reduction of CO_2 to CO or HCOOH can be determined with better reliability but only in the case of 100% selectivity and absence of concurrent background reactions and mass transport limitations. In effect, the associated kinetic parameters such as electron-transfer coefficient and exchange current density for ECR cannot be reliably determined experimentally.

The Tafel slope for ECR on Ag and Au, which are renowned for reducing CO_2 predominantly to CO, is invariably very close to 59 mV dec^{-1}, which precludes $CO_2 + e^- \rightarrow CO_2^-$ as the initial electron-transfer step.[92] ECR on Au(111) yields almost exclusively CO in ionic liquid electrolytes.[93] Owing to the aforementioned factors, it is challenging to experimentally determine ECR pathways and mechanisms for C_{2+} products that involve more than four PECT steps and multi-C coupling steps just from current-potential relationships. Online spectroscopic techniques coupled to electrochemical cells, and downstream product analysis, are thus frequently employed for reliably deducing ECR reaction pathways and mechanisms.[94]

HETEROGENEOUS CATALYSIS OF ECR TO LIQUID PRODUCTS

Selectivity of HCOOH/$HCOO^-$ formation

In pioneering work conducted by Hori and coworkers,[95] metallic catalysts were divided into four categories according to their primary ECR products, among which the *p*-block metals In,[96] Sn,[97] Pb,[98] Bi,[99] and Cd[80] are conducive for HCOOH generation. The benchmark FE for HCOOH formation is nearly 100% in H-type electrolytic cells with advanced catalysts, while the maximum HCOOH FE in a flow cell is 93% (Table S2). The highest reported absolute cathodic partial current density toward HCOOH is -2.0 A cm^{-2} at -0.95 V (vs. RHE) on a Bi_2S_3-derived catalyst in a flow cell, with stable performance for 100 h.[100] The actual active sites for CO_2 reduction on Bi-catalyzed ECR are debated, with metallic Bi and Bi^{3+} both suggested.[101] Bismuth oxide catalyst for ECR in a CO_2 electrolytic cell to produce ~5 M HCOOH gave an average FE of 80% in the first 100 h but decreased to about 75% after 500 h and 65% ~to 70% after 1,000 h.[102]

Fang and coworkers[103] used a Pb catalyst from waste lead-acid batteries to convert CO_2 into HCOOH in a proton exchange membrane (PEM) system, achieving over 93% FE in an electrolyte with pH 1.0 under a full-cell voltage of 2.4 V. They showed stable performance for 5200 h at 2.2 V and -600 mA cm^{-2}. This PEM system with cathodic ECR and anodic hydrogen oxidation reaction (HOR) offers a new concept for CO_2 catalytic conversion.

Selectivity of CH_3OH formation

To date, the highest FE for ECR to CH_3OH has been reported on a $Co(CO_3)_{0.5}(OH)\cdot 0.11H_2O$ catalyst, reaching 97% at -0.98 V (vs. saturated calomel electrode [SCE]) in a 0.5 M $NaHCO_3$ electrolyte.[104] However, the CH_3OH partial current density was only -0.59 mA cm^{-2}. For most catalysts in aqueous electrolytes, the current density is often below 50 mA cm^{-2} when the CH_3OH FE exceeds 50% (Table S3).

Cu-based catalysts show excellent potential for ECR into CH_3OH. Cuprous cyanamide (Cu_2NCN) achieved an FE of 70% for CH_3OH production, with a partial current density of -92.3 mA cm^{-2}.[105] Using 0.5 M $KHCO_3$ as the electrolyte, the production rate of CH_3OH in a membrane electrode assembly (MEA)-based electrolyzer reached 0.16 μmol s^{-1} cm^{-2}. Cu_2NCN significantly reduces the Cu−O interaction of the adsorbed *OCH_3 intermediate, making it weaker than the O−C interaction at the critical reaction bifurcation point of Cu−*O−CH_3, directing the reaction pathway toward the release of *OCH_3 and the formation of CH_3OH.

Mahrim and coworkers discussed using ionic liquids to form catalytic Cu NPs for reducing CO_2 to HCOOH.[106] Recently Noh et al.[107] showed that imidazolium cations (from ionic liquids) can promote *CO formation during ECR by lowering the activation energy to about zero through lower entropy ordering of imidazolium cations at the electrode surface. The negative view[108] of ionic liquids as potential electrolytes for ECR is disappearing as their costs decrease.

In addition to metal-based catalysts, metal-free catalysts for efficient CH_3OH generation remains challenging. B-doped diamond[109] and B, N co-doped nanodiamonds[110] have been used to convert CO_2 into CH_3OH, but the FE is below 30%. The best performance for a metal-free catalyst for CH_3OH formation was observed on boron phosphide, which afforded a 92% FE at −0.5 V (vs. RHE) in a 0.1 M $KHCO_3$ electrolyte,[111] although it was attained at a low current density.

Selectivity of C_2H_5OH formation

A low onset potential of −0.4 V (vs. RHE) for CO_2 conversion to C_2H_5OH was attained with Cu_x clusters (x = 3 and 4) stabilized by hydroxyl groups and water on a C support. This catalyst delivered a remarkable C_2H_5OH FE of 91% at −0.6/−0.7 V (vs. RHE).[112] However, the obtained maximum C_2H_5OH partial current density (1.12 mA cm^{-2}) is too low for industrial applications and declined when the Cu loading was increased to 1.6%. Kinetic analysis is required to elucidate whether the reaction is via either an HCOO* and/or a CO* mechanism. Cu-based catalysts for C_2H_5OH FEs are often lower than 70% when the absolute partial current density is greater than 200 mA cm^{-2} (Table S4). Recently, Hu et al.[113] constructed oxygen-bridged binuclear Cu sites on mesoporous N-doped C nanocages, with an FE for C_2H_5OH of 56.3% at a potential of −0.3 V (vs. RHE), which represents the lowest overpotential reported to date.

Beyond Cu, transition metal phosphides such as Ni_2P, FeP, and MoP also enable C_2H_5OH formation.[114] In particular, a MoP electrocatalyst coated with an imidazolium-functionalized ionomer provides a C_2H_5OH FE of 77.4% at a potential of only −200 mV (vs. RHE).[114] CoO NPs immobilized on N-doped mesoporous C and C nanotubes were shown to yield C_2H_5OH with an FE of 60.1% at −0.32 V (vs. RHE) and a partial current density of −5.1 mA cm^{-2}.[115] Ag particles anchored on graphene-wrapped N-doped C foams were reported to give a higher C_2H_5OH FE of 82.1–85.2% at −0.6 to −0.7 V (vs. RHE).[116] The good activity was attributed to pyridinic N species in the catalyst enhancing the binding of *CO intermediates, while the Ag particles promote the conversion of *CO to *OC−COH and then to C_2H_5OH.

A 93.2% FE for C_2H_5OH was observed on B, N co-doped nanodiamond at −1.0 V (vs. RHE).[110] The doped B atom improves CO_2 capture by connecting with one O atom of $*CO_2$, while the doped N facilitates *H transfer during hydrogenation. B atoms play an important role by forming B−O bonds in the steps required for C_2H_5OH formation.

Selectivity of CH_3COOH/CH_3COO^- formation

Only a few catalysts selectively produce CH_3COOH/CH_3COO^-. Polymeric Cu-ligand complex core-shell microspheres can catalyze the ECR to CH_3COO^- with an FE of ∼64% in a 0.5 M $KHCO_3$ electrolyte at −0.37 V (vs. RHE).[117] A higher CH_3COO^- FE of 90.3% at a more negative potential (−0.8 V vs. RHE) was achieved by using a conductive 2D copper phthalocyanine-based covalent organic framework (COF) electrocatalyst in 0.1 M $KHCO_3$,[33] with stable performance over 80 h.

Reduction of CO_2 to CH_3COOH at −0.1 V (vs. RHE) for up to 100 h was obtained on bismuth-based transition metal chalcogenides, such as $AgBiS_2$, $CuBiS_2$, and $AgBiSe_2$.[118] Lone-pair electrons in Bi^{3+} expedites adsorption and activation of CO_2 by assisting electron transfer, while nucleophilic chalcogens boost water activation and trap CO_2, forming C-bound $CO_2^{\cdot -}$ species. The shorter X−Bi (X = S/Se)-bond distance of $AgBiSe_2$ and $CuBiS_2$ favors closer proximity of adsorbed C atoms relative to $AgBiS_2$, which enables C−C coupling to generate CH_3COOH at lower potentials. The facile charge transfer between Bi and Se in $AgBiSe_2$ accelerates adsorption of the *COOH species, leading to a higher CH_3COOH FE on $AgBiSe_2$ compared to $CuBiS_2$.

A tandem catalytic system consisting of a Ni-based COF and a Cu-(3,5-diethyl-1,2,4-triazole) metal-organic framework was designed to convert CO_2 to CO and subsequently CO to CH_3COO^- via a *CCO intermediate, respectively.[119] A CH_3COO^- FE of 51.2% with a current density of −410 mA cm^{-2} was attained using an MEA electrolyzer with a solid-state electrolyte (SSE), yielding up to 2.72 mmol m^{-2} s^{-1} CH_3COO^- at a cell voltage of 3.1 V. Competition between the CO donor and CO consumer was proposed, favoring ECR to produce CO over CH_3COO^-.

Aside from catalyst design, selective ECR of CH_3COOH can also be achieved by adjusting the type and properties of the electrolyte. Using the Keggin-type polyoxometalates (POM) $[SiW_9V_3O_{40}]^-$ (SiW_9V_3) as a catholyte,[120] the CH_3COO^- FE on In reached 96.5% at −0.7 V (vs. RHE). The impressive CH_3COO^- FE was linked to the V-center valence transition of SiW_9V_3 during electrolysis of the CO_2, where the electron-transfer process in SiW_9V_3 participates in the ECR, leading to reduced overpotential of the reaction.

Selectivity of CH_3CHO formation

The production of CH_3CHO during ECR is seldom reported. By using synergistic catalysis of Au (for CO_2 to *CO), Cu (for *CO dimerization), and 4,4′-bipyridine (for CO_2* stabilization and protonation), an FE of ∼25% for CO_2 to CH_3CHO conversion was achieved at −0.9 V (vs. RHE).[121]

A high CH_3CHO FE of up to 60% with a cathodic current density of −5.1 mA cm^{-2} at −0.4 V (vs. RHE) was obtained using hexagonal-close-packed Co nanosheets in a 0.5 M $KHCO_3$ electrolyte.[122] Interlayer electron transfer in the Co nanosheets promoted *[OC−CO] coupling and inhibited the complete hydrogenation of the intermediates to C_2H_4, thereby resulting in a high CH_3CHO selectivity.

Ag adatoms on Cu were shown to weaken the binding strength of CH_3CHO intermediates and hinder its further conversion to C_2H_5OH, thus enabling the reduction of CO to CH_3CHO with ∼70% FE and over 90% selectivity on a C basis at −0.536 V (vs. RHE).[123] Inspired by this finding, a route to improve CH_3CHO yield would be the design of a cascade system combining the CO_2-to-CO step and CO-to-CH_3CHO step.

Selectivity of $(CH_2OH)_2$ formation

The electrochemical reduction of CO_2 to diols is challenging due to the need to retain both oxygen atoms. The unique spatial-confinement provided by densely arrayed Cu nanopyramids (Cu-DAN) has been predicted to facilitate C−C coupling in the ECR to generate $(CH_2OH)_2$. The structure is supposed to preserve the oxygen atoms and favor the *COH−CHO pathway toward $(CH_2OH)_2$ and C−O bond cleavage in $*CH_2OH-CH_2O$ intermediates is also inhibited.

CO_2 can be reduced to $(CH_2OH)_2$ at low overpotentials on Fe_2P, reaching 10% FE at 0 V (vs. RHE) and ∼25% at −0.05 V in 0.5 M K_2SO_4.[37] The strong Fe−P bond and weak hydride adsorption energy on Fe_2P promote the glyoxal intermediate to be rapidly hydrogenated, suppressing the formation of long-chain C_3 and C_4 products. HCOO*, rather than *CO, is first generated from surface phosphino-hydrides, which is then converted to $*H_2CO$ that undergoes C−C coupling with other $*H_2CO$ molecules on adjacent Fe sites to yield $(CH_2OH)_2$.

Selectivity of $C_2H_5CH_2OH$ formation

In early studies of the ECR reaction, Hori et al.[124] observed the formation of $C_2H_5CH_2OH$ when using a Cu foil electrode. However, the reported FE (3.0% at −1.44 V vs. normal hydrogen electrode [NHE]) is low. The highest $C_2H_5CH_2OH$ FE reported thus far originates from graphene/ZnO/Cu_2O heterostructures (Table S5).[125] The composite was supposed to stabilize Cu(I) species and improve the selectivity of $C_2H_5CH_2OH$. The maximum FE of $C_2H_5CH_2OH$ was 30%, attained at −0.9 V (vs. Ag/AgCl), but the partial current density was only −0.55 mA cm^{-2}. Zhang et al.[126] achieved a $C_2H_5CH_2OH$ FE of 12.1% at a potential of −1.1 V (vs. RHE) with a partial current density reaching −101.6 mA cm^{-2} by constructing bicontinuous Cu_2O/Cu nanodomains. By using a two-step tandem catalytic system, where the CO_2-to-CO step occurs on Ni single atoms and the subsequent CO-to-$C_2H_5CH_2OH$ step occurs on Cu_2O, yielded a $C_2H_5CH_2OH$ FE of 15.9% along with a half-cell power conversion efficiency of 19.3%.[127] A Br-bridged dinuclear Cu(I) complex (CuBr−4PP) that has a ligand rich in π electrons and robust active sites, $Cu_2(\mu\text{-}Br)_2$(triphenylphosphine)$_2$(4-phenylpyridine)$_2$, was recently demonstrated to enable $C_2H_5CH_2OH$ production with an FE of approximately 10% at −2.2 V (vs. Ag/AgCl).[128] The CuBr−4PP was capable of adsorbing and retaining CO_2 owing to the phenyl group in the complex. A bridging intermediate between two Cu sites (e.g., $[Cu-CHOCO-Cu]^+$) was formed, which flexibly altered the Cu−Cu distance, attracting reducing moieties to one Cu with another inserted intermediate coordinated to the other Cu. C−C coupling occurred at the bridging site in the flexible Cu binuclear complex, giving rise to $C_2H_5CH_2OH$. The structure and Cu(I) oxidation state of the molecular catalyst were preserved during the ECR.

www.the-innovation.org

Besides Cu, MoS_2 electrodes were first observed to catalyze the ECR to produce $C_2H_5CH_2OH$ as the major ECR product with an FE of 2%–5% in 0.1 M Na_2CO_3 acidified to pH 6.8 and 1 atm CO_2.[129] The terraces rather than edges of MoS_2 were hypothesized to be the active sites for reduction of CO_2 to $C_2H_5CH_2OH$. Despite the progress outlined above, selective production of $C_2H_5CH_2OH$ still remains challenging.

Selectivity of methylglyoxal (CH_3COCHO) and 2,3-furanediol formation

CH_3COCHO is an intermediate in the formation of 2,3-furanediol. Nickel phosphide catalysts such as NiP_2 can convert CO_2 into methylglyoxal with an FE of 84% at −1.0 V (vs. RHE) in 0.5 M $KHCO_3$.[49] Ni_2P can reduce CO_2 to 2,3-furanediol with a 71% FE at 0 V (vs. RHE), which is attributed to the multi-center synergy of densely dispersed Ni_3 sites.[50] The trinuclear Ni_3 catalytic center in Ni_2P can adsorb two C_1 species with a shortened distance as compared to that on a metal surface, benefiting C−C coupling. In addition, the electron-delocalized Ni_3 sites can promote electron transfer to adsorbates, which is conducive to their activation. Furthermore, transfer of surface protons between neighboring Ni_3 sites facilitates *CO hydrogenation and reduces the energy penalty for C−C coupling. Despite this low overpotential and high C_3 and C_4 selectivity, the absolute cathodic partial current densities toward methylglyoxal and 2,3-furanediol are low, less than 1.0 mA cm^{-2}, indicating a need for further study to enhance reaction rates.

Selectivity of *n*-C_4H_9OH, 3-hydroxybutanal ($CH_3CHOHCH_2CHO$), and *t*-C_4H_9OH ($(CH_3)_3COH$) formation

n-C_4H_9OH was first observed to be generated on CuO-derived Cu with an FE of 0.056% and a partial current density of −0.08 mA cm^{-2} at −0.48 V (vs. RHE) in an alkaline medium.[130] CO_2 was initially electro-reduced to CH_3CHO, which was then converted to 3-hydroxybutanal via a base-catalyzed aldol condensation reaction followed by OH^- elimination to form crotonaldehyde ($CH_3CHCHCHO$) on Cu, with a final two-step electroreduction to *n*-C_4H_9OH. The highest FE for *n*-C_4H_9OH formation attained was up to 42% at −1.48 V (vs. Ag/AgCl) and pH 4.1 on Ni-incorporated $(Cr_2O_3)_3(Ga_2O_3)$.[51] A cascade scheme was proposed; i.e., $(Cr_2O_3)_3(Ga_2O_3)$ generates CH_3CHO while Ni catalyzes its aldol condensation and further hydrogenation to form *n*-C_4H_9OH.

During the production of *n*-C_4H_9OH, $CH_3CHOHCH_2CHO$ was also detected as the primary ECR product in the potential range from −1.0 to −1.4 V (vs. Ag/AgCl) on Ni-enhanced $(Cr_2O_3)_3(Ga_2O_3)$.[51] The maximum observed FE of $CH_3CHOHCH_2CHO$ was ~23% at −1.4 V (vs. Ag/AgCl), beyond which it was converted to *n*-C_4H_9OH.

Selective formation of C_4H_9OH is attractive with facile phase separation from water at concentrations above 9%. Multiple liquid products may be generated during ECR, and they are typically mixed with the electrolyte solvents such as $KHCO_3$ and KOH. In application scenarios, additional separation and concentration steps are needed to recover the pure liquid products from solution, and the extra downstream separation steps increase the cost of industrial ECR technology.

Converting CO_2 to $(CH_3)_3COH$ has been rarely reported. Only Cu_xIr_{1-x} alloy NPs with oxophilic Ir-rich surfaces were found to catalyze the ECR to produce $(CH_3)_3COH$ with reasonable selectivity.[53] A $(CH_3)_3COH$ FE of ~14.8% with a partial current density of ~ −0.21 mA cm^{-2} was attained at −0.57 V (vs. RHE) on $Cu_{0.48}Ir_{0.52}$ NPs. The C_4 product likely evolves from a C_3 intermediate $(CH_3)_2CO$ on CuIr. Hydrogenation of CH_2CHO* to CH_3CHO* appears to be the potential-determining step (PDS) for formation of $(CH_3)_3COH$. Oxophilic Ir helps stabilize the O-bound intermediates to facilitate the generation of the C_4 product.

COMPUTATIONAL SCREENING OF MATERIALS FOR ECR TO YIELD LIQUID PRODUCTS

The pursuit of effective catalysts for ECR to produce liquid fuels has led to exploring diverse materials like two-dimensional (2D) materials and alloys[131–135] However, the vast structural and compositional diversity within this domain presents an exploration challenge for simple trial-and-error approaches. Computational screening, aided by increased computation power and workflow management tools such as FireWorks and the Atomic Simulation Environment (ASE),[136,137] offers systematic and cost-effective approaches for material evaluation and design.[138] This section highlights how computational screening leverages DFT calculations and ML approaches for catalyst selection.

DFT calculations for ECR catalysts screening

Since the 2000s when early works of high-throughput screening emerged,[139–141] DFT-based computational screening has matured as a prominent method for identifying promising catalyst candidates for various reactions. Effective screening requires well-defined descriptors and criteria, tailored to the specific domain. This section highlights key descriptors obtained by DFT calculation for screening stable, active, and selective ECR catalysts.

Stability descriptors like formation energy, ΔE_f, and dissolution potential, U_{dis}, ensure thermodynamic and electrochemical stability, guided by negative formation energy and positive dissolution potential.[127,133] In single-atom catalysts (SACs) or dual-atom catalysts (DACs), adsorption energy, ΔE_{ad} (or binding energy, E_{bind}), of the atom(s) and its diffusion barrier on the host inform of possibility of agglomeration of the single/dual atoms into particles.[133,142,143] Molecular dynamics (MD) simulations can observe notable structural modifications, such as the detachment of a single atom in SAC with increasing temperature.[143]

For catalyst activity, limiting potential is a straightforward activity descriptor. The computational hydrogen electrode[144,145] helps establish the energetics of complex electrochemical reactions involving proton-electron pairs. The elementary step with the highest barrier is identified as the PDS, with its reaction barrier, ΔG_{max}, expressed as $\Delta G_{max} = \max\{\Delta G_1, \Delta G_2, \ldots, \Delta G_N\}$, where N is the number of elementary steps. The limiting potential, U_{lim}, is defined as $U_{lim} = -\Delta G_{max}/ne$, where n is the number of electrons involved in the PDS. Thus, less positive ΔG_{max} or less negative U_{lim} is a straightforward indicator for lower overpotential and higher activity of the catalyst.[142,146]

ΔE_{ad}(CO) is another popular activity descriptor. Linear scaling relationships between the ΔE_{ad} of adsorbates with equivalent binding species[147] lead to ΔG_N as a linear function of ΔE_{ad} of a key adsorbate. This linear scaling relationship simplifies catalyst design into finding optimal ΔE_{ad} values, often represented in a volcano plot, a quantitative manifestation of Sabatier's principle. The ΔE_{ad}(CO) and ΔE_{ad}(OH) are mostly adopted as ECR activity descriptors in the linear scaling relationship scheme.[148,149] As another example of catalytic activity descriptor, the $\Delta E_{ad}(CO_2)$, or first PCET step energy, $\Delta G_{(*CO2 + H+ + e- \rightarrow *COOH/*HCOO)}$, was utilized to ensure CO_2 activation.[127,150]

Selectivity is a crucial attribute of an ECR catalyst. A catalyst's proficiency in exclusively activating specific ECR products while inhibiting others is assessed by comparing the limiting potentials of competing reactions. For instance, a positive difference in limiting potentials between ECR and HER indicates the selectivity of ECR over the HER.[133]

Selectivity for a specific ECR product is also evaluated by comparing the limiting potentials for different ECR pathways or reaction energies at a bifurcation point.[127,148,151] For example, comparing $\Delta G_{(*HCOOH + H+ + e- \rightarrow *CHO)}$ and $\Delta G_{(*HCOOH \rightarrow HCOOH(l))}$ ensures selective HCOOH production on an SAC on supported silicomolybdic acid.[143] Zhi et al. leveraged M−H affinity and M−O affinity as another selectivity descriptor to categorize 12 SAAs into three groups by their products: two-electron-transfer products (CO and HCOOH), more reduced C_1 products (CH_4 and CH_3OH), and C_2 products.[146] Pederson and coworkers utilized ΔG_{ad}(CO) and ΔG_{ad}(C) as selectivity descriptors to screen C_{2+}-selective binary alloy surfaces out of 142 candidates.[152] Their selectivity maps, including one in Figure 5A, suggested Cu-based and non-Cu-based binary alloys in the green-colored region as the C_{2+}-selective alloy.[153] The rationale for employing ΔG_{ad}(CO) and ΔG_{ad}(C) as selectivity descriptors is that coupling of C and CO is a feasible route to C_{2+} chemical production. However, establishing the C−C coupling mechanism is still under debate, so careful selection of descriptors is necessary.[56,65,154–156]

In summary, combining stability, activity, and selectivity descriptors helps identify promising ECR catalysts. Behrendt et al. used high-throughput computations to screen over 450 dual-atom alloy (DAA) or SAA combinations embedded in Cu(111) for C_{2+}-selective ECR.[157] The initial selection prioritized thermodynamically stable and potential CO_2-activating catalysts by excluding materials with positive ΔE_f and $\Delta E_{ad}(CO_2)$ greater than −0.5 eV, yielding approximately 100 viable candidates located in the bottom-left region of Figure 5B. The selection was further refined based on the proximity to optimum ΔE_{ad}(CO) on Cu(111), −0.93 eV. Too-strong CO adsorption leads to CO poisoning, while weak CO adsorption leads to CO desorption without C−C coupling, represented in the red region and the yellow region of Figure 5C, respectively. Their work suggested potential ECR catalysts for C_{2+} products, highlighting two SAAs and seven DAAs. The results of this work, as well as other promising ECR catalysts suggested by

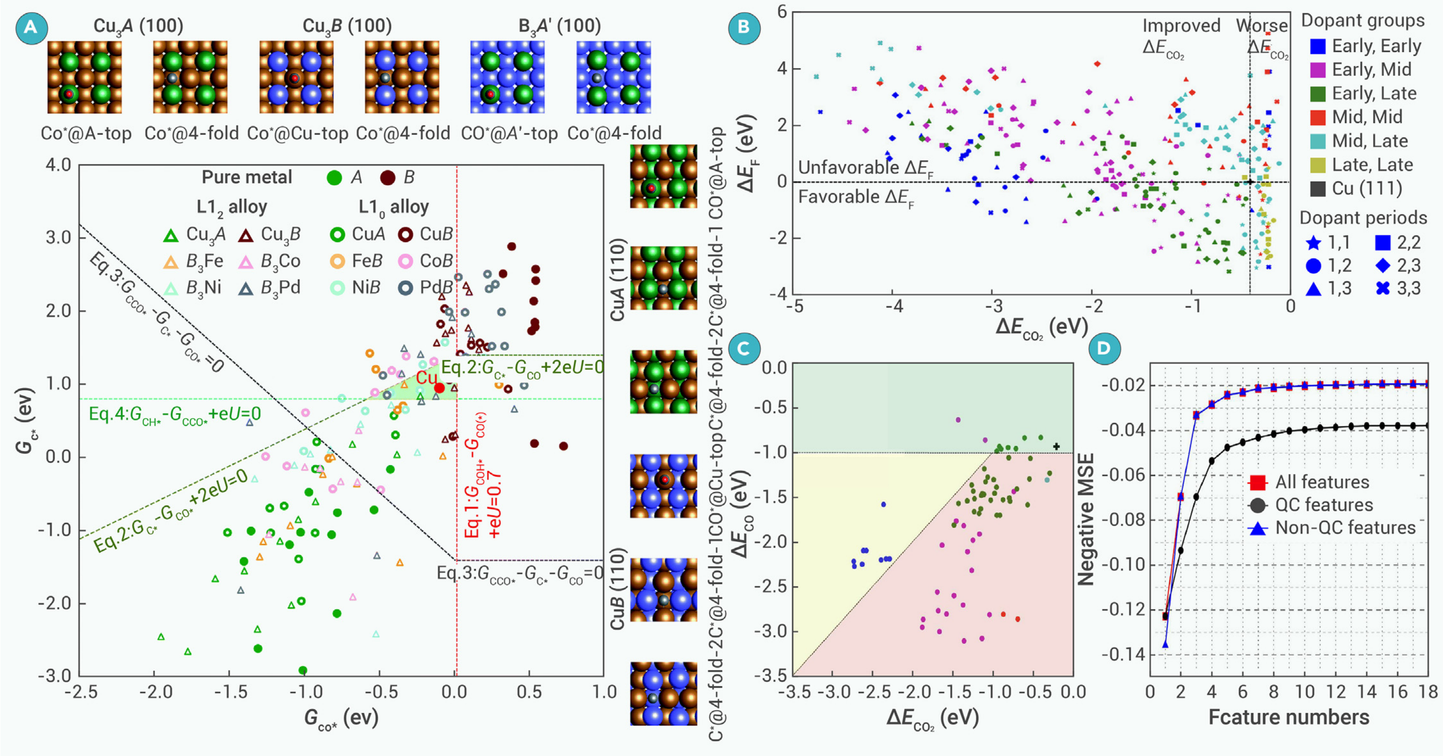

Figure 5. Computational screening for ECR to liquid products (A) Selectivity map of (100) $L1_2$ and (110) $L1_0$ alloy surfaces (U_{RHE} = −0.7 V, pH = 7). The light green region represents the sets of promising surfaces with primary selectivity toward $C_{2(+)}$ products.[153] Copyright 2022, Royal Society of Chemistry. (B) Scatterplot showing the screening results for the initial set of candidate DAAs. The dotted lines denote the energies of Cu. SAAs are considered to be DAAs with the late transition metals (TM) being Cu. Early TM, groups 3–5; mid TM, groups 6–9; late TM, groups 10–12. Gamma-point high-throughput calculations are performed for all the alloys.[157] Copyright 2023, American Chemical Society. (C) The candidates in the green region have CO binding similar to pristine Cu (black cross). The yellow and red regions represent materials whose CO adsorption is stronger than desired, while materials in the yellow region could be outcompeted by stronger CO_2 binding, and materials in the red regions are prone to CO poisoning.[157] Copyright 2023, American Chemical Society. (D) Cross-validation scores (negative mean square error [MSE]) versus the corresponding feature numbers of the best ML models obtained by the modified sequential forward selection (SFS) algorithm.[158] Copyright 2023, American Chemical Society.

computational screening research, are listed in Table S6 with target liquid fuels and corresponding limiting potential.

ML methods for high-throughput screening of ECR electrocatalysts

The ML method streamlines the prediction of catalyst properties using a trained non-linear surrogate function, reducing reliance on expensive DFT calculations. This expands the exploration space in computational screening for ECR catalysts, allowing the consideration of multi-elements and complex structures.[159–162] Notable achievements include suggestions of novel binary intermetallics[163,164] and a DAC with an unreported structure,[165] followed by experimental validation. This section outlines the ML approach for ECR catalyst screening and discusses strategies to address challenges in ML-aided ECR catalyst screening and future research prospects.

ML-aided ECR catalyst screening. ML methods have aided ECR catalyst design, where, for example, Ma et al. demonstrated their effectiveness over traditional *d*-band-center-based models in predicting ΔE_{ad}(CO) on multi-metallic alloys.[166] The ML-aided research process encompasses task definition, data collection, feature engineering, model selection, and validation, with emphasis on selecting appropriate features and models for better accuracy and generalizability. The development of feature and model selection has enhanced ML model prediction accuracy to below DFT uncertainty levels (~0.2 eV), supporting the feasibility of ML in aiding computational screening.

We distinguish the term "feature" from "descriptor" for clarity throughout this section. Often, they are used interchangeably, but in the current manuscript we use feature to emphasize its role as an input for the ML model while descriptor serves as a measure of catalytic property as used in the previous section.

Feature engineering is a process of selecting and optimizing features that effectively represent materials, such as atomic radius, atomic number, coordination number, valence electrons, and electronegativity.[167] Effective feature selection, such as Wu et al.'s identification of six dominant features out of 52 features to predict CO dimerization energy,[168] enhances model performance and interpretability while avoiding overfitting. Feature importance weighing, Pearson correlation coefficient, or advanced algorithms like sequential forward selection have been used to refine feature sets without significantly compromising accuracy.[158,168–171] Composite features and new feature development can further improve model accuracy.[169,172–174] Ding et al. transformed 88 features into 10 mathematical expressions by a symbolic transformer, significantly enhancing prediction accuracy in limiting potentials of ECR toward HCOOH.[174] Intrinsic descriptors, estimated from valence electrons and electronegativity, can effectively predict ECR intermediate adsorption behavior.[160,175–177] Additionally, novel features like the Fukui function, complemented with work function, showed strong predictive power for ΔE_{ad}(CO) on different facets of Ni and NiGa alloys.[173] Context-specific features for alloys, with encapsulating information about the adsorption site environment, are vital as they determine the electronic structure of the surface via ligand effects. The microstructure of the adsorption environment can be divided into zones according to the adjacency to the binding site. Pederson et al.[178] and Roy et al.[135] converted the zone-divided microstructure into a feature vector, while Mok et al.[179] and Roy et al.[177] in their successive works incorporated the atomistic properties with zone information. Good features, containing physically and chemically relevant information, should be readily obtainable. Noh et al.[180] and Xing et al.[149] demonstrated the effectiveness of non-*ab initio*-based features in predicting ΔE_{ad} of ECR intermediates using kernel ridge regression (KRR) and gradient-boosting regression (GBR) models, respectively. Furthermore, it was reported, as shown in Figure 5D, that models utilizing features not requiring quantum chemistry (QC) calculations (non-QC features) outperform those relying on QC-derived features in predicting ΔE_{ad}(CO) of layered alloys.[158] However, an exception occurs when the feature vector is one-dimensional; in this case, models leveraging QC features, particularly the d-band center, demonstrate superior performance due to its strong correlation with adsorption energy.

Model selection is key in ML-aided catalyst design. Classical ML models are suitable for limited data, while neural-network (NN)-based models are better for large datasets. Blending ML models can improve performance. Ulissi and his coworkers utilized the tree-based pipeline optimization tool (TPOT) to

construct composite models for accurate prediction of ΔE_{ad} on intermetallic compounds with various facets.[163,164] The root-mean-square error (RMSE) for ΔE_{ad}(CO) prediction was 0.46 eV and the RMSE for ΔE_{ad}(H) prediction was 0.41 eV. Amaral et al.[181] constructed ensemble models of three ML models (support vector regression [SVR], model, NN-based model, and Extreme Gradient Boosting Regressor [XGBR] model) and stacked a meta-model on them to balance the variance and bias of the prediction results, enhancing accuracy in ΔE_{ad}(CO) and ΔE_{ad}(CO_2) prediction.

Crystal graph convolutional neural network (CGCNN) and other graph-based variant models like labeled-site CGCNN (LS-CGCNN) have shown remarkable accuracy for ΔE_{ad} prediction (ΔE_{ad}(CO) MAE < 0.13 eV, and ΔE_{ad}(H) prediction MAE <0.1 eV).[182–184] These models convert catalyst surfaces into graphic-based data according to the constituent atom positions and their connectivity. These graphic types of data can delineate information of the local environment information effectively into their nodes and edges, which alleviates the requirement of feature engineering. However, careful selection of ML models is essential, as advanced models do not always guarantee enhanced performance. Mok et al. employed the GBR model, a classical ML approach, to predict ΔE_{ad} using surface microstructure-embedded input features that do not require DFT calculation, still maintaining comparable accuracy with much less training data.[179] This implies model selection should be context-optimized.

Challenges in ML-aided ECR catalyst screening and outlook. ML-aided screening has gained prominence, yet several challenges should be addressed, including data scarcity, insufficient catalytic performance descriptors, and the gap between computation and experimentation.

One of the main challenges in harnessing ML models is securing a database with both sufficient quantity and high quality. High quality means consistency within the data-generation procedure and unbiased distribution of data within chemical space. In this context, pre-established databases in surface chemistry, such as the datasets from the Open Catalyst Project (OC20,[185] OC22,[186] ODAC23,[187] OC20-Dense,[188] OC20NEB,[189] and OMat24[190]) and GASpy,[163] facilitate ML-aided research. Comprehensive datasets from computation can complement success bias often present in experimental data, where records of failures are frequently absent. Including failure data is essential for robust ML models. However, the specificity of heterogeneous catalytic systems often limits database utilization, necessitating efficient use of limited data or data augmentation through accelerated computational processes.

Active learning (AL) effectively trains ML models with minimal data by selecting informative data with a greedy selection algorithm that will be validated by human intervention and utilized to update and retrain the model. This iterative process optimizes the search trajectory in chemical space. The success of AL in ECR catalyst design includes Ulissi et al.'s exploration of over ~1.7 million distinct binding sites of 1,499 bimetallics.[163,164] They selected ~20,000 binding sites for each of the CO and H adsorptions by searching a surface with a near-optimal ΔE_{ad}, which is a very small selection considering the size of the search space. AL also demonstrated its viability in smaller chemical spaces, achieving a 70% success rate in discovering HCOOH-selective dual-metal-site catalysts (DMSCs) from a dataset containing 282 DMSCs over three iterations.[174]

Transfer learning (TL) addresses data scarcity challenge by transferring knowledge efficiently from the source domain to the target domain, enhancing performance through fine-tuning the parameters of the model trained in the source domain. Although the TL framework has not been fully exploited in ML-aided ECR catalyst screening for liquid fuels, a proof-of-concept study employed TL with a pre-trained model for the prediction of ΔE_{ad}(CO).[191] Discussions on transferability include applying reaction energy-prediction models trained on metal-zeolite systems to metal-organic frameworks (MOFs), 2D materials, and molecular complex systems[192] and applying a ΔE_{ad} prediction model trained for Cu-based binary alloys to tertiary alloys.[193] Since the success of TL is largely dependent on relevancy between the domains, the discussions on the transferability imply the potential of applying TL in designing ECR catalysts to produce liquid fuels.

ML potential (MLP) provides a computationally efficient approach to predict the total energy and atomic forces of a system without solving the Kohn-Sham equation,[194] significantly accelerating various simulations like geometry optimization, NEB calculation, and MD simulation.[189,195] By overcoming DFT's limited scalability, MLP facilitates exploring vast chemical spaces for complex catalyst systems.[196] For instance, minimum energy paths (MEPs) for the conversion of CO_2 to ethanol were identified on several types of metal oxide surfaces, including surfaces with oxygen vacancies, reduced metal oxides, and doped metal oxides.[197] The high computational efficiency of MLP enables the examination of multiple MEPs for each reaction step, ultimately leading to the identification of an alternative path with a 40% reduced activation energy. MLP also helps identify catalyst surface structures and active sites,[188,198–201] as shown by Lan et al.'s graph-based MLP models that reduce computational load significantly.[188] State-of-the-art MLP models,[202–204] pre-trained on OC20 datasets, exhibits comparable or enhanced accuracy compared to heuristics-based DFT calculations with reduced computational load by orders of magnitude. This result underscores the potential of MLP to accelerate data generation while retaining reliability.

Selecting suitable electrocatalytic descriptors, beyond ΔE_{ad}(CO), is crucial for ECR catalyst screening. While ΔE_{ad}(CO) is a common ECR activity descriptor,[205] its limitation as the sole descriptor has been noted.[159,160,178] Recent ML models predict various activity descriptors, such as ΔE_{ad} of adsorbates (i.e., CH_nO (n = 1–3), COOH, HCOO, COCOH, etc.),[135,149,177,181,192] CO dimerization energy and CO hydrogenation energy,[168] and the limiting potential of the reaction.[165,174] The difference between predicted limiting potentials of products or between predicted ΔE_{ad} of intermediates has been employed as a measure of selectivity. Also, descriptors for selectivity and stability have been suggested. For example, product probability on zeolite was predicted to determine product selectivity between CH_3OH and CH_4.[192] Also, it was reported that the difference between binding energy and cohesive energy of a metal at the active center of SACs is a stability descriptor.[165]

Bridging the gap between computational screening and experimental application remains challenging. Exclusive reliance on computational data fails to capture the role of operating conditions (synthesis, type of cell, membrane, electrolyte, applied potential, and morphological change).[206] Accelerated by ML, multi-scale modeling techniques such as microkinetic modeling or MD enable the extrapolation of atomistic-level insights to a more practical, application-oriented scale, as has been comprehensively outlined.[207] Additionally, incorporating experimental data offers a complementary approach. There have been attempts to leverage experimental data from literature or databases to train ML models and predict catalytic performance, including the ECR FE and rate.[208,209] The integration of computational and experimental data can be facilitated by autonomous laboratories and high-throughput experimentation combined with ML.[210–213]

ML's support in computational screening is shown by the list of candidate catalysts suggested in Table S6, which could be of interest for experimental investigation. It should be noted that some ML studies did not specify the type of target products being pursued. However, since they adopted the property of Cu, which is known to reduce CO_2 to various multi-C products, as a benchmarking material, we present their screening results (Table S6).

STRATEGIES FOR TAILORING CO_2 REDUCTION PATHWAYS TOWARD LIQUID PRODUCTS

Modification of catalysts

Surface functionalization. Surface modification plays multiple roles in regulating ECR performance. It can affect mass transfer of reactants by modifying hydrophobic groups on a catalyst surface to prevent proton migration to the active sites or by modifying functional moieties to promote CO_2 adsorption.[214] It may also change the intrinsic activity of metal catalysts, such as by reducing the activation energy of the rate-determining step of the reaction through modification of the active sites, alteration of the surface chemical state, or changing the configuration of intermediates on the catalyst surface.[215] Thirdly, the species used for surface modification may confer potent new active sites to enhance the ECR catalytic activity.[216] Fourthly, incorporation of modifiers in the surface of catalysts can enhance their stability toward leaching, aggregation, or rapid reduction during ECR.

The cleavage of the $CH_2 = CHO^*$ intermediate, connected to Cu via a Cu–O–C bond, determines the production of C_2H_4 or C_2H_5OH. Modifying the Cu surface with methyl benzenesulfonyl azide is beneficial for constructing an electron-delocalized state on the Cu surface, facilitating the delocalization of electrons from Cu to the methyl benzenesulfonyl azide molecule (Figure 6A).[217] This promotes the cleavage of the Cu–O bond, leading to the formation of C_2H_5OH.

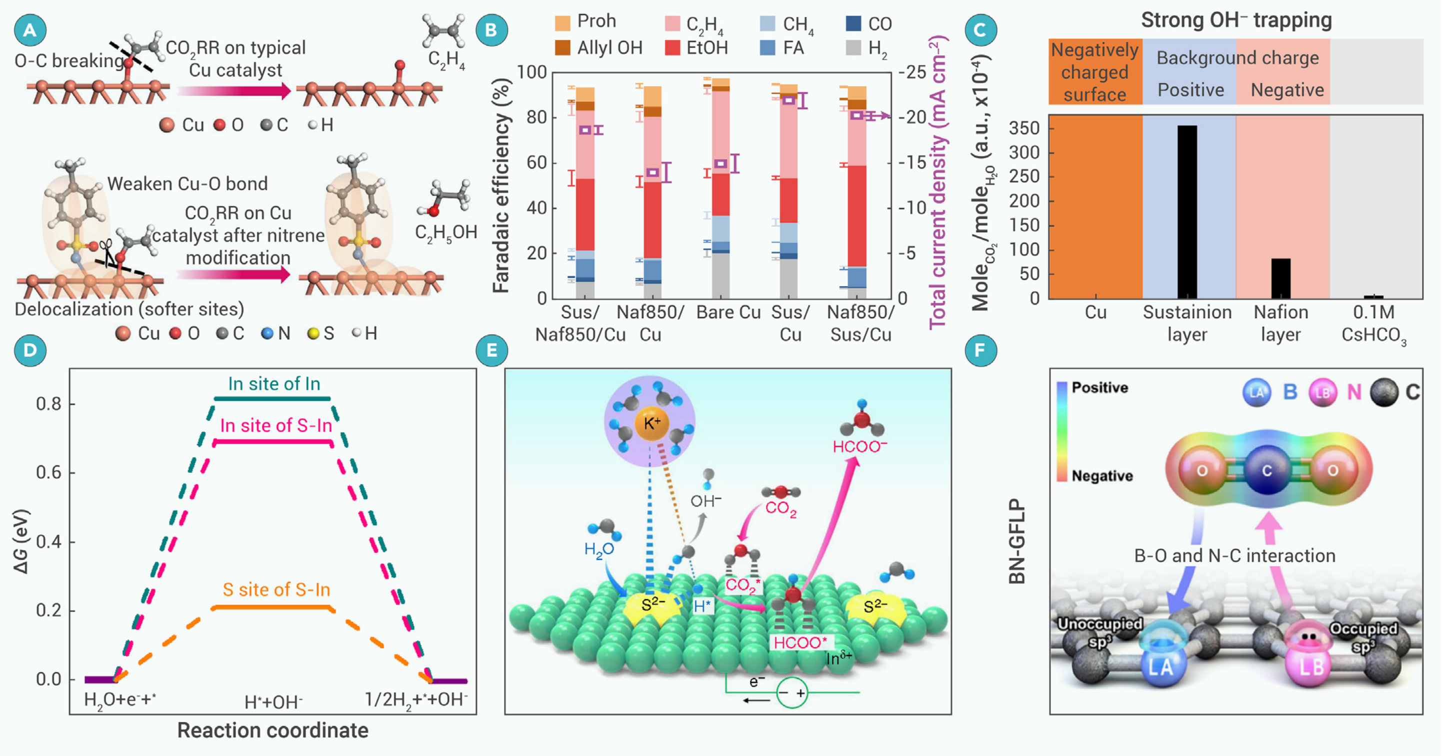

Figure 6. Surface functionalization, heteroatom engineering strategies for ECR to liquid products (A) Schematic illustration of the adsorbed CH_2=CHO* intermediate on a Cu surface, as the bifurcation point toward the ethylene or ethanol pathway during ECR. On a typical Cu surface, the O–C bond energy is lower than that of the O–Cu bond and easier to cleave, thus producing ethylene. On a nitrene-functionalized Cu surface, the electron delocalization makes the Cu site a "softer" acid and thus reduces the O–Cu bond energy, so the O–Cu bond is easier to cleave than the O–C bond and the selectivity is switched to produce ethanol.[217] Copyright 2024, American Chemical Society. (B) ECR performance at −1.15 V vs. RHE using stacked ionomers on Cu in the presence of 0.1 M $CsHCO_3$ electrolyte.[218] Copyright 2021, Springer Nature. (C) Schematic illustration of Naf850/Sus/Cu and Sus/Naf850/Cu in terms of local CO_2/H_2O ratio and spatial charge configuration. Areas shaded orange, blue, red, and gray correspond to Cu, Sus., Naf., and electrolyte solution, respectively.[218] Copyright 2021, Springer Nature. (D) Gibbs free energies for the formation of H* on pure In(101), In, and S sites of S–In(101) surfaces.[219] Copyright 2019, Springer Nature. (E) Schematic illustration for the role of S^{2-} in promoting water dissociation and H* formation for the reduction of CO_2 to formate.[219] Copyright 2019, Springer Nature. (F) Schematic of molecular interactions between BN-GFLP and CO_2.[220] Copyright 2024, American Chemical Society.

Methyl benzenesulfonyl azide-functionalized Cu (Cu=N) achieved an FE_{C2H5OH} of 45% at −0.82 V (vs. RHE), which is 3.2 times that of bare Cu.

The microenvironments of Cu-catalyzed CO_2 electrolysis can be tailored for C_2H_5OH selectivity by introducing functional layers. Modifying Cu with metal hydroxide of Pourbaix stability (e.g., $Ce(OH)_x$) facilitates water dissociation and hydrogen adsorption (H_{ad}), but it is unlikely to impact the adsorption of carbonaceous intermediates.[29] The H_{ad} attacks *HCCOH, forming *HCCHOH toward C_2H_5OH instead of C_2H_4. Coating Cu with ion-conducting polymers (ionomers) such as Naf. (a perfluorosulfonic acid, cation-conducting ionomer) and Sustainion (Sus., a polystyrene vinylbenzyl methylimidazolium, anion-conducting ionomer) was demonstrated to create favorable microenvironments (higher local CO_2/H_2O ratio and pH) for selective C_2H_5OH and C_{2+} production.[218] The surface roughness of Cu remains unaffected, and the electrochemical surface areas for bare Cu and all ionomer-coated Cu samples were close. The partial current density for ECR increases with the local CO_2/H_2O ratio following the order of bare Cu < Naf.1100 (Naf. with 1,100 g mol_{eq}^{-1} equivalent weight)/Cu < Sus./Cu (Figure 6B). The enhanced C_{2+} selectivity (including C_2H_5OH and $C_2H_5CH_2OH$) for Naf.1100/Cu and the further enhancement for Naf.850/Cu are likely due to a Donnan exclusion at the interface between the Cu and the ionomer. Naf.850 relative to Naf.1100 possesses a higher background charge density and stronger Donnan exclusion, leading to more local accumulated OH^- anions and increased C_{2+} selectivity. An optimized coating with Naf. as the outermost layer followed by Sus. (i.e., Naf.850/Sus./Cu) provides the best C_{2+} FE, originating from its high local ratio of CO_2/H_2O, greater amounts of OH^-, and hindrance of HCO_3^- transport from the bulk electrolyte to the reaction surface (Figure 6C).

Graphene nanodisks with oxygen-containing groups (–COOH, –OH, C–O–C, and –C=O) facilitated $HCOO^-$ yield with an FE of 90% at −0.68 V (vs. RHE).[221] The C atoms adjacent to these moieties were surmised to be the active center where the main species contributing to the activity is the carboxyl group. In another work, modification of N-doped CNTs with polyethylenimine (PEI) enhanced the FE toward $HCOO^-$ from 59% with a partial current density of −3.0 mA cm^{-2} to 85% with a partial current density of −7.2 mA cm^{-2} at a potential of −1.13 V (vs. RHE).[222] The N atoms in PEI were hypothesized to stabilize the singly reduced intermediate CO_2^- via the formation of NCNT–N–C(O)O* … H–N–PEI, with the PEI coating concentrating CO_2 near the electrode surface and boosting the reaction rate.

Surface modification also protects active sites from deterioration during CO_2 electrolysis. For example, the reduction-induced deactivation of cobalt phthalocyanine (CoPc) during ECR was alleviated by introducing four amino groups at the β sites of the phthalocyanine ligand.[223] The electron-donating $-NH_2$ substituents lowered the reduction potential of CoPc. in contrast to pristine CoPc dispersed on C nanotubes (CNTs), which yielded a CH_3OH FE of only 0.6% after 5 h of electrolysis. The modified CoPc catalyst maintained good stability with minor degradation in CH_3OH FE after 12 h of reaction. Coating Cu with poly(4-vinyl pyridine) (P4VP/Cu) increases the electrode robustness with the pyridyl groups in P4VP bound to the Cu sites, screening the Cu against leaching into the electrolyte.[224] As a consequence, $HCOO^-$ production by P4VP/Cu was sustained for 30 h, exceeding the prior performance reported for Cu, Sn, and Bi electrodes. Likewise, depositing a C layer over a CuO_x surface was found to stabilize Cu^+ species, leading to a C_2H_5OH FE of 46% with a partial current density of −166 mA cm^{-2}.[225]

In addition, since electrolytes containing alkali metal cations can induce carbonate deposition on the cathode and block the CO_2 mass transfer channel, the conversion of CO_2 to HCOOH in acidic electrolyte without alkali metal cations can be achieved by loading polyelectrolyte on the surface of catalysts. Cross-linked poly(diallyldimethylammonium chloride) (c-PDDA)-modified In NPs achieve 75% FE_{HCOOH} at −1.84 V (vs. SHE) in 0.1 M H_2SO_4.[226] The cation sites carried by the polymer layer play a similar role to that of the alkali cations dissolved in the acidic electrolyte in enabling CO_2 reduction (that is, inhibiting the migration of H and enhancing the electric field of the Stern layer).[227,228]

For surface functionalization, two issues need to be considered. Some modified systems exhibit lower current densities than bare Cu references, suggesting unwanted blocking of active sites by the modifiers. Deconvoluting whether a

modified catalyst possesses better intrinsic activity than the pristine catalyst requires metrics such as the partial current density normalized against ECSA and the turnover frequency (TOF).[229]

Heteroatom engineering. Introducing heteroatoms into a catalyst can regulate product formation by adjusting the electronic structure of the catalysts and/or the adsorption dynamics of the intermediates. For example, Co-doped Bi nanosheets achieved ~90% selectivity for $HCOO^-$ at a current density of −200 mA cm^{-2} and a low overpotential of ~1.0 V. Compared to bare Bi, Co-doped Bi has a stronger binding energy for the key intermediate OCHO*, thus requiring a lower applied potential for the conversion of CO_2 to HCOOH.[230] Replacing O in Sn-based oxides with less electronegative chalcogens S and Se to form SnS_x[231] and $SnSe_2$[232] increases the electronic conductivity, improves CO_2 adsorption, and decreases *OCOH adsorption energy, thus boosting $HCOO^-$ production. S doping may also hamper hydrogen or water adsorption (Figure 6D). S-doped In (S-In) catalyst was reported to enhance the $HCOO^-$ yield, outperforming bare In, Se-In, and Te-In catalysts (Figure 6E).[219] To address the over-reduction of S dopants at large overpotentials (and a resulting rapid increase in HER), phase engineering of a SnS (π-SnS) pre-catalyst was demonstrated to stabilize S dopants in the derived metallic Sn for selective acidic CO_2-to-HCOOH electrolysis at −1 A cm^{-2}.[233] S, being more electronegative than Cu, withdraws electrons from Cu, allowing the negatively charged S atom to adsorb a CO_2 molecule or a hydride. The adsorbed CO_2 is selectively reduced to $HCOO^-$ on the neighboring Cu site through a protonation of the C atom or alternatively through a *H pathway.[234]

Metal-like doping of Cu with boron (B) and phosphorus (P) stabilizes *CO and alters the reactivity and selectivity for $C_2H_5CH_2OH$ production.[235] B doping promotes C_{2+} products, while P doping leads to faster *CO consumption. B doping reduces the thermodynamic energy barrier in the CO hydrogenation reaction, thereby improving the selectivity of C_2H_5OH on Cu(111).[236] Electrons transfer from Cu to B and gather around the B atom, which acts as the charge-transfer medium. The intermediate isomerization step puts C in a reverse charge state, making C−C coupling and C_2H_5OH formation more favorable. As opposed to doping with S, P, and O, an N-engineered Cu catalyst was demonstrated to exhibit the best CO_2-to-C_{2+} productivity with ampere-level current.[237] Engineering with N atoms promoted *CO adsorption on both the bridge and atop sites of Cu, lowering the energy barrier for C−C coupling. Additionally, B and N co-doping can construct surface-frustrated Lewis pairs (FLPs), with B and N serving as Lewis acid and Lewis base sites, respectively (Figure 6F).[220] The lone-pair electrons of the O atom in CO_2 can be donated to the surface Lewis acid site, while the C atom in CO_2 can receive electrons from the Lewis base site. The synergistic effect of FLPs reduces the free energy for CO_2 activation and C−C coupling. B and N co-doped graphene achieved an FE of 87.9% for C_{2+} products at the potential of −0.7 V (vs. RHE), with an FE of 50.4% for *n*-propanol. In contrast, B-doped graphene and N-doped graphene only produced formate.

Design of bi(multi-)metallics. Bi(multi-)metallic catalysts can enhance performance relative to their monometallic counterparts via electronic effects (including the ionic ligand effect, covalent ligand effect, and strain effect) and geometric effects (including the ensemble effect, ordering effect, and steric effect). These effects usually function concurrently to facilitate adsorption and catalysis. Addition of another element can alter the electronic structure, thereby influencing the binding affinity of adsorbed intermediates. Bi(multi-)metallics may break the scaling relationships among adsorbates to lower the overpotential. The atomic number and/or configuration of active sites may also be changed, which varies the nature of adsorption of the intermediates at the surface. In addition, adjacent metallic sites can impart distinct catalytic roles, enabling synergistic activity (sequential catalysis on bi(multi-)metallics is discussed in the following section). Incorporating a metal that has a higher oxophilicity and strong M−Cu bonding energy (e.g., early 3*d* metals such as Sc, Ti and *p*-group metals such as Al, In, and Bi) tends to stabilize Cu against reconstruction during the ECR.[238]

Bimetallic Cu-M (M refers to *p*-block metals such as Bi,[239] Sn,[240] and Pb[241]) have been reported to enhance HCOOH formation. For example, the addition of Bi in Pd to form intermetallic Pd_3Bi prevents the overbinding of *H or *CO on Pd and avoids CO poisoning, substantially lowering the *CO adsorption energy from −1.11 to −0.1 eV,[239] leading to selective reduction of CO_2 to HCOOH with an FE approaching 100%. Incorporation of Ag in Cu can induce compressive strains on Cu surface atoms, shifting the valence band structure of Cu to deeper levels, dampening *H adsorption and thereby lowering the HER activity,[242] while the FE for multi-C oxygenates (mainly CH_3COO^- and CH_3CHO) was significantly improved. This was ascribed to the lowered rates of C−O bond cleavage as a result of HER inhibition and the depressed oxophilicity of the compressively strained Cu, which weakens the deep reduction of the carbonyl-containing oxygenates. Using $Pd_{83}Cu_{17}$ alloy electrocatalyst and 1-butyl-3-methylimidazolium tetrafluoroborate ($[Bmim]BF_4$) aqueous solution catholyte promotes the selective formation of CH_3OH via ECR with an FE up to 80%,[243] whereas the catalyst is inactive for ECR and produces predominantly H_2 in 0.5 M $NaHCO_3$ and Na_2SO_4 aqueous solutions. In a 0.5 M $KHCO_3$ aqueous solution, an impressive CH_3OH FE of ~77.3% at a low reduction potential of −0.3 V (vs. RHE) was achieved on $CuGa_2$.[244] It was hypothesized that the adsorbed H at the Cu site can spill over to the Ga site and then further react with the adsorbed *OCHO to produce CH_3OH.

The emerging area of high-entropy alloys (HEAs), which confine multiple atomic species into the same lattice, can provide multi-center active site cooperation. Compositional and structural engineering of HEAs enables optimization of catalytic properties. As an example, a PdCuAuAgBiIn HEA aerogel (Figure S1A) was reported to be active for ECR to yield HCOOH with an FE of 98.1% at −1.1 V (vs. RHE).[226] The good activity was likely from the interactions between the different metals and the surface unsaturated sites, optimizing HCOO* intermediate adsorption/desorption for enhanced HCOOH production.

Construction of tandem/cascade architectures. In recent studies, cascade catalysis has enhanced the selectivity of C_{2+} liquid products. In a typical tandem catalytic system, a mainly CO-producing metal-based catalyst (e.g., Au,[245] Ag,[246] Pd,[247] Zn,[248] and Fe-porphyrin[112]) is coupled with Cu, allowing *CO or CO molecules[249] formed by the ECR to be transferred and reduced on the Cu to generate high-order hydrocarbons and oxygenates (Figure S1B). Increasing the density of CO-selective sites on Cu improves *CO coverage at the expense of *H, expediting C−C coupling reactions and favoring oxygenate yield over hydrocarbons. On a tandem electrode (e.g., Cu_xZn and Ag-Cu_2O) with low hydrogen-generation rates and small overpotential regions (e.g., Au/Cu), the selectivity of oxygenated C−C coupled products (i.e., C_2H_5OH) is promoted over hydrogenated C−C coupled products (i.e., C_2H_4).[250] Another report demonstrated that C_2H_5OH is selectively enhanced through the coupling of $*CH_x$ and *CO at the terraces of Cu(111)-Ag in the CO-enriched environment provided by the Ag spheres.[251] Distinctively, on Cu(100)-Ag, C_2H_5OH is selectively generated following the same pathway at the edges and corners of Cu(100), while C_2H_4 is produced at the terraces.

Another type of tandem system is Cu(II)- and Bi(III)-based MOFs, which afford a C_2H_5OH FE of 28.3%.[252] The improved C_2H_5OH formation can be explained by the reduction of CO_2 at Bi to $HCOO^-$, which is subsequently transported to Cu and further reduced to alcohol. However, the poor stability of the reported electrodes remains an issue.

Tandem catalysis has also been achieved using with interdigitated Au and Cu microfabricated electrodes on an insulating SiO_2 substrate.[253] CO formed on the Au lines can be further reduced by the Cu lines. Reducing Cu coverage shifts the C_{2+} product distribution to oxygenates over C_2H_4. Alternatively, sequential catalysis can be achieved by combining two tandem electrolytic cells[127] that convert CO_2 to CO and CO to $C_2H_5CH_2OH$, respectively.

A cascade system integrating CO_2 reduction and CO reduction steps enables high-efficiency production of $C_2H_5CH_2OH$ through the precise voltage control of the CO_2 electrolyzer system. The FE toward $C_2H_5CH_2OH$ reached 15.9% with a partial current density of −20 mA cm^{-2} and half-cell energy conversion efficiency up to 19.3%.[254] A medium-temperature solid-oxide electrolysis cell avoids carbonate formation ($2OH^- + CO_2 \rightarrow CO_3^{2-} + H_2O$) and achieves a reasonable single-pass conversion (i.e., the percentage of C that is transformed to reduction products in a single pass-through electrolyzer) during the CO_2-to-CO conversion step (Figure S1C).[255] A cascade approach was also demonstrated for HOC_2H_4OH production using CO_2 instead of fossil-fuel-derived C_2H_4 as the feedstock with a production rate of 0.5 mmol h^{-1}.[256]

Defect regulation. Vacancies and low-coordination sites enhance electron capture and accessibility of selective sites.[257] Oxygen-vacancy-rich In_2O_3 exhibited better HCOOH production performance during ECR than its less defective counterpart, as oxygen vacancies reduced the barrier for the formation of *COOH intermediates.[258] The introduction of S vacancies in In_4SnS_8 boosted HCOOH production with an FE of 91% at −1.0 V (vs. RHE) (Figure S1D),[259] facilitating CO_2 adsorption and favoring the *HCOO-mediated HOOH pathway while suppressing the competing HER (Figure S1E). Relatedly, the creation of S vacancies

in a Cu_2S_{1-x} electrocatalyst afforded enhanced C_2H_5OH FE of 73.3% at a low overpotential of 0.19 V,[260] with DFT calculations showing strong electron-donating ability of abundant $Cu^{\delta+}$ catalyst surface sites, which reduce the energy barrier of the C−C coupling step to produce C_2H_5OH. Alternatively, the presence of surface Cu vacancies in a Cu_2S−Cu core-shell nanostructure was shown to increase the energy barrier toward C_2H_4 formation, whereas the C_2H_5OH pathway was unaffected, thus boosting the selectivity of C_2H_5OH formation.[43]

Lattice dislocations can improve conductivity, create more active sites, and regulate the adsorption energetics of reactants and intermediates.[261] A Bi nanowire catalyst with lattice dislocation on copper foam showed a 95% FE for HCOOH production at −0.69 V (vs. RHE).[262] There are more undercoordinated step sites on metal catalysts with high dislocation density, and the energy barrier for the formation of CO_2 reduction intermediates is lower.[263] Grain boundaries formed by adjacent nanocrystals are also important active sites that promote electrocatalytic CO_2 reduction by regulating the binding energy of reaction intermediates.[264]

Selective facet exposure. Some facets may break the adsorption-energy scaling relations, thus promoting activation and reduction of CO_2 and key intermediates. Designing materials with tailored crystal facets is a rational strategy to regulate product selectivity. Wei et al.[159] tailored the growth orientation of the crystal facets of an In-Cu electrocatalyst by controlling the In/Cu ratio. Only the In(101) facet in the $In_{1.5}Cu_{0.5}$ catalyst effectively stabilized the *OCHO intermediate, which was favorable for HCOOH formation. The exposed (111) facet on Bi monolayers was observed to have a lower CO_2 reduction overpotential (0.58 V) than the HER (1.28 V) and facilitates HCOOH formation through an *OCHO intermediate, while the (011) facet of thick Bi layers binds the intermediate too strongly, thus poisoning the catalyst.[265]

On Cu electrodes, ECR shows a strong facet dependence for the formed product, which can be described according to the binding energies of O*, H*, C*, OH*, CO*, OCCOH*, and COOH*.[266] Cu(111) favors CH_4 and $HCOO^-$, while Cu(100) favors C_2H_4 formation. The (111) steps on (100) facets are mainly selective to C_2H_4, whereas the (110) steps on (100) planes are more inclined to generate C_2H_5OH.

Cu(110) enables secondary C_2 products such as CH_3COO^-, CH_3CHO, and C_2H_5OH.[267] The (111)−(100) motif in periodic face-centered cubic Cu is proposed as an active ensemble for promoting C_{2+} yield due to its favorable formation energy for *OCCOH.[268] Among the four planar-square (p-sq), step-square (s-sq), concave-square (cc-sq), and convex-square (cv-sq) facets that were extracted as slab models to calculate the energetics of *CO coupling over oxide-derived Cu by DFT, the s-sq provided the strongest affinity to bind $*CH_2CHO$ for further protonation to C_2H_5OH.[269] The n(100) × (110) steps have been also identified as active sites for C_2H_5OH production.[266] Wrinkled Cu films with exposed (310) facets were shown to deliver impressive C_2H_5OH selectivity with an FE reaching 40% at −0.9 V (vs. RHE).[270] Such high selective production of C_2H_5OH was calculated to be due to the high density of step sites with abundant (310) facets, which possess a low C−C coupling energy penalty (0.5 eV) toward C_2H_5OH formation.

Surface oxidation state modulation. A metastable oxide/hydroxide layer on Sn[271] and Pb[272] electrodes enhances $HCOO^-$ formation, whereas $RuO_x \cdot IrO_x$ and RuO_x/IrO_x modified with Cu and Cd adatoms produced $(CH_3)_2CHO$ and CH_3OH.[273] Theoretical calculations suggest that the major intermediate on transition metal oxides (TMO) is *OCHO (for HCOOH formation) instead of *COOH (for CO formation). At regions with *OH adsorption energies lower than −0.34 eV, CH_3OH is preferably generated on TMOs, whereas HCOOH is favored at *OH binding energies higher than −0.21 eV.[274] Among the various tin oxides, Sn_3O_4 was reported to deliver the highest HCOOH FE of 97.7% at −0.9 V (vs. RHE).[275] The superior activity was attributed to the collaborative effect of Sn^{2+} and Sn^{4+}, which bind the intermediates weakly and strongly, respectively.

The thermodynamics and kinetics of the CO_2 reduction and *CO dimerization on Cu were calculated to be greatly enhanced in the presence of subsurface oxygen through orbital overlapping between oxygen from Cu and CO_2 molecules.[276] As opposed to Cu^{2+}, which produces mainly CH_4,[277] a synergy between Cu^+ and Cu^0 (with an optimal Cu^0-to-Cu^+ surface ratio of ∼0.5) can accelerate C_{2+} formation (e.g., C_2H_5OH).[278] Cu^0 promotes CO_2 activation by lowering the thermodynamic energy barrier, while Cu^+ strengthens *CO binding, thereby boosting C−C coupling. In another study by Arán-Ais et al.[279] Cu^+-Cu^0 pairs and defects were shown to enable higher C_2H_5OH production. When the reduction of Cu(I) is minimized at 278.15 K during pulsed ECR, C_2H_5OH dominates the C_2 products. Alternatively, it has been suggested that strongly bound bridge-adsorbed CO (CO_B) occurs on low-coordination Cu^0, while a Cu^+ site binds linearly to adsorbed CO (CO_L).[280] The adjacent CO_B and CO_L are readily coupled to form C_{2+} products. From these scenarios, strategies such as oxygen plasma treatment,[281] elemental doping,[246] anodic pulse,[282] and engineering of mixed metal oxide interfaces[283] have been developed to create and stabilize $Cu^{\delta+}$. Selection of some supports that possess rapid electron transport channels has been postulated to hinder the accumulation of electrons around $Cu^{\delta+}$ sites, thus protecting them against electrochemical reduction.[277] An alternative avenue is to re-oxidize lower-valence-state species back to the original higher valence state through pulsed electrolysis.[282] However, how to maintain the mixed oxide states during prolonged operation and under aggressively reducing conditions still remains an issue. Recently, He et al.[284] reported the stable existence of Cu_2O-Cu^0 catalysts during ECR, where Cu^+ formation was speculated to be due to *in situ* reoxidation (Figure 7A) from the small amount of O_2 generated from the oxidation of the anode H_2O passing through the membrane to the cathode. The high concentration of OH^- on the surface of the catalyst might also promote Cu^0 reoxidation.

Nanoconfinement control. Nanoconfinement describes confining a reaction within nanosized regions, with dimensions of less than 100 nm, to break the linear scaling limitation in electrocatalysis.[289] In nanoconfined spaces, the transport of HCO_3^- (into) and generated OH^- is hindered, resulting in higher local pH values compared to the bulk solutions. This suppresses the HER while lowering the C−C coupling energy barrier and facilitating the reduction of the *CO intermediate to C_{2+} products (including C_2H_5OH and C_3H_7OH).

Creation of porosity and/or cavities can prolong intermediate residence time and speed up the reactions of adsorbed CO_2 and intermediates to create C_{2+} hydrocarbon and alcohol products (Figure 7B).[290] Construction of surface nanocavities can stabilize Cu^+ and prevent its reduction under ECR reaction conditions, thereby enhancing C_{2+} production.[291] Alteration of the size of the nanoconfined region can influence the reaction kinetics by causing strain as a result of changes in curvature and coordination number,[292] thus impacting the binding strength of reactants. Of note is that nanoconfinement is most effective when the reaction is limited by electron-transfer kinetics rather than mass transport. Alternatively, the effectiveness of nanoconfinement can be influenced by the adsorption affinity of reactants or intermediates. Reactants that bind weakly tend to collide more frequently with active sites within confined regions, resulting in a more significant nanoconfinement effect compared to those that bind strongly. In addition to surface coverage of *CO intermediate, the configuration of adsorbed *CO can be tuned to improve selectivity toward C_{2+} alcohols through nanoconfinement.[286] Ordered mesoporous CuO was found to favor bridged adsorption of *CO, while cylindrical CuO preferred a top adsorption configuration. Bridge-adsorbed *CO species were inclined to undergo deep protonation to form $*OCH_3$, which promoted its further coupling with *CO to yield C_2H_5OH and $C_2H_5CH_2OH$. This contrasted with top adsorbed *CO, which tended to couple with the formed *COH to generate C_2H_4 (Figure 7C).

To understand nanoconfinement's impact, designing well-defined nanoconfined systems with controlled size, distribution, and hierarchy of pores, cavities, and channels as well as degree of freedom is crucial. This, in turn, can guide the development of high-performing ECR electrocatalysts toward oxygenated liquid compounds. A Cu catalyst with a wrinkle structure, tunable in shape and dimension (in terms of shrinkage degree [i.e., areal strain, ϵ] and Cu film thickness [h_{Cu}]) was fabricated and investigated for ECR.[287] Increasing ϵ enabled variation of the wrinkle from an open (non-folded, $\epsilon < \sim 0.6$) to a confined (folded, $\epsilon > \sim 0.6$) structure. Manipulation of h_{Cu} and ϵ enabled tuning of the dimensions of the wrinkles (i.e., wavelength and depth). A pronounced increase in C_2H_4 FE was observed with the increase of confinement degree for the non-folded shape (i.e., ϵ from 0.25 to 0.50), while there was no such change for CH_4 FE and C_2H_5OH FE. As the wrinkle transformed from a non-folded shape to a folded structure (with ϵ elevated from 0.50 to 0.75), the C_2H_5OH FE increased markedly from 14.3% to 27% at 1.3 V (vs. RHE) in contrast to a notable decrease in CH_4 FE from 21.0% to 8.0% and an unchanged C_2H_4 FE. The C−C coupling was enhanced in more strained wrinkles, maximizing in folded wrinkles regardless of h_{Cu}. The folded wrinkle geometry limited the mass transfer of C_1 intermediates and OH^- ions, especially favoring C_2H_5OH generation (Figure 7D). The C_2H_5OH FE was further improved to 41.9% on a folded wrinkle using 0.1 M $KClO_4$

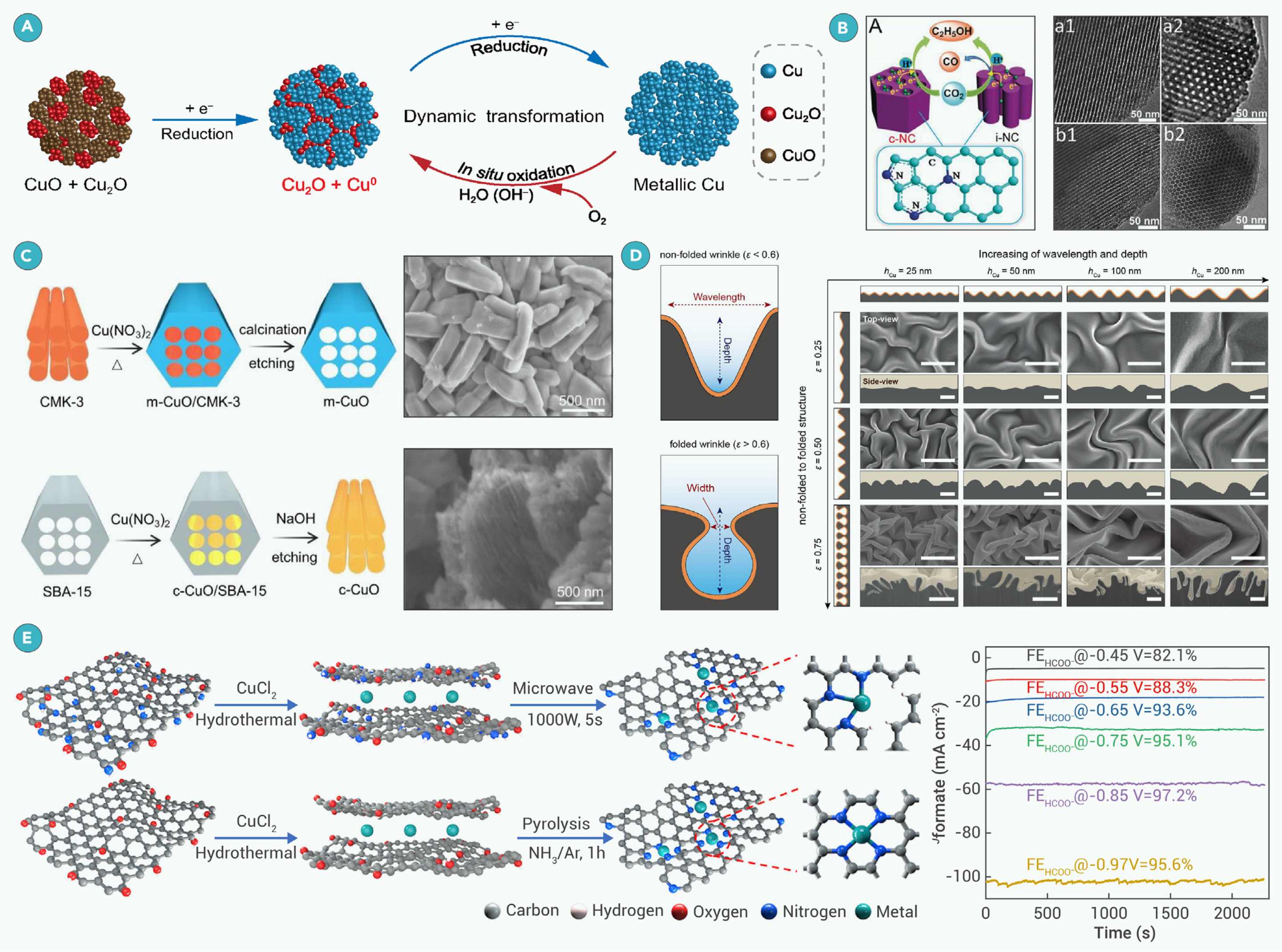

Figure 7. Oxidation state modulation, nanoconfinement control and coordination engineering strategies for ECR to liquid products (A) Schematic diagram of dynamic reduction and re-oxidation mechanism of copper for the Cu_2O-Cu^0 catalyst during ECR.[284] Copyright 2024, Springer Nature. (B) Illustration of cylindrical mesoporous N-doped C (denoted as c-NC) and inverse mesoporous N-doped C (denoted as i-NC) for CO_2 electroreduction. Transmission electron microscopy (TEM) images of (a1, a2) c-NC and (b1, b2) i-NC viewed along (a1, b1) [110] and (a2, b2) [100] directions.[285] Copyright 2017, Wiley-VCH GmbH. (C) Schematic illustration of the template routes to *m*-CuO and c-CuO catalysts and SEM and TEM images.[286] Copyright 2024, Wiley-VCH GmbH. (D) Feature shape and dimension of Cu wrinkle model.[287] Copyright 2023, Wiley-VCH GmbH. (E) Schematic illustration of the preparation strategy for PSB-CuN_3 and PS-CuN_4 and chronoamperometric responses of PSB-CuN_3 in CO_2-saturated electrolyte at different potentials. The potential was corrected with 100% *iR* compensation.[288] Copyright 2023, Springer Nature.

solutions as a catholyte (without buffering ability, thus leading to high local pH values).

Coordination engineering. C-supported SACs mostly possess a quasi-planar D_{4h} local symmetry with a MN_4 coordination structure, which limits modulation of the metal sites' electronic configuration.[293,294] By disrupting the quasi-planar D_{4h} local symmetry through coordination engineering, the electrons in specific *d*/*s*/*p* orbitals of the metal sites can be redistributed across energy levels, thereby maximizing the modulation of their hybrid interactions. Consequently, this manipulation of local symmetry can enhance the adsorption and activation of CO_2 molecules and reactive intermediates, resulting in high activity and selectivity for the ECR reaction.[295] For example, at a potential of −0.73 V (vs. RHE), the locally planar-symmetry-broken CuN_3 (PSB-CuN_3) SAC achieves a formate FE of 94.3%, surpassing the planar-symmetry CuN_4 (PS-CuN_4) catalyst (72.4% at −0.93 V vs. RHE) (Figure 7E). The overlap between the Cu-*d* orbitals and O-*p* orbitals in the PSB-CuN_3 catalyst is significantly greater than that between the Cu-*d* and C-*p* orbitals, making the PSB-CuN_3 more inclined to bind with *OCO. Additionally, the overlap between the Cu-*d* orbitals and O-*p* orbitals in the PSB-CuN_3 catalyst is much higher than that in the PS-CuN_4, indicating that adjusting the local coordination structure can effectively modulate the binding capability between the catalyst and intermediates.[288] SACs tend to generate C_1 products due to their single active site. Despite this being the case, construction of a 2D MOF with Cu-N_1O_3 asymmetric units was shown to enable re-distribution of local electron structure, leading to atop- and bridge-type *CO adsorption on Cu and Cu−N sites, respectively.[296] The atop-type *CO species on Cu sites was protonated to form *COH/*CHO intermediates, which could couple with bridge-type *CO on Cu−N sites to generate C_2H_4/C_2H_5OH.

Synergy with supports. An appropriate support can enhance dispersion and mechanical stability of catalyst materials. Support properties contributing to CO_2 electrocatalysis include (1) base property (e.g., MgO), (2) redox property (e.g., CeO_2 and HfO_2), (3) oxygen storage/release capability (e.g., CeO_2 and ZrO_2), (4) conductivity (e.g., C), and (5) strong metal−support interaction (e.g., COFs, SiO_2).[297−299] In particular, the interaction between support and active phases can alter the electronic structure of the catalyst and/or add interfacial sites, affecting the catalyst performance. For example, electronically asymmetric Cu-Cu/Cu-N-C (Cu/CuNC) interface sites anchored on three-dimensional honeycomb-like porous C were demonstrated to enhance the adsorption of *CO intermediates and lower the reaction barrier of C−C coupling in ECR.[300] This composite boosted electrocatalytic CO_2-to-C_2H_5OH conversion with a C_2H_5OH FE of 55% at a relatively positive applied potential of −0.35 V (vs. RHE).

Interfacial electrolyte tuning

Cation impacts. The FE for C_2 products derived from CO_2 reduction demonstrates an unexpected dependence on cation size, with the efficiency increasing in the order of $Li^+ < Na^+ < K^+ < Rb^+ < Cs^+$.[301−303] There are four main hypotheses

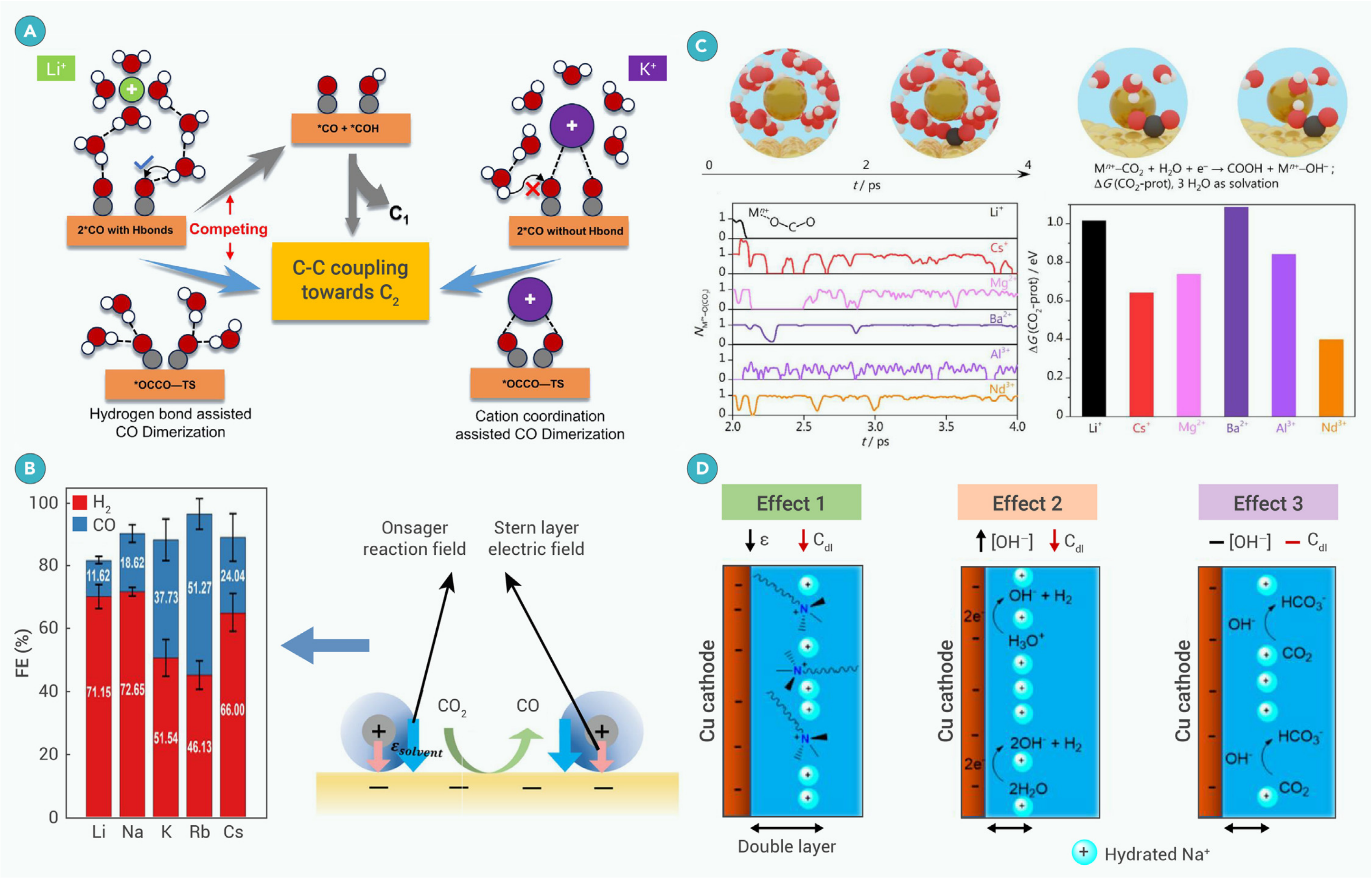

Figure 8. Interfacial electrolyte tuning strategie for ECR to liquid products (A) Schematic representation of the integrated mechanism elucidating the cation-dependent C_2 selectivity through the combination effects of interfacial water molecules and cations.[310] Copyright 2024, American Chemical Society. (B) Schematic diagram of the Onsager field resulting from the solvation shell of cations.[311] Copyright 2022, The Authors. Published by American Chemical Society. (C) Models for *Ab initio* molecular dynamics (AIMD) simulation performed at 300 K: equilibration of Au/water/M^{n+} systems (0–2 ps); equilibration of Au/water/M^{n+}/*CO_2 (2–4 ps), with M^{n+} = Li^+, Cs^+, Mg^{2+}, Ba^{2+}, Al^{3+}, and Nd^{3+}.[312] Copyright 2021, The Authors. Published by American Chemical Society. (D) Proposed mechanism for changes in capacitive behavior.[313] Copyright 2019, American Chemical Society.

to explain how metal cations at the interface alter the activity and selectivity during CO_2 electrolysis: (1) cation accumulation at the outer Helmholtz plane (OHP) suppresses the HER[304]; (2) hydrated cations act as pH-buffering agents to adjust local pH and CO_2 concentration[305]; (3) the electrostatic field generated by hydrated cations promotes the stabilization of adsorbed CO_2 or other polar intermediates (i.e., *CO_2, *CO, and *OCCO) with large dipole moments according to a field-dipole theory, while not affecting the HER due to the absence of a dipole in *H[306]; (4) metal cations may stabilize CO_2 by forming complexes, thus facilitating the formation of short-lived *CO_2^- and subsequent protonation to *COOH.[307]

Larger cations accumulate more at the surface/electrolyte interface, resulting in a more positive potential at the OHP, as per a recent theoretical study that solved the generalized modified Poisson-Nernst-Planck equations for CO_2 reduction.[308] As a consequence, H_3O^+ coverage is lowered due to electrostatic repulsion and the competitive adsorption effect, whereas neutral CO_2 molecules are unaffected, thus hindering mass transport of protons. Meanwhile, the adsorbed cations were demonstrated to have a negligible impact on the hydrogen adsorption energy in acid.[309] These aspects favor ECR over HER in acidic media. A recent theoretical study has shown that, for the production of C_2 products,[310] larger cations can coordinate with *OCCO, partially replacing the hydrogen bonds with water for stability. When larger cations (e.g., K^+) coordinate with *CO + *CO, they can repel surrounding water molecules, thereby creating a localized hydrophobic environment. This reduces the risk of *CO being protonated to form C_1 products and also explains why larger cations are beneficial in promoting C–C coupling (Figure 8A).[310]

Near a negatively charged electrode, the hydration shell of a large alkali cation (with low cation-water coordination numbers and short distances) generates a stronger electrostatic field, giving rise to a smaller pK_a of hydrolysis (Table S7)[314] and a more neutral interfacial pH, consequently increasing CO_2 concentrations at the surface. In the absence of alkali cations, rapid neutralization of CO_2 can occur by adjacent OH^- to form carbonate, resulting in a significant decrease in CO_2 concentration and ECR activity.

An induced electrostatic field was found to stabilize *CO more effectively than *CHO, thus lifting the energy barrier for the conversion of *CO to *CHO.[315] Alternatively, large cations such as Cs^+ have been suggested to stabilize the adsorbed intermediates through surface interactions but are unlikely to block the CO-binding sites.[316] However, the Onsager field resulting from the solvation shell of cations instead of the Stern layer field was proposed to play a role in enhancing kinetics by stabilizing bent CO_2 molecules following the trend $Li^+ < Na^+ < K^+ < Rb^+$ (Figure 8B).[311]

Quaternary alkyl ammonium cations (methyl$_4$N$^+$, ethyl$_4$N$^+$, propyl$_4$N$^+$, and butyl$_4$N$^+$) were claimed to affect the intermolecular interaction between surface-adsorbed CO and interfacial H_2O.[317] Larger cations impair this interaction, hindering hydrogenation of surface-bound CO to form C_{2+} products. However, a different trend was observed in acetonitrile electrolytes. Larger cations ($TBA^+ > TPA^+ > TEA^+$) resulted in a less notable increase in CO FE due to the weaker Cu–CO interaction in acetonitrile than in H_2O. Larger cations were more likely to perturb the interfacial structure of acetonitrile and tetraalkylammonium cations, contributing to boosted CO desorption.[318] Small cations (e.g., TEA^+) favored oxalate generation via CO_2^- radical dimerization without H_2O being involved, whereas, with the increase of cation chain length, the yield of oxalate substantially decreased, pointing to the reaction pathway toward CO with the involvement of H_2O.

A short-range interaction between cations and CO_2 molecules was demonstrated to take place especially for weakly hydrated cations (e.g., K^+ and Cs^+).[307] This may boost CO_2 adsorption, decrease the O–C–O angle (to ~130°), and enhance the first e^- transfer from the surface to form the *$CO_2^{\cdot-}$ intermediate. For multivalent cations, ECR activity in terms of cation–CO_2 coordination number was shown to decrease with an increase in cation acidity

(defined as the ratio of charge and ionic radius) at low overpotentials with weak water reduction.[319] Only Ba^{2+} and Cs^+ were calculated to exhibit high ECR rates at large overpotentials (Figure 8C). However, to rationalize the actual roles of cations, the interplay between the promotional impacts on both ECR and HER should be considered.

Besides the above effects, alkali cations were observed to induce Cu cathodic corrosion at potentials more negative than the onset value (e.g., −0.4 V vs. RHE when using 0.1 M $KHCO_3$).[320] The resulting surface reconstruction of Cu led to degradation of long-term selectivity and activity. Although Cu nanocubes provided better stability and ECR selectivity compared to Cu NPs, future research needs to address such alkali-cation-induced cathodic corrosion issues.

Anion impacts. Anions influence ECR through four possible mechanisms: (1) buffering local pH, (2) bicarbonate anions provide protons in electrochemical reactions, (3) promoting CO_2 activation and interactions with intermediates, (4) specifically adsorbing and maybe poisoning the surface.

For bicarbonate electrolytes, instead of acting as a buffer or proton donor, HCO_3^- increases the local CO_2 concentration via rapid chemical equilibration with $CO_{2(aq)}$ and may also serve as a CO_2 donor.[321] Meanwhile, HCO_3^- was postulated to stabilize the adsorption of $*OCO^-$ instead of *COOH, thereby promoting $HCOO^-$ production.[277] Nonetheless, high HCO_3^- concentrations usually give rise to severe formation of H_2 and CH_4. Therefore, low concentrations of HCO_3^- (e.g., 0.1 M $KHCO_3$) are usually employed.

Transfer of electrons from Cl^- to the unoccupied orbital of CO_2 via the formation of C−Cl bonds was reported, thereby accelerating the activation of CO_2.[322] Adsorbed Cl^- anions on Bi electrodes have also been demonstrated to facilitate chemical interactions with reduction intermediates to boost $HCOO^-$ formation.[323]

Anions may be adsorbed on the surface of catalysts even at potentials below the potential of zero charge (PZC). SO_4^{2-} and $H_xPO_4^{(3-x)-}$ were observed to be adsorbed on the surface of Cu in the potential range from −0.2 to −0.7 V (vs. RHE).[324] The adsorbed anions may poison the Cu surface and shift the onset of ECR to more negative potentials. HPO_4^{2-} and PO_4^{3-} were calculated to be specifically adsorbed on Cu(100) and Cu(111), respectively, therefore restraining CO adsorption.[325]

Local pH effects. Surface pH (or local/localized pH) can vary substantially owing to proton consumption during ECR and/or OH^- production from HER, affecting the conversion of CO_2 into the relatively inert CO_3^{2-} (which can be transported to the anode through the membrane; i.e., CO_2 crossover), lowering the concentrations of CO_2 (or cations) and HCO_3^-, thereby causing loss of ECR activity. Additionally, the HER is affected by the local pH. Increasing the surface pH was surmised to favor C_{2+} production because the rate-limiting step for the C_{2+} formation is independent on the pH (SHE scale), therefore reducing the overpotential by 59 mV per unit increase of pH.[41] Note that a pH gradient layer can form originating from a neutralization reaction between the CO_2 and OH^-. Hence, the decrease in overpotential reported in prior literature may be overestimated in strongly alkaline electrolytes. The selectivity for CH_3COO^- versus the other C_2 products was found to increase in concentrated alkaline solutions. This was attributed to the reaction of a ketene intermediate (H_2CCO) with OH^-.[326]

The local pH can be manipulated by introducing concentrated K^+ to enhance CO_2 activation in strong acids. Concurrently, the HER from water reduction is largely inhibited under a proton-depleted local environment.[327] Alternatively, the HER can be dramatically hampered under mildly acidic pH resulting from proton consumption by OH^- obtained from the ECR, thus eliminating CO_2/CO_3^{2-} homogeneous reactions.[328]

Electrolyte engineering with additives. The water content and structure near the surface profoundly affect the reactivity and selectivity of ECR. Adding organic molecules like *N*,*N*-dimethylformamide (DMF) to an aqueous electrolyte alters interfacial water structure, reducing HER by excluding water from the interface and intensifying DMF−water hydrogen bonds,[329] as observed by *in situ* surface-enhanced infrared absorption spectroscopy in the attenuated total reflection mode (ATR-SEIRAS). Benzotriazole (BTA) in 0.5 M $KHCO_3$ was found to facilitate the reduction of CO_2 to CH_3COO^- (with an FE ∼21% at −1.33 V vs. RHE at 0°C) on Cu-Ag clusters in stark contrast to the major product of CO in the absence of BTA.[330] However, the role of BTA remains elusive. Ionic liquid electrolytes on copper catalysts favor CO_2 reduction and inhibit the HER.[331]

Cationic surfactants such as alkyl-trimethylammonium bromides have also been used as electrolyte additives to induce an interfacial electric field due to the accumulation of the alkyl-trimethylammonium head groups at the interface, which acts to limit the available protons and thwart the competing HER (Figure 8D).[313] The hydrophobic interactions of the alkyl chain were surmised to dominate at high surfactant concentrations, causing a more compact double layer and increasing the double-layer capacitance. Increasing the chain length to induce a more hydrophobic environment, however, was observed to result in low C−C product formation but high $HCOO^-$ activity.[332]

The impact of aqueous electrolytes or additives on the surface restructuring of catalysts is less explored and deserves further investigation.

Solid electrolytes. GDEs overcome CO_2 diffusion limitations in aqueous systems, achieving current densities orders of magnitude higher. Although flow cells currently seem to be a better platform for ECR studies at industry-related current densities, flooding of the GDE with liquid electrolytes in the flow cell is a source of instability for the reaction system and greatly reduces the diffusion of CO_2 to the catalyst. The liquid electrolyte also increases the overall resistance of the electrolytic cell, resulting in a higher voltage under high current densities. The cathode side of MEA cells with a solid polymer as an electrolyte does not need liquid electrolytes, which can eliminate the flooding problem of GDEs and improve the stability of the system. The application of MEA also avoids catalyst deactivation caused by impurities in the electrolyte. However, in an MEA system, the high concentration of liquid products at the catalyst-membrane interface leads to the cross-reaction of products through the membrane, which may lead to the reoxidation of products to CO_2 at the anode. Therefore, developing stable and efficient ECR membranes is one way to promote the industrial application of ECR technology.

Inspired by solid-state batteries, SSEs have recently been used for ECR, facilitating the transfer of electrogenic cations or anions to form high-purity liquid products. Impressive production of HCOOH with a concentration of 12 M via ECR with an SSE was reported.[333] In a 100 h stability test, the selectivity of HCOOH remained above 80%. However, the application of SSEs still faces many challenges (Figure 9A).[334] For example, due to the absence of cations in SSEs, the cationic promotion observed in flow cells cannot be replicated directly in SSE reactors. In addition, the extra layer between the cathode and the anode contributes additional ohmic resistance, which reduces the energy efficiency of the reactor.

CO_2 electrolysis coupled with bio-electrocatalysis

The role of bio-electrocatalysis and the integration of electrocatalysis and biocatalysis in producing fuels has recently been reviewed by Tan and Nielson.[337] More complex chemicals, such as butyrate,[338] glucose,[75] and polyhydroxybutyrate,[339] can be synthesized using bio-electrocatalytic systems. Biocatalyst systems include microbial electrosynthesis systems (MESs) and enzyme electrocatalysis systems (EESs), depending on the nature of the biocatalyst. MESs rely on chemolithotrophic bacteria, which are able to absorb electrons from a cathode in order to catalyze a low-potential reduction of CO_2 into fuels or value-added chemicals.[340,341] EESs use enzymes to replace microorganisms, showing higher selectivity and electrocatalytic efficiency than metals and MESs.[342] However, microbial attachment characteristically has several challenges. For instance, this approach only works for a tiny subset of organisms, primarily anaerobic microorganisms that function through a reductive acetyl-coenzyme A (acetyl-CoA) pathway, leading to a narrow range of products, primarily CH_3COO^- and CH_4.[343]

In recent years, the products of microbial electrosynthesis have been expanding, especially butyric acid and caproic acid. It will be productive to combine ECR with microbial electrosynthesis to produce chemicals with higher value in the pursuit of C neutrality. A coupled electro-biosystem has been developed and utilized to upgrade CO_2 into long-chain compounds (Figure 9B). Zheng et al.[75] combined a nanostructured copper catalyst and a solid-electrolyte reactor to first convert CO_2 into high-purity CH_3COOH with a partial current density of −413 mA cm^{-2} (Figure 9C). The electrocatalytic process was followed by microbial fermentation using growing *Saccharomyces cerevisiae* to further transform CH_3COOH into glucose *in vitro*. It is envisioned that, with the production of more high-purity chemicals such as CO, HCOOH, and CH_3OH from ECR, a wider variety of valuable chemicals can be obtained via the engineering of bio-electrocatalysts using such a cascade hybrid system. This area is now attracting more attention in biochemistry.

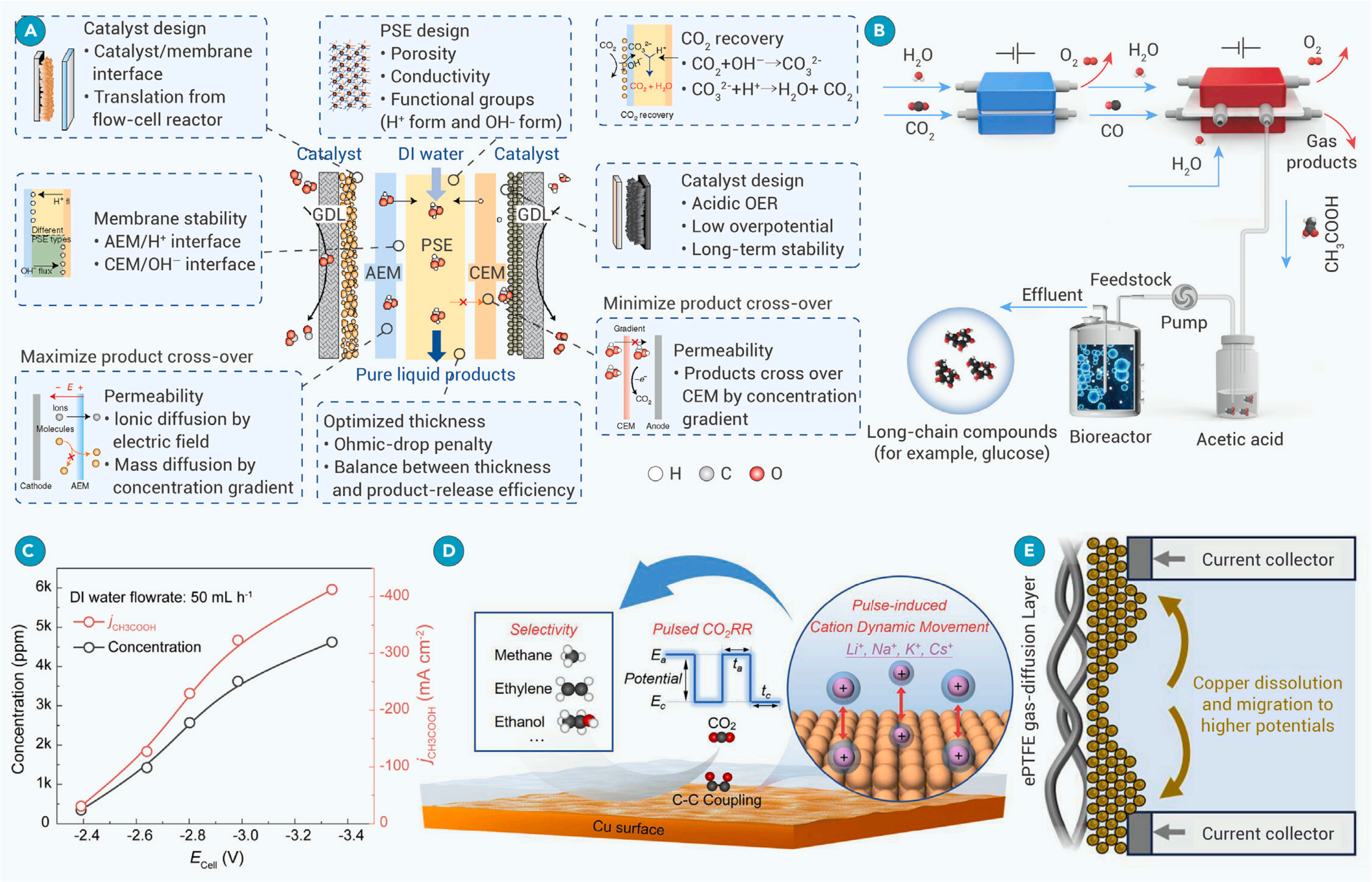

Figure 9. Solid electrolytes, coupled with bio-electrocatalysis and pulse-facilitated CO_2 electrolysis sttrategies for ECR to liquid products (A) Schematic representation of an ECR electrolyzer with a porous solid electrolyte (PSE). As shown in the main cell schematic, from left to right, the components of an electrolyzer are gas-diffusion layer (GDL), cathodic catalyst, AEM, PSE (or solid-electrolyte layer), cation-exchange membrane (CEM), anodic catalyst, and GDL.[334] Copyright 2021, Springer Nature. (B) Schematic illustration of the *in vitro* artificial sugar synthesis system. CO_2 was first converted to pure acetic acid through two-step electrolysis; this product was then directly fed into a bioreactor for microorganism fermentation to produce long-chain compounds (for example, glucose).[75] Copyright 2022, Springer Nature. (C) The average partial current density and concentrations of generated acetic acid under a deionized (DI) water flow rate of 50 mL h^{-1}.[75] Copyright 2022, Springer Nature. (D) Schematic of pulsed ECR.[335] Copyright 2024, American Chemical Society. (E) Schematic illustrating directional dissolution and migration as a result of uneven potential distribution.[336] Copyright 2024, The Authors. Published by American Chemical Society.

Pulse-facilitated CO_2 electrolysis

Compared to static electrolysis, dynamically controlling the cathode potential (pulsing between two different voltages) can (1) modulate the local CO_2 concentration, thus promoting CO_2 mass transport; and (2) reconstruct the catalyst surface by conducting pulsed electrolysis with cathode potentials either above (anodic scan) or below (cathodic scan) the standard reduction potential of the catalyst, thereby improving the ECR and mitigating the HER.[344] Differential electrochemical mass spectroscopy (DEMS)[345] in tandem with GC and high-pressure liquid chromatography (HPLC) can be utilized to probe both local and bulk concentrations of ECR products and enable direct observation of product-evolution hysteresis between cathodic and anodic scans. Upon a cathodic scan, the local pH increases due to the stoichiometric generation of OH^-, causing a slower drop in surface CO_2 concentration. The continuous pulsing results in a transient state of high local pH and CO_2 concentration as a result of the replenishment of the local CO_2 concentration during the anodic scan and hence boosts the ECR selectivity. A shorter potential pulse (~5 s) was observed to facilitate enhanced C_{2+} FEs, plausibly owing to more repeated access to the enhanced transient state.[346] Pulsed electrolysis was observed to induce cation enrichment, consequently boosting C_2H_5OH and $C_2H_5CH_2OH$ production. In contrast to static condition where the C_{2+}/CH_4 ratio increased from 0.7 to 1.9 following the order $Li^+ < Na^+ < K^+ < Cs^+$, a much more pronounced increase in C_{2+}/CH_4 ratio from 0.1 to 8.4 was attained when using pulsed electrolysis. The induced cation enrichment was associated with the radius of hydrated ions with smaller hydrated cations being more easily enriched than the larger hydrated cations (Figure 9D).[335]

More recently, an asymmetric low-frequency pulsed strategy (ALPS) was adopted to control ECR product selectivity by manipulating the size, crystal plane, and oxidation state of Cu-based nanoclusters.[282] This ALPS maximizes the effective ECR time and also enhances the energy utilization efficiency. Coupling of electrolyte engineering and pulsed CO_2 electrolysis can further improve the ECR, warranting deeper investigation.

Multiple oxidation/reduction cycles could lead to the dissolution and re-deposition of Cu fragments on both sides of the GDE (Figure 9E).[336] Combining chemical oxidation by increasing O_2 levels (to promote the ORR to form hydroxide, an oxygen source for Cu_2O) and electrochemical oxidation by applying anodic potentials can minimize the reconstruction process to lengthen Cu lifetimes.

Supercritical (SC) CO_2-assisted ECR

SC CO_2 serves as reaction medium and reactant alike, providing high CO_2 availability, limiting H_2O mass transport, and accelerating ECR kinetics to yield HCOOH on Cu electrodes.[347] Strikingly, a 4.6-fold increase in current densities was obtained in an SC CO_2/acetonitrile (MeCN)/H_2O system using an electrode comprising Cu NPs supported on graphite. The FE of the HER was greatly reduced from about 60% to less than 8% at elevated pressures. Additionally, a 2-fold increase in HCOOH selectivity (66% current efficiency) was observed. Another interesting aspect is that the SC CO_2 medium provides an alternative pathway for the formation of CH_3OH via the ECR. Employing a nonpolar SC CO_2 mixture in place of the polar MeCN electrolyte may change the CO_2 intermediate from *OCOH to *COOH, which is regarded as the most likely intermediate for CH_3OH formation. Future research should focus on advanced catalysts for SC CO_2, effective co-solvents, and enhancing electrode and membrane stability.

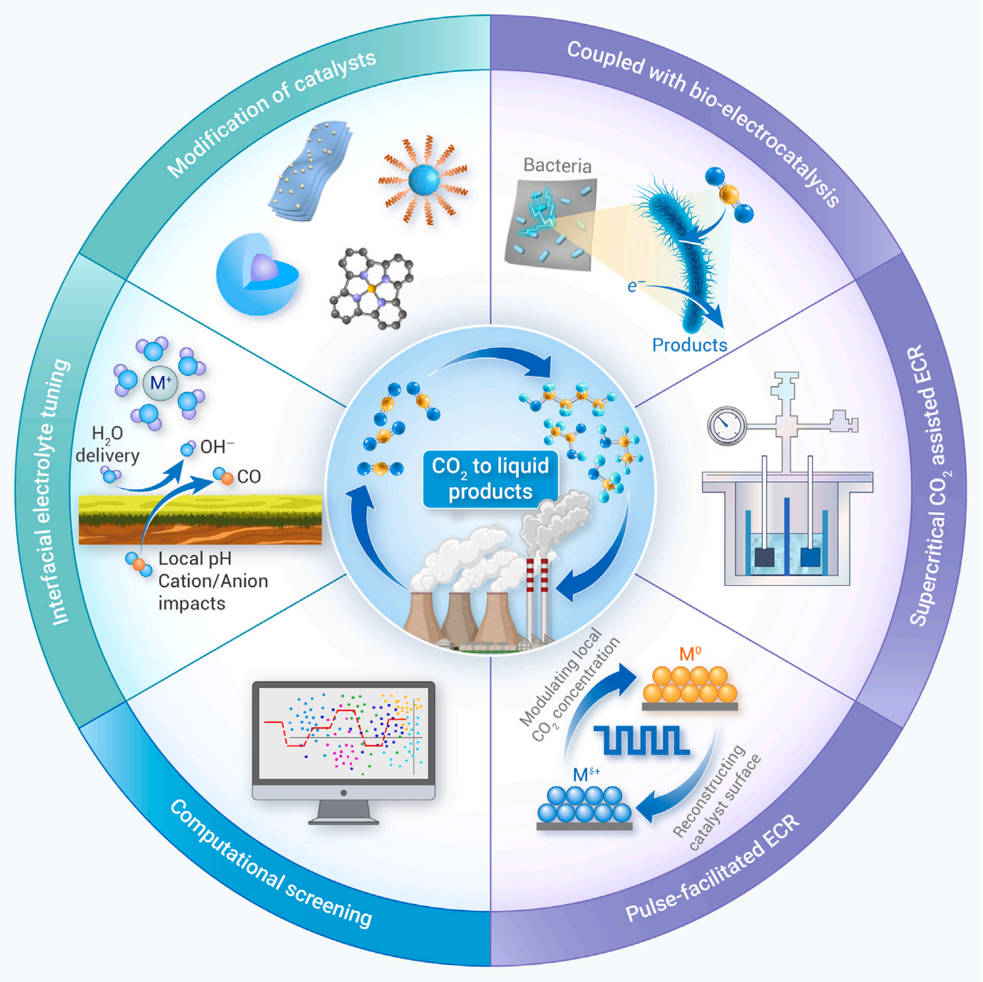

Figure 10. Various strategies for enhancing ECR to liquid products

SUMMARY

Significant advances in CO_2 conversion have been made to close the C cycle and store intermittent renewable energy. Although research efforts aiming at producing liquid chemicals or fuels by ECR have registered a lot of progress, both in terms of mechanistic studies and understanding of catalyst properties, translating laboratory results to meaningful industrial scales is still somewhat distant. Electrocatalysis is crucial to overcome ECR's kinetic and thermodynamic barriers, which determine product selectivity and turnover rate.

We have elaborated the possible reaction pathways for different liquid products of ECR from both theoretical and experimental standpoints and introduced design strategies for advanced catalysts and catalytic systems to improve the selectivity of ECR (Figure 10). In-depth understanding of the reaction pathways and mechanisms that favor specific products for a given catalyst is necessary to inform the rational design of more effective advanced electrocatalysts. However, owing to the complex nature of ECR, which involves the transfer of up to 16 electrons via competing reaction pathways, deciphering the rate-limiting reactions for a particular product using standard Tafel slope analysis is challenging. Consequently, this information is typically acquired from coupled *in situ* and online product analysis techniques that are in themselves not without errors due to the tendency of the ECR to concurrently form multiple products.

Selectivity and mechanistic pathways for electrochemical CO_2 conversion to formate, methanol, ethanol, acetal, acetate, ethylene glycol, 2-propanol, 1,2-propane diol, 3-hydroxybutanal, *n*-butanol, and *t*-butanol were articulated. FEs in excess of 90% have been reported for formate (94% with planar symmetry-broken CuN_3 catalyst),[288] methanol ($Co(CO_3)_{0.5}(OH)$ electrocatalyst, 97%[104]; metal-free boron phosphide catalyst, 92%[111]), ethanol (B, N co-doped nanodiamond[110]), and acetate (Keggin-based electrolyte-catalyst, $[SiW_9V_3O_{40}]^{-}$[120]). Conversions of CO_2 to ethanol using Ag particles anchored on graphene-wrapped N-doped C foams as heterostructured electrocatalysts and to methylglyoxal with a nickel phosphide catalyst have been demonstrated with FEs of 82%–85%[116] and 84%,[49] respectively. An FE of 71% for subsequent conversion of methylglyoxal to 2,3-furandiol has been obtained.[50] *n*-Butanol was generated with an FE of 42% using Ni-incorporated $(Cr_2O_3)_3(-Ga_2O_3)$ as a catalyst.[51] This same catalyst achieved CO_2 to 3-hydroxybutanal conversion with an FE of 23%. The highest *n*-propanol FE (30%)[125] reported so far was obtained using graphene/ZnO/Cu_2O heterostructured catalysts. Cu_xIr_{1-x} NPs were used to produce *t*-butanol with an FE of 15%.[53]

Computational approaches to catalyst screening to address stability, activity, and selectivity for promising ECR catalysts have been described. The use of ionic-liquid-based electrolytes for ECR is steadily increasing. Similarly, polymerized ionic liquids are being developed as solid electrolytes and offer opportunities for tuning activity via counter-ion exchange.

Electrocatalytic CO_2 reduction to high value-added chemicals provides a feasible strategy for negating the greenhouse effect and fostering environmental protection. Although ECR technologies still face many challenges, especially in producing high-purity liquid chemicals, it is anticipated that practical CO_2 electrolysis for clean fuels and chemical production will be realized as a result of sustained research and development of improved catalysts and optimization of the concepts of tandem catalysis, cascade electrolysis, and cell perfection.

OUTLOOK

Replication of the demonstrated catalytic efficacy of nanosheet-based catalysts to other important reactions is envisaged to increase steadily. Bio-electrocatalysis (microbial based) will become increasingly significant for the industrial conversion of CO_2 to liquid fuels.[337] ML and artificial intelligence (AI) will play increasingly important roles in catalyst, reactor, and ECR system design and optimization.

The costs of purifying CO_2 must be decreased.[348] Alternatively, catalysts robust to impure CO_2 feed streams are needed. Important issues associated with controlling overall product distribution, unsatisfactory current efficiencies, and limited durability of catalyst systems must be more intensively addressed. For example, the selectivity and stability of electrocatalysts remain significant obstacles to industrializing ECR,[349,350] especially[351] when generating higher hydrocarbons. It is crucial to improve the selectivity and stability of liquid products produced by ECR through rational catalyst design. In particular, reaction mechanisms after the first C–C dimerization step need to be further explored. An understanding of energy dissipation and how to block side reactions is needed for tuning selectivities and lengthening performance lifetimes of catalysts.[352,353]

Coupling ECR with more valuable and thermodynamically favorable anodic oxidation reactions to substitute the OER is highly desirable. This maximizes the use of electrical energy while co-producing high-value chemicals at the anode, thus boosting the overall economic value. Three main types of reactions are intriguing: (1) oxidative upgrading of biomass-derived compounds (e.g., HMF to FDCA, HMFCA, 2,5-diformylfuran, and furfurylidenelevulinic acid; glycerol to formic acid, glycolic acid, oxalic acid, glyceraldehyde, dihydroxyacetone, glyceric acid, lactic acid, and tartronic acid; glucose to gluconic acid, glucaric acid, and formic acid); (2) upcycling of plastics including polyesters (polyethylene

terephthalate and polybutylene terephthalate) and vinyl polymers (polyethylene, polystyrene, and polyvinyl chloride); and (3) electro-organic synthesis via dissociation and formation of C-, H-, O-, N-, and S-based bonds through dehydrogenation, oxygenation, and oxidative coupling, among others. Furthermore, integrating CO_2 with other substrates such as nitrogenous small molecules, propargyl alcohols, and epoxides via electrocatalysis deserves future major efforts to produce a wider range of chemicals and fuels, facilitating the effective and sustainable utilization of CO_2.

Gas-diffusion electrode membrane assembly cells will dominate reactor designs for the foreseeable future. Electrolyzer cell (reactor) designs will be optimized in order to support ECR commercialization.[348] These designs include those for various 3D materials and membrane electrode cell assemblies.[337]

Facile ion diffusion will be key for at least one phase used in advanced electrodes, and MXenes may provide this important property. Improved structure-activity relationships are needed in order to better inform approaches to optimizing catalyst and system designs for reaction steps encountered in synthesizing particular liquid fuels and feedstocks.

Liquid product crossover remains a pervasive problem in ECR.[354] Advances are needed in designing polyelectrolyte membranes that more extensively suppress liquid product crossover while facilitating ion diffusion.

The development of effective strategies to modify the electronic structure and properties of electrocatalysts in a controlled manner are needed. Variations in the band gap as a function of dimensionality (for example, modulating the band gap through control of nanosheet thicknesses) distinguish the properties and performance of many of these 2D materials from their bulk counterparts and offer potential tuning efficiencies for electron-hole separation and for intrinsic CO_2 photo/electroreduction activity.[355]

Further advances in fabricating and controlling surface chemistry are needed to enhance ECR performance. Tailoring 2D-based nanostructures and their interfaces with specific synergies between different catalytic functionalities is especially important for maximizing catalytic activity. Heightened attention should be paid to compositions intercalated between 2D layers that are otherwise difficult to prepare via traditional synthesis protocols. These compositions are expected to afford unusual catalytic properties due to confinement effects and electrolyte sequestering properties.

Improved *in situ* characterization methods are needed for catalytic mechanistic studies and for quantifying ion transport and diffusion through multiphase heterostructures. Developing *in situ* characterization techniques will help inform theoretical models and deepen our understanding of catalytic structure-property relationships. Li and coworkers have summarized[356] the recent progress in this area.

The importance and impact of heterostructured catalysts, tandem catalysis, and cascade reactors will steadily increase, particularly for designing multistep fuel syntheses emanating from CO_2. New catalysts with nanoarchitectures will be developed by manipulating 2D materials and scaffolds. For example, nanosheets can be surface decorated with catalytic NP structures to direct particularly desired reaction steps, and such surface modifications can be used to design multiple mechanistic catalytic steps.

REFERENCES

1. Dou, X., Wang, Y., Ciais, P. et al. (2022). Near-real-time global gridded daily CO_2 emissions. *Innovation* **3**:100182. DOI:https://doi.org/10.1016/j.xinn.2021.100182.
2. Lan, X., Tans, P. and Thoning, K.W. (2025). Thoning: Trends in Globally-Averaged CO2 Determined from NOAA Global Monitoring Laboratory measurements. https://doi.org/10.15138/9N0H-ZH07.
3. Ebi, K.L. (2012). Key themes in the working group II contribution to the intergovernmental panel on climate change 5th assessment report. *Clim. Change* **114**:417–426. DOI:https://doi.org/10.1007/s10584-012-0442-4.
4. Kibria, M.G., Edwards, J.P., Gabardo, C.M. et al. (2019). Electrochemical CO_2 reduction into chemical feedstocks: from mechanistic electrocatalysis models to system design. *Adv. Mater.* **31**:e1807166. DOI:https://doi.org/10.1002/adma.201807166.
5. Fan, X., Sun, X., Robertson, A.W. et al. (2024). Charged sorbents for efficient CO_2 removal. *Innov. Mater.* **2**:100088. DOI:https://doi.org/10.59717/j.xinn-mater.2024.100088.
6. Chen, X. and Wu, X. (2022). The roles of carbon capture, utilization and storage in the transition to a low-carbon energy system using a stochastic optimal scheduling approach. *J. Clean. Prod.* **366**:132860. DOI:https://doi.org/10.1016/j.jclepro.2022.132860.
7. Zhang, L., Dang, Y., Zhou, X. et al. (2021). Direct conversion of CO_2 to a jet fuel over CoFe alloy catalysts. *Innovation* **2**:100170. DOI:https://doi.org/10.1016/j.xinn.2021.100170.
8. Li, H., Fang, W., Wang, L.X. et al. (2023). Physical regulation of copper catalyst with a hydrophobic promoter for enhancing CO_2 hydrogenation to methanol. *Innovation* **4**:100445. DOI:https://doi.org/10.1016/j.xinn.2023.100445.
9. De, S., Dokania, A., Ramirez, A. et al. (2020). Advances in the design of heterogeneous catalysts and thermocatalytic processes for CO_2 utilization. *ACS Catal.* **10**:14147–14185. DOI:https://doi.org/10.1021/acscatal.0c04273.
10. Pan, F. and Yang, Y. (2020). Designing CO_2 reduction electrode materials by morphology and interface engineering. *Energy Environ. Sci.* **13**:2275–2309. DOI:https://doi.org/10.1039/D0EE00900H.
11. Kvaleski, D. (2024). DOE awards $41M to 14 projects for renewables-to-liquids technologies, **6** (Daily Energy Insider). https://dailyenergyinsider.com/.
12. Lin, L., He, X., Zhang, X.G. et al. (2023). A nanocomposite of bismuth clusters and $Bi_2O_2CO_3$ sheets for highly efficient electrocatalytic reduction of CO_2 to formate. *Angew. Chem., Int. Ed.* **62**:e202214959. DOI:https://doi.org/10.1002/anie.202214959.
13. Guo, C., Guo, Y., Shi, Y. et al. (2022). Electrocatalytic reduction of CO_2 to ethanol at close to theoretical potential via engineering abundant electron-donating $Cu^{\delta+}$ species. *Angew. Chem., Int. Ed.* **61**:e202205909. DOI:https://doi.org/10.1002/anie.202205909.
14. Tan, D., Wulan, B., Ma, J. et al. (2023). Electrochemical-driven reconstruction for efficient reduction of carbon dioxide into alcohols. *Chem Catal.* **3**:100512. DOI:https://doi.org/10.1016/j.checat.2023.100512.
15. Bushuyev, O.S., De Luna, P., Dinh, C.T. et al. (2018). What should we make with CO_2 and how can we make it? *Joule* **2**:825–832. DOI:https://doi.org/10.1016/j.joule.2017.09.003.
16. Greenblatt, J.B., Miller, D.J., Ager, J.W. et al. (2018). The technical and energetic challenges of separating (photo) electrochemical carbon dioxide reduction products. *Joule* **2**:381–420. DOI:https://doi.org/10.1016/j.joule.2018.01.014.
17. Benson, E.E., Kubiak, C.P., Sathrum, A.J. et al. (2009). Electrocatalytic and homogeneous approaches to conversion of CO_2 to liquid fuels. *Chem. Soc. Rev.* **38**:89–99. DOI:https://doi.org/10.1039/B804323J.
18. Costentin, C., Robert, M. and Savéant, J.-M. (2013). Catalysis of the electrochemical reduction of carbon dioxide. *Chem. Soc. Rev.* **42**:2423–2436. DOI:https://doi.org/10.1039/C2CS35360A.
19. Liu, Y., Zhao, J. and Cai, Q. (2016). Pyrrolic-nitrogen doped graphene: a metal-free electrocatalyst with high efficiency and selectivity for the reduction of carbon dioxide to formic acid: a computational study. *Phys. Chem. Chem. Phys.* **18**:5491–5498. DOI:https://doi.org/10.1039/c5cp07458d.
20. Zhang, Y., Yang, R., Li, H. et al. (2022). Boosting electrocatalytic reduction of CO_2 to HCOOH on Ni single atom anchored WTe_2 monolayer. *Small* **18**:e2203759. DOI:https://doi.org/10.1002/smll.202203759.
21. Nakata, K., Ozaki, T., Terashima, C. et al. (2014). High-yield electrochemical production of formaldehyde from CO_2 and seawater. *Angew. Chem., Int. Ed.* **53**:871–874. DOI:https://doi.org/10.1002/anie.201308657.
22. Calle-Vallejo, F. and Koper, M.T.M. (2013). Theoretical considerations on the electroreduction of CO to C_2 species on Cu(100) electrodes. *Angew. Chem., Int. Ed.* **52**:7282–7285. DOI:https://doi.org/10.1002/anie.201301470.
23. Cheng, T., Xiao, H. and Goddard, W.A. (2017). Full atomistic reaction mechanism with kinetics for CO reduction on Cu(100) from ab initio molecular dynamics free-energy calculations at 298 K. *Proc. Natl. Acad. Sci. USA* **114**:1795–1800. DOI:https://doi.org/10.1073/pnas.1612106114.
24. Goodpaster, J.D., Bell, A.T. and Head-Gordon, M. (2016). Identification of possible pathways for C–C bond formation during electrochemical reduction of CO_2: new theoretical insights from an improved electrochemical model. *J. Phys. Chem. Lett.* **7**:1471–1477. DOI:https://doi.org/10.1021/acs.jpclett.6b00358.
25. Zhang, Z., Bian, L., Tian, H. et al. (2022). Tailoring the surface and interface structures of copper-based catalysts for electrochemical reduction of CO_2 to ethylene and ethanol. *Small* **18**:2107450. DOI:https://doi.org/10.1002/smll.202107450.
26. Kortlever, R., Shen, J., Schouten, K.J.P. et al. (2015). Catalysts and reaction pathways for the electrochemical reduction of carbon dioxide. *J. Phys. Chem. Lett.* **6**:4073–4082. DOI:https://doi.org/10.1021/acs.jpclett.5b01559.
27. Shen, H., Wang, Y., Chakraborty, T. et al. (2022). Asymmetrical C–C coupling for electroreduction of CO on bimetallic Cu–Pd catalysts. *ACS Catal.* **12**:5275–5283. DOI:https://doi.org/10.1021/acscatal.2c00646.
28. Xie, L., Liu, X., Huang, F. et al. (2022). Regulating Pd-catalysis for electrocatalytic CO_2 reduction to formate via intermetallic PdBi nanosheets. *Chin. J. Catal.* **43**:1680–1686. DOI:https://doi.org/10.1016/S1872-2067(21)63999-2.
29. Luo, M., Wang, Z., Li, Y.C. et al. (2019). Hydroxide promotes carbon dioxide electroreduction to ethanol on copper via tuning of adsorbed hydrogen. *Nat. Commun.* **10**:5814. DOI:https://doi.org/10.1038/s41467-019-13833-8.
30. Luan, P., Dong, X., Liu, L. et al. (2024). Selective electrosynthesis of ethanol via asymmetric C–C coupling in tandem CO_2 reduction. *ACS Catal.* **14**:8776–8785. DOI:https://doi.org/10.1021/acscatal.4c01579.
31. Birdja, Y.Y. and Koper, M.T.M. (2017). The importance of cannizzaro-type reactions during electrocatalytic reduction of carbon dioxide. *J. Am. Chem. Soc.* **139**:2030–2034. DOI:https://doi.org/10.1021/jacs.6b12008.
32. Garza, A.J., Bell, A.T. and Head-Gordon, M. (2018). Mechanism of CO_2 reduction at copper surfaces: pathways to C_2 products. *ACS Catal.* **8**:1490–1499. DOI:https://doi.org/10.1021/acscatal.7b03477.
33. Qiu, X.-F., Huang, J.-R., Yu, C. et al. (2022). A stable and conductive covalent organic framework with isolated active sites for highly selective electroreduction of carbon dioxide to acetate. *Angew. Chem., Int. Ed.* **61**:e202206470. DOI:https://doi.org/10.1002/anie.202206470.
34. Luc, W., Fu, X., Shi, J. et al. (2019). Two-dimensional copper nanosheets for electrochemical reduction of carbon monoxide to acetate. *Nat. Catal.* **2**:423–430. DOI:https://doi.org/10.1038/s41929-019-0269-8.

www.the-innovation.org

35. Liu, Y., Chen, S., Quan, X. et al. (2015). Efficient electrochemical reduction of carbon dioxide to acetate on nitrogen-doped nanodiamond. *J. Am. Chem. Soc.* **137**:11631–11636. DOI:https://doi.org/10.1021/jacs.5b02975.
36. Lv, L., Lu, R., Zhu, J. et al. (2023). Coordinating the edge defects of bismuth with sulfur for enhanced CO_2 electroreduction to formate. *Angew. Chem., Int. Ed.* **62**:e202303117. DOI:https://doi.org/10.1002/anie.202303117.
37. Calvinho, K.U.D., Alherz, A.W., Yap, K.M.K. et al. (2021). Surface hydrides on Fe_2P electrocatalyst reduce CO_2 at low overpotential: steering selectivity to ethylene glycol. *J. Am. Chem. Soc.* **143**:21275–21285. DOI:https://doi.org/10.1021/jacs.1c03428.
38. Zheng, Y., Vasileff, A., Zhou, X. et al. (2019). Understanding the roadmap for electrochemical reduction of CO_2 to multi-carbon oxygenates and hydrocarbons on copper-based catalysts. *J. Am. Chem. Soc.* **141**:7646–7659. DOI:https://doi.org/10.1021/jacs.9b02124.
39. Clark, E.L. and Bell, A.T. (2018). Direct observation of the local reaction environment during the electrochemical reduction of CO_2. *J. Am. Chem. Soc.* **140**:7012–7020. DOI:https://doi.org/10.1021/jacs.8b04058.
40. Pang, Y., Li, J., Wang, Z. et al. (2019). Efficient electrocatalytic conversion of carbon monoxide to propanol using fragmented copper. *Nat. Catal.* **2**:251–258. DOI:https://doi.org/10.1038/s41929-019-0225-7.
41. Hori, Y., Takahashi, R., Yoshinami, Y. et al. (1997). Electrochemical reduction of CO at a copper electrode. *J. Phys. Chem. B* **101**:7075–7081. DOI:https://doi.org/10.1021/jp970284i.
42. Montoya, J.H., Shi, C., Chan, K. et al. (2015). Theoretical insights into a CO dimerization mechanism in CO_2 electroreduction. *J. Phys. Chem. Lett.* **6**:2032–2037. DOI:https://doi.org/10.1021/acs.jpclett.5b00722.
43. Zhuang, T.T., Liang, Z.Q., Seifitokaldani, A. et al. (2018). Steering post-C–C coupling selectivity enables high efficiency electroreduction of carbon dioxide to multi-carbon alcohols. *Nat. Catal.* **1**:421–428. DOI:https://doi.org/10.1038/s41929-018-0084-7.
44. Zhao, K., Nie, X., Wang, H. et al. (2020). Selective electroreduction of CO_2 to acetone by single copper atoms anchored on N-doped porous carbon. *Nat. Commun.* **11**:2455. DOI:https://doi.org/10.1038/s41467-020-16381-8.
45. Liu, G., Adesina, P., Nasiri, N. et al. (2022). Elucidating reaction pathways of the CO_2 electroreduction via tailorable tortuosities and oxidation states of Cu nanostructures. *Adv. Funct. Mater.* **32**:220493. DOI:https://doi.org/10.1002/adfm.202204993.
46. Qi, K., Zhang, Y., Onofrio, N. et al. (2023). Unlocking direct CO_2 electrolysis to C_3 products via electrolyte supersaturation. *Nat. Catal.* **6**:319–331. DOI:https://doi.org/10.1038/s41929-023-00938-z.
47. Chen, L., Tang, C., Zheng, Y. et al. (2022). C_3 production from CO_2 reduction by concerted *CO trimerization on a single-atom alloy catalyst. *J. Mater. Chem. A* **10**:5998–6006. DOI:https://doi.org/10.1039/D1TA09608G.
48. Banerjee, S., Kakekhani, A., Wexler, R.B. et al. (2021). Mechanistic insights into CO_2 electroreduction on Ni_2P: understanding its selectivity toward multicarbon products. *ACS Catal.* **11**:11706–11715. DOI:https://doi.org/10.1021/acscatal.1c03639.
49. Calvinho, K.U.D., Laursen, A.B., Yap, K.M.K. et al. (2018). Selective CO_2 reduction to C_3 and C_4 oxyhydrocarbons on nickel phosphides at overpotentials as low as 10 mV. *Energy Environ. Sci.* **11**:2550–2559. DOI:https://doi.org/10.1039/C8EE00936H.
50. Zheng, S., Liang, X., Pan, J. et al. (2023). Multi-center cooperativity enables facile C–C coupling in electrochemical CO_2 reduction on a Ni_2P catalyst. *ACS Catal.* **13**:2847–2856. DOI:https://doi.org/10.1021/acscatal.2c05611.
51. Cronin, S.P., Dulovic, S., Lawrence, J.A. et al. (2023). Direct synthesis of 1-butanol with high faradaic efficiency from CO_2 utilizing cascade catalysis at a Ni-enhanced $(Cr_2O_3)_3Ga_2O_3$ electrocatalyst. *J. Am. Chem. Soc.* **145**:6762–6772. DOI:https://doi.org/10.1021/jacs.2c12251.
52. Ting, L.R.L., García-Muelas, R., Martín, A.J. et al. (2020). Electrochemical reduction of carbon dioxide to 1-butanol on oxide-derived copper. *Angew. Chem., Int. Ed.* **59**:21072–21079. DOI:https://doi.org/10.1002/anie.202008289.
53. Kim, M.G., Park, J., Choi, Y. et al. (2023). CuIr nanoparticles for electrochemical reduction of CO_2 to t-BuOH. *Adv. Energy Mater.* **13**:2300749. DOI:https://doi.org/10.1002/aenm.202300749.
54. Nitopi, S., Bertheussen, E., Scott, S.B. et al. (2019). Progress and perspectives of electrochemical CO_2 reduction on copper in aqueous electrolyte. *Chem. Rev.* **119**:7610–7672. DOI:https://doi.org/10.1021/acs.chemrev.8b00705.
55. Tan, Y.C., Lee, K.B., Song, H. et al. (2020). Modulating local CO_2 concentration as a general strategy for enhancing C–C coupling in CO_2 electroreduction. *Joule* **4**:1104–1120. DOI:https://doi.org/10.1016/j.joule.2020.03.013.
56. Liu, X., Schlexer, P., Xiao, J. et al. (2019). pH effects on the electrochemical reduction of CO_2 towards C_2 products on stepped copper. *Nat. Commun.* **10**:32. DOI:https://doi.org/10.1038/s41467-018-07970-9.
57. Bligaard, T., Nørskov, J.K., Dahl, S. et al. (2004). The Brønsted-Evans-Polanyi relation and the volcano curve in heterogeneous catalysis. *J. Catal.* **224**:206–217. DOI:https://doi.org/10.1016/j.jcat.2004.02.034.
58. Bang, G.J., Gu, G.H., Noh, J. et al. (2022). Activity trends of methane oxidation catalysts under emission conditions. *ACS Catal.* **12**:10255–10263. DOI:https://doi.org/10.1021/acscatal.2c00842.
59. Kumar, A., Iyer, J., Jalid, F. et al. (2021). Machine learning enabled screening of single atom alloys: predicting reactivity trend for ethanol dehydrogenation. *ChemCatChem* **14**:e202101481. DOI:https://doi.org/10.1002/cctc.202101481.
60. Motagamwala, A.H. and Dumesic, J.A. (2021). Microkinetic modeling: a tool for rational catalyst design. *Chem. Rev.* **121**:1049–1076. DOI:https://doi.org/10.1021/acs.chemrev.0c00394.
61. Lu, X., Obata, K. and Takanabe, K. (2023). Microkinetic studies for mechanism interpretation in electrocatalytic CO and CO_2 reduction: current and perspective. *EES Catal.* **1**:590–618. DOI:https://doi.org/10.1039/D3EY00079F.
62. Marshall, A.T. (2018). Using microkinetic models to understand electrocatalytic reactions. *Curr. Opin. Electrochem.* **7**:75–80. DOI:https://doi.org/10.1016/j.coelec.2017.10.024.
63. Baz, A., Dix, S.T., Holewinski, A. et al. (2021). Microkinetic modeling in electrocatalysis: applications, limitations, and recommendations for reliable mechanistic insights. *J. Catal.* **404**:864–872. DOI:https://doi.org/10.1016/j.jcat.2021.08.043.
64. Cerrón-Calle, G.A., Senftle, T.P. and Garcia-Segura, S. (2022). Strategic tailored design of electrocatalysts for environmental remediation based on density functional theory (DFT) and microkinetic modeling. *Curr. Opin. Chem. Eng.* **35**:101062. DOI:https://doi.org/10.1016/j.coelec.2022.101062.
65. Goodpaster, J.D., Bell, A.T. and Head-Gordon, M. (2016). Identification of possible pathways for C–C bond formation during electrochemical reduction of CO_2: new theoretical insights from an improved electrochemical model. *J. Phys. Chem. Lett.* **7**:1471–1477. DOI:https://doi.org/10.1021/acs.jpclett.6b00358.
66. Singh, M.R., Goodpaster, J.D., Weber, A.Z. et al. (2017). Mechanistic insights into electrochemical reduction of CO_2 over Ag using density functional theory and transport models. *Proc. Natl. Acad. Sci. USA* **114**:E8812–E8821. DOI:https://doi.org/10.1073/pnas.1713164114.
67. Rihm, S.D., Akroyd, J. and Kraft, M. (2023). Modelling a detailed kinetic mechanism for electrocatalytic reduction of CO_2. *Proc. Combust. Inst.* **39**:5647–5655. DOI:https://doi.org/10.1016/j.proci.2022.07.096.
68. Huang, Y., Handoko, A.D., Hirunsit, P. et al. (2017). Electrochemical reduction of CO_2 using copper single-crystal surfaces: effects of CO* coverage on the selective formation of ethylene. *ACS Catal.* **7**:1749–1756. DOI:https://doi.org/10.1021/acscatal.6b03147.
69. Weng, L.C., Bell, A.T. and Weber, A.Z. (2019). Towards membrane-electrode assembly systems for CO_2 reduction: a modeling study. *Energy Environ. Sci.* **12**:1950–1968. DOI:https://doi.org/10.1039/C9EE00909D.
70. Verma, S., Lu, S. and Kenis, P.J.A. (2019). Co-electrolysis of CO_2 and glycerol as a pathway to carbon chemicals with improved technoeconomics due to low electricity consumption. *Nat. Energy* **4**:466–474. DOI:https://doi.org/10.1038/s41560-019-0374-6.
71. Li, D., Yang, J., Lian, J. et al. (2023). Recent advances in paired electrolysis coupling CO_2 reduction with alternative oxidation reactions. *J. Energy Chem.* **77**:406–419. DOI:https://doi.org/10.1016/j.jechem.2022.10.031.
72. Na, J., Seo, B., Kim, J. et al. (2019). General technoeconomic analysis for electrochemical coproduction coupling carbon dioxide reduction with organic oxidation. *Nat. Commun.* **10**:5193. DOI:https://doi.org/10.1038/s41467-019-12744-y.
73. Wang, T., Tao, L., Zhu, X. et al. (2021). Combined anodic and cathodic hydrogen production from aldehyde oxidation and hydrogen evolution reaction. *Nat. Catal.* **5**:66–73. DOI:https://doi.org/10.1038/s41929-021-00721-y.
74. Prajapati, A., Collins, B.A., Goodpaster, J.D. et al. (2021). Fundamental insight into electrochemical oxidation of methane towards methanol on transition metal oxides. *Proc. Natl. Acad. Sci. USA* **118**:e2023233118. DOI:https://doi.org/10.1073/pnas.2023233118.
75. Zheng, T., Zhang, M., Wu, L. et al. (2022). Upcycling CO_2 into energy-rich long-chain compounds via electrochemical and metabolic engineering. *Nat. Catal.* **5**:388–396. DOI:https://doi.org/10.1038/s41929-022-00775-6.
76. Clark, E.L., Resasco, J., Landers, A. et al. (2018). Standards and protocols for data acquisition and reporting for studies of the electrochemical reduction of carbon dioxide. *ACS Catal.* **8**:6560–6570. DOI:https://doi.org/10.1021/acscatal.8b01340.
77. Ding, P., An, H., Zellner, P. et al. (2023). Elucidating the roles of nafion/solvent formulations in copper-catalyzed CO_2 electrolysis. *ACS Catal.* **13**:5336–5347. DOI:https://doi.org/10.1021/acscatal.2c05235.
78. Leung, K.Y. and McCrory, C.C.L. (2019). Effect and prevention of trace Ag^+ contamination from Ag/AgCl reference electrodes on CO_2 reduction product distributions at polycrystalline copper electrodes. *ACS Appl. Energy Mater.* **2**:8283–8293. DOI:https://doi.org/10.1021/acsaem.9b01759.
79. Hori, Y., Konishi, H., Futamura, T. et al. (2005). "Deactivation of copper electrode" in electrochemical reduction of CO_2. *Electrochim. Acta* **50**:5354–5369. DOI:https://doi.org/10.1016/j.electacta.2005.03.015.
80. Jovanov, Z.P., Ferreira de Araujo, J., Li, S. et al. (2019). Catalyst preoxidation and EDTA electrolyte additive remedy activity and selectivity declines during electrochemical CO_2 reduction. *J. Phys. Chem. C* **123**:2165–2174. DOI:https://doi.org/10.1021/acs.jpcc.8b08794.
81. Papangelakis, P., Miao, R.K., Lu, R. et al. (2024). Improving the SO_2 tolerance of CO_2 reduction electrocatalysts using a polymer/catalyst/ionomer heterojunction design. *Nat. Energy* **9**:1011–1020. DOI:https://doi.org/10.1038/s41560-024-01577-9.
82. He, M., Li, C., Zhang, H. et al. (2020). Oxygen induced promotion of electrochemical reduction of CO_2 via co-electrolysis. *Nat. Commun.* **11**:3844. DOI:https://doi.org/10.1038/s41467-020-17690-8.
83. Wang, M., Wang, B., Zhang, J. et al. (2024). Acidic media enables oxygen-tolerant electrosynthesis of multicarbon products from simulated flue gas. *Nat. Commun.* **15**:1218. DOI:https://doi.org/10.1038/s41467-024-45527-1.
84. Kreft, S., Schoch, R., Schneidewind, J. et al. (2019). Improving selectivity and activity of CO_2 reduction photocatalysts with oxygen. *Chem* **5**:1818–1833. DOI:https://doi.org/10.1016/j.chempr.2019.04.006.
85. Fu, Z., Ouyang, Y., Wu, M. et al. (2024). Mechanism of surface oxygen-containing species promoted electrocatalytic CO_2 reduction. *Sci. Bull.* **69**:1410–1417. DOI:https://doi.org/10.1016/j.scib.2024.03.012.

86. Niu, Z.Z., Chi, L.P., Liu, R. et al. (2021). Rigorous assessment of CO_2 electroreduction products in a flow cell. *Energy Environ. Sci.* **14**:4169–4176. DOI:https://doi.org/10.1039/D1EE01664D.
87. McCallum, C., Gabardo, C.M., O'Brien, C.P. et al. (2021). Reducing the crossover of carbonate and liquid products during carbon dioxide electroreduction. *Cell Rep. Phys. Sci.* **2**:100522. DOI:https://doi.org/10.1016/j.xcrp.2021.100522.
88. Lu, X., Obata, K. and Takanabe, K. (2023). Microkinetic studies for mechanism interpretation in electrocatalytic CO and CO_2 reduction: current and perspective. *EES Catal.* **1**:590–618. DOI:https://doi.org/10.1039/D3EY00079F.
89. Löwe, A., Rieg, C., Hierlemann, T. et al. (2019). Influence of temperature on the performance of gas diffusion electrodes in the CO_2 reduction reaction. *Chemelectrochem* **6**:4497–4506. DOI:https://doi.org/10.1002/celc.201900872.
90. Duan, Z., Sun, R., Zhu, C. et al. (2006). An improved model for the calculation of CO_2 solubility in aqueous solutions containing Na^+, K^+, Ca^{2+}, Mg^{2+}, Cl^-, and SO_4^{2-}. *Mar. Chem.* **98**:131–139. DOI:https://doi.org/10.1016/j.marchem.2005.09.001.
91. Huang, L., Gao, G., Yang, C. et al. (2023). Pressure dependence in aqueous-based electrochemical CO_2 reduction. *Nat. Commun.* **14**:2958. DOI:https://doi.org/10.1038/s41467-023-38775-0.
92. Dunwell, M., Luc, W., Yan, Y. et al. (2018). Understanding surface-mediated electrochemical reactions: CO_2 reduction and beyond. *ACS Catal.* **8**:8121–8129. DOI:https://doi.org/10.1021/acscatal.8b02181.
93. Ratschmeier, B., Paulsen, C., Stallberg, K. et al. (2024). Cu/Au(111) surfaces and AuCu intermetallics for electrocatalytic reduction of CO_2 in ionic liquid electrolytes. *ACS Catal.* **14**:1773–1784. DOI:https://doi.org/10.1021/acscatal.3c04592.
94. Birdja, Y.Y., Pérez-Gallent, E., Figueiredo, M.C. et al. (2019). Advances and challenges in understanding the electrocatalytic conversion of carbon dioxide to fuels. *Nat. Energy* **4**:732–745. DOI:https://doi.org/10.1038/s41560-019-0450-y.
95. Hori, Y. (2008). Electrochemical CO_2 reduction on metal electrodes. In Mod. Aspects Electrochem., C.G. Vayenas, R.E. White, and M.E. Gamboa-Aldeco, eds. (Springer), pp. 89–189. DOI:https://doi.org/10.1007/978-0-387-49489-0_3.
96. Jiang, M., Zhu, M., Wang, H. et al. (2023). Rapid and green electric-explosion preparation of spherical indium nanocrystals with abundant metal defects for highly-selective CO_2 electroreduction. *Nano Lett.* **23**:291–297. DOI:https://doi.org/10.1021/acs.nanolett.2c04335.
97. Duan, X.Q., Duan, G.Y., Wang, Y.F. et al. (2023). Sn-Ag synergistic effect enhances high-rate electrocatalytic CO_2-to-formate conversion on porous poly(ionic liquid) support. *Small* **19**:e2207219. DOI:https://doi.org/10.1002/smll.202207219.
98. Guo, S., Liu, Y., Murphy, E. et al. (2022). Robust palladium hydride catalyst for electrocatalytic formate formation with high CO tolerance. *Appl. Catal., B* **316**:121659. DOI:https://doi.org/10.1016/j.apcatb.2022.121659.
99. Wu, Z., Wu, H., Cai, W. et al. (2021). Engineering bismuth-tin interface in bimetallic aerogel with a 3D porous structure for highly selective electrocatalytic CO_2 reduction to HCOOH. *Angew. Chem., Int. Ed.* **60**:12554–12559. DOI:https://doi.org/10.1002/anie.202102832.
100. Lin, L., He, X., Zhang, X.G. et al. (2023). A nanocomposite of bismuth clusters and $Bi_2O_2CO_3$ sheets for highly efficient electrocatalytic reduction of CO_2 to formate. *Angew. Chem., Int. Ed.* **62**:e202214959. DOI:https://doi.org/10.1002/anie.202214959.
101. Yang, Q., Liu, X., Peng, W. et al. (2021). Vanadium oxide integrated on hierarchically nanoporous copper for efficient electroreduction of CO_2 to ethanol. *J. Mater. Chem. A* **9**:3044–3051. DOI:https://doi.org/10.1039/D0TA09522B.
102. Masel, R.I., Liu, Z., Yang, H. et al. (2021). An industrial perspective on catalysts for low-temperature CO_2 electrolysis. *Nat. Nanotechnol.* **16**:118–128. DOI:https://doi.org/10.1038/s41565-020-00823-x.
103. Fang, W., Guo, W., Lu, R. et al. (2024). Durable CO_2 conversion in the proton-exchange membrane system. *Nature* **626**:86–91. DOI:https://doi.org/10.1038/s41586-023-06917-5.
104. Huang, J., Hu, Q., Guo, X. et al. (2018). Rethinking $Co(CO_3)_{0.5}(OH)\cdot 0.11H_2O$: a new property for highly selective electrochemical reduction of carbon dioxide to methanol in aqueous solution. *Green Chem.* **20**:2967–2972. DOI:https://doi.org/10.1039/C7GC03744A.
105. Kong, S., Lv, X., Wang, X. et al. (2022). Delocalization state-induced selective bond breaking for efficient methanol electrosynthesis from CO_2. *Nat. Catal.* **6**:6–15. DOI:https://doi.org/10.1038/s41929-022-00887-z.
106. Mahrim, A.H.B., Aun, T.T., Alias, Y.B. et al. (2023). Ionic liquids/polyionic liquids assisted synthesis of CuO nanoparticles for CO_2 reduction to formic acid. *Ionics* **30**:951–970. DOI:https://doi.org/10.1007/s11581-023-05338-8.
107. Noh, S., Cho, Y.J., Zhang, G. et al. (2023). Insight into the role of entropy in promoting electrochemical CO_2 reduction by imidazolium cations. *J. Am. Chem. Soc.* **145**:27657–27663. DOI:https://doi.org/10.1021/jacs.3c09687.
108. Yang, Y., Fu, J., Ouyang, Y. et al. (2023). In-situ constructed Cu/CuNC interfaces for low-overpotential reduction of CO_2 to ethanol. *Natl. Sci. Rev.* **10**:nwac248. DOI:https://doi.org/10.1093/nsr/nwac248.
109. Jiwanti, P.K., Natsui, K., Nakata, K. et al. (2016). Selective production of methanol by the electrochemical reduction of CO_2 on boron-doped diamond electrodes in aqueous ammonia solution. *RSC Adv.* **6**:102214–102217. DOI:https://doi.org/10.1039/C6RA20466J.
110. Liu, Y., Zhang, Y., Cheng, K. et al. (2017). Selective electrochemical reduction of carbon dioxide to ethanol on a boron- and nitrogen-co-doped nanodiamond. *Angew. Chem., Int. Ed.* **56**:15607–15611. DOI:https://doi.org/10.1002/anie.201706311.
111. Mou, S., Wu, T., Xie, J. et al. (2019). Boron phosphide nanoparticles: a nonmetal catalyst for high-selectivity electrochemical reduction of CO_2 to CH_3OH. *Adv. Mater.* **31**:1903499. DOI:https://doi.org/10.1002/adma.201903499.
112. Xu, H., Rebollar, D., He, H. et al. (2020). Highly selective electrocatalytic CO_2 reduction to ethanol by metallic clusters dynamically formed from atomically dispersed copper. *Nat. Energy* **5**:623–632. DOI:https://doi.org/10.1038/s41560-020-0666-x.
113. Xu, F., Feng, B., Shen, Z. et al. (2024). Oxygen-bridged Cu binuclear sites for efficient electrocatalytic CO_2 reduction to ethanol at ultralow overpotential. *J. Am. Chem. Soc.* **146**:9365–9374. DOI:https://doi.org/10.1021/jacs.4c01610.
114. Esmaeilirad, M., Kondori, A., Shan, N. et al. (2022). Efficient electrocatalytic conversion of CO_2 to ethanol enabled by imidazolium-functionalized ionomer confined molybdenum phosphide. *Appl. Catal., B* **317**:121681. DOI:https://doi.org/10.1016/j.apcatb.2022.121681.
115. Du, J., Li, S., Liu, S. et al. (2020). Selective electrochemical reduction of carbon dioxide to ethanol via a relay catalytic platform. *Chem. Sci.* **11**:5098–5104. DOI:https://doi.org/10.1039/D0SC01133A.
116. Lv, K., Fan, Y., Zhu, Y. et al. (2018). Elastic Ag-anchored N-doped graphene/carbon foam for the selective electrochemical reduction of carbon dioxide to ethanol. *J. Mater. Chem. A* **6**:5025–5031. DOI:https://doi.org/10.1039/C7TA10802H.
117. Guo, F., Liu, B., Liu, M. et al. (2021). Selective electrocatalytic CO_2 reduction to acetate on polymeric Cu−L (L = pyridinic N and carbonyl group) complex core−shell microspheres. *Green Chem.* **23**:5129–5137. DOI:https://doi.org/10.1039/D1GC01464A.
118. Sarkar, S., Raj, J., Bagchi, D. et al. (2023). Structural ordering enhances highly selective production of acetic acid from CO_2 at ultra-low potential. *EES Catal.* **1**:162–170. DOI:https://doi.org/10.1039/D2EY00081D.
119. Zhu, H.L., Huang, J.R., Zhang, M.D. et al. (2024). Continuously producing highly concentrated and pure acetic acid aqueous solution via direct electroreduction of CO_2. *J. Am. Chem. Soc.* **146**:1144–1152. DOI:https://doi.org/10.1021/jacs.3c12423.
120. Zha, B., Li, C. and Li, J. (2020). Efficient electrochemical reduction of CO_2 into formate and acetate in polyoxometalate catholyte with indium catalyst. *J. Catal.* **382**:69–76. DOI:https://doi.org/10.1016/j.jcat.2019.12.010.
121. Fu, J., Zhu, W., Chen, Y. et al. (2019). Bipyridine-assisted assembly of Au nanoparticles on Cu nanowires to enhance the electrochemical reduction of CO_2. *Angew. Chem., Int. Ed.* **58**:14100–14103. DOI:https://doi.org/10.1002/anie.201905318.
122. Yin, J., Yin, Z., Jin, J. et al. (2021). A new hexagonal cobalt nanosheet catalyst for selective CO_2 conversion to ethanal. *J. Am. Chem. Soc.* **143**:15335–15343. DOI:https://doi.org/10.1021/jacs.1c06877.
123. Wang, L., Higgins, D.C., Ji, Y. et al. (2020). Selective reduction of CO to acetaldehyde with CuAg electrocatalysts. *Proc. Natl. Acad. Sci. USA* **117**:12572–12575. DOI:https://doi.org/10.1073/pnas.1821683117.
124. Hori, Y., Wakebe, H., Tsukamoto, T. et al. (1994). Electrocatalytic process of CO selectivity in electrochemical reduction of CO_2 at metal electrodes in aqueous media. *Electrochim. Acta* **39**:1833–1839. DOI:https://doi.org/10.1016/0013-4686(94)85172-7.
125. Geioushy, R.A., Khaled, M.M., Alhooshani, K. et al. (2017). Graphene/ZnO/Cu_2O electrocatalyst for selective conversion of CO_2 into n-propanol. *Electrochim. Acta* **245**:456–462. DOI:https://doi.org/10.1016/j.electacta.2017.
126. Zhang, R., Zhang, J., Wang, S. et al. (2024). Synthesis of n-propanol from CO_2 electroreduction on bicontinuous Cu_2O/Cu nanodomains. *Angew. Chem., Int. Ed.* **63**:e202405733. DOI:https://doi.org/10.1002/anie.202405733.
127. Wu, G., Song, Y., Zheng, Q. et al. (2022). Selective electroreduction of CO_2 to n-propanol in two-step tandem catalytic system. *Adv. Energy Mater.* **12**:2202054. DOI:https://doi.org/10.1002/aenm.202202054.
128. Sakamoto, N., Sekizawa, K., Shirai, S. et al. (2024). Dinuclear Cu(I) molecular electrocatalyst for CO_2-to-C_3 product conversion. *Nat. Catal.* **7**:574–584. DOI:https://doi.org/10.1038/s41929-024-01147-y.
129. Francis, S.A., Velazquez, J.M., Ferrer, I.M. et al. (2018). Reduction of aqueous CO_2 to 1-propanol at MoS_2 electrodes. *Chem. Mater.* **30**:4902–4908. DOI:https://doi.org/10.1021/acs.chemmater.7b04428.
130. Ting, L.R.L., García-Muelas, R., Martín, A.J. et al. (2020). Electrochemical reduction of carbon dioxide to 1-butanol on oxide-derived copper. *Angew. Chem., Int. Ed.* **59**:21072–21079. DOI:https://doi.org/10.1002/anie.202008289.
131. Zhu, X. and Li, Y. (2019). Review of two-dimensional materials for electrochemical CO_2 reduction from a theoretical perspective. *WIREs Comput. Mol. Sci.* **9**:e1416. DOI:https://doi.org/10.1002/wcms.1416.
132. Zhang, J., Cai, W., Hu, F.X. et al. (2021). Recent advances in single atom catalysts for the electrochemical carbon dioxide reduction reaction. *Chem. Sci.* **12**:6800–6819. DOI:https://doi.org/10.1039/d1sc01375k.
133. Meng, Y., Li, K., Xiao, D. et al. (2020). High selective and efficient Fe_2–N_6 sites for CO_2 electroreduction: a theoretical investigation. *Int. J. Hydrogen Energy* **45**:14311–14319. DOI:https://doi.org/10.1016/j.ijhydene.2020.03.134.
134. Vasileff, A., Xu, C., Jiao, Y. et al. (2018). Surface and interface engineering in copper-based bimetallic materials for selective CO_2 electroreduction. *Chem* **4**:1809–1831. DOI:https://doi.org/10.1016/j.chempr.2018.05.001.
135. Roy, D., Mandal, S.C. and Pathak, B. (2021). Machine learning-driven high-throughput screening of alloy-based catalysts for selective CO_2 hydrogenation to methanol. *ACS Appl. Mater. Interfaces* **13**:56151–56163. DOI:https://doi.org/10.1021/acsami.1c16696.
136. Jain, A., Ong, S.P., Chen, W. et al. (2015). FireWorks: a dynamic workflow system designed for high-throughput applications. *Concurr. Comp-Pract. E.* **27**:5037–5059. DOI:https://doi.org/10.1002/cpe.3505.
137. Hjorth Larsen, A., Jørgen Mortensen, J., Blomqvist, J. et al. (2017). The atomic simulation environment-a Python library for working with atoms. *J. Phys. Condens. Matter* **29**:273002. DOI:https://doi.org/10.1088/1361-648X/aa680e.

www.the-innovation.org

138. Wu, Q., Pan, M., Zhang, S. et al. (2022). Research progress in high-throughput screening of CO_2 reduction catalysts. *Energies* **15**:6666. DOI:https://doi.org/10.3390/en15186666.
139. Strasser, P., Fan, Q., Devenney, M. et al. (2003). High throughput experimental and theoretical predictive screening of materials - a comparative study of search strategies for new fuel cell anode catalysts. *J. Phys. Chem. B* **107**:11013–11021. DOI:https://doi.org/10.1021/jp030508z.
140. Greeley, J., Jaramillo, T.F., Bonde, J. et al. (2006). Computational high-throughput screening of electrocatalytic materials for hydrogen evolution. *Nat. Mater.* **5**:909–913. DOI:https://doi.org/10.1038/nmat1752.
141. Greeley, J. and Nørskov, J.K. (2007). Large-scale, density functional theory-based screening of alloys for hydrogen evolution. *Surf. Sci.* **601**:1590–1598. DOI:https://doi.org/10.1016/j.susc.2007.01.037.
142. Li, F., Ai, H., Shi, C. et al. (2021). Single transition metal atom catalysts on Ti_2CN_2 for efficient CO_2 reduction reaction. *Int. J. Hydrogen Energy* **46**:12886–12896. DOI:https://doi.org/10.1016/j.ijhydene.2021.01.096.
143. Zhao, C., Su, X., Wang, S. et al. (2022). Single-atom catalysts on supported silicomolybdic acid for CO_2 electroreduction: a DFT prediction. *J. Mater. Chem. A* **10**:6178–6186. DOI:https://doi.org/10.1039/d1ta08285j.
144. Norskov, J.K., Rossmeisl, J., Logadottir, A. et al. (2004). Origin of the overpotential for oxygen reduction at a fuel-cell cathode. *J. Phys. Chem. B* **108**:17886–17892. DOI:https://doi.org/10.1021/jp047349j.
145. Peterson, A.A., Abild-Pedersen, F., Studt, F. et al. (2010). How copper catalyzes the electroreduction of carbon dioxide into hydrocarbon fuels. *Energy Environ. Sci.* **3**:1311–1315. DOI:https://doi.org/10.1039/c0ee00071j.
146. Zhi, X., Jiao, Y., Zheng, Y. et al. (2020). Selectivity roadmap for electrochemical CO_2 reduction on copper-based alloy catalysts. *Nano Energy* **71**:104601. DOI:https://doi.org/10.1016/j.nanoen.2020.104601.
147. Greeley, J. (2016). Theoretical heterogeneous catalysis: scaling relationships and computational catalyst design. *Annu. Rev. Chem. Biomol. Eng.* **7**:605–635. DOI:https://doi.org/10.1146/annurev-chembioeng-080615-034413.
148. Xiao, Y., Shen, C. and Hadaeghi, N. (2021). Quantum mechanical screening of 2D MBenes for the electroreduction of CO_2 to C_1 hydrocarbon fuels. *J. Phys. Chem. Lett.* **12**:6370–6382. DOI:https://doi.org/10.1021/acs.jpclett.1c01499.
149. Xing, M., Zhang, Y., Li, S. et al. (2022). Prediction of carbon dioxide reduction catalyst using machine learning with a few-feature model: WLEDZ. *J. Phys. Chem. C* **126**:17025–17035. DOI:https://doi.org/10.1021/acs.jpcc.2c02161.
150. Wei, X., Cao, S., Wei, S. et al. (2022). Theoretical investigation on electrocatalytic reduction of CO_2 to methanol and methane by bimetallic atoms $TM_1/TM_{2\text{-}N}$@Gra (TM = Fe, Co, Ni, Cu). *Appl. Surf. Sci.* **593**:153377. DOI:https://doi.org/10.1016/j.apsusc.2022.153377.
151. Zhang, Y., Yao, Z., Yang, Y. et al. (2024). Breaking the scaling relations of effective CO_2 electrochemical reduction in diatomic catalysts by adjusting the flow direction of intermediate structures. *Chem. Sci.* **15**:13160–13172. DOI:https://doi.org/10.1039/D4SC03085K.
152. Wu, Q., Pan, M., Zhang, S. et al. (2022). Research progress in high-throughput screening of CO_2 reduction catalysts. *Energies* **15**:6666. DOI:https://doi.org/10.3390/en15186666.
153. Li, J., Halldin Stenlid, J., Tang, M.T. et al. (2022). Screening binary alloys for electrochemical CO_2 reduction towards multi-carbon products. *J. Mater. Chem. A* **10**:16171–16181. DOI:https://doi.org/10.1039/D2TA02749F.
154. Luo, W., Nie, X., Janik, M.J. et al. (2015). Facet dependence of CO_2 reduction paths on Cu electrodes. *ACS Catal.* **6**:219–229. DOI:https://doi.org/10.1021/acscatal.5b01967.
155. Shah, A.H., Wang, Y., Woldu, A.R. et al. (2018). Revisiting electrochemical reduction of CO_2 on Cu electrode: where do we stand about the intermediates? *J. Phys. Chem. C* **122**:18528–18536. DOI:https://doi.org/10.1021/acs.jpcc.8b05348.
156. Kastlunger, G., Wang, L., Govindarajan, N. et al. (2022). Using pH dependence to understand mechanisms in electrochemical CO reduction. *ACS Catal.* **12**:4344–4357. DOI:https://doi.org/10.1021/acscatal.1c05520.
157. Behrendt, D., Banerjee, S., Clark, C. et al. (2023). High-throughput computational screening of bioinspired dual-atom alloys for CO_2 activation. *J. Am. Chem. Soc.* **145**:4730–4735. DOI:https://doi.org/10.1021/jacs.2c13253.
158. Shi, T.T., Liu, G.Y. and Chen, Z.X. (2023). Machine learning prediction of CO adsorption energies and properties of layered alloys using an improved feature selection algorithm. *J. Phys. Chem. C* **127**:9573–9583. DOI:https://doi.org/10.1021/acs.jpcc.2c09020.
159. Wei, B., Xiong, Y., Zhang, Z. et al. (2021). Efficient electrocatalytic reduction of CO_2 to HCOOH by bimetallic In-Cu nanoparticles with controlled growth facet. *Appl. Catal., B* **283**:119646. DOI:https://doi.org/10.1016/j.apcatb.2020.119646.
160. Sun, Z., Yin, H., Liu, K. et al. (2022). Machine learning accelerated calculation and design of electrocatalysts for CO_2 reduction. *SmartMat* **3**:68–83. DOI:https://doi.org/10.1002/smm2.1107.
161. Hu, E., Liu, C., Zhang, W. et al. (2023). Machine learning assisted understanding and discovery of CO_2 reduction reaction electrocatalyst. *J. Phys. Chem. C* **127**:882–893. DOI:https://doi.org/10.1021/acs.jpcc.2c08343.
162. Roy, D., Das, A., Manna, S. et al. (2023). A route map of machine learning approaches in heterogeneous CO_2 reduction reaction. *J. Phys. Chem. C* **127**:871–881. DOI:https://doi.org/10.1021/acs.jpcc.2c06924.
163. Tran, K. and Ulissi, Z.W. (2018). Active learning across intermetallics to guide discovery of electrocatalysts for CO_2 reduction and H_2 evolution. *Nat. Catal.* **1**:696–703. DOI:https://doi.org/10.1038/s41929-018-0142-1.
164. Zhong, M., Tran, K., Min, Y. et al. (2020). Accelerated discovery of CO_2 electrocatalysts using active machine learning. *Nature* **581**:178–183. DOI:https://doi.org/10.1038/s41586-020-2242-8.
165. Yu, L., Li, F., Huang, J. et al. (2023). Double-atom catalysts featuring inverse sandwich structure for CO_2 reduction reaction: a synergetic first-principles and machine learning investigation. *ACS Catal.* **13**:9616–9628. DOI:https://doi.org/10.1021/acscatal.3c01584.
166. Ma, X., Li, Z., Achenie, L.E.K. et al. (2015). Machine-learning-augmented chemisorption model for CO_2 electroreduction catalyst screening. *J. Phys. Chem. Lett.* **6**:3528–3533. DOI:https://doi.org/10.1021/acs.jpclett.5b01660.
167. Liu, J., Luo, W., Wang, L. et al. (2022). Toward excellence of electrocatalyst design by emerging descriptor-oriented machine learning. *Adv. Funct. Mater.* **32**:2110748. DOI:https://doi.org/10.1002/adfm.202110748.
168. Wu, D., Zhang, J., Cheng, M.J. et al. (2021). Machine learning investigation of supplementary adsorbate influence on copper for enhanced electrochemical CO_2 reduction performance. *J. Phys. Chem. C* **125**:15363–15372. DOI:https://doi.org/10.1021/acs.jpcc.1c05004.
169. Xi, S., Zhao, P., He, C. et al. (2024). High-throughput screening of single-atom catalysts on 1 T-TMD for highly active and selective CO_2 reduction reaction: computational and machine learning insights. *J. Catal.* **436**:115610. DOI:https://doi.org/10.1016/j.jcat.2024.115610.
170. Rittiruam, M., Khamloet, P., Ektarawong, A. et al. (2024). Screening of Cu-Mn-Ni-Zn high-entropy alloy catalysts for CO_2 reduction reaction by machine-learning-accelerated density functional theory. *Appl. Surf. Sci.* **652**:159297. DOI:https://doi.org/10.1016/j.apsusc.2024.159297.
171. Sun, H. and Liu, J.y. (2024). Advancing CO_2RR with O-coordinated single-atom nanozymes: a DFT and machine learning exploration. *ACS Catal.* **14**:14021–14030. DOI:https://doi.org/10.1021/acscatal.4c02799.
172. Chen, A., Zhang, X., Chen, L. et al. (2020). A machine learning model on simple features for CO_2 reduction electrocatalysts. *J. Phys. Chem. C* **124**:22471–22478. DOI:https://doi.org/10.1021/acs.jpcc.0c05964.
173. Gusarov, S., Stoyanov, S.R. and Siahrostami, S. (2020). Development of fukui function based descriptors for a machine learning study of CO_2 reduction. *J. Phys. Chem. C* **124**:10079–10084. DOI:https://doi.org/10.1021/acs.jpcc.0c03101.
174. Ding, H., Shi, Y., Li, Z. et al. (2023). Active learning accelerating to screen dual-metal-site catalysts for electrochemical carbon dioxide reduction reaction. *ACS Appl. Mater. Interfaces* **15**:12986–12997. DOI:https://doi.org/10.1021/acsami.2c21332.
175. Gao, W., Chen, Y., Li, B. et al. (2020). Determining the adsorption energies of small molecules with the intrinsic properties of adsorbates and substrates. *Nat. Commun.* **11**:1196. DOI:https://doi.org/10.1038/s41467-020-14969-8.
176. Yang, Z., Gao, W. and Jiang, Q. (2020). A machine learning scheme for the catalytic activity of alloys with intrinsic descriptors. *J. Mater. Chem. A* **8**:17507–17515. DOI:https://doi.org/10.1039/D0TA06203K.
177. Roy, D., Mandal, S.C. and Pathak, B. (2022). Machine learning assisted exploration of high entropy alloy-based catalysts for selective CO_2 reduction to methanol. *J. Phys. Chem. Lett.* **13**:5991–6002. DOI:https://doi.org/10.1021/acs.jpclett.2c00929.
178. Pedersen, J.K., Batchelor, T.A.A., Bagger, A. et al. (2020). High-entropy alloys as catalysts for the CO_2 and CO reduction reactions. *ACS Catal.* **10**:2169–2176. DOI:https://doi.org/10.1021/acscatal.9b04343.
179. Mok, D.H. and Back, S. (2021). Atomic structure-free representation of active motifs for expedited catalyst discovery. *J. Chem. Inf. Model.* **61**:4514–4520. DOI:https://doi.org/10.1021/acs.jcim.1c00726.
180. Noh, J., Back, S., Kim, J. et al. (2018). Active learning with non-ab initio input features toward efficient CO_2 reduction catalysts. *Chem. Sci.* **9**:5152–5159. DOI:https://doi.org/10.1039/c7sc03422a.
181. Amaral, P.H.R., Torrez-Baptista, A.D., Dionisio, D. et al. (2022). A machine learning model for adsorption energies of chemical species applied to CO_2 electroreduction. *J. Electrochem. Soc.* **169**:116505. DOI:https://doi.org/10.1149/1945-7111/ac9f7a.
182. Xie, T. and Grossman, J.C. (2018). Crystal graph convolutional nural networks for an accurate and interpretable prediction of material properties. *Phys. Rev. Lett.* **120**:145301. DOI:https://doi.org/10.1103/PhysRevLett.120.145301.
183. Back, S., Yoon, J., Tian, N. et al. (2019). Convolutional neural network of atomic surface structures to predict binding energies for high-throughput screening of catalysts. *J. Phys. Chem. Lett.* **10**:4401–4408. DOI:https://doi.org/10.1021/acs.jpclett.9b01428.
184. Gu, G.H., Noh, J., Kim, S. et al. (2020). Practical deep-learning representation for fast heterogeneous catalyst screening. *J. Phys. Chem. Lett.* **11**:3185–3191. DOI:https://doi.org/10.1021/acs.jpclett.0c00634.
185. Chanussot, L., Das, A., Goyal, S. et al. (2021). Open catalyst 2020 (OC20) dataset and community challenges. *ACS Catal.* **11**:6059–6072. DOI:https://doi.org/10.1021/acscatal.0c04525.
186. Tran, R., Lan, J., Shuaibi, M. et al. (2023). The open oatalyst 2022 (OC22) dataset and challenges for oxide electrocatalysts. *ACS Catal.* **13**:3066–3084. DOI:https://doi.org/10.1021/acscatal.2c05426.
187. Sriram, A., Choi, S., Yu, X. et al. (2024). The open DAC 2023 dataset and challenges for sorbent discovery in direct air capture. *ACS Cent. Sci.* **10**:923–941. DOI:https://doi.org/10.1021/acscentsci.3c01629.
188. Lan, J., Palizhati, A., Shuaibi, M. et al. (2023). AdsorbML: a leap in efficiency for adsorption energy calculations using generalizable machine learning potentials. *npj Comput. Mater.* **9**:172. DOI:https://doi.org/10.1038/s41524-023-01121-5.
189. Wander, B., Shuaibi, M., Kitchin, J.R. et al. (2024). CatTSunami: accelerating transition state energy calculations with pre-trained graph neural networks. Preprint at *arXiv*. DOI:https://doi.org/10.48550/arXiv.2405.02078.

190. Barroso-Luque, L., Shuaibi, M., Fu, X. et al. (2024). Open materials 2024 (OMat24) inorganic materials dataset and models. Preprint at *arXiv*. DOI:https://doi.org/10.48550/arXiv.2410.12771.
191. Kolluru, A., Shoghi, N., Shuaibi, M. et al. (2022). Transfer learning using attentions across atomic systems with graph neural networks (TAAG). *J. Chem. Phys.* **156**:184702. DOI:https://doi.org/10.1063/5.0088019.
192. Zhu, Q., Gu, Y., Liang, X. et al. (2022). A machine learning model to predict CO_2 reduction reactivity and products transferred from metal-zeolites. *ACS Catal.* **12**:12336–12348. DOI:https://doi.org/10.1021/acscatal.2c03250.
193. Gariepy, Z., Chen, G., Xu, A. et al. (2023). Machine learning assisted binary alloy catalyst design for the electroreduction of CO_2 to C_2 products. *Energy Adv.* **2**:410–419. DOI:https://doi.org/10.1039/D2YA00316C.
194. Brockherde, F., Vogt, L., Li, L. et al. (2017). Bypassing the Kohn-Sham equations with machine learning. *Nat. Commun.* **8**:872. DOI:https://doi.org/10.1038/s41467-017-00839-3.
195. Lan, J., Palizhati, A., Shuaibi, M. et al. (2023). AdsorbML: a leap in efficiency for adsorption energy calculations using generalizable machine learning potentials. *npj Comput. Mater.* **9**:172. DOI:https://doi.org/10.1038/s41524-023-01121-5.
196. Choung, S., Park, W., Moon, J. et al. (2024). Rise of machine learning potentials in heterogeneous catalysis: developments, applications, and prospects. *Chem. Eng. J.* **494**:152757. DOI:https://doi.org/10.1016/j.cej.2024.152757.
197. Schaaf, L.L., Fako, E., De, S. et al. (2023). Accurate energy barriers for catalytic reaction pathways: an automatic training protocol for machine learning force fields. *npj Comput. Mater.* **9**:180. DOI:https://doi.org/10.1038/s41524-023-01124-2.
198. Musa, E., Doherty, F. and Goldsmith, B.R. (2022). Accelerating the structure search of catalysts with machine learning. *Curr. Opin. Chem. Eng.* **35**:100771. DOI:https://doi.org/10.1016/j.coche.2021.100771.
199. Jung, H., Sauerland, L., Stocker, S. et al. (2023). Machine-learning driven global optimization of surface adsorbate geometries. *npj Comput. Mater.* **9**:114. DOI:https://doi.org/10.1038/s41524-023-01065-w.
200. Men, Y., Wu, D., Hu, Y. et al. (2023). Understanding alkaline hydrogen oxidation reaction on PdNiRuIrRh high-entropy-alloy by machine learning potential. *Angew. Chem., Int. Ed.* **62**:e202217976. DOI:https://doi.org/10.1002/anie.202217976.
201. Kim, H.J., Lee, G., Oh, S.H.V. et al. (2024). Recalibrating the experimentally derived structure of the metastable surface oxide on copper via machine learning-accelerated In silico global optimization. *ACS Nano* **18**:4559–4569. DOI:https://doi.org/10.1021/acsnano.3c12249.
202. Johannes, G., Anuroop, S., Stephan, G. et al. (2022). GemNet-OC: developing graph neural networks for large and diverse molecular simulation datasets. https://openreview.net/forum?id=u8tvSxm4Bs.
203. Zitnick, C.L. (2022). Spherical channels for modeling atomic interactions. Preprint at *arXiv*. DOI:https://doi.org/10.48550/arXiv.2206.14331.
204. Passaro, S. and Zitnick, C.L. (2023). Reducing SO_3 convolutions to SO_2 for efficient equivariant GNNs. Preprint at *arXiv*. DOI:https://doi.org/10.48550/arXiv.2302.03655.
205. Liu, X., Xiao, J., Peng, H. et al. (2017). Understanding trends in electrochemical carbon dioxide reduction rates. *Nat. Commun.* **8**:15438. DOI:https://doi.org/10.1038/ncomms15438.
206. Esterhuizen, J.A., Goldsmith, B.R. and Linic, S. (2022). Interpretable machine learning for knowledge generation in heterogeneous catalysis. *Nat. Catal.* **5**:175–184. DOI:https://doi.org/10.1038/s41929-022-00744-z.
207. Mou, T., Pillai, H.S., Wang, S. et al. (2023). Bridging the complexity gap in computational heterogeneous catalysis with machine learning. *Nat. Catal.* **6**:122–136. DOI:https://doi.org/10.1038/s41929-023-00911-w.
208. Günay, M.E., Türker, L. and Tapan, N.A. (2018). Decision tree analysis for efficient CO_2 utilization in electrochemical systems. *J. CO_2 Util.* **28**:83–95. DOI:https://doi.org/10.1016/j.jcou.2018.09.011.
209. Gao, Y., Wang, L., Chen, X. et al. (2023). Revisiting electrocatalyst design by a knowledge graph of Cu-based catalysts for CO_2 reduction. *ACS Catal.* **13**:8525–8534. DOI:https://doi.org/10.1021/acscatal.3c00759.
210. Batchelor, T.A.A., Löffler, T., Xiao, B. et al. (2021). Complex-solid-solution electrocatalyst discovery by computational prediction and high-throughput experimentation. *Angew. Chem., Int. Ed.* **60**:6932–6937. DOI:https://doi.org/10.1002/anie.202014374.
211. Park, J., Kim, Y.M., Hong, S. et al. (2023). Closed-loop optimization of nanoparticle synthesis enabled by robotics and machine learning. *Matter* **6**:677–690. DOI:https://doi.org/10.1016/j.matt.2023.01.018.
212. Szymanski, N.J., Rendy, B., Fei, Y. et al. (2023). An autonomous laboratory for the accelerated synthesis of novel materials. *Nature* **624**:86–91. DOI:https://doi.org/10.1038/s41586-023-06734-w.
213. Liu, Y., Li, X., Zhang, Y. et al. (2024). Ultra-large scale stitchless AFM: advancing nanoscale characterization and manipulation with zero stitching error and high throughput. *Small* **20**:e2303838. DOI:https://doi.org/10.1002/smll.202303838.
214. Ye, C., Raaijman, S.J., Chen, X. et al. (2022). Enhanced electrochemical CO_2 reduction to formate on poly(4-vinylpyridine)-modified copper and gold electrodes. *ACS Appl. Mater. Interfaces* **14**:45263–45271. DOI:https://doi.org/10.1021/acsami.2c10452.
215. Duan, G.Y., Li, X.Q., Ding, G.R. et al. (2022). Highly efficient electrocatalytic CO_2 reduction to C_{2+} products on a poly(ionic liquid)-based Cu^0-Cu^I tandem catalyst. *Angew. Chem., Int. Ed.* **61**:e202110657. DOI:https://doi.org/10.1002/anie.202110657.
216. Payra, S., Devaraj, N., Tarafder, K. et al. (2022). Unprecedented electroreduction of CO_2 over metal organic framework-derived intermetallic nano-alloy $Cu_{0.85}Ni_{0.15}$/C. *ACS Appl. Energy Mater.* **5**:4945–4955. DOI:https://doi.org/10.1021/acsaem.2c00330.
217. Liu, Z., Song, L., Lv, X. et al. (2024). Switching CO_2 electroreduction toward ethanol by delocalization state-tuned bond cleavage. *J. Am. Chem. Soc.* **146**:14260–14266. DOI:https://doi.org/10.1021/jacs.4c03830.
218. Kim, C., Bui, J.C., Luo, X. et al. (2021). Tailored catalyst microenvironments for CO_2 electroreduction to multicarbon products on copper using bilayer ionomer coatings. *Nat. Energy* **6**:1026–1034. DOI:https://doi.org/10.1038/s41560-021-00920-8.
219. Ma, W., Xie, S., Zhang, X.G. et al. (2019). Promoting electrocatalytic CO_2 reduction to formate via sulfur-boosting water activation on indium surfaces. *Nat. Commun.* **10**:892. DOI:https://doi.org/10.1038/s41467-019-08805-x.
220. Kim, H.-T., Park, J., Mun, J. et al. (2024). Selective electroreduction of CO_2 to C_{2+} alcohols using graphitic frustrated lewis pair catalyst. *ACS Catal.* **14**:10392–10402. DOI:https://doi.org/10.1021/acscatal.3c04275.
221. Yang, F., Ma, X., Cai, W.B. et al. (2019). Nature of oxygen-containing groups on carbon for high-efficiency electrocatalytic CO_2 reduction reaction. *J. Am. Chem. Soc.* **141**:20451–20459. DOI:https://doi.org/10.1021/jacs.9b11123.
222. Zhang, S., Kang, P., Ubnoske, S. et al. (2014). Polyethylenimine-enhanced electrocatalytic reduction of CO_2 to formate at nitrogen-doped carbon nanomaterials. *J. Am. Chem. Soc.* **136**:7845–7848. DOI:https://doi.org/10.1021/ja5031529.
223. Wu, Y., Jiang, Z., Lu, X. et al. (2019). Domino electroreduction of CO_2 to methanol on a molecular catalyst. *Nature* **575**:639–642. DOI:https://doi.org/10.1038/s41586-019-1760-8.
224. Ponnurangam, S., Yun, C.M. and Chernyshova, I.V. (2016). Robust electroreduction of CO_2 at a poly(4-vinylpyridine)-copper electrode. *Chemelectrochem* **3**:74–82. DOI:https://doi.org/10.1002/celc.201500421.
225. Zang, Y., Liu, T., Wei, P. et al. (2022). Selective CO_2 electroreduction to ethanol over a carbon-coated CuO_x catalyst. *Angew. Chem., Int. Ed.* **61**:e202209629. DOI:https://doi.org/10.1002/anie.202209629.
226. Qin, H.G., Du, Y.F., Bai, Y.Y. et al. (2023). Surface-immobilized cross-linked cationic polyelectrolyte enables CO_2 reduction with metal cation-free acidic electrolyte. *Nat. Commun.* **14**:5640. DOI:https://doi.org/10.1038/s41467-023-41396-2.
227. Gu, J., Liu, S., Ni, W. et al. (2022). Modulating electric field distribution by alkali cations for CO_2 electroreduction in strongly acidic medium. *Nat. Catal.* **5**:268–276. DOI:https://doi.org/10.1038/s41929-022-00761-y.
228. Qin, H.G., Li, F.-Z., Du, Y.F. et al. (2022). Quantitative understanding of cation effects on the electrochemical reduction of CO_2 and H^+ in acidic solution. *ACS Catal.* **13**:916–926. DOI:https://doi.org/10.1021/acscatal.2c04875.
229. Christensen, O., Zhao, S., Sun, Z. et al. (2022). Can the CO_2 reduction reaction be improved on Cu: selectivity and intrinsic activity of functionalized Cu surfaces. *ACS Catal.* **12**:15737–15749. DOI:https://doi.org/10.1021/acscatal.2c04200.
230. Nankya, R., Xu, Y., Elgazzar, A. et al. (2024). Cobalt-doped bismuth nanosheet catalyst for enhanced electrochemical CO_2 reduction to electrolyte-free formic acid. *Angew. Chem., Int. Ed.* **63**:e202403671. DOI:https://doi.org/10.1002/anie.202403671.
231. Chen, Z., Zhang, X., Jiao, M. et al. (2020). Engineering electronic structure of stannous sulfide by amino-functionalized carbon: toward efficient electrocatalytic reduction of CO_2 to formate. *Adv. Energy Mater.* **10**:1903664. DOI:https://doi.org/10.1002/aenm.201903664.
232. He, B., Jia, L., Cui, Y. et al. (2019). $SnSe_2$ nanorods on carbon cloth as a highly selective, active, and flexible electrocatalyst for electrochemical reduction of CO_2 into formate. *ACS Appl. Energy Mater.* **2**:7655–7662. DOI:https://doi.org/10.1021/acsaem.9b01643.
233. Shen, H., Jin, H., Li, H. et al. (2023). Acidic CO_2-to-HCOOH electrolysis with industrial-level current on phase engineered tin sulfide. *Nat. Commun.* **14**:2843. DOI:https://doi.org/10.1038/s41467-023-38497-3.
234. García-Muelas, R., Dattila, F., Shinagawa, T. et al. (2018). Origin of the selective electroreduction of carbon dioxide to formate by chalcogen modified copper. *J. Phys. Chem. Lett.* **9**:7153–7159. DOI:https://doi.org/10.1021/acs.jpclett.8b03212.
235. Li, H., Qin, X., Jiang, T. et al. (2019). Changing the product selectivity for electrocatalysis of CO_2 reduction reaction on plated Cu electrodes. *ChemCatChem* **11**:6139–6146. DOI:https://doi.org/10.1002/cctc.201901748.
236. Wang, J.-S., Zhao, G.-C., Qiu, Y.-Q. et al. (2021). Strong boron–carbon bonding interaction drives CO_2 reduction to ethanol over the boron-doped Cu(111) surface: an insight from the first-principles calculations. *J. Phys. Chem. C* **125**:572–582. DOI:https://doi.org/10.1021/acs.jpcc.0c09661.
237. Zheng, M., Wang, P., Zhi, X. et al. (2022). Electrocatalytic CO_2-to-C_{2+} with ampere-level current on heteroatom-engineered copper via tuning *CO intermediate coverage. *J. Am. Chem. Soc.* **144**:14936–14944. DOI:https://doi.org/10.1021/jacs.2c06820.
238. Okatenko, V., Loiudice, A., Newton, M.A. et al. (2023). Alloying as a strategy to boost the stability of copper nanocatalysts during the electrochemical CO_2 reduction reaction. *J. Am. Chem. Soc.* **145**:5370–5383. DOI:https://doi.org/10.1021/jacs.2c13437.
239. Jia, L., Sun, M., Xu, J. et al. (2021). Phase-dependent electrocatalytic CO_2 reduction on Pd_3Bi nanocrystals. *Angew. Chem., Int. Ed.* **60**:21741–21745. DOI:https://doi.org/10.1002/anie.202109288.
240. Zhang, M., Zhang, Z., Zhao, Z. et al. (2021). Tunable selectivity for electrochemical CO_2 reduction by bimetallic Cu–Sn catalysts: elucidating the roles of Cu and Sn. *ACS Catal.* **11**:11103–11108. DOI:https://doi.org/10.1021/acscatal.1c02556.
241. Zheng, T., Liu, C., Guo, C. et al. (2021). Copper-catalysed exclusive CO_2 to pure formic acid conversion via single-atom alloying. *Nat. Nanotechnol.* **16**:1386–1393. DOI:https://doi.org/10.1038/s41565-021-00974-5.
242. Clark, E.L., Hahn, C., Jaramillo, T.F. et al. (2017). Electrochemical CO_2 reduction over compressively strained CuAg surface alloys with enhanced multi-carbon oxygenate selectivity. *J. Am. Chem. Soc.* **139**:15848–15857. DOI:https://doi.org/10.1021/jacs.7b08607.

243. Lu, L., Sun, X., Ma, J. et al. (2018). Highly efficient electroreduction of CO_2 to methanol on palladium−copper bimetallic aerogels. *Angew. Chem., Int. Ed.* **57**:14149−14153. DOI:https://doi.org/10.1002/anie.201808964.
244. Bagchi, D., Raj, J., Singh, A.K. et al. (2022). Structure-tailored surface oxide on Cu−Ga intermetallics enhances CO_2 reduction selectivity to methanol at ultralow potential. *Adv. Mater.* **34**:2109426. DOI:https://doi.org/10.1002/adma.202109426.
245. Jia, H., Yang, Y., Chow, T.H. et al. (2021). Symmetry-broken Au−Cu heterostructures and their tandem catalysis process in electrochemical CO_2 reduction. *Adv. Funct. Mater.* **31**:2101255. DOI:https://doi.org/10.1002/adfm.202101255.
246. Ma, Y., Yu, J., Sun, M. et al. (2022). Confined growth of silver-copper janus nanostructures with {100} facets for highly selective tandem electrocatalytic carbon dioxide reduction. *Adv. Mater.* **34**:e2110607. DOI:https://doi.org/10.1002/adma.202110607.
247. Rahaman, M., Kiran, K., Montiel, I.Z. et al. (2020). Selective n-propanol formation from CO_2 over degradation-resistant activated PdCu alloy foam electrocatalysts. *Green Chem.* **22**:6497−6509. DOI:https://doi.org/10.1039/D0GC01636E.
248. Ren, D., Ang, B.S.H. and Yeo, B.S. (2016). Tuning the selectivity of carbon dioxide electroreduction toward ethanol on oxide-derived Cu_xZn catalysts. *ACS Catal.* **6**:8239−8247. DOI:https://doi.org/10.1021/acscatal.6b02162.
249. Louisia, S., Kim, D., Li, Y. et al. (2022). The presence and role of the intermediary CO reservoir in heterogeneous electroreduction of CO_2. *Proc. Natl. Acad. Sci. USA* **119**:e2201922119. DOI:https://doi.org/10.1073/pnas.2201922119.
250. Nitopi, S., Bertheussen, E., Scott, S.B. et al. (2019). Progress and perspectives of electrochemical CO_2 reduction on copper in aqueous electrolyte. *Chem. Rev.* **119**:7610−7672. DOI:https://doi.org/10.1021/acs.chemrev.8b00705.
251. Iyengar, P., Kolb, M.J., Pankhurst, J.R. et al. (2021). Elucidating the facet-dependent selectivity for CO_2 electroreduction to ethanol of Cu−Ag tandem catalysts. *ACS Catal.* **11**:4456−4463. DOI:https://doi.org/10.1021/acscatal.1c00420.
252. Albo, J., Perfecto-Irigaray, M., Beobide, G. et al. (2019). Cu/Bi metal-organic framework-based systems for an enhanced electrochemical transformation of CO_2 to alcohols. *J. CO_2 Util.* **33**:157−165. DOI:https://doi.org/10.1016/j.jcou.2019.05.025.
253. Lum, Y. and Ager, J.W. (2018). Sequential catalysis controls selectivity in electrochemical CO_2 reduction on Cu. *Energy Environ. Sci.* **11**:2935−2944. DOI:https://doi.org/10.1039/C8EE01501E.
254. Wu, G., Song, Y., Zheng, Q. et al. (2022). Selective electroreduction of CO_2 to n-propanol in two-step tandem catalytic system. *Adv. Energy Mater.* **12**:2202054. DOI:https://doi.org/10.1002/aenm.202202054.
255. Ozden, A., García de Arquer, F.P., Huang, J.E. et al. (2022). Carbon-efficient carbon dioxide electrolysers. *Nat. Sustain.* **5**:563−573. DOI:https://doi.org/10.1038/s41893-022-00879-8.
256. Fan, L., Zhao, Y., Chen, L. et al. (2023). Selective production of ethylene glycol at high rate via cascade catalysis. *Nat. Catal.* **6**:585−595. DOI:https://doi.org/10.1038/s41929-023-00977-6.
257. An, X., Li, S., Hao, X. et al. (2021). Common strategies for improving the performances of tin and bismuth-based catalysts in the electrocatalytic reduction of CO_2 to formic acid/formate. *Renew. Sustain. Energy Rev.* **143**:110952. DOI:https://doi.org/10.1016/j.rser.2021.110952.
258. Du, X., Qin, Y., Gao, B. et al. (2021). Plasma-assisted and oxygen vacancy-engineered In_2O_3 films for enhanced electrochemical reduction of CO_2. *Appl. Surf. Sci.* **563**:150405. DOI:https://doi.org/10.1016/j.apsusc.2021.150405.
259. Niu, Z., Gao, X., Lou, S. et al. (2023). Theory-guided S-defects boost selective conversion of CO_2 to HCOOH over In_4SnS_8 nanoflowers. *ACS Catal.* **13**:2998−3006. DOI:https://doi.org/10.1021/acscatal.2c05957.
260. Guo, C., Guo, Y., Shi, Y. et al. (2022). Electrocatalytic reduction of CO_2 to ethanol at close to theoretical potential via engineering abundant electron-donating $Cu^{\delta+}$ species. *Angew. Chem., Int. Ed.* **61**:e202205909. DOI:https://doi.org/10.1002/anie.202205909.
261. Zhao, S., Liu, A., Li, Y. et al. (2022). Boosting the electrocatalytic CO_2 reduction reaction by nanostructured metal materials via defects engineering. *Nanomaterials* **12**:2389. DOI:https://doi.org/10.3390/nano12142389.
262. Zhang, X., Sun, X., Guo, S.X. et al. (2019). Formation of lattice-dislocated bismuth nanowires on copper foam for enhanced electrocatalytic CO_2 reduction at low overpotential. *Energy Environ. Sci.* **12**:1334−1340. DOI:https://doi.org/10.1039/C9EE00018F.
263. Kumar, B., Atla, V., Brian, J.P. et al. (2017). Reduced SnO_2 porous nanowires with a high density of grain boundaries as catalysts for efficient electrochemical CO_2-into-HCOOH conversion. *Angew. Chem., Int. Ed.* **56**:3645−3649. DOI:https://doi.org/10.1002/anie.201612194.
264. Wu, Z., Jing, H., Zhao, Y. et al. (2023). Grain boundary and interface interaction Co-regulation promotes SnO_2 quantum dots for efficient CO_2 reduction. *Chem. Eng. J.* **451**:138477. DOI:https://doi.org/10.1016/j.cej.2022.138477.
265. Oh, J.M., Venters, C.C., Di, C. et al. (2020). U1 snRNP regulates cancer cell migration and invasion *in vitro*. *Nat. Commun.* **11**:1. DOI:https://doi.org/10.1038/s41467-019-13993-7.
266. Bagger, A., Ju, W., Varela, A.S. et al. (2019). Electrochemical CO_2 reduction: classifying Cu facets. *ACS Catal.* **9**:7894−7899. DOI:https://doi.org/10.1021/acscatal.9b01899.
267. Zheng, Y., Vasileff, A., Zhou, X. et al. (2019). Understanding the roadmap for electrochemical reduction of CO_2 to multi-carbon oxygenates and hydrocarbons on copper-based catalysts. *J. Am. Chem. Soc.* **141**:7646−7659. DOI:https://doi.org/10.1021/jacs.9b02124.
268. Huang, Y., Chen, Y., Cheng, T. et al. (2018). Identification of the selective sites for electrochemical reduction of CO to C_{2+} products on copper nanoparticles by combining reactive force fields, density functional theory, and machine learning. *ACS Energy Lett.* **3**:2983−2988. DOI:https://doi.org/10.1021/acsenergylett.8b01933.
269. Cheng, D., Zhao, Z.J., Zhang, G. et al. (2021). The nature of active sites for carbon dioxide electroreduction over oxide-derived copper catalysts. *Nat. Commun.* **12**:395. DOI:https://doi.org/10.1038/s41467-020-20615-0.
270. Kim, J.Y., Park, W., Choi, C. et al. (2021). High facets on nanowrinkled Cu via chemical vapor deposition graphene growth for efficient CO_2 reduction into ethanol. *ACS Catal.* **11**:5658−5665. DOI:https://doi.org/10.1021/acscatal.0c05263.
271. Won, D.H., Choi, C.H., Chung, J. et al. (2015). Rational design of a hierarchical tin dendrite electrode for efficient electrochemical reduction of CO_2. *ChemSusChem* **8**:3092−3098. DOI:https://doi.org/10.1002/cssc.201500694.
272. Lee, C.H. and Kanan, M.W. (2015). Controlling H^+ vs CO_2 reduction selectivity on Pb electrodes. *ACS Catal.* **5**:465−469. DOI:https://doi.org/10.1021/cs5017672.
273. Popić, J.P., Avramov-Ivić, M.L. and Vuković, N.B. (1997). Reduction of carbon dioxide on ruthenium oxide and modified ruthenium oxide electrodes in 0.5 M $NaHCO_3$. *J. Electroanal. Chem.* **421**:105−110. DOI:https://doi.org/10.1016/S0022-0728(96)04823-1.
274. Tayyebi, E., Hussain, J., Abghoui, Y. et al. (2018). Trends of electrochemical CO_2 reduction reaction on transition metal oxide catalysts. *J. Phys. Chem. C* **122**:10078−10087. DOI:https://doi.org/10.1021/acs.jpcc.8b02224.
275. Chen, Z., Gao, M.R., Duan, N. et al. (2020). Tuning adsorption strength of CO_2 and its intermediates on tin oxide-based electrocatalyst for efficient CO_2 reduction towards carbonaceous products. *Appl. Catal., B* **277**:119252. DOI:https://doi.org/10.1016/j.apcatb.2020.119252.
276. Favaro, M., Xiao, H., Cheng, T. et al. (2017). Subsurface oxide plays a critical role in CO_2 activation by Cu(111) surfaces to form chemisorbed CO_2, the first step in reduction of CO_2. *Proc. Natl. Acad. Sci. USA* **114**:6706−6711. DOI:https://doi.org/10.1073/pnas.1701405114.
277. Zhou, X., Shan, J., Chen, L. et al. (2022). Stabilizing Cu^{2+} ions by solid solutions to promote CO_2 electroreduction to methane. *J. Am. Chem. Soc.* **144**:2079−2084. DOI:https://doi.org/10.1021/jacs.1c12212.
278. Wang, D., Li, L., Xia, Q. et al. (2022). Boosting CO_2 electroreduction to multicarbon products via tuning of the copper surface charge. *ACS Sustainable Chem. Eng.* **10**:11451−11458. DOI:https://doi.org/10.1021/acssuschemeng.2c03963.
279. Arán-Ais, R.M., Scholten, F., Kunze, S. et al. (2020). The role of *in situ* generated morphological motifs and Cu^I species in C_{2+} product selectivity during CO_2 pulsed electroreduction. *Nat. Energy* **5**:317−325. DOI:https://doi.org/10.1038/s41560-020-0594-9.
280. Bai, H., Cheng, T., Li, S. et al. (2021). Controllable CO adsorption determines ethylene and methane productions from CO_2 electroreduction. *Sci. Bull.* **66**:62−68. DOI:https://doi.org/10.1016/j.scib.2020.06.023.
281. Mistry, H., Varela, A.S., Bonifacio, C.S. et al. (2016). Highly selective plasma-activated copper catalysts for carbon dioxide reduction to ethylene. *Nat. Commun.* **7**:12123. DOI:https://doi.org/10.1038/ncomms12123.
282. Zhang, X.D., Liu, T., Liu, C. et al. (2023). Asymmetric low-frequency pulsed strategy enables ultralong CO_2 reduction stability and controllable product selectivity. *J. Am. Chem. Soc.* **145**:2195−2206. DOI:https://doi.org/10.1021/jacs.2c09501.
283. Zhang, W., Jin, Z. and Chen, Z. (2022). Rational-designed principles for electrochemical and photoelectrochemical upgrading of CO_2 to value-added chemicals. *Adv. Sci.* **9**:2105204. DOI:https://doi.org/10.1002/advs.202105204.
284. He, X., Lin, L., Li, X. et al. (2024). Roles of copper(I) in water-promoted CO_2 electrolysis to multi-carbon compounds. *Nat. Commun.* **15**:9923. DOI:https://doi.org/10.1038/s41467-024-54282-2.
285. Song, Y., Chen, W., Zhao, C. et al. (2017). Metal-free nitrogen-doped mesoporous carbon for electroreduction of CO_2 to ethanol. *Angew. Chem., Int. Ed.* **56**:10840−10844. DOI:https://doi.org/10.1002/anie.201706777.
286. Li, Z., Sun, B., Xiao, D. et al. (2025). Mesostructure-specific configuration of *CO adsorption for selective CO_2 electroreduction to C_{2+} products. *Angew. Chem., Int. Ed.* **64**:e202413832. DOI:https://doi.org/10.1002/anie.202413832.
287. Kim, Y., Yun, G.T., Kim, M. et al. (2024). Effect of feature shape and dimension of a confinement geometry on selectivity of electrocatalytic CO_2 reduction. *Angew. Chem., Int. Ed* **63**:e202316264. DOI:https://doi.org/10.1002/anie.202316264.
288. Dong, J., Liu, Y., Pei, J. et al. (2023). Continuous electroproduction of formate via CO_2 reduction on local symmetry-broken single-atom catalysts. *Nat. Commun.* **14**:6849. DOI:https://doi.org/10.1038/s41467-023-42539-1.
289. Wordsworth, J., Benedetti, T.M., Somerville, S.V. et al. (2022). The influence of nanoconfinement on electrocatalysis. *Angew. Chem., Int. Ed.* **61**:e202200755. DOI:https://doi.org/10.1002/anie.202200755.
290. Song, Y., Chen, W., Zhao, C. et al. (2017). Metal-free nitrogen-doped mesoporous carbon for electroreduction of CO_2 to ethanol. *Angew. Chem. Int. Ed.* **56**:10840−10844. DOI:https://doi.org/10.1002/anie.201706777.
291. Yang, P.P., Zhang, X.L., Gao, F.Y. et al. (2020). Protecting copper oxidation state via intermediate confinement for selective CO_2 electroreduction to C_{2+} fuels. *J. Am. Chem. Soc.* **142**:6400−6408. DOI:https://doi.org/10.1021/jacs.0c01699.
292. Calle-Vallejo, F., Tymoczko, J., Colic, V. et al. (2015). Finding optimal surface sites on heterogeneous catalysts by counting nearest neighbors. *Science* **350**:185−189. DOI:https://doi.org/10.1126/science.aab3501.
293. Fei, H., Dong, J., Feng, Y. et al. (2018). General synthesis and definitive structural identification of MN_4C_4 single-atom catalysts with tunable electrocatalytic activities. *Nat. Catal.* **1**:63−72. DOI:https://doi.org/10.1038/s41929-017-0008-y.
294. Li, Z., Ji, S., Liu, Y. et al. (2020). Well-defined materials for heterogeneous catalysis: from nanoparticles to isolated single-atom sites. *Chem. Rev.* **120**:623−682. DOI:https://doi.org/10.1021/acs.chemrev.9b00311.
295. Zhang, T., Han, X., Liu, H. et al. (2022). Site-specific axial oxygen coordinated FeN_4 active sites for highly selective electroreduction of carbon dioxide. *Adv. Funct. Mater.* **32**:2111446. DOI:https://doi.org/10.1002/adfm.202111446.

296. Wang, C., Lv, Z., Liu, Y. et al. (2024). Asymmetric Cu−N_1O_3 sites coupling atop-type and bridge-type adsorbed *C_1 for electrocatalytic CO_2-to-C_2 conversion. *Angew. Chem., Int. Ed.* **63**:e202411216. DOI:https://doi.org/10.1002/anie.202411216.
297. Chu, S., Yan, X., Choi, C. et al. (2020). Stabilization of Cu^+ by tuning a CuO−CeO_2 interface for selective electrochemical CO_2 reduction to ethylene. *Green Chem.* **22**:6540–6546. DOI:https://doi.org/10.1039/D0GC02279A.
298. Chen, C., Yan, X., Liu, S. et al (2020). Highly efficient electroreduction of CO_2 to C_{2+} alcohols on heterogeneous dual active sites. *Angew. Chem., Int. Ed.* **59**:16459–16464. DOI:https://doi.org/10.1002/anie.202006847.
299. Zhang, M., Lu, M., Yang, M.Y. et al. (2023). Ultrafine Cu nanoclusters confined within covalent organic frameworks for efficient electroreduction of CO_2 to CH_4 by synergistic strategy. *eScience* **3**:100116. DOI:https://doi.org/10.1016/j.esci.2023.100116.
300. Yang, Y., Fu, J., Ouyang, Y. et al. (2023). In-situ constructed Cu/CuNC interfaces for low-overpotential reduction of CO_2 to ethanol. *Natl. Sci. Rev.* **10**:nwac248. DOI:https://doi.org/10.1093/nsr/nwac248.
301. Singh, M.R., Kwon, Y., Lum, Y. et al. (2016). Hydrolysis of electrolyte cations enhances the electrochemical reduction of CO_2 over Ag and Cu. *J. Am. Chem. Soc.* **138**:13006–13012. DOI:https://doi.org/10.1021/jacs.6b07612.
302. Perez-Gallent, E., Marcandalli, G., Figueiredo, M.C. et al. (2017). Structure- and potential-dependent cation effects on CO reduction at copper single-crystal electrodes. *J. Am. Chem. Soc.* **139**:16412–16419. DOI:https://doi.org/10.1021/jacs.7b10142.
303. Resasco, J., Chen, L.D., Clark, E. et al. (2017). Promoter effects of alkali metal cations on the electrochemical reduction of carbon dioxide. *J. Am. Chem. Soc.* **139**:11277–11287. DOI:https://doi.org/10.1021/jacs.7b06765.
304. Deng, B., Huang, M., Zhao, X. et al. (2022). Interfacial electrolyte effects on electrocatalytic CO_2 reduction. *ACS Catal.* **12**:331–362. DOI:https://doi.org/10.1021/acscatal.1c03501.
305. Zhang, F. and Co, A.C. (2020). Direct evidence of local pH change and the role of alkali cation during CO_2 electroreduction in aqueous media. *Angew. Chem., Int. Ed.* **59**:1674–1681. DOI:https://doi.org/10.1002/anie.201912637.
306. Chen, L.D., Urushihara, M., Chan, K. et al. (2016). Electric field effects in electrochemical CO_2 reduction. *ACS Catal.* **6**:7133–7139. DOI:https://doi.org/10.1021/acscatal.6b02299.
307. Monteiro, M.C.O., Dattila, F., Hagedoorn, B. et al. (2021). Absence of CO_2 electroreduction on copper, gold and silver electrodes without metal cations in solution. *Nat. Catal.* **4**:654–662. DOI:https://doi.org/10.1038/s41929-021-00655-5.
308. Bohra, D., Chaudhry, J.H., Burdyny, T. et al. (2019). Modeling the electrical double layer to understand the reaction environment in a CO_2 electrocatalytic system. *Energy Environ. Sci.* **12**:3380–3389. DOI:https://doi.org/10.1039/C9EE02485A.
309. Ma, Z., Yang, Z., Lai, W. et al. (2022). CO_2 electroreduction to multicarbon products in strongly acidic electrolyte via synergistically modulating the local microenvironment. *Nat. Commun.* **13**:7596. DOI:https://doi.org/10.1038/s41467-022-35415-x.
310. Yang, X., Ding, H., Li, S. et al. (2024). Cation-induced interfacial hydrophobic microenvironment promotes the C−C coupling in electrochemical CO_2 reduction. *J. Am. Chem. Soc.* **146**:5532–5542. DOI:https://doi.org/10.1021/jacs.3c13602.
311. Zhu, Q., Wallentine, S.K., Deng, G.H. et al. (2022). The slvation-induced onsager reaction field rather than the double-layer field controls CO_2 reduction on gold. *JACS Au* **2**:472–482. DOI:https://doi.org/10.1021/jacsau.1c00512.
312. Monteiro, M.C.O., Dattila, F., López, N. et al. (2022). The role of cation acidity on the competition between hydrogen evolution and CO_2 reduction on gold electrodes. *J. Am. Chem. Soc.* **144**:1589–1602. DOI:https://doi.org/10.1021/jacs.1c10171.
313. Banerjee, S., Han, X. and Thoi, V.S. (2019). Modulating the electrode–electrolyte interface with cationic surfactants in carbon dioxide reduction. *ACS Catal.* **9**:5631–5637. DOI:https://doi.org/10.1021/acscatal.9b00449.
314. Singh, M.R., Kwon, Y., Lum, Y. et al. (2016). Hydrolysis of electrolyte cations enhances the electrochemical reduction of CO_2 over Ag and Cu. *J. Am. Chem. Soc.* **138**:13006–13012. DOI:https://doi.org/10.1021/jacs.6b07612.
315. Resasco, J., Chen, L.D., Clark, E. et al. (2017). Promoter effects of alkali metal cations on the electrochemical reduction of carbon dioxide. *J. Am. Chem. Soc.* **139**:11277–11287. DOI:https://doi.org/10.1021/jacs.7b06765.
316. Sartin, M.M., Yu, Z., Chen, W. et al. (2018). Effect of particle shape and electrolyte cation on CO adsorption to copper oxide nanoparticle electrocatalysts. *J. Phys. Chem. C* **122**:26489–26498. DOI:https://doi.org/10.1021/acs.jpcc.8b08541.
317. Li, J., Li, X., Gunathunge, C.M. et al. (2019). Hydrogen bonding steers the product selectivity of electrocatalytic CO reduction. *Proc. Natl. Acad. Sci. USA* **116**:9220–9229. DOI:https://doi.org/10.1073/pnas.1900761116.
318. Deacon-Price, C., Changeur, L., Santana, C.S. et al. (2024). The effect of the tetraalkylammonium cation in the electrochemical CO_2 reduction reaction on copper electrode. *ACS Catal.* **14**:12928–12939. DOI:https://doi.org/10.1021/acscatal.4c02297.
319. Monteiro, M.C.O., Dattila, F., López, N. et al. (2022). The role of cation acidity on the competition between hydrogen evolution and CO_2 reduction on gold electrodes. *J. Am. Chem. Soc.* **144**:1589–1602. DOI:https://doi.org/10.1021/jacs.1c10171.
320. Liu, S., Li, Y., Wang, D. et al. (2024). Alkali cation-induced cathodic corrosion in Cu electrocatalysts. *Nat. Commun.* **15**:5080. DOI:https://doi.org/10.1038/s41467-024-49492-7.
321. Dunwell, M., Lu, Q., Heyes, J.M. et al. (2017). The central role of bicarbonate in the electrochemical reduction of carbon dioxide on gold. *J. Am. Chem. Soc.* **139**:3774–3783. DOI:https://doi.org/10.1021/jacs.6b13287.
322. Fu, H.Q., Zhang, L., Zheng, L.R. et al. (2019). Enhanced CO_2 electroreduction performance over Cl^- modified metal catalysts. *J. Mater. Chem. A* **7**:12420–12425. DOI:https://doi.org/10.1039/C9TA02223F.
323. Piao, G., Yoon, S.H., Han, D.S. et al. (2020). Ion-enhanced conversion of CO_2 into formate on porous dendritic bismuth electrodes with high efficiency and durability. *ChemSusChem* **13**:698–706. DOI:https://doi.org/10.1002/cssc.201902581.
324. Hori, Y., Koga, O., Watanabe, Y. et al. (1998). FTIR measurements of charge displacement adsorption of CO on poly- and single crystal (100) of Cu electrodes. *Electrochim. Acta* **44**:1389–1395. DOI:https://doi.org/10.1016/S0013-4686(98)00261-8.
325. Sebastián-Pascual, P., Petersen, A.S., Bagger, A. et al. (2021). pH and anion effects on Cu–phosphate interfaces for CO electroreduction. *ACS Catal.* **11**:1128–1135. DOI:https://doi.org/10.1021/acscatal.0c03998.
326. Jouny, M., Luc, W. and Jiao, F. (2018). High-rate electroreduction of carbon monoxide to multi-carbon products. *Nat. Catal.* **1**:748–755. DOI:https://doi.org/10.1038/s41929-018-0133-2.
327. Huang, J.E., Li, F., Ozden, A. et al. (2021). CO_2 electrolysis to multicarbon products in strong acid. *Science* **372**:1074–1078. DOI:https://doi.org/10.1126/science.abg6582.
328. Bondue, C.J., Graf, M., Goyal, A. et al. (2021). Suppression of hydrogen evolution in acidic electrolytes by electrochemical CO_2 reduction. *J. Am. Chem. Soc.* **143**:279–285. DOI:https://doi.org/10.1021/jacs.0c10397.
329. Mohandas, N., Narayanan, T.N. and Cuesta, A. (2023). Tailoring the interfacial water structure by electrolyte engineering for selective electrocatalytic reduction of carbon dioxide. *ACS Catal.* **13**:8384–8393. DOI:https://doi.org/10.1021/acscatal.3c01223.
330. Wang, Y., Wang, D., Dares, C.J. et al. (2018). CO_2 reduction to acetate in mixtures of ultrasmall Cu_nAg_m bimetallic nanoparticles. *Proc. Natl. Acad. Sci. USA* **115**:278–283. DOI:https://doi.org/10.1073/pnas.1713962115.
331. Leal-Duaso, A., Adjez, Y. and Sánchez-Sánchez, C.M. (2024). Role of ionic solvents in the electrocatalytic CO_2 conversion and H_2 evolution suppression: from ionic liquids to deep eutectic solvents. *Chemelectrochem* **11**:e202300771. DOI:https://doi.org/10.1002/celc.202300771.
332. Banerjee, S., Gerke, C.S. and Thoi, V.S. (2022). Guiding CO_2RR selectivity by compositional tuning in the electrochemical double layer. *Acc. Chem. Res.* **55**:504–515. DOI:https://doi.org/10.1021/acs.accounts.1c00680.
333. Xia, C., Zhu, P., Jiang, Q. et al. (2019). Continuous production of pure liquid fuel solutions via electrocatalytic CO_2 reduction using solid-electrolyte devices. *Nat. Energy* **4**:776–785. DOI:https://doi.org/10.1038/s41560-019-0451-x.
334. Zhu, P. and Wang, H. (2021). High-purity and high-concentration liquid fuels through CO_2 electroreduction. *Nat. Catal.* **4**:943–951. DOI:https://doi.org/10.1038/s41929-021-00694-y.
335. Li, Z., Wang, L., Sun, L. et al. (2024). Dynamic cation enrichment during pulsed CO_2 electrolysis and the cation-promoted multicarbon formation. *J. Am. Chem. Soc.* **146**:23901–23908. DOI:https://doi.org/10.1021/jacs.4c06404.
336. Kok, J., de Ruiter, J., van der Stam, W. et al. (2024). Interrogation of oxidative pulsed methods for the stabilization of copper electrodes for CO_2 electrolysis. *J. Am. Chem. Soc.* **146**:19509–19520. DOI:https://doi.org/10.1021/jacs.4c06284.
337. Tan, X. and Nielsen, J. (2022). The integration of bio-catalysis and electrocatalysis to produce fuels and chemicals from carbon dioxide. *Chem. Soc. Rev.* **51**:4763–4785. DOI:https://doi.org/10.1039/D2CS00309K.
338. Batlle-Vilanova, P., Ganigué, R., Ramió-Pujol, S. et al. (2017). Microbial electrosynthesis of butyrate from carbon dioxide: production and extraction. *Bioelectrochemistry* **117**:57–64. DOI:https://doi.org/10.1016/j.bioelechem.2017.06.004.
339. Pepè Sciarria, T., Batlle-Vilanova, P., Colombo, B. et al. (2018). Bio-electrorecycling of carbon dioxide into bioplastics. *Green Chem.* **20**:4058–4066. DOI:https://doi.org/10.1039/C8GC01771A.
340. Bajracharya, S., Srikanth, S., Mohanakrishna, G. et al. (2017). Biotransformation of carbon dioxide in bioelectrochemical systems: state of the art and future prospects. *J. Power Sources* **356**:256–273. DOI:https://doi.org/10.1016/j.jpowsour.2017.04.024.
341. Katuri, K.P., Kalathil, S., Ragab, A. et al. (2018). Dual-function electrocatalytic and macroporous hollow-fiber cathode for converting waste streams to valuable resources using microbial electrochemical systems. *Adv. Mater.* **30**:1707072. DOI:https://doi.org/10.1002/adma.201707072.
342. Santoro, C., Arbizzani, C., Erable, B. et al. (2017). Microbial fuel cells: from fundamentals to applications. a review. *J. Power Sources* **356**:225–244. DOI:https://doi.org/10.1016/j.jpowsour.2017.03.109.
343. Claassens, N.J., Cotton, C.A.R., Kopljar, D. et al. (2019). Making quantitative sense of electromicrobial production. *Nat. Catal.* **2**:437–447. DOI:https://doi.org/10.1038/s41929-019-0272-0.
344. Xu, L., Ma, X., Wu, L. et al. (2022). In situ periodic regeneration of catalyst during CO_2 electroreduction to C_{2+} products. *Angew. Chem., Int. Ed.* **61**:e202210375. DOI:https://doi.org/10.1002/anie.202210375.
345. Ye, K., Zhang, G., Ni, B. et al. (2023). Steering CO_2 electrolysis selectivity by modulating the local reaction environment: an online DEMS approach for Cu electrodes. *eScience* **3**:100143. DOI:https://doi.org/10.1016/j.esci.2023.100143.
346. Casebolt, R., Kimura, K.W., Levine, K. et al. (2021). Effect of electrolyte composition and concentration on pulsed potential electrochemical CO_2 reduction. *Chemelectrochem* **8**:681–688. DOI:https://doi.org/10.1002/celc.202001445.
347. junge Puring, K., Evers, O., Prokein, M. et al. (2020). Assessing the influence of supercritical carbon dioxide on the electrochemical reduction to formic acid using carbon-supported copper catalysts. *ACS Catal.* **10**:12783–12789. DOI:https://doi.org/10.1021/acscatal.0c02983.
348. Zhang, X., Zhang, Z., Li, H. et al. (2022). Insight into heterogeneous electrocatalyst design understanding for the reduction of carbon dioxide. *Adv. Energy Mater.* **12**:2201461. DOI:https://doi.org/10.1002/aenm.202201461.

349. Feng, G., Chen, W., Wang, B. et al. (2018). Oxygenates from the Electrochemical Reduction of Carbon Dioxide. *Chem. Asian J.* **13**:1992–2008. DOI:https://doi.org/10.1002/asia.201800637.
350. Xu, D., Li, K., Jia, B. et al. (2023). Electrocatalytic CO_2 reduction towards industrial applications. *Carbon Energy* **5**:e230. DOI:https://doi.org/10.1002/cey2.230.
351. Okoye-Chine, C.G., Otun, K., Shiba, N. et al. (2022). Conversion of carbon dioxide into fuels—a review. *J. CO_2 Util.* **62**:102099. DOI:https://doi.org/10.1016/j.jcou.2022.102099.
352. Tao, H., Fan, Q., Ma, T. et al. (2020). Two-dimensional materials for energy conversion and storage. *Prog. Mater. Sci.* **111**:100637. DOI:https://doi.org/10.1016/j.pmatsci.2020.100637.
353. Lukatskaya, M.R., Dunn, B. and Gogotsi, Y. (2016). Multidimensional materials and device architectures for future hybrid energy storage. *Nat. Commun.* **7**:12647. DOI:https://doi.org/10.1038/ncomms12647.
354. Wang, N., Miao, R.K., Lee, G. et al. (2021). Suppressing the liquid product crossover in electrochemical CO_2 reduction. *SmartMat* **2**:12–16. DOI:https://doi.org/10.1002/smm2.1018.
355. Xu, L., Iqbal, R., Wang, Y. et al. (2024). Emerging two-dimensional materials: Synthesis, physical properties, and application for catalysis in energy conversion and storage. *Innov. Mater.* **2**:100060. DOI:https://doi.org/10.59717/j.xinn-mater.2024.100060.
356. Li, X., Wang, S., Li, L. et al. (2020). Progress and perspective for *in situ* studies of CO_2 reduction. *J. Am. Chem. Soc.* **142**:9567–9581. DOI:https://doi.org/10.1021/jacs.0c02973.

ACKNOWLEDGMENTS

This work was supported by the Joint Funds of the National Natural Science Foundation of China (U24B20201), National Natural Science Foundation of China (22372007 and 21972010), and the Fundamental Research Funds for the Central Universities (JD2427). Y.J. acknowledges the SRC Center for Electron Transfer (2021R1A5A1030054) funded by NRF Korea and AI Graduate School Program (RS-2021-II211343). The funders had no role in study design, data collection and analysis, decision to publish, or preparation of the manuscript.

AUTHOR CONTRIBUTIONS

Z.S. proposed the topic of the review. X.L. and W.K. wrote the draft. X.F. and Y.C. edited the figures and tables. X.T., J.M., A.W.R., J.T., Y.J., B.D., and Z.S. revised the manuscript. B.H. contributed to discussions about the review. All authors contributed to the manuscript and approved the final version.

DECLARATION OF INTERESTS

B.H. is an Editorial Board member of *The Innovation* and was blinded from reviewing or making final decisions on the manuscript. Peer review was handled independently of this member and their research group.

SUPPLEMENTAL INFORMATION

It can be found online at https://doi.org/10.1016/j.xinn.2025.100807.

The Innovation, Volume *6*

Supplemental Information

Electrochemical CO_2 reduction to liquid fuels: Mechanistic pathways and surface/interface engineering of catalysts and electrolytes

Xueying Li, Woojong Kang, Xinyi Fan, Xinyi Tan, Justus Masa, Alex W. Robertson, Yousung Jung, Buxing Han, John Texter, Yuanfu Cheng, Bin Dai, and Zhenyu Sun

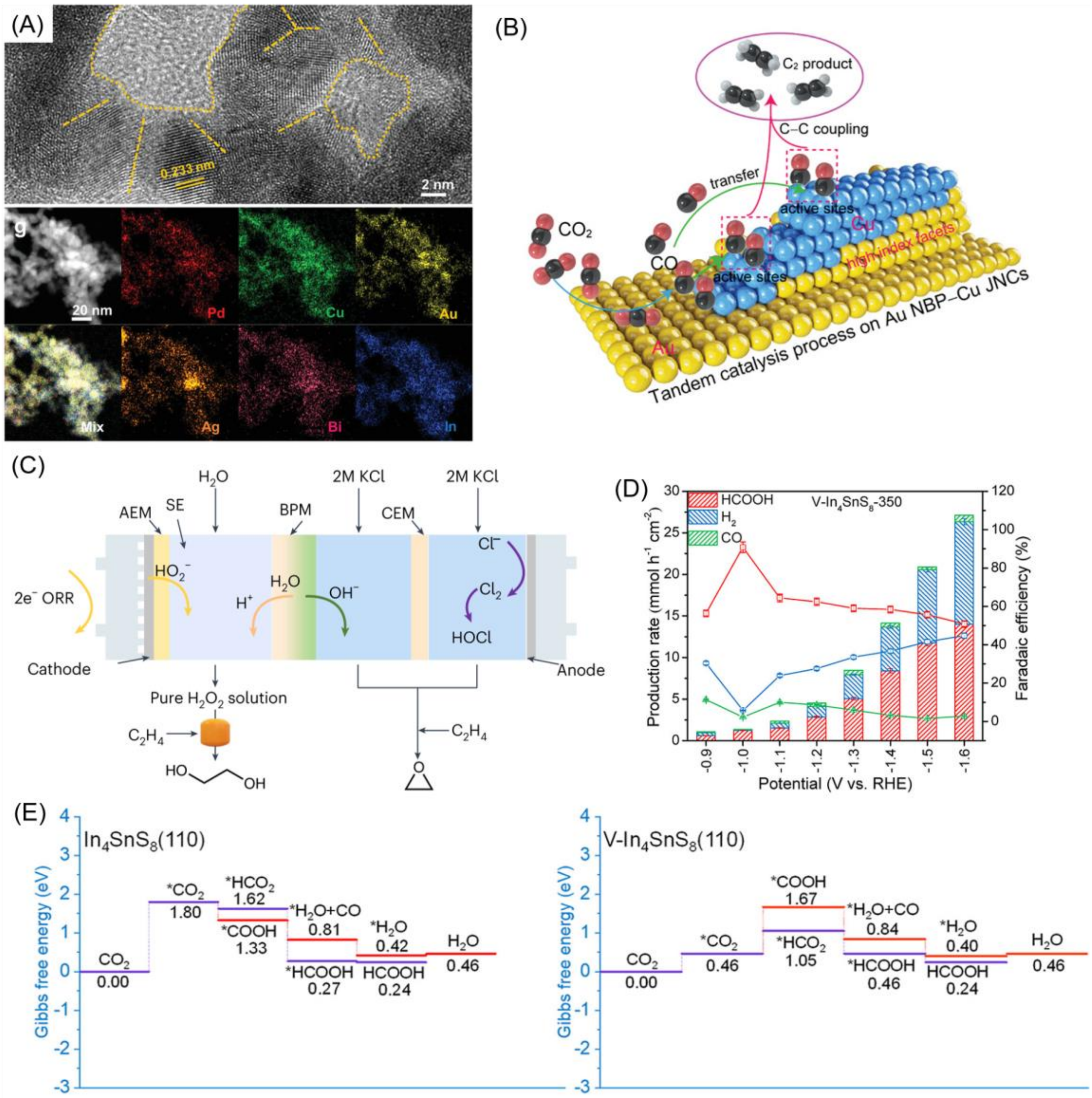

Figure S1. (A) HRTEM and STEM-EDS elemental mappings of PdCuAuAgBiIn HEAs.[1] Copyright 2023, Springer Nature. (B) Proposed mechanism for C_2 production at adjacent Au and Cu sites.[2] Copyright 2021, Wiley-VCH GmbH. (C) Schematic illustration of the three-compartment reactor for ethylene glycol production at the cathodic side and ethylene oxide production at the anodic side. BPM refers to bipolar membrane.[3] Copyright 2023, Springer Nature. (D) Electrochemical performance measurements of V-In_4SnS_8.[4] Copyright 2023, American Chemical Society. (E) Gibbs free energy diagrams of CO_2 reduction to HCOOH on pristine In_4SnS_8(110) and corresponding sulfur defective V-In_4SnS_8(110).[4] Copyright 2023, American Chemical Society.

Table S1. Various liquid products of ECR with their respective equilibrium potentials (E^0 *vs.* reversible hydrogen electrode [RHE])[5,6]

Product	Chemical reaction	E^0 /V
Formic acid ($HCOOH$)	$CO_2 + 2H^+ + 2e^- \rightarrow HCOOH$ (aq)	−0.12
Methanol (CH_3OH)	CO_2 (g) + $6H^+ + 6e^- \rightarrow CH_3OH$ (aq) + H_2O	0.03
Ethanol (C_2H_5OH)	$2CO_2$ (g) + $12H^+ + 12e^- \rightarrow C_2H_5OH$ (aq) + $3H_2O$	0.09
Acetic acid (CH_3COOH)	$2CO_2$ (g) + $8H^+ + 8e^- \rightarrow CH_3COOH$ (aq) + $2H_2O$	0.11
Acetaldehyde (CH_3CHO)	$2CO_2 + 10H^+ + 10e^- \rightarrow CH_3CHO$ (aq) + $3H_2O$	0.06
Ethylene glycol (HOC_2H_4OH)	$2CO_2 + 10H^+ + 10e^- \rightarrow HOC_2H_4OH$ (aq) + $2H_2O$	0.20
n-Propanol (*n*-C_3H_7OH)	$3CO_2 + 18H^+ + 18e^- \rightarrow$ *n*-C_3H_7OH (aq) + $5H_2O$	0.10
Propionaldehyde (C_2H_5CHO)	$3CO_2 + 16H^+ + 16e^- \rightarrow C_2H_5CHO$ (aq) + $5H_2O$	0.09
Methylglyoxal ($C_3H_4O_2$)	$3CO_2 + 12H^+ + 12e^- \rightarrow C_3H_4O_2$ (aq) + $4H_2O$	0.02
2,3-Furandiol ($C_4H_4O_3$)	$4CO_2 + 14H^+ + 14e^- \rightarrow C_4H_4O_3$ (aq) + $5H_2O$	0.01

Table S2. Summary of recent reported ECR catalysts for formic acid/formate production.

Catalyst	Electrolyte	Electrolytic cell	Applied potential (V *vs.* RHE)	Partial current density (mA cm^{-2})	Faradaic efficiency (%)	Stability (h)	Ref.
$In_{1.5}Cu_{0.5}$ NPs	0.1 M $KHCO_3$	H-type	−1.2	−4.0	90	5	7
BMNS	0.5 M $KHCO_3$	H-type	−0.8	−23.0	98	40	8
NW-SnO_2	0.5 M $KHCO_3$	H-type	−1.0	−22.0	87.4	18	9
Atomically isolated In (In−N−C)	0.5 M $KHCO_3$	H-type	−0.79	−6.8	80	/	10
Cu−MOF derived Cu	$BmimBF_4$, (0.5 M)/(MeCN)/H_2O (1.0 M)	H-type	−1.85 (V *vs.* Ag/Ag^+)	−102.1	98.2	10	11
Bi-Sn Aerogel	0.1 M $KHCO_3$	H-type	−1.0	−9.3	93.9	10	12
Bi_2S_3-derived catalyst	1.0 M KOH	Flow cell with GDE	−0.95	−2000	93	100	13
HCS/Cu	0.5 M $KHCO_3$	H-type	−0.81	−26	82.4	24 h	14
CuSn alloy @ Cu doped SnO	0.5 M $KHCO_3$	H-type	−1.2	/	95.4	10	15
Monolayer Bi_2WO_6	0.5 M $KHCO_3$	H-type	−1.0	−47.18	98.64	12	16
	1.0 M KOH	Flow cell with GDE	−1.3	−300	> 97 at −250 mA cm^{-2}	5	
Bi_2O_3 spheres	0.5 M $KHCO_3$	H-type	−0.9	−8	91	24	17
Bi-decorated SnO_x	0.5 M $KHCO_3$	H-type	−1.37	−45	90.8	10	18
$Bi_2O_2CO_3$ nanosheets	0.5 M $KHCO_3$	H-type	−1.8 (V *vs.* Ag/AgCl)	−35	92.6	8	19
Cu_3PdN nanocrystal	0.1 M $KHCO_3$	H-type	−1.0	/	43	17	20
Ch-Sn_3Se_7 derived SnO_2	0.1 M $KHCO_3$	H-type	−1.06	−11.5	94.5	100	21
Bimetallic Zn_3Sn_2	0.5 M $KHCO_3$	H-type	−1.1	−26	96.7	10	22
BiNS	0.05 M H_2SO_4 + 3 M KCl	Flow cell with GDE	−1.28	−257.1	92.2	/	23
MIL-	0.1 M	Flow cell	−1.1	−108	94.4	24	24

68(In)-NH_2	$KHCO_3$	with GDE					
Zn(Pb)-4	0.1 M $KHCO_3$	H-type	−1.2	−47	95	/	25
Bi_2O_3/BiO_2	0.5 M $KHCO_3$	Flow cell with GDE	−1.3	−111.42	98.12	/	26
	/	MEA-installed cell	−3.7 (full cell)	−220	95.4	30	
S-In_2O_3 derived In	0.5 M $KHCO_3$	H-type	−0.98	−57	93	10	27
	0.5 M $CsHCO_3$	H-type	−0.98	−84	93	/	
Cu–CTAB	0.5 M $KHCO_3$	H-type	−0.5	−2.48	82.3	10	28
Bi nanobelts	0.5 M $KHCO_3$	H-type	−0.88	−46.0 (at −1.18 V *vs.* RHE)	93.3	/	29
	1 M KOH	Flow cell with GDE	−1.47	−400	95	23	
BiOx@C	1 M $KHCO_3$	H-type	−1.7	−37.8	89.3	/	30
Oxygen vacancy-rich In_2O_3	0.1 M $KHCO_3$	H-type	−1.27	−22.1	91.2	80	31
N-Sn(S) nanosheets	0.1 M $KHCO_3$	H-type	−0.7	/	93.3	20	32
PdBi nanosheets	0.5 M $KHCO_3$	H-type	−1.0	−30.75	91.9	10	33
Ag/$Bi_2O_2CO_3$	0.5 M $KHCO_3$	H-type	−0.86	−96.6 (at −1.36 V *vs.* RHE)	98	9.7	34
BiO_{2-x} Nanosheets	0.1 M $KHCO_3$	H-type	−1.1	−12	99.1	15	35
	1 M KOH	Flow cell with GDE	−1.0	−319	91.3	/	
Bi nanotubes	0.5 M $KHCO_3$	H-type	−0.9	−31.1	97.1	65	36
Pd_3Bi intermetallic alloys	0.1 M $KHCO_3$	H-type	−0.17	−3	99	8.3	37
S-doped Bi nanosheets	0.5 M $KHCO_3$	H-type	−0.9	−25.47	96.7	35	38
	1 M KOH	Flow cell with GDE	−0.9	−182.1	97.8	/	
SnO_2/NC	0.5 M $KHCO_3$	H-type	−1.13	−18.2	87.6	20	39
$Bi_{19}Br_3S_{27}$ nanowire	1 M KOH	Flow cell with GDE	−0.95	−250	95	2.7	40
In@InO_x nanoparticle	0.5 M $KHCO_3$	H-type	−1.0	−32.6	98	25	41
InS nanorods	1 M KOH	Flow cell with GDE	−0.7	/	94.2	58	42

$Au_{0.50}NP$-in-PdNS	0.1 M $KHCO_3$	H-type	−0.6	−13.7	97.8	2.7	43
Vo-BOC-NS	0.1 M $KHCO_3$	H-type	−0.62	−286	95	12	44
Nanoporous SnTe	0.1 M $KHCO_3$	H-type	−1.0	/	93	60	45
lattice-distorted $Bi_2O_2CO_3$	1 M KOH	Flow cell with GDE	/	−800	91 at −200 mA cm^{-2}	26	46
$(BiO)_2CO_3$ Nanoflower	0.1 M $KHCO_3$	H-type	−1.0	−22.2	98.9	36	47
	1 M KOH	Flow cell with GDE	−1.2	−316	97.3	5	
$AgIn_5S_8$	0.5 M $KHCO_3$	H-type	−1.0	−30.8	91.7	12	48
	1 M KOH	Flow cell with GDE	−0.95	−560	94	/	
$Pb_3(CO_3)_2(OH)_2$ (4 wt%)	0.1 M $KHCO_3$	H-type	−1.2	−13	96.5	10	49
Sb_2Bi_6	0.5 M $KHCO_3$	H-type	−0.9	−33.7	95.8	12	50
	1 M KOH	Flow cell with GDE	−0.9	−734	95.8		
Cu-doped Bismuth	1 M KOH	Flow cell with GDE	−0.86	−660	96.1	5	51
	1 M KOH	MEA-installed cell	/	/	~80	100 at −400 mA cm^{-2}	
FTO/C	1 M KOH	Flow cell with GDE	/	/	95 at −100 mA cm^{-2}	160	52
In/N-dG	1 M KOH	Flow cell with GDE	−1.17	−1152	96	14	53
Pb-PhyA	[Bzmim]BF_4–MeCN–H_2O	H-type	−2.25	−28.3	92.7	5	54
Bi@Bi_2O_3-NDs	0.1 M $KHCO_3$	H-type	−0.9	−11.4	92.3	4	55
PVB-C	0.1 M $KHCO_3$	H-type	−1.03	−31.0	93.6	108	56
S-CuSn	0.5 M $KHCO_3$	H-type	−1.2	−54.9	91.5	30	57

Table S3. Summary of recent reported ECR catalysts for methanol production.

Catalyst	Electrolyte	Electrolytic cell	Applied potential (V *vs.* RHE)	Partial current density (mA cm^{-2})	Faradaic efficiency (%)	Stability	Ref.
CoO/CN/Ni	0.5 M $KHCO_3$	H-type	−0.7	−10.6	70.7	10	58
SA-Cu-MXene	0.1 M $KHCO_3$	H-type	−1.4	−21.3	59.1	30	59
Mo-Bi BMC	MeCN / [Bmim]BF_4 (0.5 M)	H-type	−0.7 (V *vs.* SHE)	−12.1	71.2	5	60
2D hierarchical Pd/SnO_2	0.1 M $NaHCO_3$	H-type	−0.24	−0.8	54.8	8	61
BP/CP	0.1 M $KHCO_3$	H-type	−0.5	/	92	18	62
$Cu_2O_{(OL\text{-}MH)}$/Ppy	0.5 M $KHCO_3$	H-type	−0.85	−0.21	93	1	63
$Co(CO_3)_{0.5}(OH)\cdot 0.11H_2O$	0.5 M $NaHCO_3$	H-type	−0.98 (V *vs.* SCE)	−0.59	97	10	64
$Cu_{1.63}Se(1/3)$	[Bmim]PF_6 /H_2O	H-type	−2.1 (V *vs.* Ag/Ag^+)	−41.5	77.6	25	65
Ag,S-Cu_2O/Cu	BMImBF_4/ H_2O	H-type	−1.18	−82.7	67.4	24	66
Pt_xZn/C	0.1 M $NaHCO_3$	H-type	−0.9	/	81.4	16 h	67
CoPc/CNT	0.1 M $KHCO_3$	H-type	−0.82	−10.6	44	1 h	68
CoPc–NH_2/CNT	0.1 M $KHCO_3$	H-type	−1.00	−10.2	32	12 h	
MOF-derived Cu@Cu_2O	0.5 M $KHCO_3$	H-type	−0.7	/	45	10 h	69
Ni-2D-O-SA-CNT	0.1 M $KHCO_3$	H-type	−0.9	−0.94 (at −0.95 V *vs.* RHE)	27	5.5 h	70
CuSAs/TCNFs	0.1 M $KHCO_3$	H-type	−0.9	−93	44	50 h	71
$Pd_{83}Cu_{17}$ aerogel	[Bmim]BF_4 /H_2O	H-type	−2.1 (V *vs.* Ag/ Ag^+)	−31.8	80	24 h	72
Sn/Vo-CuO	[Bmim]BF_4 /H_2O	H-type	−2.0 (V *vs.* Ag/ Ag^+)	−67	88.6	36 h	73
CuO NWs	1 M KOH	Flow cell with GDE	/	−29.7	66.1	/	74

Mo–Bi BMC@CNT	[Emim]BF_4/H_2O	H-type	−0.3 (V *vs.* SCE)	−5.6	81	12 h	75
Boron-doped diamond	1 M NH_3	H-type	−1.3 (V *vs.* Ag/AgCl)	/	24.3	/	76
cobalt-phthalocyanine-based CONs (iminium-CONs)	0.2 M KOH and 1.5 M KCl	Flow cell with GDE	−0.78	−91.7	54	6 h	77
CoPc/SWCNTs	0.5 M $KHCO_3$	H-type	−0.93	−8.8	53.4	10 h	78
		Flow cell with GDE	−0.9	−66.8	31.3	/	

Table S4. Summary of recent reported ECR catalysts for ethanol production.

Catalyst	Electrolytes	Electrolytic cell	Applied potential (V *vs.* RHE)	Partial current density (mA cm^{-2})	Faradaic efficiency (%)	Stability (h)	Ref.
D-Cu_2O/Cu	0.1 M KCl	H-type	−1.2	−25.1	20.5	10	79
ZIS NSAs/NDCC	0.5 M $KHCO_3$	H-type	−0.7	−1.3	42	4	80
nitrogen-doped graphene/Cu foam	0.1 M $KHCO_3$	H-type	−0.8	−22	33.1	1	81
Cu/Cu_2O–CV	0.1 M $KHCO_3$	H-type	−1.06	−4.2	56.6	120	80
Cu-PTFE-99 NN	0.1 M $KHCO_3$	H-type	−1.5	/	42.3	/	66
	1 M KOH	Flow cell with GDE	/	/	40.1 at −300 mA cm^{-2}	27	
Hex-2Cu-O	0.1 M $KHCO_3$	H-type	−1.2	−8.9	32.5	25	82
dCu_2O/Ag2.3%	1 M KOH	Flow cell with GDE	/	/	40.8 at −800 mA cm^{-2}	/	83
$Cu_{1.22}V_{0.19}Se$ nanotube	1 M KOH	Flow cell with GDE	−0.8	−304	68.3	14	84
	0.1 M $KHCO_3$	H-type	−0.8	−21.3	70.5	138	
Cu_3Sn	0.1 M $KHCO_3$	H-type	−1.0	−8.8	64	48	85
K–F–Cu–CO_2	1 M KOH	Flow cell with GDE	/	/	52.9 at −800 mA cm^{-2}	12	86
B/N-doped sp^3/sp^2 hybridized nanocarbon (BNHC)	0.1 M $KHCO_3$	H-type	−0.6	−0.4	56	16	87
Cu_2S_{1-x} HN	0.5 M $KHCO_3$	Flow cell with GDE	−0.3	/	73.3	6	88
Cu-KOH/Ethanol-CV	0.1 M $KHCO_3$	H-type	−1.4	−35.1	42.1	12	89
MoP-Im	3 M KOH and 3 M KCl	Flow cell with GDE	−0.5	−90.0	77.4	12	90

np-Cu@VO_2-5%	0.1 M $KHCO_3$	H-type	−0.62	−16.0	30.1	/	[91]
	1.0 M KOH	Flow cell with GDE	−0.8	−38.0	38.2		
Cu/Cu_2O aerogel	0.1 M KCl	H-type	−1.1	−32.5	41.2	/	[92]
	1 M KCl	Flow cell with GDE	−0.7	−72.1	45.0		
Cu/C-0.4	0.1 M $KHCO_3$	RDE	−0.7	−1.12	91	16	[93]
defect-site-rich Cu structure Cu-$_{DS}$	0.1 M $KHCO_3$	H-type	−1.08	/	53	/	[94]
	1 M KOH	Flow cell with GDE	−0.95	−100	52	/	
	/	MEA-installed cell	−3.5 (Full cell)	/	60 at −200 mA cm^{-2}	30	
$CuAl_2O_4$/CuO	1 M KOH	Flow cell with GDE	−1.0	−82	41	150	[95]
CuNi@C/N-npG	0.5 M $KHCO_3$	H-type	−0.78	/	84	36	[96]
Cu/CuNC	0.1 M $KHCO_3$	H-type	−0.35	−0.54	55	1	[97]
GO-VB_6-Cu	0.1 M $KHCO_3$	H-type	−0.25	−2.57	56.3	24	[98]
CuNi@C/N-npG	0.5 M $KHCO_3$	H-type	−0.78	/	84	36	[96]
$CuAl_2O_4$/CuO	1 M KOH	Flow cell with GDE	/	/	41 at −200 mA cm^{-2}	100	[99]
Ag-CuNPs 5 %	3 M KOH	Flow cell with GDE	−0.78	−173.7	52.6	60	[100]
a-CuTi@Cu	0.1 M $KHCO_3$	H-type	−0.8	/	23.96	6	[101]
FeTPP[Cl]/Cu	1 M $KHCO_3$	Flow cell with GDE	−0.82	−124	41	12	[102]
Cu GNC	0.5 M $KHCO_3$	H-type	−0.87	−10.4	70.52	/	[103]
Cu_xAu_y NWA	0.1 M $KHCO_3$	H-type	−0.7	−0.4	48	8	[104]
Cu sandwich	0.1 M $KHCO_3$	H-type	−0.3	−0.3	31	18	[105]
BND	0.1 M $NaHCO_3$	H-type	−1.0	/	93.2	16	[106]
Cu-Cu_2O-3	0.1 M KCl	H-type	−0.4	−2.94	39.2	/	[107]

Table S5. Summary of recent reported ECR catalysts for *n*-propanol production.

Catalysts	Electrolyte	Electrolytic cell	Applied potential (V *vs.* RHE)	Partial current density (mA cm^{-2})	Faradaic efficiency （%）	Stability (h)	Ref.
R-Cu-$_{\mathrm{C}}$	0.1M $KHCO_3$	H-type	−1.05	−8.1	17.3	/	108
Hex-2Cu-O	0.1M $KHCO_3$	H-type	−1.10	−1.9	17.2	25	82
CuSx-DSV	1M KOH	Flow cell	−0.85	−9.9	3.1	1.25	109
	0.1M $KHCO_3$	H-type	−1.05	−3.1	15.4	10	
Cu_2S-Cu-V	0.1M $KHCO_3$	H-type	–0.95	−2.5	8	16	110
Cu-on-Cu_3N	0.1M $KHCO_3$	H-type	–0.95	−1.3	6	30	111
Cu nanoparticles	0.1M $KHCO_3$	H-type	–0.81	−0.8	5.9	/	112
Activated Cu mesh	0.5M $KHCO_3$	H-type	–0.90	−1.3	13.1	6	113
Agglomerated Cu nanocrystals	0.1M $KHCO_3$	H-type	–0.95	−1.7	8.8	6	114
Cu_2O-derived	0.5M $NaHCO_3$	H-type	−0.85	−0.9	5.7	/	115
Oxide-derived Pd_9Cu_{91}	0.5M $KHCO_3$	H-type	−0.65	−1.15	13.7	102	116
Pulsed-Cu(100)	0.1M $KHCO_3$	H-type	−1.00	/	5.5	/	117
Cu-B	0.1M $KHCO_3$	H-type	−1.15	−0.58	7.1	/	118
Cu-P	0.1M $KHCO_3$	H-type	−1.15	−0.41	5.1	/	118
Cu nanoparticles	1M $KHCO_3$	H-type	−0.97	−13.8	4.6	20	119

Table S6. List of ECR catalysts suggested via computational screening for CO_2 reduction to liquid fuels. Catalytic selectivity encompasses either competition with HER or product selectivity in ECR, while stability encompasses at least one of thermodynamic, electrochemical, or thermal stability. The major screening method is noted, with ML/DFT implies screening results of ML-aided screening is validated by DFT calculations. Due to diverse material types, we followed the conventions of individual literature in presenting ECR catalyst candidates. Further structural and operational details of a catalyst are available in the corresponding literature.

ECR catalysts	Product	U_{lim} (V)	Selectivity considered	Stability considered	Method	Ref.
Pd/W	HCOOH	−0.23	yes	yes	DFT	120
Au/Hf	HCOOH	−0.16	yes	yes	DFT	120
Au/Zr	HCOOH	−0.17	yes	yes	DFT	120
Fe_2N_2-Gra	HCOOH	−0.23	yes	yes	DFT	121
Mn-Ti_2CN_2	HCOOH	−0.32	yes	yes	DFT	10
Fe-Ti_2CN_2	HCOOH	−0.43	yes	yes	DFT	10
Mn-Na_4SiMo_{12}	HCOOH	−0.48	yes	yes	DFT	122
Co@2D-FeS_2	HCOOH	−0.22	yes	yes	DFT	123
Pt@Ag	HCOOH	−0.25	yes	yes	DFT	124
CrPd@PC_6	HCOOH	−0.25	Yes	yes	DFT	125
ZnN_4@BC_6N	HCOOH	−0.28	yes	yes	DFT	126
Co_2-N@Gra	HCOOH	−0.34	yes	yes	DFT	127
W/Au	CH_3OH	−0.63	yes	yes	DFT	128
Pt@dv-Gr	CH_3OH	−0.27	yes	yes	DFT	129
Ag/Ta	CH_3OH	−0.67	no	yes	DFT	120
Ru@C_2N	CH_3OH	−0.56	yes	yes	DFT	130
Au_2B	CH_3OH	−0.29	yes	yes	DFT	131
Ir-Mo_2B_2	CH_3OH	−0.27	yes	yes	DFT	132
Sc@Cu	C_{2+}	N/A	yes	yes	DFT	133

Y@Cu	C_{2+}	N/A	yes	yes	DFT	133
(Sc, Ag) @Cu	C_{2+}	N/A	yes	yes	DFT	133
(Y, Ag) @Cu	C_{2+}	N/A	yes	yes	DFT	133
(Y, Fe) @Cu	C_{2+}	N/A	yes	yes	DFT	133
(Y, Ru) @Cu	C_{2+}	N/A	yes	yes	DFT	133
(Y, Cd) @Cu	C_{2+}	N/A	yes	yes	DFT	133
(Y, Au) @Cu	C_{2+}	N/A	yes	yes	DFT	133
(V, Ag) @Cu	C_{2+}	N/A	yes	yes	DFT	133
Cu_9In_4	HCOOH	−0.19	yes	yes	ML/DFT	134
CuNiZn	CH_3OH	N/A	yes	yes	ML	135
CuCoZn	CH_3OH	N/A	yes	yes	ML	135
CuCoNiZn	CH_3OH	N/A	yes	yes	ML	135
CuCoNiZnSn	CH_3OH	N/A	yes	yes	ML	136
CuNiZnSn	CH_3OH	N/A	yes	yes	ML	136
Mn-zeolite	CH_3OH	−0.5	yes	yes	ML	137
Pb-zeolite	CH_3OH	−0.87	yes	yes	ML	137
Ti@Pd	CH_3OH	−0.63	yes	no	ML/DFT	138
RhIr⊥gra	CH_3OH	−0.35	yes	yes	ML/DFT	139
RhPt⊥gra	CH_3OH	−0.25	yes	yes	ML/DFT	139
C/C on Cu	C_{2+}	N/A	yes	no	ML	140
AgCu	C_{2+}	N/A	no	no	ML	141
AuCu	C_{2+}	N/A	no	no	ML	141
AlAu	N/A	N/A	no	no	ML	142
AlNi*	N/A	N/A	no	no	ML	142
AlPd*	N/A	N/A	no	no	ML	142
AuSn	N/A	N/A	no	no	ML	142
CuAl	N/A	N/A	no	no	ML	142

CuAu	N/A	N/A	no	no	ML	142
CuGa	N/A	N/A	no	no	ML	142
CuSi	N/A	N/A	no	no	ML	142
CuSn	N/A	N/A	no	no	ML	142
PdSi*	N/A	N/A	no	no	ML	142
Co_9Ga_{42}Ni7Zn 42	N/A	N/A	yes	no	ML	143
$Ga_{83}Ni_{17}$	N/A	N/A	yes	no	ML	143
$Ag_{69}Cu_{31}$	N/A	N/A	yes	no	ML	143
$Ag_{84}Pd_{16}$	N/A	N/A	yes	no	ML	143
$Au_{84}Pd_{16}$	N/A	N/A	yes	no	ML	143
Co-CS_3	N/A	N/A	yes	no	ML	144
Fe-C_2S_2	N/A	N/A	yes	no	ML	144
Ni-C_2NP	N/A	N/A	yes	no	ML	144
Sc-CN_3	N/A	N/A	yes	no	ML	144
Ti-C_2S_2	N/A	N/A	yes	no	ML	144
V-NP_3	N/A	N/A	yes	no	ML	144
Zr-CN_2S	N/A	N/A	yes	no	ML	144
Sc-C_2O_2	N/A	N/A	yes	yes	ML	144
Sc-CN_2O	N/A	N/A	yes	yes	ML	144
Y-CN_2O	N/A	N/A	yes	yes	ML	144
P@Cu	N/A	N/A	yes	yes	ML	145
Ag@Cu	N/A	N/A	yes	yes	ML	145
Ga@Cu	N/A	N/A	yes	yes	ML	145
Zn@Cu	N/A	N/A	yes	yes	ML	145
Sn@Cu	N/A	N/A	yes	yes	ML	145
Ge@Cu	N/A	N/A	yes	yes	ML	145
In@Cu	N/A	N/A	yes	yes	ML	145

Si@Cu	N/A	N/A	yes	yes	ML	145

Table S7. p*K*a of hydrolysis of cations in the electrolyte.[146]

Cation	Cation size (pm)	pK_a in bulk electrolyte
Li^+	69	13.6
Na^+	102	14.2
K^+	138	14.5
Rb^+	149	14.6
Cs^+	170	14.7

REFERENCES

1. Qin H. G., Du Y. F., Bai Y. Y., et al. (2023). Surface-immobilized cross-linked cationic polyelectrolyte enables CO_2 reduction with metal cation-free acidic electrolyte. *Nat. Commun.* **14**:5640. DOI:10.1038/s41467-023-41396-2
2. Jia H., Yang Y., Chow T. H., et al. (2021). Symmetry-broken Au–Cu heterostructures and their tandem catalysis process in electrochemical CO_2 reduction. *Adv. Funct. Mater.* **31**:2101255. DOI:10.1002/adfm.202101255
3. Fan L., Zhao Y., Chen L., et al. (2023). Selective production of ethylene glycol at high rate via cascade catalysis. *Nat. Catal.* **6**: 585-595. DOI:10.1038/s41929-023-00977-6
4. Niu Z., Gao X., Lou S., et al. (2023). Theory-guided S-defects boost selective conversion of CO_2 to HCOOH over In_4SnS_8 nanoflowers. *ACS Catal.* **13**:2998-3006. DOI:10.1021/acscatal.2c05957
5. Nitopi S., Bertheussen E., Scott S. B., et al. (2019). Progress and perspectives of electrochemical CO_2 reduction on copper in aqueous electrolyte. *Chem. Rev.* **119**:7610-7672. DOI:10.1021/acs.chemrev.8b00705
6. Calvinho K. U. D., Alherz A. W., Yap K. M. K., et al. (2021). Surface hydrides on Fe_2P electrocatalyst reduce CO_2 at low overpotential: steering selectivity to ethylene glycol. *J. Am. Chem. Soc.* **143**:21275-21285. DOI:10.1021/jacs.1c03428
7. Wei B., Xiong Y., Zhang Z., et al. (2021). Efficient electrocatalytic reduction of CO_2 to HCOOH by bimetallic In-Cu nanoparticles with controlled growth facet. *Appl. Catal., B* **283**:119646. DOI:10.1016/j.apcatb.2020.119646
8. Li N., Yan P., Tang Y., et al. (2021). In-situ formation of ligand-stabilized bismuth nanosheets for efficient CO_2 conversion. *Appl. Catal., B* **297**:120481. DOI:10.1016/j.apcatb.2021.120481
9. Chen Z., Fan T., Zhang Y.-Q., et al. (2020). Wavy SnO_2 catalyzed simultaneous reinforcement of carbon dioxide adsorption and activation towards electrochemical conversion of CO_2 to HCOOH. *Appl. Catal., B* **261**:118243.

DOI:10.1016/j.apcatb.2019.118243

10. Lu P., Tan X., Zhao H., et al. (2021). Atomically dispersed indium sites for selective CO_2 electroreduction to formic acid. *ACS Nano* **15**:5671-5678. DOI:10.1021/acsnano.1c00858

11. Zhu Q., Yang D., Liu H., et al. (2020). Hollow metal-organic-framework-mediated in situ architecture of copper dendrites for enhanced CO_2 electroreduction. *Angew. Chem., Int. Ed.* **59**:8896-8901. DOI:10.1002/anie.202001216

12. Wu Z., Wu H., Cai W., et al. (2021). Engineering bismuth–tin interface in bimetallic aerogel with a 3D porous structure for highly selective electrocatalytic CO_2 reduction to HCOOH. *Angew. Chem., Int. Ed.* **60**:12554-12559. DOI:10.1002/anie.202102832

13. Lin L., He X., Zhang X. G., et al. (2023). A nanocomposite of bismuth clusters and $Bi_2O_2CO_3$ sheets for highly efficient electrocatalytic reduction of CO_2 to formate. *Angew. Chem., Int. Ed.* **62**:e202214959. DOI:10.1002/anie.202214959

14. Du J., Xin Y., Dong M., et al. (2021). Copper/carbon heterogenous interfaces for enhanced selective electrocatalytic reduction of CO_2 to formate. *Small* **17**:2102629. DOI:10.1002/smll.202102629

15. Zhang M., Zhang Z., Zhao Z., et al. (2021). Tunable selectivity for electrochemical CO_2 reduction by bimetallic Cu–Sn catalysts: elucidating the roles of Cu and Sn. *ACS Catal.* **11**:11103-11108. DOI:10.1021/acscatal.1c02556

16. Liu S., Wang C., Wu J., et al. (2021). Efficient CO_2 electroreduction with a monolayer Bi_2WO_6 through a metallic intermediate surface state. *ACS Catal.* **11**:12476-12484. DOI:10.1021/acscatal.1c02495

17. Deng P., Wang H., Qi R., et al. (2020). Bismuth oxides with enhanced bismuth–oxygen structure for efficient electrochemical reduction of carbon dioxide to formate. *ACS Catal.* **10**:743-750. DOI:10.1021/acscatal.9b04043

18. Yuan T., Hu Z., Zhao Y., et al. (2020). Two-dimensional amorphous SnO_x from liquid metal: mass production, phase transfer, and electrocatalytic CO_2 reduction toward formic acid. *Nano Lett.* **20**:2916-2922.

DOI:10.1021/acs.nanolett.0c00844

19. Wang Y., Wang B., Jiang W., et al. (2022). Sub-2 nm ultra-thin $Bi_2O_2CO_3$ nanosheets with abundant Bi–O structures toward formic acid electrosynthesis over a wide potential window. *Nano Res.* **15**:2919-2927. DOI:10.1007/s12274-021-3903-0

20. Jia J., Hao X., Chang Y., et al. (2021). Rational design of Cu_3PdN nanocrystals for selective electroreduction of carbon dioxide to formic acid. *J. Colloid Interface Sci.* **586**:491-497. DOI:10.1016/j.jcis.2020.10.112

21. Zhang S., Sun M., Wang K. Y., et al. (2021). Conversion of organically directed selenidostannate into porous SnO_2 exhibiting effective electrochemical reduction of CO_2 to C_1 products. *ACS Sustainable Chem. Eng.* **9**:2358-2366. DOI:10.1021/acssuschemeng.0c08634

22. Liu W., Zhang Z., Huo S., et al. (2023). Bimetallic Zn_3Sn_2 electrocatalyst derived from mixed oxides enhances formate production towards CO_2 electroreduction reaction. *Appl. Surf. Sci.* **608**:155110. DOI:10.1016/j.apsusc.2022.155110

23. Qiao Y., Lai W., Huang K., et al. (2022). Engineering the local microenvironment over Bi nanosheets for highly selective electrocatalytic conversion of CO_2 to HCOOH in strong acid. *ACS Catal.* **12**:2357-2364. DOI:10.1021/acscatal.1c05135

24. Wang Z., Zhou Y., Xia C., et al. (2021). Efficient electroconversion of carbon dioxide to formate by a reconstructed amino-functionalized indium–organic framework electrocatalyst. *Angew. Chem., Int. Ed.* **60**:19107-19112. DOI:10.1002/anie.202107523

25. Mohamed A. G. A., Zhou E., Zeng Z., et al. (2022). Asymmetric oxo-bridged ZnPb bimetallic electrocatalysis boosting CO_2-to-HCOOH reduction. *Adv. Sci.* **9**:2104138. DOI:10.1002/advs.202104138

26. Feng X., Zou H., Zheng R., et al. (2022). Bi_2O_3/BiO_2 nanoheterojunction for highly efficient electrocatalytic CO_2 reduction to formate. *Nano Lett.* **22**:1656-1664. DOI:10.1021/acs.nanolett.1c04683

27. Ma W., Xie S., Zhang X. G., et al. (2019). Promoting electrocatalytic CO_2 reduction to formate via sulfur-boosting water activation on indium surfaces. *Nat. Commun.* **10**:892. DOI:10.1038/s41467-019-08805-x

28. Tao Z., Wu Z., Wu Y., et al. (2020). Activating copper for electrocatalytic CO_2 reduction to formate via molecular interactions. *ACS Catal.* **10**:9271-9275. DOI:10.1021/acscatal.0c02237

29. Zeng G., He Y., Ma D. D., et al. (2022). Reconstruction of ultrahigh-aspect-ratio crystalline bismuth–organic hybrid nanobelts for selective electrocatalytic CO_2 reduction to formate. *Adv. Funct. Mater.* **32**:2201125. DOI:10.1002/adfm.202201125

30. Zhao X. H., Chen Q. S., Zhuo D. H., et al. (2021). Oxygen vacancies enriched Bi based catalysts for enhancing electrocatalytic CO2 reduction to formate. *Electrochim. Acta* **367**:137478. DOI:10.1016/j.electacta.2020.137478

31. Cheng Q., Huang M., Xiao L., et al. (2023). Unraveling the influence of oxygen vacancy concentration on electrocatalytic CO_2 reduction to formate over indium oxide catalysts. *ACS Catal.* **13**:4021-4029. DOI:10.1021/acscatal.2c06228

32. Cheng H., Liu S., Zhang J., et al. (2020). Surface nitrogen-injection engineering for high formation rate of CO_2 reduction to formate. *Nano Lett.* **20**:6097-6103. DOI:10.1021/acs.nanolett.0c02144

33. Xie L., Liu X., Huang F., et al. (2022). Regulating Pd-catalysis for electrocatalytic CO_2 reduction to formate via intermetallic PdBi nanosheets. *Chin. J. Catal.* **43**:1680-1686. DOI:10.1016/S1872-2067(21)63999-2

34. Guo X., Xu S.-M., Zhou H., et al. (2022). Engineering hydrogen generation sites to promote electrocatalytic CO_2 reduction to formate. *ACS Catal.* **12**:10551-10559. DOI:10.1021/acscatal.2c02548

35. Tan Z., Zhang J., Yang Y., et al. (2022). BiO_{2-x} nanosheets with surface electron localizations for efficient electrocatalytic CO_2 reduction to formate. *CCS Chem.* **5**:133-144. DOI:10.31635/ccschem.022.202202068

36. Zheng H., Wu G., Gao G., et al. (2021). The bismuth architecture assembled by nanotubes used as highly efficient electrocatalyst for CO_2 reduction to formate.

Chem. Eng. J. **421**:129606. DOI:10.1016/j.cej.2021.129606

37. Jia L., Sun M., Xu J., et al. (2021). Phase-dependent electrocatalytic CO_2 reduction on Pd_3Bi nanocrystals. *Angew. Chem., Int. Ed.* **60**:21741-21745. DOI:10.1002/anie.202109288

38. Wang M., Liu S., Chen B., et al. (2023). Co-regulation of intermediate binding and water activation in sulfur-doped bismuth nanosheets for electrocatalytic CO_2 reduction to formate. *Chem. Eng. J.* **451**:139056. DOI:10.1016/j.cej.2022.139056

39. Wu Z., Jing H., Zhao Y., et al. (2023). Grain boundary and interface interaction Co-regulation promotes SnO_2 quantum dots for efficient CO_2 reduction. *Chem. Eng. J.* **451**:138477. DOI:10.1016/j.cej.2022.138477

40. Lv L., Lu R., Zhu J., et al. (2023). Coordinating the edge defects of bismuth with sulfur for enhanced CO_2 electroreduction to formate. *Angew. Chem., Int. Ed.* **62**:e202303117. DOI:10.1002/anie.202303117

41. Yang Y., Fu J. j., Tang T., et al. (2022). Regulating surface In–O in In@InO_x core-shell nanoparticles for boosting electrocatalytic CO_2 reduction to formate. *Chin. J. Catal.* **43**:1674-1679. DOI:10.1016/S1872-2067(21)63943-8

42. Zhang Y., Lan J., Xie F., et al. (2022). Aligned InS nanorods for efficient electrocatalytic carbon dioxide reduction. *ACS Appl. Mater. Interfaces* **14**:25257-25266. DOI:10.1021/acsami.2c01152

43. Gao N., Wang F., Ding J., et al. (2022). Intercalated gold nanoparticle in 2D palladium nanosheet avoiding CO poisoning for formate production under a wide potential window. *ACS Appl. Mater. Interfaces* **14**:10344-10352. DOI:10.1021/acsami.1c23430

44. Zhang Y., Chen Y., Liu R., et al. (2023). Oxygen vacancy stabilized $Bi_2O_2CO_3$ nanosheet for CO_2 electroreduction at low overpotential enables energy efficient CO-production of formate. *InfoMat* **5**:e12375. DOI:10.1002/inf2.12375

45. Yang Q., Zhao Y., Meng L., et al. (2022). Nanoporous intermetallic SnTe enables efficient electrochemical CO_2 reduction into formate via promoting the fracture of metal–oxygen bonding. *Small* **18**:2107968.

DOI:10.1002/smll.202107968

46. Wang Z., Zu X., Li X., et al. (2022). Industrial-current-density CO_2-to-formate conversion with low overpotentials enabled by disorder-engineered metal sites. *Nano Res.* **15**:6999-7007. DOI:10.1007/s12274-022-4335-1

47. Sui P. F., Gao M. R., Liu S., et al. (2022). Carbon dioxide valorization via formate electrosynthesis in a wide potential window. *Adv. Funct. Mater.* **32**:2203794. DOI:10.1002/adfm.202203794

48. Zhang J., Fan T., Huang P., et al. (2022). Electro-reconstruction-induced strain regulation and synergism of Ag-In-S toward highly efficient CO_2 electrolysis to formate. *Adv. Funct. Mater.* **32**:2113075. DOI:10.1002/adfm.202113075

49. Huang W., Wang Y., Liu J., et al. (2022). Efficient and selective CO_2 reduction to formate on Pd-doped $Pb_3(CO_3)_2(OH)_2$: dynamic catalyst reconstruction and accelerated CO_2 protonation. *Small* **18**:2107885. DOI:10.1002/smll.202107885

50. Yang W., Si C., Zhao Y., et al. (2022). Activating inert antimony for selective CO_2 electroreduction to formate via bimetallic interactions. *Appl. Catal., B* **316**:121619. DOI:10.1016/j.apcatb.2022.121619

51. Shen H., Zhao Y., Zhang L., et al. (2023). In-situ constructuring of copper-doped bismuth catalyst for highly efficient CO_2 electrolysis to formate in ampere-level. *Adv. Energy Mater.* **13**:2202818. DOI:10.1002/aenm.202202818

52. Ko Y. J., Kim J. Y., Lee W. H., et al. (2022). Exploring dopant effects in stannic oxide nanoparticles for CO_2 electro-reduction to formate. *Nat. Commun.* **13**:2205. DOI:10.1038/s41467-022-29783-7

53. Bi J., Li P., Liu J., et al. (2023). High-rate CO_2 electrolysis to formic acid over a wide potential window: an electrocatalyst comprised of indium nanoparticles on chitosan-derived graphene. *Angew. Chem., Int. Ed.* **62**:e202307612. DOI:10.1002/anie.202307612

54. Wu H., Song J., Xie C., et al. (2018). Design of naturally derived lead phytate as an electrocatalyst for highly efficient CO_2 reduction to formic acid. *Green Chem.* **20**:4602-4606. DOI:10.1039/C8GC02457J

55. Yang S., Jiang M., Zhang W., et al. (2023). In situ structure refactoring of

bismuth nanoflowers for highly selective electrochemical reduction of CO_2 to formate. *Adv. Funct. Mater.* **33**:2301984. DOI:10.1002/adfm.202301984

56. Zhang Z., Dou H., Gao R., et al. (2022). Steering carbon hybridization state in carbon-based metal-free catalysts for selective and durable CO_2 electroreduction. *ACS Catal.* **12**:15218-15229. DOI:10.1021/acscatal.2c03055

57. Li K., Xu J., Zheng T., et al. (2022). In situ dynamic construction of a copper tin sulfide catalyst for high-performance electrochemical CO_2 conversion to formate. *ACS Catal.* **12**:9922-9932. DOI:10.1021/acscatal.2c02627

58. Wang L., Xu Y., Chen T., et al. (2021). Ternary heterostructural CoO/CN/Ni catalyst for promoted CO_2 electroreduction to methanol. *J. Catal.* **393**:83-91. DOI:10.1016/j.jcat.2020.11.012

59. Zhao Q., Zhang C., Hu R., et al. (2021). Selective etching quaternary MAX phase toward single atom copper immobilized MXene ($Ti_3C_2Cl_x$) for efficient CO_2 electroreduction to methanol. *ACS Nano* **15**:4927-4936. DOI:10.1021/acsnano.0c09755

60. Sun X., Zhu Q., Kang X., et al. (2016). Molybdenum–bismuth bimetallic chalcogenide nanosheets for highly efficient electrocatalytic reduction of carbon dioxide to methanol. *Angew. Chem., Int. Ed.* **55**:6771-6775. DOI:10.1002/anie.201603034

61. Zhang W., Qin Q., Dai L., et al. (2018). Electrochemical reduction of carbon dioxide to methanol on hierarchical Pd/SnO_2 nanosheets with abundant Pd–O–Sn interfaces. *Angew. Chem., Int. Ed.* **57**:9475-9479. DOI:10.1002/anie.201804142

62. Mou S., Wu T., Xie J., et al. (2019). Boron phosphide nanoparticles: a nonmetal catalyst for high-selectivity electrochemical reduction of CO_2 to CH_3OH. *Adv. Mater.* **31**:1903499. DOI:10.1002/adma.201903499

63. Periasamy A. P., Ravindranath R., Senthil Kumar S. M., et al. (2018). Facet- and structure-dependent catalytic activity of cuprous oxide/polypyrrole particles towards the efficient reduction of carbon dioxide to methanol. *Nanoscale* **10**:11869-11880. DOI:10.1039/C8NR02117A

64. Huang J., Hu Q., Guo X., et al. (2018). Rethinking $Co(CO_3)_{0.5}(OH)\cdot 0.11H_2O$: a new property for highly selective electrochemical reduction of carbon dioxide to methanol in aqueous solution. *Green Chem.* **20**:2967-2972. DOI:10.1039/C7GC03744A
65. Yang D., Zhu Q., Chen C., et al. (2019). Selective electroreduction of carbon dioxide to methanol on copper selenide nanocatalysts. *Nat. Commun.* **10**:677. DOI:10.1038/s41467-019-08653-9
66. Yang B., Liu K., Li H., et al. (2022). Accelerating CO_2 electroreduction to multicarbon products via synergistic electric–thermal field on copper nanoneedles. *J. Am. Chem. Soc.* **144**:3039-3049. DOI:10.1021/jacs.1c11253
67. Payra S., Shenoy S., Chakraborty C., et al. (2020). Structure-sensitive electrocatalytic reduction of CO_2 to methanol over carbon-supported intermetallic PtZn nano-alloys. *ACS Appl. Mater. Interfaces* **12**:19402-19414. DOI:10.1021/acsami.0c00521
68. Wu Y., Jiang Z., Lu X., et al. (2019). Domino electroreduction of CO_2 to methanol on a molecular catalyst. *Nature* **575**:639-642. DOI:10.1038/s41586-019-1760-8
69. Yang X., Cheng J., Yang X., et al. (2022). MOF-derived Cu@Cu_2O heterogeneous electrocatalyst with moderate intermediates adsorption for highly selective reduction of CO_2 to methanol. *Chem. Eng. J.* **431**:134171. DOI:10.1016/j.cej.2021.134171
70. Liang Z., Wang J., Tang P., et al. (2022). Molecular engineering to introduce carbonyl between nickel salophen active sites to enhance electrochemical CO_2 reduction to methanol. *Appl. Catal., B* **314**:121451. DOI:10.1016/j.apcatb.2022.121451
71. Yang H., Wu Y., Li G., et al. (2019). Scalable production of efficient single-atom copper decorated carbon membranes for CO_2 electroreduction to methanol. *J. Am. Chem. Soc.* **141**:12717-12723. DOI:10.1021/jacs.9b04907
72. Lu L., Sun X., Ma J., et al. (2018). Highly efficient electroreduction of CO_2 to methanol on palladium–copper bimetallic aerogels. *Angew. Chem., Int. Ed.*

57:14149-14153. DOI:10.1002/anie.201808964

73. Guo W., Liu S., Tan X., et al. (2021). Highly efficient CO_2 electroreduction to methanol through atomically dispersed Sn ccoupled with defective CuO catalysts. *Angew. Chem., Int. Ed.* **60**:21979-21987. DOI:10.1002/anie.202108635

74. Azenha C., Mateos-Pedrero C., Alvarez-Guerra M., et al. (2020). Enhancement of the electrochemical reduction of CO_2 to methanol and suppression of H_2 evolution over CuO nanowires. *Electrochim. Acta* **363**:137207. DOI:10.1016/j.electacta.2020.137207

75. Chi C., Duan D., Zhang Z., et al. (2020). Mo–Bi bimetallic chalcogenide nanoparticles supported on CNTs for the efficient electrochemical reduction of CO_2 to methanol. Coatings.

76. Jiwanti P. K., Natsui K., Nakata K., et al. (2016). Selective production of methanol by the electrochemical reduction of CO_2 on boron-doped diamond electrodes in aqueous ammonia solution. *RSC Adv.* **6**:102214-102217. DOI:10.1039/C6RA20466J

77. Song Y., Guo P., Ma T., et al. (2023). Ultrathin, cationic covalent organic nanosheets for enhanced CO_2 electroreduction to methanol. *Adv. Mater.* **36**:2310037. DOI:10.1002/adma.202310037

78. Su J. J., Musgrave C. B., Song Y., et al. (2023). Strain enhances the activity of molecular electrocatalysts via carbon nanotube supports. *Nat. Catal.* **6**:818-828. DOI: 10.1038/s41929-023-01005-3

79. Chang F., Wei J., Liu Y., et al. (2023). Surface/interface reconstruction in-situ on Cu_2O catalysts with high exponential facets toward enhanced electrocatalysis CO_2 reduction to C_{2+} products. *Appl. Surf. Sci.* **611**:155773. DOI:10.1016/j.apsusc.2022.155773

80. Cai F., Hu X., Gou F., et al. (2023). Ultrathin $ZnIn_2S_4$ nanosheet arrays activated by nitrogen-doped carbon for electrocatalytic CO_2 reduction reaction toward ethanol. *Appl. Surf. Sci.* **611**:155696. DOI:10.1016/j.apsusc.2022.155696

81. Zang D., Gao X. J., Li L., et al. (2022). Confined interface engineering of self-

supported Cu@N-doped graphene for electrocatalytic CO_2 reduction with enhanced selectivity towards ethanol. *Nano Res.* **15**:8872-8879. DOI:10.1007/s12274-022-4698-3

82. Yang B., Chen L., Xue S., et al. (2022). Electrocatalytic CO_2 reduction to alcohols by modulating the molecular geometry and Cu coordination in bicentric copper complexes. *Nat. Commun.* **13**:5122. DOI: 10.1038/s41467-022-32740-z

83. Wang P., Yang H., Tang C., et al. (2022). Boosting electrocatalytic CO_2-to-ethanol production via asymmetric C–C coupling. *Nat. Commun.* **13**:3754. DOI:10.1038/s41467-022-31427-9

84. Sun W., Wang P., Jiang Y., et al. (2022). V-doped Cu_2Se hierarchical nanotubes enabling flow-cell CO_2 electroreduction to ethanol with high efficiency and selectivity. *Adv. Mater.* **34**:2207691. DOI:10.1002/adma.202207691

85. Shang L., Lv X., Zhong L., et al. (2022). Efficient CO_2 electroreduction to ethanol by Cu_3Sn catalyst. *Small Methods* **6**:2101334. DOI:10.1002/smtd.202101334

86. Peng C., Yang S., Luo G., et al. (2022). Surface Co-modification of halide anions and potassium cations promotes high-rate CO_2-to-ethanol electrosynthesis. *Adv. Mater.* **34**:2204476. DOI:10.1002/adma.202204476

87. Liu Y., Yang H., Fan X., et al. (2022). Promoting electrochemical reduction of CO_2 to ethanol by B/N-doped sp^3/sp^2 nanocarbon electrode. *Chin. Chem. Lett.* **33**:4691-4694. DOI:10.1016/j.cclet.2021.12.063

88. Guo C., Guo Y., Shi Y., et al. (2022). Electrocatalytic reduction of CO_2 to ethanol at close to theoretical potential via engineering abundant electron-donating $Cu^{\delta+}$ species. *Angew. Chem., Int. Ed.* **61**:e202205909. DOI:10.1002/anie.202205909

89. Fu Y., Xie Q., Wan L., et al. (2022). Ethanol assisted cyclic voltammetry treatment of copper for electrochemical CO_2 reduction to ethylene. *Mater. Today Energy* **29**:101105. DOI:10.1016/j.mtener.2022.101105

90. Esmaeilirad M., Kondori A., Shan N., et al. (2022). Efficient electrocatalytic

conversion of CO_2 to ethanol enabled by imidazolium-functionalized ionomer confined molybdenum phosphide. *Appl. Catal., B* **317**:121681. DOI:10.1016/j.apcatb.2022.121681

91. Yang Q., Liu X., Peng W., et al. (2021). Vanadium oxide integrated on hierarchically nanoporous copper for efficient electroreduction of CO_2 to ethanol. *J. Mater. Chem. A* **9**:3044-3051. DOI:10.1039/D0TA09522B

92. Kim C., Cho K. M., Park K., et al. (2021). Cu/Cu_2O interconnected porous aerogel catalyst for highly productive electrosynthesis of ethanol from CO_2. *Adv. Funct. Mater.* **31**:2102142. DOI:10.1002/adfm.202102142

93. Xu H., Rebollar D., He H., et al. (2020). Highly selective electrocatalytic CO_2 reduction to ethanol by metallic clusters dynamically formed from atomically dispersed copper. *Nat. Energy* **5**:623-632. DOI:10.1038/s41560-020-0666-x

94. Gu Z., Shen H., Chen Z., et al. (2021). Efficient electrocatalytic CO_2 reduction to C_{2+} alcohols at defect-site-rich Cu surface. *Joule* **5**:429-440. DOI:10.1016/j.joule.2020.12.011

95. Zhang T., Yuan B., Wang W., et al. (2023). Tailoring *H intermediate coverage on the $CuAl_2O_4$/CuO catalyst for enhanced electrocatalytic CO_2 reduction to ethanol. *Angew. Chem., Int. Ed.* **62**:e202302096. DOI:10.1002/anie.202302096

96. Zhang K., Wang J., Zhang W., et al. (2023). Regulated surface electronic states of CuNi nanoparticles through metal-support interaction for enhanced electrocatalytic CO_2 reduction to ethanol. *Small* **19**:2300281. DOI:10.1002/smll.202300281

97. Yang Y., Fu J., Ouyang Y., et al. (2023). In-situ constructed Cu/CuNC interfaces for low-overpotential reduction of CO_2 to ethanol. *Natl. Sci. Rev.* **10**:nwac248. DOI:10.1093/nsr/nwac248

98. Yuan J., Yang M.-P., Zhi W.-Y., et al. (2019). Efficient electrochemical reduction of CO_2 to ethanol on Cu nanoparticles decorated on N-doped graphene oxide catalysts. *J. CO_2 Util.* **33**:452-460. DOI:10.1016/j.jcou.2019.07.014

99. Zhang T., Yuan B., Wang W., et al. (2023). Tailoring *H intermediate coverage on the $CuAl_2O_4$/CuO catalyst for enhanced electrocatalytic CO_2 reduction to

ethanol. *Angewandte Chemie International Edition* **62**:e202302096. DOI:10.1002/anie.202302096

100. Cai Z., Cao N., Zhang F., et al. (2023). Hierarchical Ag-Cu interfaces promote C−C coupling in tandem CO_2 electroreduction. *Appl. Catal., B* **325**:122310. DOI:10.1016/j.apcatb.2022.122310

101. Hu F., Yang L., Jiang Y., et al. (2021). Ultrastable Cu catalyst for CO_2 electroreduction to multicarbon liquid fuels by tuning C–C coupling with CuTi subsurface. *Angew. Chem., Int. Ed.* **60**:26122-26127. DOI:10.1002/anie.202110303

102. Li F., Li Y. C., Wang Z., et al. (2020). Cooperative CO_2-to-ethanol conversion via enriched intermediates at molecule–metal catalyst interfaces. *Nat. Catal.* **3**:75-82. DOI:10.1038/s41929-019-0383-7

103. Zhang Y., Li K., Chen M., et al. (2020). Cu/Cu_2O nanoparticles supported on vertically ZIF-L-coated nitrogen-doped graphene nanosheets for electroreduction of CO_2 to ethanol. *ACS Appl. Nano Mater.* **3**:257-263. DOI:10.1021/acsanm.9b01935

104. Zhu W., Zhao K., Liu S., et al. (2019). Low-overpotential selective reduction of CO_2 to ethanol on electrodeposited Cu_xAu_y nanowire arrays. *J. Energy Chem.* **37**:176-182. DOI:10.1016/j.jechem.2019.03.030

105. Daiyan R., Saputera W. H., Zhang Q., et al. (2019). 3D heterostructured copper electrode for conversion of carbon dioxide to alcohols at low overpotentials. *Adv. Sustainable Syst.* **3**:1800064. DOI:10.1002/adsu.201800064

106. Liu Y., Zhang Y., Cheng K., et al. (2017). Selective electrochemical reduction of carbon dioxide to ethanol on a boron- and nitrogen-co-doped nanodiamond. *Angew. Chem., Int. Ed.* **56**:15607-15611. DOI:10.1002/anie.201706311

107. Zhu Q., Sun X., Yang D., et al. (2019). Carbon dioxide electroreduction to C_2 products over copper-cuprous oxide derived from electrosynthesized copper complex. *Nat. Commun.* **10**:3851. DOI:10.1038/s41467-019-11599-7

108. Tan D., Wulan B., Ma J., et al. (2023). Electrochemical-driven reconstruction for efficient reduction of carbon dioxide into alcohols. *Chem Catal.* **3**:100512.

DOI:10.1016/j.checat.2023.100512

109. Peng C., Luo G., Zhang J., et al. (2021). Double sulfur vacancies by lithium tuning enhance CO_2 electroreduction to n-propanol. *Nat. Commun.* **12**:1580. DOI:10.1038/s41467-021-21901-1

110. Zhuang T. T., Liang Z. Q., Seifitokaldani A., et al. (2018). Steering post-C–C coupling selectivity enables high efficiency electroreduction of carbon dioxide to multi-carbon alcohols. *Nat. Catal.* **1**:421-428. DOI:10.1038/s41929-018-0084-7

111. Liang Z. Q., Zhuang T. T., Seifitokaldani A., et al. (2018). Copper-on-nitride enhances the stable electrosynthesis of multi-carbon products from CO_2. *Nat. Commun.* **9**:3828. DOI:10.1038/s41467-018-06311-0

112. Kim D., Kley C. S., Li Y., et al. (2017). Copper nanoparticle ensembles for selective electroreduction of CO_2 to C_2–C_3 products. *Proc. Natl. Acad. Sci. U. S. A.* **114**:10560-10565. DOI:10.1073/pnas.1711493114

113. Rahaman M., Dutta A., Zanetti A., et al. (2017). Electrochemical reduction of CO_2 into multicarbon alcohols on activated Cu mesh catalysts: an identical location (IL) study. *ACS Catal.* **7**:7946-7956. DOI:10.1021/acscatal.7b02234

114. Ren D., Wong N. T., Handoko A. D., et al. (2016). Mechanistic insights into the enhanced activity and stability of agglomerated Cu nanocrystals for the electrochemical reduction of carbon dioxide to n-propanol. *J. Phys. Chem. Lett.* **7**:20-24. DOI:10.1021/acs.jpclett.5b02554

115. Li C. W. and Kanan M. W. (2012). CO_2 reduction at low overpotential on Cu electrodes resulting from the reduction of thick Cu_2O films. *J. Am. Chem. Soc.* **134**:7231-7234. DOI:10.1021/ja3010978

116. Rahaman M., Kiran K., Montiel I. Z., et al. (2020). Selective n-propanol formation from CO_2 over degradation-resistant activated PdCu alloy foam electrocatalysts. *Green Chem.* **22**:6497-6509. DOI:10.1039/D0GC01636E

117. Arán-Ais R. M., Scholten F., Kunze S., et al. (2020). The role of in situ generated morphological motifs and Cu^{I} species in C_{2+} product selectivity during CO_2 pulsed electroreduction. *Nat. Energy* **5**:317-325.

DOI:10.1038/s41560-020-0594-9

118. Li H., Qin X., Jiang T., et al. (2019). Changing the product selectivity for electrocatalysis of CO_2 reduction reaction on plated Cu electrodes. *ChemCatChem* **11**:6139-6146. DOI:10.1002/cctc.201901748

119. Romero Cuellar N. S., Wiesner-Fleischer K., Fleischer M., et al. (2019). Advantages of CO over CO_2 as reactant for electrochemical reduction to ethylene, ethanol and n-propanol on gas diffusion electrodes at high current densities. *Electrochim. Acta* **307**:164-175. DOI:10.1016/j.electacta.2019.03.142

120. Zhao Z. L. and Lu G. (2018). Computational screening of near-surface alloys for CO electroreduction. *ACS Catal.* **8**:3885-3894. DOI:10.1021/acscatal.7b03705

121. Meng Y., Li K., Xiao D., et al. (2020). High selective and efficient Fe_2–N_6 sites for CO_2 electroreduction: a theoretical investigation. *Int. J. Hydrogen Energy* **45**:14311-14319. DOI:10.1016/j.ijhydene.2020.03.134

122. Zhao C. C., Su X. F., Wang S., et al. (2022). Single-atom catalysts on supported silicomolybdic acid for CO electroreduction: a DFT prediction. *J. Mater. Chem. A* **10**:6178-6186. DOI:10.1039/d1ta08285j

123. Yang Y. J., Liu J., Wu D. W., et al. (2021). Two-dimensional pyrite supported transition metal for highly-efficient electrochemical CO reduction: a theoretical screening study. *Chem. Eng. J.* **424**. DOI:10.1016/j.cej.2021.130541

124. Xiong B., Yang Y. J., Liu J., et al. (2022). Electrocatalytic reduction of CO to C products over bimetal catalysts: a DFT screening study. *Fuel Process. Technol.* **233**:107315. DOI:10.1016/j.fuproc.2022.107315

125. Li C. Y., Liu X., Xu F., et al. (2022). High-throughput screening of dual-atom doped PC6 electrocatalysts for efficient CO_2 electrochemical reduction to CH_4 by breaking scaling relations. *Electrochim. Acta* **426**. DOI:10.1016/j.electacta.2022.140764

126. Xu F., Wang X. H., Liu X., et al. (2022). Computational screening of TMN based graphene-like BCN for CO electroreduction to C hydrocarbon products.

Mol. Catal. **530**:112571. DOI:10.1016/j.mcat.2022.112571

127. Wei X., Cao S., Wei S., et al. (2022). Theoretical investigation on electrocatalytic reduction of CO_2 to methanol and methane by bimetallic atoms TM_1/TM_{2-N}@Gra (TM = Fe, Co, Ni, Cu). *Appl. Surf. Sci.* **593**:153377. DOI:10.1016/j.apsusc.2022.153377

128. Back S., Kim H. and Jung Y. (2015). Selective heterogeneous CO_2 electroreduction to methanol. *ACS Catal.* **5**:965-971. DOI:10.1021/cs501600x

129. Back S., Lim J., Kim N. Y., et al. (2017). Single-atom catalysts for CO_2 electroreduction with significant activity and selectivity improvements. *Chem. Sci.* **8**:1090-1096. DOI:10.1039/c6sc03911a

130. Cui X., An W., Liu X., et al. (2018). C_2N-graphene supported single-atom catalysts for CO_2 electrochemical reduction reaction: mechanistic insight and catalyst screening. *Nanoscale* **10**:15262-15272. DOI:10.1039/c8nr04961k

131. Xiao Y., Shen C. and Hadaeghi N. (2021). Quantum mechanical screening of 2D MBenes for the electroreduction of CO_2 to C_1 hydrocarbon fuels. *J. Phys. Chem. Lett.* **12**:6370-6382. DOI:10.1021/acs.jpclett.1c01499

132. Bai X., Zhao Z. and Lu G. (2023). Breaking the scaling relationship on single-atom embedded MBene for selective CO_2 electroreduction. *J. Phys. Chem. Lett.* **14**:5172-5180. DOI:10.1021/acs.jpclett.3c00903

133. Behrendt D., Banerjee S., Clark C., et al. (2023). High-throughput computational screening of bioinspired dual-atom alloys for CO_2 activation. *J. Am. Chem. Soc.* **145**:4730-4735. DOI:10.1021/jacs.2c13253

134. Xing M., Zhang Y., Li S., et al. (2022). Prediction of carbon dioxide reduction catalyst using machine learning with a few-feature model: WLEDZ. *J. Phys.Chem. C* **126**:17025-17035. DOI:10.1021/acs.jpcc.2c02161

135. Roy D., Mandal S. C. and Pathak B. (2021). Machine learning-driven high-throughput screening of alloy-based catalysts for selective CO_2 hydrogenation to methanol. *ACS Appl. Mater. Interfaces* **13**:56151-56163. DOI:10.1021/acsami.1c16696

136. Roy D., Mandal S. C. and Pathak B. (2022). Machine learning assisted

exploration of high entropy alloy-based catalysts for selective CO_2 reduction to methanol. *J. Phys. Chem. Lett.* **13**:5991-6002. DOI:10.1021/acs.jpclett.2c00929

137. Zhu Q., Gu Y., Liang X., et al. (2022). A machine learning model to predict CO_2 reduction reactivity and products transferred from metal-zeolites. *ACS Catal.* **12**:12336-12348. DOI:10.1021/acscatal.2c03250

138. Yang Z., Gao W. and Jiang Q. (2020). A machine learning scheme for the catalytic activity of alloys with intrinsic descriptors. *J. Mater. Chem. A* **8**:17507-17515. DOI:10.1039/D0TA06203K

139. Yu L., Li F., Huang J., et al. (2023). Double-atom catalysts featuring inverse sandwich structure for CO_2 reduction reaction: a synergetic first-principles and machine learning investigation. *ACS Catal.* **13**:9616-9628. DOI:10.1021/acscatal.3c01584

140. Wu D., Zhang J., Cheng M. J., et al. (2021). Machine learning investigation of supplementary adsorbate influence on copper for enhanced electrochemical CO_2 reduction performance. *J. Phys.Chem. C* **125**:15363-15372. DOI:10.1021/acs.jpcc.1c05004

141. Gariepy Z., Chen G., Xu A., et al. (2023). Machine learning assisted binary alloy catalyst design for the electroreduction of CO_2 to C_2 products. *Energy Adv.* **2**:410-419. DOI:10.1039/D2YA00316C

142. Tran K. and Ulissi Z. W. (2018). Active learning across intermetallics to guide discovery of electrocatalysts for CO_2 reduction and H_2 evolution. *Nat. Catal.* **1**:696-703. DOI:10.1038/s41929-018-0142-1

143. Pedersen J. K., Batchelor T. A. A., Bagger A., et al. (2020). High-entropy alloys as catalysts for the CO_2 and CO reduction reactions. *ACS Catal.* **10**:2169-2176. DOI:10.1021/acscatal.9b04343

144. Chen A., Zhang X., Chen L., et al. (2020). A machine learning model on simple features for CO_2 reduction electrocatalysts. *J. Phys.Chem. C* **124**:22471-22478. DOI:10.1021/acs.jpcc.0c05964

145. Wang D., Cao R., Hao S., et al. (2023). Accelerated prediction of Cu-based single-atom alloy catalysts for CO_2 reduction by machine learning. *Green*

Energy Environ. **8**:820-830. DOI:10.1016/j.gee.2021.10.003

146. Singh M. R., Kwon Y., Lum Y., et al. (2016). Hydrolysis of electrolyte cations enhances the electrochemical reduction of CO_2 over Ag and Cu. *J. Am. Chem. Soc.* **138**:13006-13012. DOI:10.1021/jacs.6b07612
